# Reprogramming of *Yersinia* from Virulent to Persistent Mode Revealed by Complex *In Vivo* RNA-seq Analysis

**DOI:** 10.1371/journal.ppat.1004600

**Published:** 2015-01-15

**Authors:** Kemal Avican, Anna Fahlgren, Mikael Huss, Ann Kathrin Heroven, Michael Beckstette, Petra Dersch, Maria Fällman

**Affiliations:** 1 Department of Molecular Biology, Umeå Centre for Microbial Research (UCMR), Umeå University, Umeå, Sweden; 2 Department of Molecular Biology, Laboratory for Molecular Infection Medicine Sweden (MIMS), Umea University, Umeå, Sweden; 3 Science for Life Laboratory, Royal Institute of Technology, Solna, Sweden; 4 Department of Molecular Infection Biology, Helmholtz Centre for Infection Research, Braunschweig, Germany; Yale University School of Medicine, UNITED STATES

## Abstract

We recently found that *Yersinia pseudotuberculosis* can be used as a model of persistent bacterial infections. We performed *in vivo* RNA-seq of bacteria in small cecal tissue biopsies at early and persistent stages of infection to determine strategies associated with persistence. Comprehensive analysis of mixed RNA populations from infected tissues revealed that *Y. pseudotuberculosis* undergoes transcriptional reprogramming with drastic down-regulation of T3SS virulence genes during persistence when the pathogen resides within the cecum. At the persistent stage, the expression pattern in many respects resembles the pattern seen *in vitro* at 26oC, with for example, up-regulation of flagellar genes and *invA*. These findings are expected to have impact on future rationales to identify suitable bacterial targets for new antibiotics. Other genes that are up-regulated during persistence are genes involved in anaerobiosis, chemotaxis, and protection against oxidative and acidic stress, which indicates the influence of different environmental cues. We found that the Crp/CsrA/RovA regulatory cascades influence the pattern of bacterial gene expression during persistence. Furthermore, *arcA*, *fnr*, *frdA*, and *wrbA* play critical roles in persistence. Our findings suggest a model for the life cycle of this enteropathogen with reprogramming from a virulent to an adapted phenotype capable of persisting and spreading by fecal shedding.

## Author Summary

To establish infection and colonize within a host, infecting pathogens have to cope with a variety of destructive surroundings. The food-borne pathogen *Y. pseudotuberculosis* can cause persistent infection in mice. Upon infection, *Y. pseudotuberculosis* passes the anti-microbial gastrointestinal milieu and finally remains associated with lymphoid follicles in cecal tissue surrounded by polymorphonuclear leukocytes, indicating that the bacteria are exposed to multiple environmental cues. We performed complex RNA-seq of small cecal biopsies of infected mice to reveal *Y. pseudotuberculosis* gene expression *in vivo*. We found that *Y. pseudotuberculosis* underwent reprogramming from a virulent phenotype, expressing virulence genes during early infection, to an adapted phenotype capable of persisting in the harsh cecal environment. Persistence was characterized by a novel expression pattern with down-regulation of virulence genes and up-regulation of genes involved in anaerobiosis, chemotaxis, and protection against oxidative and acidic stress. Mutagenesis of selected genes revealed that the regulator *rovA* was critical for the establishment of infection, and that *arcA, fnr, frdA*, and *wrbA* play critical roles in maintaining infection for long periods of time. Our study shows the power of RNA deep sequencing, which can be used to reveal the *in vivo* expression patterns of small amounts of bacteria in complex intestinal environments.

## Introduction

*Yersinia pseudotuberculosis* is a food borne pathogen that can penetrate the intestinal epithelium and cause gastroenteritis. This enteropathogen invades lymphoid follicles of Peyer’s patches and cecum, where it survives extracellularly before breaking the barrier and becoming systemic [1,2]. All pathogenic *Yersinia* species are capable of inhibiting important host immune mechanisms in local lymph nodes, and this essential virulence property is dependent on the plasmid-encoded *Yersinia* outer proteins (Yops) YopE, YopH, YopJ, YopM, YopT, YpkA, and YopK. Upon intimate contact with a target host cell, the Yops are delivered into the host cell via the *Yersinia* type three secretion system (T3SS) [3]. Inside the target cell, the Yop effectors interfere with several key mechanisms of the host immune defense; for example, YopH and YopE inhibit phagocytosis and YopJ interferes with the production of pro-inflammatory signaling molecules [4]. Polymorphonuclear neutrophils (PMNs), which are rapidly recruited to infection sites, are the main target cells for *Y. pseudotuberculosis* T3SS-mediated Yop translocation during infection [2,5]. Current knowledge of *Y. pseudotuberculosis* virulence mechanisms is based, to a great extent, on studies using the acute mouse infection model in which infection results in systemic infection.

We recently found that the enteric pathogen *Y. pseudotuberculosis* can cause persistent infection in mice, where it persists associated with the lymphoid follicles of the cecum [6]. In this model, low dose oral infection (10^6^–10^7^ colony-forming units (CFUs)) leads to asymptomatic infection in 20–30% of infected mice with an observed infection duration as long as 115 days. Even if no signs of disease are present, *Y. pseudotuberculosis* persistence is associated with an immune response in which the *Y. pseudotuberculosis* foci are surrounded by PMNs and bacteria are shed in feces [6]. *Yersinia, Salmonella*, and *Campylobacter* have all been reported to infect and affect the ileocecal area in humans [7], suggesting that the cecum is a beneficial niche for bacterial persistence.

Many pathogenic bacteria are capable of maintaining infection in mammalian hosts, giving rise to persistent infections [8]. Diagnosis of a persistent infection can be difficult, as symptoms are not always obvious. Prolonged persistent infections can cause chronic inflammation, which can lead to complications, or even precipitation of certain diseases in susceptible hosts [9]. In addition, persistent bacterial infections are a major cause of the overuse of antibiotics in both humans and animal husbandry. Increasing evidence indicates that persistence contributes to the development and spread of antibiotic resistance [10]. Therefore, the identification of bacterial mechanisms involved in the development of persistent infections is of great interest. One well-established model of persistence is *Salmonella typhimurium*; upon infection of Nramp1-expressing mice, this intracellular pathogen can persist inside phagocytic cells in classical granuloma lesions in the spleen, liver, and mesenteric lymph nodes [11,12]. Another model of *S. typhimurium* is the infection of antibiotic-treated DBA/2 and 129Sv/Ev mice, which results in colitis and chronic cholangitis [13]. Similar to the *Y. pseudotuberculosis* persistence model, the colitis phase is associated with PMN infiltration into the cecum and bacterial shedding in the feces. Studies of *S. typhimurium* persistence using these models have shown a variety of factors that contribute to persistent infection [14], including effectors encoded by the pathogenicity islands SPI1 and SPI2, which are required for initial invasion and intracellular growth. Two SPI2 factors, Sse1 and SseK2, have been implicated as being important for later stages of infection, with Sse1 affecting host cell adhesion and migration. Different adhesive proteins and factors that protect against host-derived antimicrobial peptides and factors that aid in coping with oxidative and nitrosative stress have been found important for sustained colonization of *S. typhimurium* in the gastrointestinal tract, as well as systemic persistence [14].

The mechanisms enabling *Y. pseudotuberculosis* to persist in cecal tissue in the presence of immune cells for a prolonged period of time are largely unknown. Our previous study showed that the T3SS effectors YopH and YopE contributed to *Y. pseudotuberculosis* persistence in the cecum, likely by enabling initial colonization in the presence of phagocytic cells [6]. One way to understand mechanisms and metabolic traits that are important for *Y. pseudotuberculosis* persistence in the cecum is to identify the genes involved. Several methods have been used to identify genes induced *in vivo* during infection, such as *in vivo* expression technology (IVET) [15], signature-tagged mutagenesis (STM) [16], cDNA microarray analysis [17,18], and the recently developed RNA sequencing technology with massively parallel cDNA sequencing (RNA-seq) [19]. IVET and STM, which are both based on infections with bacterial libraries, are not suitable for *in vivo* studies of enteropathogenic *Yersinia* due to restricted clonal invasion of the intestinal tissue by this pathogen [20,21]. Furthermore, because the intestinal tract is colonized by the intestinal microflora and harbors many commensal bacteria, the reliability of DNA microarray is greatly diminished due to cross-reactions between species-specific probes on the microarray chips. In contrast, RNA-seq provides a promising approach for monitoring gene expression in a specific organism in the presence or absence of others. This method is sensitive and allows accurate discrimination between similar RNAs originating from different species, offering an excellent opportunity to reveal the gene expression patterns of pathogens within host tissues, even in heavily colonized environments, such as the intestine, stomach, and cecum.

In this study, we performed RNA-seq on *Y. pseudotuberculosis* YPIII in small cecal tissue biopsies from mice at early and persistent stages of infection to reveal mechanisms of importance for persistent infection. We found that the bacteria underwent substantial transcriptional reprogramming. Initially, genes encoded on the virulence plasmid, including T3SS and associated effectors, were highly up-regulated. At the persistent stage these genes were found to be down-regulated, and other genes were up-regulated, including those encoding for anaerobic growth, motility, protection against acidic and oxidative stress, and genes indicating envelope perturbation, suggesting adaptation to the harsh environment in cecal tissue.

## Results

### Heterogeneous RNA populations of *Y. pseudotuberculosis*-infected cecal tissues sequenced by RNA-seq

**Figure 1:**
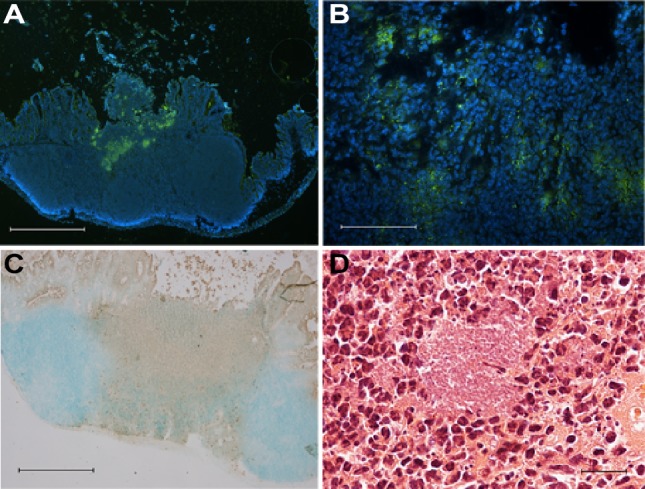
Persistent *Y. pseudotuberculosis* resides in cecal tissue in the presence of an immune response. (A-B) Immunofluorescent staining of *Y. pseudotuberculosis* in cecum from a mouse with persistent asymptomatic infection (35 dpi) using anti-*Yersiniae* rabbit polyclonal serum detected by anti-rabbit Al488 (green). Nuclei were stained with DAPI (blue); (A) 4× magnification, scale bar 500 μm, (B) 40× magnification, scale bar 50 μm. (C) Immunohistochemical staining of PMNs with anti-Ly6G6C in cecal tissue from a persistently infected asymptomatic mouse (35 dpi). Positive cells are brown (DAB) and the background is green. (methyl green). 4× magnification, scale bar 500 μm. (D) Hematoxylin-eosin staining of persistently infected cecal tissue (42 dpi). 60× magnification, scale bar 20 μm.

To identify mechanisms promoting *Y. pseudotuberculosis* persistence, RNA-seq was employed to determine the differential gene expression profiles of bacteria in the cecum during the early phase of infection and during persistence. To obtain infected tissue for the isolation of *Y. pseudotuberculosis* RNA, FVB/N mice were infected orally with bioluminescent wild-type (wt) bacteria at an infection dose of ∼2×10^7^ CFUs. The infection was monitored in real time by an *in vivo* imaging system (IVIS) at certain intervals for 42 days. In agreement with that reported earlier [6], we found bacterial foci associated with the cecal lymphoid tissue (Fig. 1A–B), massive infiltration of PMNs surrounding the bacterial foci (Fig. 1C–D) as well as superficial destruction of the epithelial lining and mixed inflammatory infiltrates.

**Figure 2:**
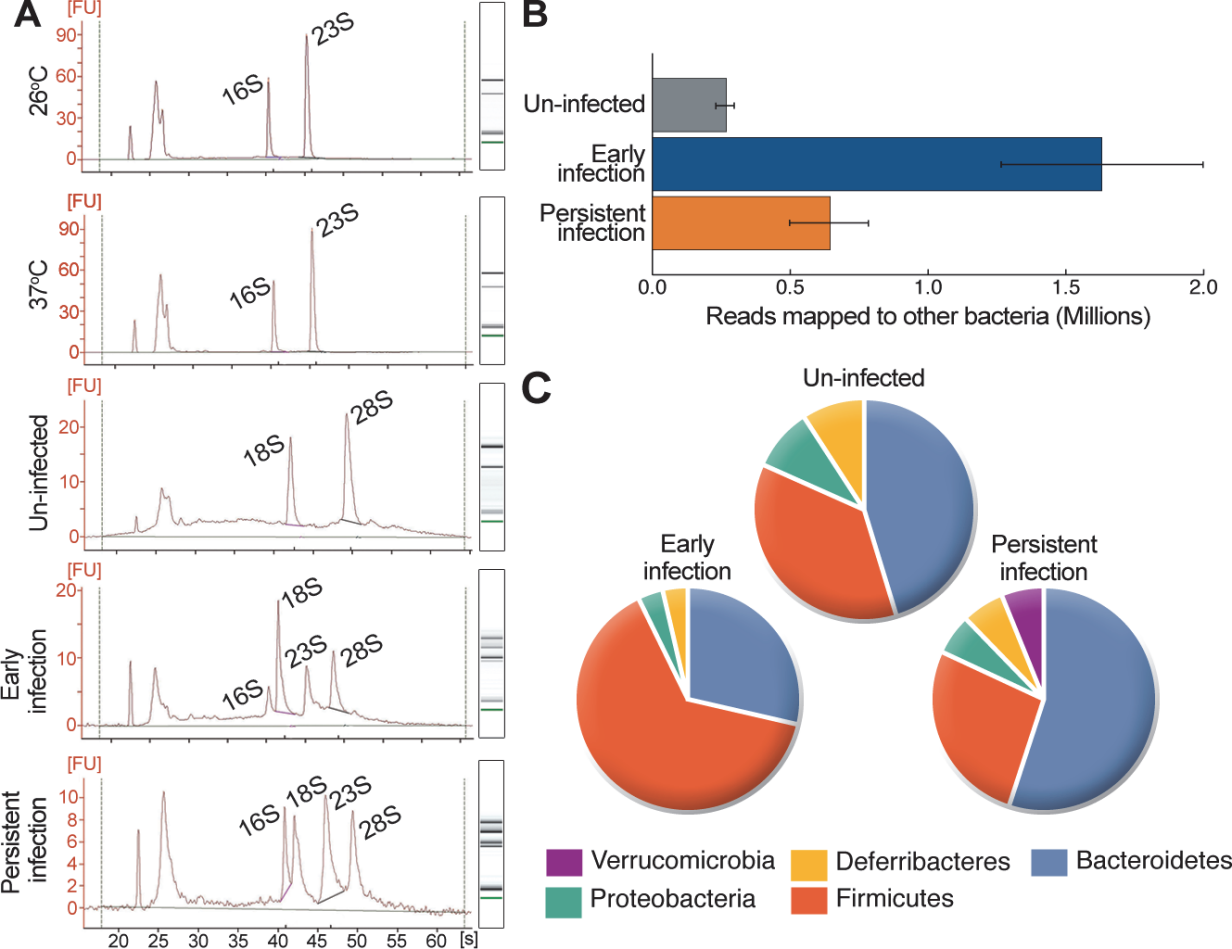
*Y. pseudotuberculosis* infection alters the bacterial composition of the cecum. (A) Representative Bioanalyzer 2100 electrographs and associated gel pictures for replicates of *in vitro*-derived RNA samples (grown at 26°C and 37°C), *in vivo*-derived samples of early (isolated from mouse cecal tissue 2 dpi) and persistent infection (isolated from mouse cecal tissue 42 dpi), and uninfected samples (isolated from uninfected mouse cecal tissue). (B) The number of reads mapping to 16S rRNA from different bacteria in non-depleted *in vivo*-derived samples. Data represent the mean ± SD of the two replicates for each sample group. (C) Relative abundance of different bacterial phyla in samples according to reads mapped to the 16SMicrobial database. The proportions are given as the percent of bacterial phyla identified in specific samples.

For sample preparation, isolated cecal tissues from 2 and 42 days post-infection (dpi) were analyzed by IVIS to verify that they contained *Yersinia*. The bioluminescent signal from *Y. pseudotuberculosis* allowed identification of the precise location of the bacteria in the tissue. Small biopsies (3 mm Ø) of cecal tissue containing bioluminescent bacteria were isolated using a hole punch. Total RNAs were extracted from biopsies from two mice infected for 2 days and two asymptomatic mice infected for 42 days. As a control, we extracted total RNAs from the cecal tissue of two un-infected mice. We also included RNA samples from bacteria grown in Luria broth (LB) *in vitro* at 26°C and in Ca^2+^-depleted LB at 37°C, a condition known to induce T3SS [22], hereafter referred to as T3SS-inducing conditions. The quality and quantity of all RNA samples were determined using an Agilent Bioanalyzer 2100, and all total RNA preparations had RIN values >7. This analysis revealed pure bacterial RNA in the *in vitro* samples (16S and 23S rRNAs) and mouse RNA (18S and 26S rRNAs) in samples from un-infected cecal tissue. As expected, both eukaryotic and prokaryotic RNAs were detected in the samples from infected cecal tissue and appeared as four distinct bands: 16S, 18S, 23S, and 26S rRNAs (Fig. 2A). However, as we previously recovered only 1×10^5^ to 2×10^6^ CFUs *Y. pseudotuberculosis* from cecal tissues [6], the amount of prokaryotic RNA was unexpectedly high in the infected tissues. Therefore, we performed qPCR to determine the *Yersinia* RNA abundance in infected tissues by comparing *ymoA* expression as an indication of *Yersinia* RNAs and *GAPDH* expression as an indication of host RNAs, finding ∼0.2% *Yersinia* RNA in the total RNA preparations. Therefore, the remarkably higher amount of prokaryotic RNA compared to that predicted for *Yersinia* RNA was assumed to reflect the presence of other microbial inhabitants in the samples.

### *Y. pseudotuberculosis* infection alters the bacterial composition of the cecum

To identify other bacteria in the cecum during the two different phases of infection, the *in vivo*-derived total RNA samples were analyzed by RNA-seq. The sequencing reads from these samples were mapped initially to the NCBI 16SMicrobial database with a full alignment parameter in order to map only unique reads to each 16S rRNA sequence. Next, matched 16S rRNA sequences were filtered with at least 80% coverage due to conserved regions in the 16S rRNA sequences. The number of reads mapped to each sample was normalized to the depth of sequencing in order to estimate the relative bacterial load in the tissue samples. Compared to un-infected samples, the bacterial RNA content of samples from early infection was 5.2-fold higher, and from persistent infection 3.7-fold higher, according to the normalized ratio of mapped reads for each tissue sample (Fig. 2B and S1 Table). The presence of other bacteria in uninfected samples likely reflects luminal bacteria, whereas the greater amount of bacteria in infected cecum samples may indicate that the infection leads to dysregulation of the luminal microbiota and/or that luminal bacteria gained access to the tissue. We identified 11 species in uninfected samples, 30 in early infection samples, and 11 in persistent infection samples (S1 Table). The identified species were grouped by phylum using RDP Classifier [23]. The abundance and composition of bacterial phyla in uninfected samples were similar to previous reports [24,25] but differed in samples from infected cecums. Samples from early infection (*Firmicutes* 60%, *Bacteroidetes* 27%, *Proteobacteria* 3.3%) differed from samples from persistent infection (*Firmicutes* 27%, *Bacteroidetes* 55%, *Proteobacteria* 9%, *Verrucomicrobia* 9%; Fig. 2C. The two independent replicates of the persistent sample contained similar species, and the strictly anaerobic Gram-negative bacterium *Akkermansia muciniphila* [26] was present in high abundance (75% of its transcriptome was revealed by RNA-seq; S1 Fig.).

### Mapping *Y. pseudotuberculosis*-specific reads in mixed populations of cDNAs

Given the relatively small amount of *Y. pseudotuberculosis* in the cecal tissue, samples were enriched for bacterial mRNA by depleting fractions of poly(A)-tagged RNAs, rRNAs, and tRNAs prior to RNA-seq. The enrichment procedure was performed with both *in vivo* and *in vitro* total RNA samples.

**Figure 3:**
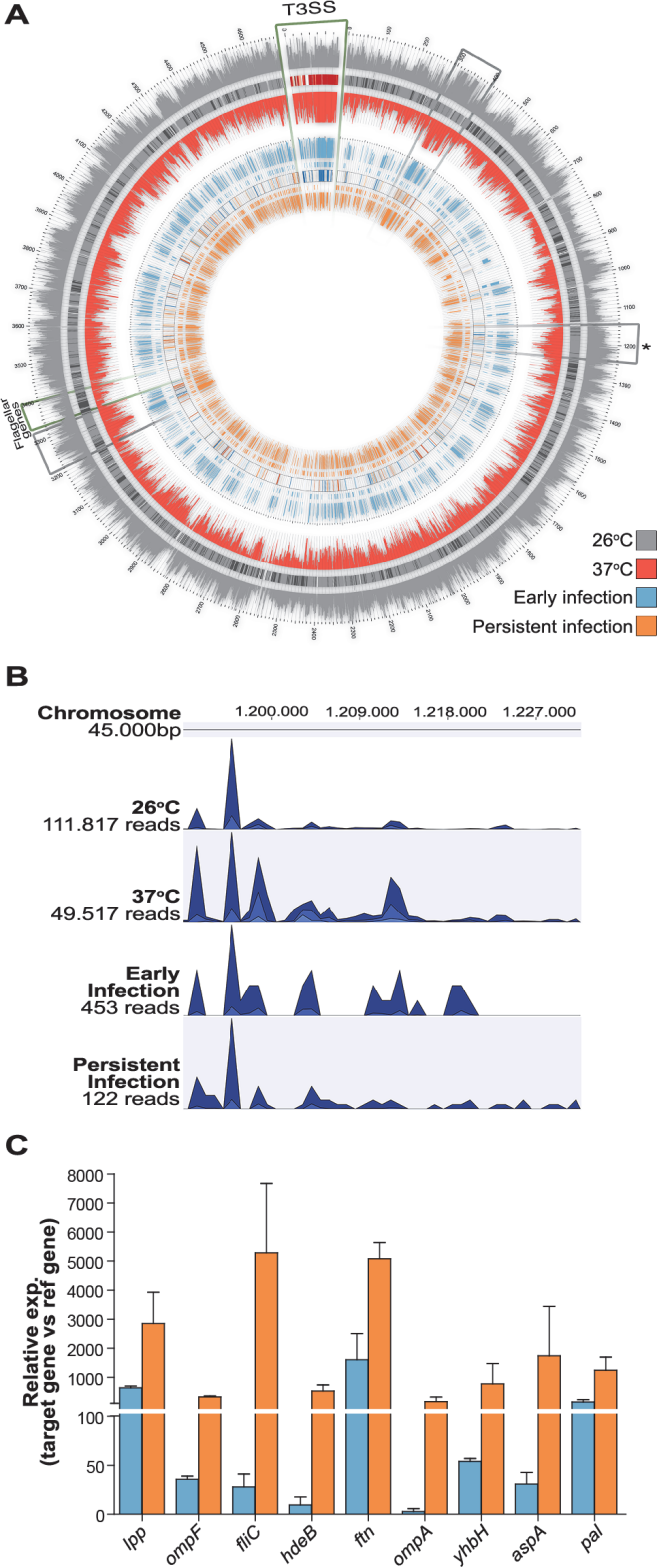
*In vivo Y. pseudotuberculosis* gene expression revealed by RNA-seq. (A) Gene expression data for *Y. pseudotuberculosis* from two biological replicates of *in vitro* and *in vivo*-derived samples. From outside to inside, the 10 circles in the plot correspond to: (1) a histogram of RPKMO values for each gene expressed at 26°C; (2) genes expressed only at 26°C under *in vitro* conditions; (3) heat map (combined gray and red Brewer palettes) of the log2 difference in RPKMO values for genes expressed at both 26°C and 37°C; (4) genes expressed only at 37°C under *in vitro* conditions; (5) histogram of RPKMO values for each gene expressed at 37°C; (6) histogram of RPKMO values for each gene expressed during the early phase of infection (2 dpi) in FVB/N cecum; (7) genes expressed only during the early phase of infection in FVB/N cecum; (8) heat map (combined blue and orange Brewer palettes) of the log2 difference in RPKMO values for genes expressed during both the early phase of infection and persistent infection (42 dpi) in the cecum; (9) genes expressed only during persistent infection in the FVB/N cecum; and (10) a histogram of RPKMO values for each gene expressed during persistent infection in FVB/N cecum. Regions outlined with green borders indicate the locations of genes encoding T3SS components and effectors of the virulence plasmid and genes involved in flagellar assembly on the chromosome. Regions outlined with gray borders indicate the locations of transcriptionally active regions. The asterisk indicates a chromosomal region shown in Fig. 3B. The plots were created using Circos [62]. (B) Distributions of reads mapped to a specific transcriptionally active region on the *Y. pseudotuberculosis* YPIII chromosome (from 1,191 Mb to 1,230 Mb) in one replicate of each sample group. The height of each peak corresponds to the relative number of reads mapped to the region. The tracks were created using CLC Genomic Workbench. (C). Expression of indicated genes during early (blue; 2 dpi) and persistent infections (orange; 42 dpi) determined by qPCR of cDNAs from two biological and three technical replicates for each gene. *lpp, ompF, fliC, hdeB, ftn, ompA, yhbH, aspA* and *pal* gene expressions were 1.1, 6.8, 9.2, 4.8, 9.7, 1.7, 2.3, 1.1 and 1.1 log2fold upregulated respectively during persistent infection in RNA-seq analysis.

Rigorous validations on mapped reads were required due to the presence of RNAs from other microbial inhabitants and homologous mRNAs that did not represent true *Y. pseudotuberculosis* transcripts. The genomes available for bacterial species found in the *in vivo* samples (42 genomes) were used as reference to optimize the alignment parameters for sequencing reads. Eventually, the optimization trials ended with a strict criterion of 95% alignment specificity to retrieve reads uniquely mapped to *Y. pseudotuberculosis* in the presence of other bacterial genomes. Finally, the uniqueness of these reads was double-checked with probabilistic variant detection, which searches for single nucleotide polymorphisms (SNPs) using CLC Genomic Workbench. With sample enrichment, optimized alignment parameters, and high sequencing depth, we revealed ∼92% of the metatranscriptome, which was composed of mouse and 42 other bacterial species in addition to *Y. pseudotuberculosis*. The reads mapped to *Y. pseudotuberculosis* were used in the subsequent RNA-seq analysis in order to calculate the expression of each open reading frame (ORF). The range of reads per ORF for *in vitro* and *in vivo*-derived samples was up to more than 200,000 and 490, respectively. Therefore, fewer ORFs were mapped (1551 ORFs, 36% transcriptome coverage) *in vivo* than *in vitro*, which had complete coverage (Table 1). Because we reached full coverage of the mouse transcriptome, and in some cases up to 75% coverage of other bacterial transcriptomes in the *in vivo*-derived samples (S1 Fig.), the relatively low coverage of *Y. pseudotuberculosis* was due to the very low abundance of its transcripts. Nevertheless, the RNA-seq results for *Y. pseudotuberculosis* showed very high correlation (Pearson and Spearman correlation *R*-values ≥0.98) of normalized RPKMO values (i.e., reads per kilobase pairs of a gene per million reads aligning to annotated ORFs) between biological replicates of all samples, which verifies the robustness of the analysis (Table 1). In addition, transcriptionally active regions were found to be the same for both *in vitro* and *in vivo*-derived samples (Fig. 3A, highlighted with gray borders). Even though the number of reads varied (53 to 111,817 reads) between different *in vivo* and *in vitro* samples, the distributions of the reads were similar for active regions (Fig. 3B). As qPCR requires more starting material than RNA-seq, we tested the differential expression of 9 genes that were highly expressed during persistent infection. The qPCR analysis confirmed the differential expression of all tested genes (Fig. 3C). Thus, the RNA-seq results for the *in vivo* samples provided valid information about differentially expressed genes in *Y. pseudotuberculosis* during early versus persistent infection in mice.

**Table 1:** Summary of RNA-seq reads.

**Sample**	**Biological replicates**	**Sequenced reads (millions)**	**mRNA aligned reads**	**Non-zero mRNAs**	**R-value**
**26°C**	a	20	3.6 million	4214	1
	b	20	3.9 million		
**37°C**	a	18	4.2 million	4283	0.99
	b	23	4.1 million		
**Early infection**	a	141	3708	841	0.98
	b	176	3125		
	a*	175	0	0	N/A
	b*	246	0		
**Persistent infection**	a	194	3144	1195	0.98
	b	193	4415		
	a*	150	0	0	N/A
	b*	132	0		
**Un-infected**	a*	193	0	0	N/A
	b*	236	0		

Asterisks indicate samples without rRNA depletion.

### *In vivo*-based RNA-seq disclosed transcriptional reprogramming with repression of T3SS and induction of motility during persistence

RNA-seq analysis of the *in vitro* samples revealed that 665 genes were differentially expressed (log2 fold change ≥0.7, p <0.05) under the different *in vitro* growth conditions. A total of 146 genes were up-regulated at 37°C (T3SS-inducing conditions), 55 of which were located on the 70-kb virulence plasmid that encodes the T3SS components; 519 genes, including 29 flagellar genes, were up-regulated at 26°C. This confirmation of T3SS induction by the temperature shift to 37°C, combined with Ca^2+^-depletion [22] and the motile phenotype of *Y. pseudotuberculosis* at 26°C [27], verifies the reliability of the RNA-seq analysis. The global expression patterns of cultured bacteria at 26°C and 37°C are shown on a histogram and heat map in Fig. 3A (see also S2 Table).

**Figure 4:**
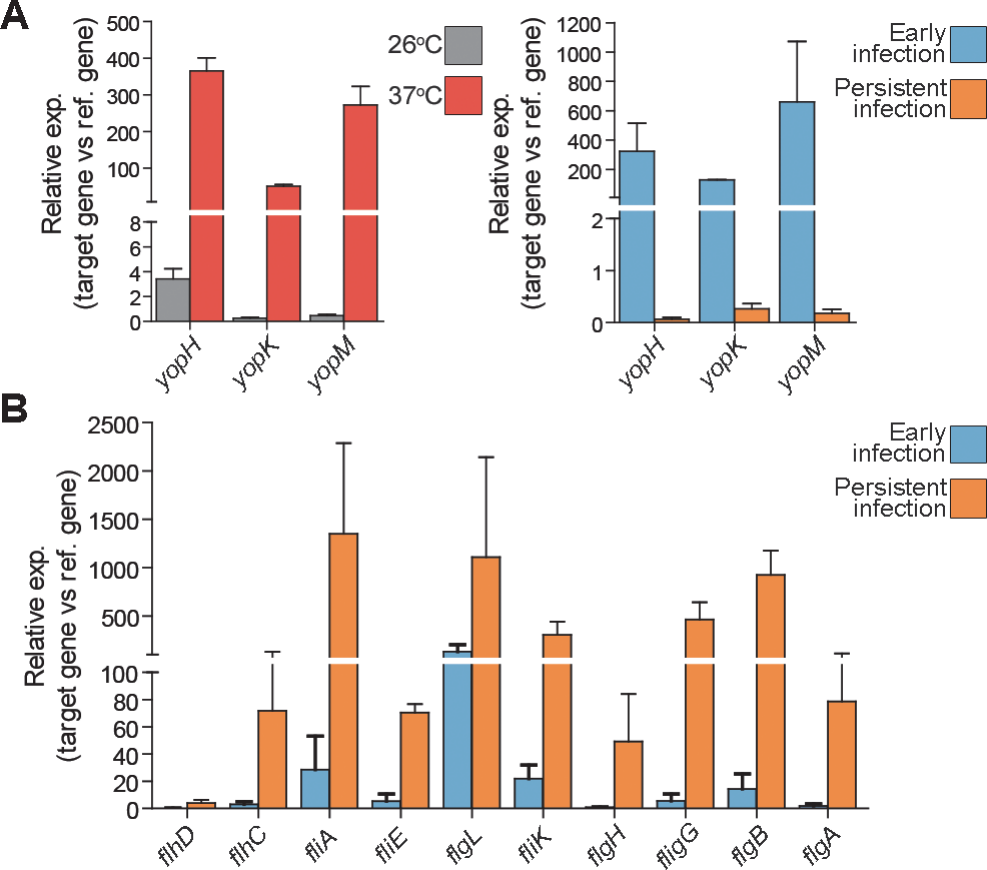
T3SS genes and flagellar genes are differentially regulated during persistent infection. (A) Expression of indicated yop effectors *in vitro* (left) at 26°C (gray) and at 37°C inducing conditions (red), and *in vivo* (right) during early (2 dpi; blue) and persistent infections (42 dpi; orange) as determined by qPCR of cDNAs from two biological and three technical replicates for each gene. (B) Expression of indicated flagellar genes *in vivo* during early (2 dpi; blue) and persistent infection (42 dpi; orange) as determined by qPCR. All qPCR analyses were performed with cDNAs from two biological and three technical replicates. *flhDC, fliA, fliE, flgL, fliK, flgH, flgG, flgB*, and *flgA* genes expressions were 5.2, 8.3, 9.9, 3.6, 2.3, 3.8, 3.1, 5.2 and 3.6 log2fold upregulated respectively during persistent infection according to RNA-seq analysis.

**Figure 5:**
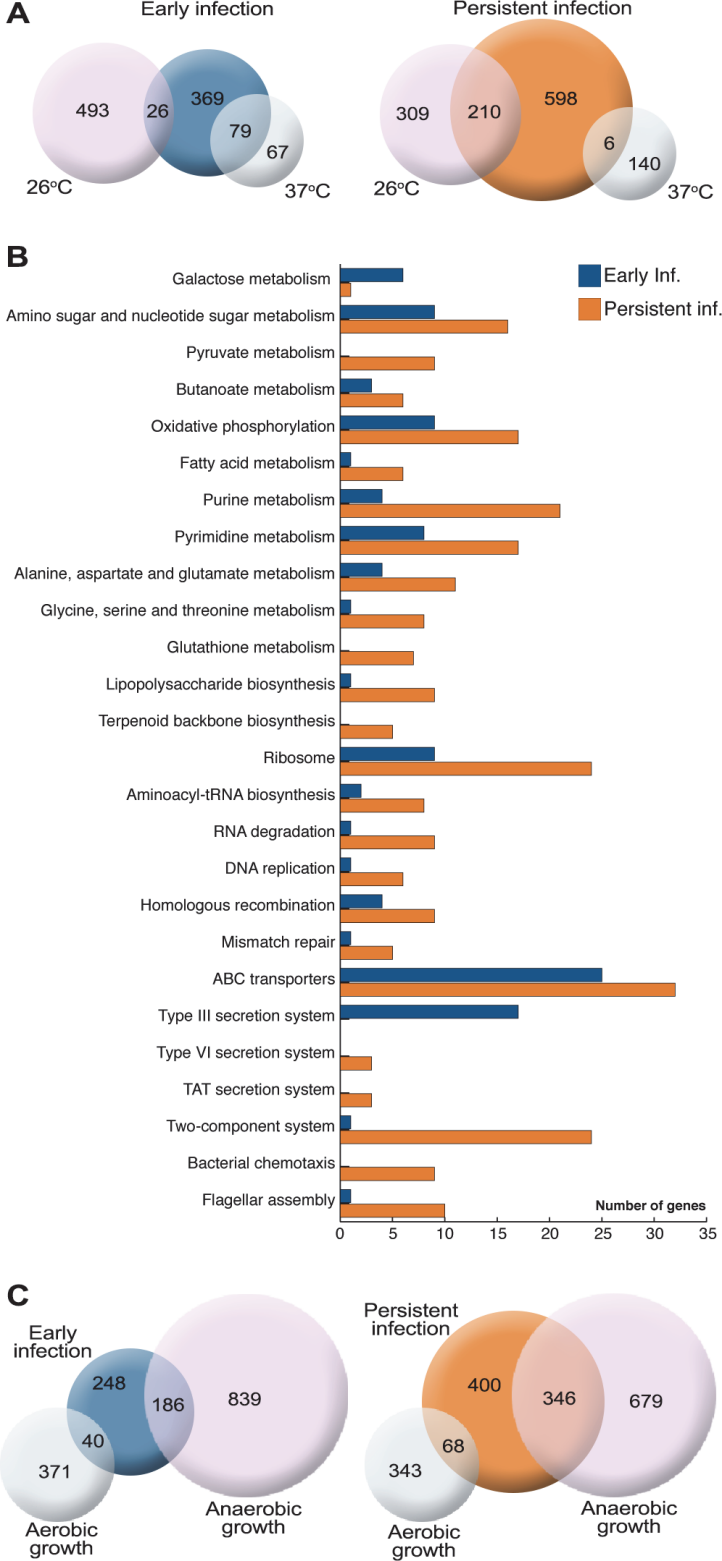
*Y. pseudotuberculosis* undergoes transcriptional reprogramming for adaption to persistence. (A) Comparison of genes up-regulated in *Y. pseudotuberculosis in vitro* at 26°C and 37°C compared to *in vivo* during early (2 dpi) and persistent (42 dpi) stages of infection. Similarities are shown with the number of genes up-regulated in both groups. (B) Functional annotation of *Y. pseudotuberculosis* genes up-regulated during early and persistent infection (KEGG pathway mapping tool). (C) Comparison of the *in vivo* gene expression profiles and the expression profiles of bacteria grown under anaerobic conditions *in vitro*. The analysis included genes up-regulated (>1.8-fold) during anaerobic or aerobic growth in both the exponential and stationary growth phase compared to genes up-regulated during early and persistent infection. Similarities are shown with the number of genes up-regulated in both groups.

A total of 1288 genes were found to be differentially expressed (log2 fold change ≥0.7) *in vivo*. RPKMO values detected by RNA-seq are shown in a histogram and heat map in Fig. 3A to highlight the differences in individual ORFs during early and persistent infection (see also S3 Table). Surprisingly, the T3SS components encoded on the virulence plasmid that were highly expressed during the early stage of infection were distinctly down-regulated during persistent infection. Another conspicuous finding was the up-regulation of flagella and chemotaxis genes. T3SS is known to be induced at 37°C, but flagella are down-regulated at this temperature; *in vitro*, flagella are expressed only at 26°C (confirmed by qPCR in the same samples used in RNA-seq; Fig. 4A–B). In analogy, the T3SS master regulator *lcrF* was up-regulated during early infection and down-regulated during persistence (S3 Table), and the flagellar regulator *flhCD* was down-regulated during early infection and up-regulated during persistence (Fig. 4B). In addition, up-regulation of the gene encoding the adhesion protein *invA*, which is co-regulated with flagella [28], and its positive regulator *rovA* [29] suggested that other genes that are only expressed at 26°C *in vitro* could be up-regulated during persistence. Accordingly, a comparison of the expression patterns of *in vivo* and *in vitro*-derived samples showed that, during the early phase of infection, bacteria have an expression pattern similar to that seen *in vitro* at 37°C, whereas the expression pattern of persistent bacteria was much more similar to that of bacteria grown *in vitro* at 26°C (Fig. 5A and S4 Table). These results clearly indicate that, though increased temperature triggers T3SS and associated virulence genes during initial infection, other environmental cues are responsible for the observed transcriptional reprogramming of *Y. pseudotuberculosis* during prolonged infection.

Flagellar genes are known to be down-regulated at 37°C; therefore, the up-regulation of flagellar genes at later time points of infection at this temperature suggests that motility may be important for certain stages of persistence. However, *Y. pseudotuberculosis* is expected to remain flagellated for some time after the temperature shift and therefore flagella have also been assumed to participate in initial infection. To investigate this possibility, *Y. pseudotuberculosis* was grown at 26°C and then shifted to 37°C, followed by sampling at different time points for the detection of flagellated bacteria using atomic force microscopy. This analysis showed that the flagella remained for at least 2 hours after shifting temperature (S2 Fig.).

### Functional clustering of differentially expressed genes uncovers environmental cues that promote reprogramming

To obtain an overview of the diversity of metabolic pathways and other functional systems utilized by persistent bacteria, functional clustering was performed using KEGG pathway mapping [30] for the 1288 genes differentially expressed *in vivo* (Fig. 5B). Down-regulation of T3SS and up-regulation of motility genes during persistence were verified. Induction of genes involved in ribosome biogenesis, amino-acyl tRNA biosynthesis, and RNA degradation suggests an active metabolic state during persistent infection. Induction of DNA replication and repair, as well as purine and pyrimidine biosynthesis, indicates proliferation of persistent bacteria and correlates well with constant bacterial shedding from the tissue to luminal sites during infection. The induction of TAT secretion components, which are involved in the transport of proteins synthesized mostly under anaerobic conditions [31], and induction of genes indicative of oxidative phosphorylation reflect an anaerobic/microaerophilic environment. Moreover, up-regulation of genes associated with two-component signal transduction systems, type VI secretion system, and chemotaxis indicates the presence of various external stimuli within the cecal environment. In addition to the functional annotations, the up-regulation of other genes indicates that the bacteria are influenced by different environmental conditions, such as acidic, oxidative, and other forms of stress (Table 2). The up-regulation of genes encoding proteins involved in envelope biogenesis, including a variety of inner and outer membrane proteins, and lipopolysaccharide biosynthesis also suggests an environmental influence. Therefore, the expression pattern of persistent bacteria suggests adaptation to an environment with limited oxygen and oxidative and acidic stress, a need for motility/chemotaxis, and modulation of the bacterial surface (Table 2).

**Table 2:** Up-regulated genes indicative of different environmental cues.

**Gene name**	**Description**
**Oxygen limitation**	
*arcA*	two-component response regulator
*arcB*	aerobic respiration control sensor protein
*fnr*	fumarate/nitrate reduction transcriptional regulator
*frdA*	fumarate reductase flavoprotein subunit
*frdB*	fumarate reductase iron-sulfur subunit
*napA*	nitrate reductase catalytic subunit
*cydA/B*	cytochrome d ubiquinol oxidase subunit I/II
*ubiE*	ubiquinone/menaquinone biosynthesis methyltransferase
*ytfB*	opacity-associated protein A
**Envelope perturbation**	
*rfaH*	transcriptional activator rfaH
*lpxC*	udp-3-o-[3-hydroxymyristoyl] n-acetylglucosamine deacetylase
*rfaF*	adp-heptose—lps heptosyltransferase
*kdsA*	2-dehydro-3-deoxyphosphooctonate aldolase
*ftsI*	peptidoglycan glycosyltransferase, cell division protein FtsI
*ytfB*	opacity-associated protein A
YPK_1854	LPP repeat-containing protein
*tatB*	twin-arginine translocation protein subunit
*tatA*	twin arginine-targeting protein translocase
*mipA*	mlta-interacting MipA family protein
*nlpD*	lipoprotein NlpD
*ompR*	osmolarity response regulator
*yfgL*	outer membrane protein assembly complex subunit
*ompF*	outer membrane pore protein F
**Bacteria-bacteria communication**	
*ypsI*	autoinducer synthesis protein
*flhD*	transcriptional activator FlhD
*hmsP*	biofilm formation regulator HmsP
*hmsF*	outer membrane n-deacetylase
*vasL*	ImpA domain-containing protein
*clpV*	type VI secretion ATPase
YPK_0802	OmpA/MotB domain-containing protein
YPK_1490	type VI secretion system lysozyme-like protein
YPK_3562	type VI secretion system lysozyme-like protein
YPK_3060	hcp1 family type VI secretion system effector
**Oxidative stress**	
*sodB*	superoxide dismutase Fe-Mn family
*katE*	catalase
*trxA*	thioredoxin
*ahpC*	alkyl hydroperoxide reductase, *reduces peroxidases*
*wrbA*	TrpR binding protein WrbA
*pgl*	6-phosphogluconolactonase
*bfr*	bacterioferritin
*ftnA*	ferritin
*dps*	DNA starvation/stationary phase protection protein
**General stress**	
*uspA*	uspA domain-containing protein
*clpX*	ATP-dependent protease ATP-binding subunit ClpX
*clpP*	ATP-dependent Clp protease proteolytic subunit
*sspB*	ClpXP protease specificity-enhancing factor
*clpA*	ATP-dependent Clp protease ATP-binding subunit
**Acid stress**	
*hdeB*	periplasmic chaperone
aspA	aspartate ammonia-lyase, cytosolic
**Motility, chemotaxis**	
*fliA*	flagellar biosynthesis sigma factor
*fliC*	flagellin
*fliE*	flagellar hook-basal body protein FliE
*motA*	flagellar motor protein MotA
*motB*	flagellar motor protein MotB
*cheW*	purine-binding chemotaxis protein
*cheB*	chemotaxis-specific methylesterase
*cheZ*	chemotaxis regulator CheZ
*tsr*	methyl-accepting chemotaxis sensory transducer
*trg*	methyl-accepting chemotaxis sensory transducer
*malE*	maltose ABC transporter periplasmic protein

### The cecal microaerophilic environment influences the expression pattern of *Y. pseudotuberculosis*

The up-regulation of several genes encoding proteins involved in oxidative phosphorylation in persistent bacteria (Fig. 5B) raised questions about energy metabolism and nutrient utilization. The cecal environment is expected to be anaerobic, and this was indicated by the sequencing data. The induction of genes involved in switching from aerobic to anaerobic respiration, such as the two-component system genes *arcA-arcB*, the fumarate nitrate reductase gene *fnr* encoding a global regulator of anaerobic growth, and other functional genes encoding proteins involved in microaerophilic/anaerobic respiration (Table 2), prompted us to investigate the influence of the anaerobic environment. We compared the expression profiles of *Y. pseudotuberculosis* grown *in vitro* under aerobic and anaerobic conditions at 26°C during the exponential and stationary phases using microarrays. Comparison of the differentially expressed genes identified in persistent bacteria by RNA-seq with the genes identified by microarray to be differentially expressed during anaerobiosis showed high similarity between persistent infection and *in vitro* anaerobic growth (Fig. 5C). Up to 42.5% of the genes up-regulated during persistence were also up-regulated during anaerobic growth (Fig. 5C and S4–S5 Tables). We found no obvious bias towards genes differentially expressed in the logarithmic or stationary phase (S3 Fig.). Thus, a substantial part of the expression profile of persistent bacteria is due to limited oxygen availability. The induction of some genes associated with anaerobic growth was also evident in samples from early infection (Fig. 5C), suggesting adaptation to the new environment at this stage.

### Crp/CsrA/RovA regulatory cascades influence the expression pattern of persistent bacteria

The observed up-regulation of *invA* and its positive regulator *rovA* suggests that the RovA regulatory cascade contributes to the expression pattern of persistent *Y. pseudotuberculosis*. RovA is a regulator of the MarR/SlyA family, which controls different physiological processes [32]. Expression of *rovA* is controlled by the global regulators CsrA and Crp [33], which were both up-regulated during persistence. PhoP, which positively regulates *rovA* in *Y. pestis* and some *Y. pseudotuberculosis* strains, is not functional in the YPIII strain, where instead the Csr system via differential regulation of Csr RNAs influences production of RovA [34]. The RovA regulon of the *Y. pseudotuberculosis* YPIII strain (grown *in vitro* at 26°C) was recently revealed by microarray analysis [35]. A comparison of the gene expression patterns of persistent *Y. pseudotuberculosis* and the reported regulon [35] revealed 27.7% of the RovA regulon with 62 activated (*lpp, fliC*, and *ftn* verified by qPCR; Fig. 3C) and 26 suppressed genes in persistent bacteria (S4 Fig. and S4 Table). A comparison of the *Y. pseudotuberculosis in vivo* transcriptome and the Crp and CsrA regulons [35] revealed that more than 20% of the respective regulons represented genes identified as being differentially expressed during persistence. Some of the identified genes were shared and others were unique for Crp or CsrA (S4 Fig. and S4 Table). Notably, the fractions of Crp, CsrA, and RovA regulons observed in persistent bacteria appear to be relatively high with regard to the low coverage of the *Y. pseudotuberculosis in vivo* transcriptome.

### Establishment of persistent infection requires *arcA, fnr, frdA*, and *wrbA*

**Figure 6:**
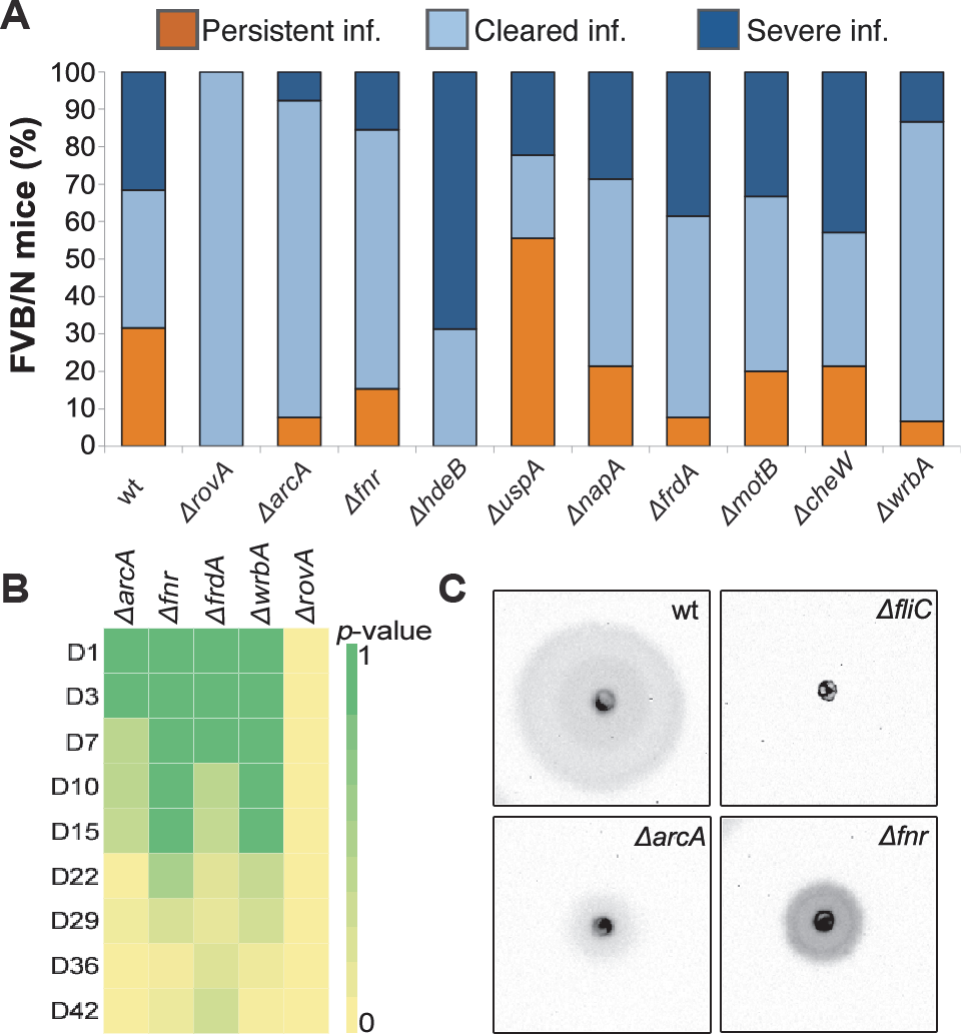
Establishment of persistent infection requires *arcA, fnr, frdA*, and *wrbA*. (A) Infection profile 42 dpi for FVB/N mice infected orally with10^7^ CFUs of wt *Y. pseudotuberculosis* (n = 20) and indicated mutant strains (each group n = 16). The infections were monitored by IVIS at certain intervals up to 42 dpi. (B) Heatmap showing differences in clearance (by *p*-value) between wt and indicated mutant strains at different time points during the 42 day infection period. Heatmap color scale, from green to yellow, was adjusted according to *p*-values from 1 to 0. *p*-values were calculated with 2×2 contingency table by Fisher’s Exact Test, see also S6 Table. (C) Motility profile of wt *Y. pseudotuberculosis* and indicated mutant strains under anaerobic conditions at 26°C. Images were captured by the ChemiDoc XRS System (Bio-Rad), showing the bioluminescent signal produced by *Y. pseudotuberculosis* YPIII/pIBX.

To determine the importance of potential persistence genes identified in the RNA-seq analysis, we constructed a set of single gene deletion mutants to test in the mouse infection model of persistent infection. We selected genes implicated in different environmental responses (i.e., *rovA, arcA, fnr, hdeB, uspA, napA, frdA, motB, cheW*, and *wrbA*). Infection was achieved and monitored with IVIS for up to 42 dpi. Among the mice infected with the wt strain 31.6% had persistent infection, 31.6% cleared the infection, and 36.8% succumbed to severe disease (Fig. 6A), and this distribution is in accordance with that reported earlier [6]. The *ΔrovA* strain was completely attenuated and did not establish infection, indicating that it is indispensable for initiation of infection. Three of the mutants lacking genes involved in anaerobic respiration (*arcA* and *fnr*) or oxidative stress (*wrbA*) had a reduced capacity to establish persistence (*Δfnr*, 15.4%; *ΔarcA*, 7.7%; and *ΔwrbA*, 6.7%). These strains also gave less and later onset of severe disease than the wt strain *(Δfnr* 15.4%; *ΔarcA*, 7.7%; and *ΔwrbA*, 14.2%; Fig. 6A and S5 Fig.). A similar but less dramatic phenotype was observed for the *ΔfrdA* mutant, which had a reduced capacity to establish persistence (7.7%) but still caused severe disease (38.5%), with even earlier onset than the wt strain (Fig. 6A and S6 Fig.). *ΔarcA, Δfnr, ΔfrdA*, and *ΔwrbA*, established infections as wt, with almost similar levels of clearance during the first 7–14 days. However, after this, there were significant differences between these mutants and the wild-type strain with higher degrees of clearance, suggesting that these gene products are important during the persistent state (Fig. 6B). Interestingly, *ΔhdeB* was cleared to almost the same extent as the wt strain but was unable to establish persistent infection; this strain also appeared to be more aggressive, with infection resulting in more frequent and faster onset severe disease (68.8% vs. 31.6% for the wt strain; Fig. 6A and S5 Fig.). The *ΔuspA* mutant had a higher frequency of persistence than the wt strain (55.6% vs. 31.6%). The other mutants, *ΔnapA, ΔmotB*, and *ΔcheW*, had infection profiles similar to the wt strain (Fig. 6A).

### Microaerophilic and acidic environments influence T3SS and motility

The decreased virulence and persistence of the mutants related to anaerobic growth (*ΔarcA, Δfnr*, and *ΔfrdA*), combined with the down-regulation of T3SS and up-regulation of motility genes during persistent infection, prompted us to analyze T3SS secretion and motility *in vitro* in the presence and absence of oxygen. Secretion of T3SS effectors was reduced under anaerobic conditions for wt and all tested mutants (S6A Fig.). The reduction in T3SS effectors was also confirmed by qPCR (S6B Fig.). Moreover, to evaluate the contributions of the selected genes to bacterial motility, strains with mutations affecting the establishment of persistent infection were subjected to a motility test at 26°C and under T3SS-inducing conditions at 37°C in the presence and absence of oxygen. Neither the wt nor any of the mutants were motile at 37°C, independent of oxygen, but all strains except a *ΔfliC* mutant were motile at 26°C in the presence of oxygen. Interestingly, in the absence of oxygen at 26°C, the motility of *Δfnr* and *ΔarcA* mutants was significantly reduced (Fig. 6C).

Next, we investigated the influence of acid on T3SS and motility. Similar to what was observed for low oxygen, acidic conditions (pH 5.2) inhibited the induction of T3SS under inducing conditions at 37°C (S6A–S6B Fig.). Inhibition of T3SS under acidic conditions in *Y. pseudotuberculosis* was reported previously and suggested to involve pH-dependent inhibition of YscU proteolysis [36]. Furthermore, acidic conditions completely repressed the motility of the wt strain and all mutants tested in this study. Thus, both low oxygen and acidic conditions have a negative effect on T3SS induction and represent environmental cues that can contribute to the observed reprogramming of *Y. pseudotuberculosis* from a virulent to adaptive state.

## Discussion

In this study, we applied *in vitro* and *in vivo*-based RNA-seq to determine key players that enable *Y. pseudotuberculosis* to establish a persistent infection. We found that the bacterium undergoes reprogramming from a virulent phenotype, massively expressing T3SS components in the early invasive phase of infection, to an adapted phenotype capable of persisting in a microaerophilic and hostile environment. This finding has clear impact on future rationales to identify bacterial targets for new antibiotics.

As shown here, RNA-seq is a powerful method for retrieving robust information about bacterial gene expression profiles during *in vivo* infection. We show that pathogen gene induction can be detected even if the amount of infecting bacteria in the isolated tissue is very low and mixed with other bacterial species. Thorough work with varied optimization steps allowed us to discriminate *Yersinia*-specific reads at the resolution of a single base with partial coverage from *in vivo*-derived samples and full coverage from *in vitro*-derived samples. We found that very strict read mapping parameters should be used to discriminate *Y. pseudotuberculosis*-specific reads for *in vivo* data. This strategy, which was established and optimized in this study, provides a controlled solution for discriminating between species-specific transcripts in complex RNA populations. Using this methodology we obtained robust data, revealing 36% of the *Y. pseudotuberculosis in vivo* transcriptome and providing novel information about bacterial gene expression during infection.

A discrepancy in the level of coverage between *in vitro* and *in vivo*-derived bacteria was expected based on previous studies of *Vibrio cholerae* [19] and *Campylobacter jejuni* [37]. The starting materials in those studies were cecal or intestinal contents, not infected tissue, and contained 100–300 times more bacteria. In addition, the overall coverage in those studies was higher than what we obtained with our tissue biopsy approach. However, the overall coverage for the metatranscriptome was >92%, and the ratio of *Y. pseudotuberculosis* total RNA was ∼0.2%. Full coverage of a bacterial transcriptome in such a complex population with low abundance is estimated to require a sequencing depth of at least 1.5–2 billions reads [38]. This estimate was calculated based on only the host and *Y. pseudotuberculosis* RNA being present in the sample, whereas additional RNAs from many bacterial species were present in our samples. Therefore, the depth of sequencing for such biopsy samples may need to be several times higher than 1.5–2 billion reads.

The massive expression of T3SS virulence genes during the early phase of infection is likely necessary to break the epithelial barrier and defend against innate immune cells. This assumption is supported by previous data showing that *yopH* or *yopE* mutants are defective in establishing the initial infection and less able to cause persistent infection [6]. Later, the bacteria become persistent with a novel expression profile, suggesting substantial transcriptional reprogramming. At this stage, the T3SS components are down-regulated. Thus, the bacteria prefer to use other genetic resources to adapt to the environment instead of producing massive amounts of invasive T3SS components.

Taking the host temperature into consideration, the repertoire of up-regulated genes in persistent bacteria was remarkably different from the repertoire of bacteria grown *in vitro* at 37°C. One striking observation was the up-regulation of flagella at 37°C, as achieving the induction of flagella is impossible at this temperature *in vitro*. Therefore, the situation in the animal greatly differs from laboratory settings. The surprising finding that the expression pattern seen during persistence is similar to the pattern seen at 26°C in vitro, indicates that the pathogens during infection encounters multiple environmental cues, other than the temperature that substantially influences its gene expression. Consequently, the reprogramming likely enables bacteria to persist in the harsh environment in cecum lymphoid follicles, where the tissue-associated bacteria are surrounded by PMNs. The functional annotation analysis revealed genes indicative of a microaerophilic environment with acidic and oxidative stress factors. We found similarities between the repertoire of up-regulated genes in bacteria grown *in vitro* under anaerobic conditions and the repertoire of persistent bacteria in the cecums of infected mice. Given the presence of PMNs in the cecum during persistence, it is not surprising that adaption requires protection against acidic and oxidative stress, or that it involves modulation of the bacterial surface for protection. Reprogramming probably occurs after initial colonization that requires T3SS, and later on certain environmental cues force the pathogen to reprogram its transcriptome where the induced gene products aid in maintaining long-term survival in this particular niche. At this late stage of infection the pathogen resists elimination by PMNs with very low expression of its T3SS-associated virulence arsenal, which is puzzling. However, the pathogen may be less recognized by innate immune cells due to its adapted phenotype with altered surface, and maybe also secretion of protective factors. In addition, we cannot exclude that presence of other microbial inhabitant(s) contributes. The observed up-regulation of type VI secretion genes previously reported to participate in bacteria-bacteria communication [39], as well as up-regulation of genes involved in biofilm formation and quorum sensing, may reflect interactions with other bacteria.

Up-regulation of genes involved in DNA replication and repair, RNA degradation, tRNA biosynthesis, and ribosome biogenesis suggests a metabolically active state for persistent bacteria, but it is not a direct indication of whether persistent bacteria are in a dormant or replicative form. We hypothesize that bacteria have a restricted replicative form in order to maintain bacterial load with consistent bacterial shedding into the feces. Motility may also be required for efficient shedding and spread of the bacteria within a restricted host environment, as shown by reduce bacterial shedding into the cecal lumen in a flagellar mutant of avian pathogenic *Escherichia coli* in a chick persistence model [40].

We show that regulators of anaerobic growth, as well as genes involved in oxidative/acidic stress, are important for the establishment of persistent infection with *Y. pseudotuberculosis*. Both *arcA* and *fnr* mutants demonstrated a markedly reduced ability to establish severe or persistent infection in mice, demonstrating the importance of reprogramming to anaerobic respiration. The function and importance of *arcA* and *fnr* have not been studied extensively in *Yersinia*. Here, we show that these gene products control motility, which has also been shown for *arcA* in *Salmonella enterica sv. Typhimurium* and *E. coli* [41,42]. In an earlier study, *arcA* was reported to be dispensable for acute *Y. pseudotuberculosis* infection upon intragastric inoculation in BALB/c mice [43], whereas another more recent study showed that a *Y. pseudotuberculosis arcA* mutant had attenuated virulence [35]. Whether the former study is contradictory to the latter and our results or just reflects different requirements of *arcA* depending on infection dose or intestinal delivery of bacteria remains to be elucidated. However, in agreement with our data, *arcA* has been implicated in virulence for a variety of bacteria [44,45]–[46–48]. Importantly, our data also show that a reduced ability to cause persistent infection is not directly coupled to decreased virulence in general, as increased virulence and an absence or reduced level of persistence was observed for the *hdeB* and *frdA* mutants. However, the mechanisms responsible for the virulence phenotypes of the *hdeB* and *uspA* mutants, with the former resulting in increased severe disease and the latter increased persistence, is not obvious and requires further investigation.

The regulatory pathways responsible for the switch, where T3SS is down-regulated is a central question in this context. We show that T3SS cannot be induced under low oxygen or acidic conditions. Therefore, regulatory circuits mediating T3SS repression are active under these conditions. Notably, FNR indirectly represses the expression of T3SS effectors under anaerobic conditions in *Shigella flexneri* [49]. However, we showed here that FNR *per se* has no effect on T3SS secretion in *Y. pseudotuberculosis*, as T3SS was repressed at the same level in the *fnr* mutant and wt strains under anaerobic conditions. The same was observed with the *arcA* mutant.

We found that many of the genes (20%) that were differentially expressed during persistence overlapped with the Crp/CsrA/RovA regulons, indicating that this regulatory circuit contributes to persistence in the host. The global regulatory systems of Crp and CsrA involves energy metabolism, but also control of certain virulence functions [33]. The interplay between these regulators and RovA is delicate and incorporates a series of complex regulatory loops that can be influenced by other regulators including UvrY and Hfq (all up-regulated during persistence). CsrA can control RovA via RovM, and independently of RovA, positively regulate flagella/motility genes as well as *arcA* [33,35]. Induction of flagella/motility genes is also influenced by Crp, which can control CsrA and promote induction of *rovA*. We hypothesize that genes induced by the Crp/CsrA/RovA regulatory cascades, which are mainly down-regulated during the early phase when T3SS is on, participate in reprogramming of *Yersinia* physiology by promoting expression of genes necessary for persisting in the cecal environment. RovA has been shown to be critical for virulence in *Y. enterocolitica* and *Y. pestis* [29,50]. By analogy, we found that RovA was required for virulence upon low dose infection. Interestingly, in contrast to the *arcA, fnr, frdA*, and *wrbA* mutants that initially infected cecum, but thereafter were efficiently cleared, the *rovA* mutant did not establish infection at all upon oral infection of mice. Hence, not only is RovA required for the positive regulation of many genes expressed at 26°C *in vitro* and during persistence, but is important also for initial infection where the gene expression pattern actually resembles that seen at 37°C *in vitro* with expression of T3SS. This suggests that RovA contributes to adaption to the host environment also at early stages of infection. Regulators of the Mar/SlyA family have been implicated in the regulation of genes involved in coping with diverse environmental stresses [32]. As such, RovA could contribute to the initial infection by regulating genes important for resistance to low pH in the stomach and to reactive oxygen metabolites produced by innate immune cells in cecum.

**Figure 7:**
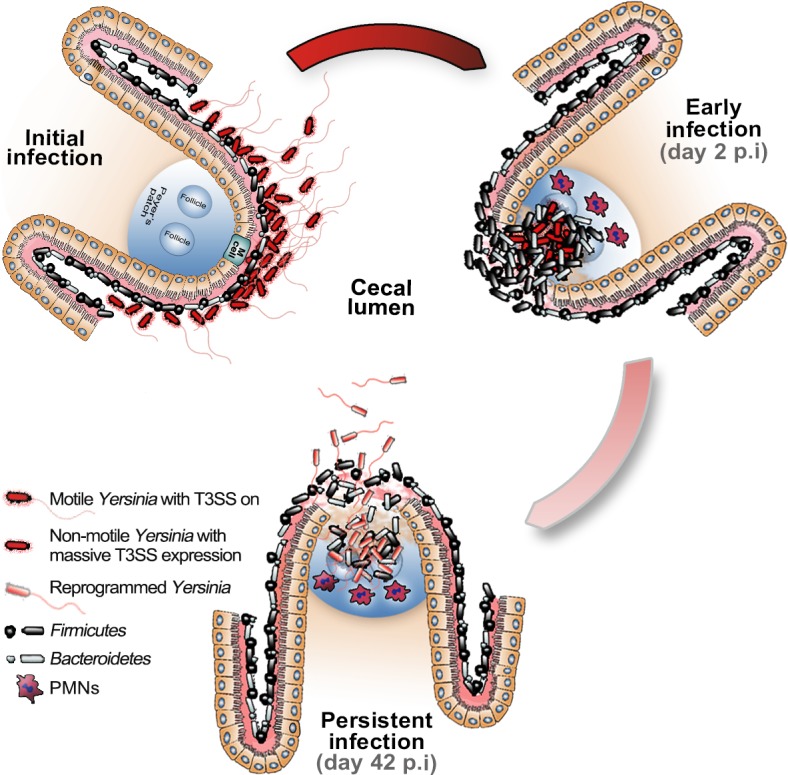
Hypothetical model of *Y. pseudotuberculosis* reprogramming for persistent infection in cecum. Upon initial infection, *Y. pseudotuberculosis* is still flagellated and expresses T3SS virulence genes. At the early stage of infection (2 dpi) the T3SS is important for colonization of tissue, including breaking the epithelial barrier and resisting the attack from arriving PMNs. At the persistent stage of infection (42 dpi), *Y. pseudotuberculosis* had reprogrammed its transcriptome by reducing the expression of T3SS components and increasing the expression of genes important for survival in the cecal lymphoid compartment. At this stage the bacteria are flagellated and can spread to other hosts by shedding into the feces, possibly through motility.

The persistence route may reflect the life cycle of this enteropathogen. In such a cycle we hypothesize that, during the initiation of infection, *Y. pseudotuberculosis* still has flagella and expresses T3SS virulence genes for breaking the epithelial barrier (Fig. 7). Flagella expression is supported by *in vitro* data, which showed flagellated bacteria up to 2 hours after shifting the temperature to 37°C. T3SS components are expected to be instrumental for resisting the attack from arriving PMNs during the early phase. In later stages, the bacterium adapts to the environment by reducing the expression of T3SS components and increasing the expression of genes important for survival in the cecum lymphoid compartment, from where it can spread to other hosts by fecal shedding, possibly through motility. In this context, *Y. pseudotuberculosis* has been found in the colon of wild mice with hyperemic cecal membranes [51], suggesting that this compartment is a potential reservoir for this pathogen.

## Materials and Methods

### Strains and growth conditions

YPIII/pIBX, a kanamycin-resistant bioluminescent *Y. pseudotuberculosis* strain (S6 Table), was used in this study. The YPIII strain represents a well established model for *Y. pseudotuberculosis* that has been used for decades to elucidate various aspects of *Y. pseudotuberculosis* pathogenesis, including identification of Yops [2,4–6,33,36,52]. For *in vitro* total RNA preparation, the strain was cultured at 26°C or 37°C in brain heart infusion (BHI) broth or LB at acidic (pH 5.2) or normal pH supplemented with 50 μg/ml kanamycin, 5 mM EGTA, and 20 mM MgCl_2_ for T3SS induction at 37°C. For microarray analysis, wt *Y. pseudotuberculosis* YPIII was grown at 25°C in LB medium supplemented with 10 g/l glucose and 0.2 M HEPES buffer under aeration or under anaerobic growth conditions (in a nitrogen atmosphere). Additional glucose was added to maximize energy production and the growth rate under anaerobic growth conditions. The absence of oxygen in the culture medium was tested by a gas chromatograph (GC-WLD, Carlo Erba Vega Series 6000) coupled with a detector and integrator (Spectra-Physics, SP4270) using a Poropak QS (100–120 Mesh) column and helium (Westalen 4.6) at 300 kPa. See Supplemental Experimental Procedure for the mutant strains used in this study.

### Mutant construction

In order to generate an in-frame deletion mutant of the gene of interest, approximately 200 nucleotides from the 5’ and 3’ flanking regions of the gene were amplified by PCR and ligated together into *Sal*I and *Bgl*II (New England Biolabs, Inc) linearized pDM4 [53] using the In-Fusion HD Cloning Kit (Clontech Laboratories, Inc) according to the manufacturer’s instructions. The plasmid was transformed into *E. coli* DH5αλpir and selected on a Cml (25 μg/ml)-containing agar plate. Positive colonies were confirmed by colony PCR. Plasmids purified from positive clones were sequenced to confirm insertion. Confirmed plasmid constructs were transformed into *E. coli* conjugation strain S17-1λpir for conjugal mating with *Y. pseudotuberculosis* YPIII-Xen04. Positive allelic exchange was selected as described previously [53]. Finally, in-frame deletion mutants of *arcA, fnr, hdeB, uspA, cheW, frdA*, and *motB* were confirmed by sequencing (S7 Table).

### Ethics statement

Mice were housed in accordance with the Swedish National Board for Laboratory Animals guidelines. All animal procedures were approved by the Animal Ethics Committee of Umeå University (Dnr A108-10). Mice were allowed to acclimate to the new environment for one week before the experiments.

### Mouse infection and bioluminescent imaging

Eight-week-old female FVB/N (Taconic Farms, Inc) mice were deprived of food and water for 16 hours prior to oral infection with ∼10^7^ CFUs of wt or mutant *Y. pseudotuberculosis* YPIII-Xen04 strains, which were supplied in their drinking water for 6 hours. Bacteria were subcultured on LB agar plates supplemented with kanamycin (50 μg/ml). For infection, the bacteria were grown overnight in LB at 26°C and concentrations estimated by absorbance at OD_600nm_. Cultures were re-suspended to 10^7^ CFUs/ml in sterilized tap water supplemented with 150 mM NaCl. The infection dose was determined by viable count and drinking volume. Mice were inspected frequently for signs of infection and to ensure that infected mice showing prominent clinical signs were euthanized promptly to prevent suffering. The infections were monitored using IVIS Spectrum (Caliper LifeSciences, Inc.) every third day after infection to 15 dpi, and then every week up to 42 dpi. Prior to imaging, the mice were anesthetized using the XGI-8 gas anesthesia system (Caliper LifeSciences, Inc), which allowed control over the duration of anesthesia. Oxygen mixed with 2.5% IsoFloVet (Orion Pharma, Abbott Laboratories Ltd, Great Britain) was used for the initial anesthesia, and 0.5% isoflurane in oxygen was used during imaging. To analyze bacterial localization within organs, mice were euthanized, the intestine, mesenteric lymph nodes, liver, and spleen removed, and the organs imaged by bioluminescent imaging (BLI). Acquisition and analysis were performed using Living Image software, version 3.1 (Caliper LifeSciences, Inc.).

### Immunohistochemistry

Cecal tissue was fresh frozen in isopentane pre-chilled with liquid nitrogen and kept at −80°C. For detection of *Yersinia* in the tissue, 10-μm cryosections were fixed and stained with α-*Yersinia* serum and for immunohistochemistry sections were stained with rat-α-mouse Gr-1 (clone RB6-8C5, BD Biosciences Pharmingen) as described previously [6]. Cecal sections were also stained with hematoxylin-eosin using standard methods. Analysis was performed using a NIKON Eclipse 90i microscope and images captured with a Hamamatsu Orcha C4742-95 camera or NIKON DSFi1 camera and NIS-Elements AR 3.2 software (Nikon Instruments).

### Bacterial total RNA isolation from mouse cecal tissue and *in vitro* bacterial cultures

Total RNA was isolated as described previously [54,55] with small modifications. Dissected cecums were emptied by flushing the luminal contents several times with 1× PBS using a sterile syringe. Parts of the cecal tissue associated with *Y. pseudotuberculosis-Xen4* (bioluminescent) was cut out using a sterile 3 mm hole punch and immediately transferred to RNAlater (Ambion) for overnight incubation at 4°C after IVIS confirmation of bacterial in the isolated tissue. The RNAlater solution was removed the next day and the tissue samples stored at −80°C. The tissue was homogenized using Dispomix Drive (Medic Tools AG, Switzerland) and all steps performed at 4°C. The samples were transferred to previously cooled Dispomix homogenization tubes (Medic Tools AG, Switzerland) containing 1 ml of Solution D and homogenized twice using homogenization program 9. Tissue lysates were spun down with a quick spin. Each sample was aliquoted (0.5 ml) into separate 2 ml bead beater tubes containing small (0.1 mm) and big (1 mm) glass beads and treated with Mini-Beadbeater (Biospec Products Inc, USA) at a fixed speed for 1 min. Samples were cooled on ice for 1 min and the following added sequentially: 50 μl 2M sodium acetate (pH 4.0), 500 μl water-saturated phenol (Invitrogen, CA, USA), and 100 μl chloroform:isoamyl alcohol (49:1). The samples were inverted vigorously by hand. Suspensions were centrifuged for 20 min at 10,000*g* after cooling on ice for 15 min. The upper aqueous phase was transferred to RNase-free 1.5 ml tubes and 1 ml isopropanol added to precipitate the RNA. Samples were incubated at −20°C for 2 hours and centrifuged for 20 min at 10,000*g*. The RNA pellet was dissolved in 0.3 ml Solution D, 0.3 ml isopropanol added, and the resulting aliquots for each sample combined in one tube. The final suspensions were incubated for 30 min at −20°C and centrifuged for 20 min at 10,000*g*. The RNA pellets were suspended in 50 μl RNase-free water. DNA contamination was removed using the Qiagen DNase Kit according to the manufacturer’s instructions. The same procedure was applied to bacterial cultures grown *in vitro* except for the homogenization step with the Dispomix Drive homogenizer. The quality and concentration of total RNA isolated from the cecum and *in vitro* cultured bacteria was assessed by microcapillary electrophoresis using an Agilent 2100 Bioanalyzer (Agilent Technologies, Palo Alto, CA). All preparations used in this study had an RIN value >7.0.

### Depletion of rRNA and poly(A)-tagged RNA from total RNA preparations

The MICROB*Enrich* Kit (Ambion) was used to enrich bacterial mRNAs in total RNA samples from cecums by removing 18S and 26S rRNAs and polyadenylated mRNAs according to the manufacturer’s instructions. To deplete the bacterial rRNA and tRNA in total RNAs from *in vivo* and *in vitro* samples, we used a MICROB*Express* Kit (Ambion) according to the manufacturer’s instructions.

### Illumina TruSeq RNA library preparation

RNA libraries for sequencing were prepared using TruSeq RNA kits (Illumina, CA, USA) according to the manufacturer’s instructions, but with the following changes. The RNA samples were EtOH precipitated and subsequent protocols (starting from cDNA synthesis in the Illumina provided protocol) were automated using an MBS 1200 pipetting station (Nordiag AB, Sweden). All purification steps and gel-cuts were replaced by the magnetic bead clean-up methods described previously [56].

### RNA-seq

Quality and base trimming (5 nt from 5’ end and 5 nt from 3’ end of each read) were performed on 100-nt-long paired-end Illumina 2000 Hiseq reads from *in vivo* and *in vitro* sample libraries. Trimmed reads for each *in vivo* library were mapped to the NCBI 16SMicrobial database to determine the bacterial species in the cecal tissue biopsies with 100% identity. Identified bacterial species with available reference genomes (42 annotated bacterial genomes), *Y. pseudotuberculosis* YPIII, and the pYV plasmid from NCBI were used as reference genomes for mapping. A variety of mapping tests were performed by loosening or strengthening the alignment settings to optimize the filtration of non-*Y. pseudotuberculosis* YPIII-specific reads with SNP calling using Probabilistic Variant Detection in CLC Genomics Workbench after each mapping attempt. The rRNA and tRNA annotations were removed from the reference genomes prior to RNA-seq in order to avoid bias from the rRNA depletion procedures. The mRNA expression level and RPKMO value was calculated for each gene in *in vivo* and *in vitro* samples using annotated NC_010465 and NC_006153 as reference genomes in CLC Genomic Workbench for RNA-seq. Analysis of differentially expressed genes were performed on normalized RPKMO values by CLC Genomic Workbench for RNA-seq. The zero read values for the ORFs in *in vivo* samples were normalized by adding 0.01 pseudocounts in order to avoid dividing by zero [57–59]. As many of the reads detected for individual ORFs were only present in samples from either early or persistent infection, proper *p*-values could not be calculated for the *in vivo* data set. For *in vitro* data, the ORFs with less than 10 reads in all four replicates and p >0.05 were removed from the analysis to filter out false-positive values. The raw RNA-seq data have been deposited in NCBI’s Gene Expression Omnibus and are accessible through GEO Series accession number GSE55292.

### cDNA preparation and qPCR

Total pure bacterial RNA isolated from bacterial cultures grown in BHI/LB medium at pH 7.2 at 26°C/37°C (in some experiments at acidic pH or under anaerobic conditions at 37°C) and heterogenous RNA isolated from infected cecums were used as templates for cDNA synthesis with the RevertAid H Minus First Strand cDNA Synthesis Kit (Fermentas). The qPCR reactions were performed in triplicate for each condition using the Quantimix Easy Syg Kit (Biotools) or KAPA SYBR FAST qPCR Kit (Kapabiosystems) and Bio-Rad i5 Light Cycler. After optimization experiments (S7 Fig.) *gyrB* was selected as internal control to calculate the relative expression of tested genes.

### Microarray and data analysis

The design and analysis of the microarray (Agilent, 8×15K format) for the transcriptome analysis of *Y. pseudotuberculosis* YPIII were described previously [33]. YPIII was grown aerobically and anaerobically in four independent cultures at 25°C to the exponential and stationary phase. Bacteria were pelleted, mixed with 0.2 volumes of stop solution (5% water-saturated phenol), and snap frozen in liquid nitrogen. After thawing on ice, total RNA was prepared using the SV Total RNA Purification Kit (Promega) and remaining genomic DNA removed by rDNAse (Macherey-Nagel) digestion as described by the manufacturer. RNA concentration and quality were determined by measuring A_260_ and A_280_ with an Agilent 2100 Bioanalyzer using the Nano 6000 kit, and the absence of DNA was excluded by PCR of intergenic regions. Total RNA from the independent cultures was labeled using the ULS™ Fluorescent Labeling Kit for Agilent Arrays (Kreatech) as follows: 1 μg total RNA was used for RNA-labeling with Cy5 (for wt RNA) and Cy3 (for mutant RNA). Non-incorporated Cy5/Cy3 was removed using KREA*pure* purification columns from the ULS™ Fluorescent Labeling Kit as suggested by the manufacturer, and the degree of labeling was analyzed with a Nanodrop (Peqlab). Subsequently, 300 ng Cy5-labelled RNA and 300 ng Cy3-labelled RNA were mixed, fragmented, and hybridized for 17 h at 65°C to custom-made Agilent microarray slides using the Agilent Gene Expression Hybridization Kit as described by the manufacturer. Four replicates were utilized in each experiment. After washing and drying the microarray slide, the data were scanned using an Axon GenePix Personal 4100A scanner and array images captured using the software package GenePix Pro 6.015. The microarray data was processed using the software package R (www.r-project.org) in combination with the “Bioconductor” software framework [60] as described previously [33]. The overall fold-changes of a gene represented by at least three probes are given as median values for all probes. The set of resulting differentially expressed genes (fold-change ≥ 2) was analyzed by the topGO package for Gene Ontology (GO) term enrichment [61]. MIAME compliant array data were deposited in the Gene Expression Omnibus (GEO) database and are available via the following accession numbers: GSE56475 and GSE56475.

### Motility assay

Bacteria from overnight cultures were inoculated into LB and grown to exponential phase. A 5 μl aliquot of each culture was spotted on LB (pH 7.4 or pH 5) with 0.25% agar. Plates were incubated at 26°C or 37°C under aerobic or anaerobic conditions for 48 hours. The bioluminescent signal from bacteria on the plates was monitored using a ChemiDoc XRS System (Bio-Rad).

### Analysis of protein secretion by *Y. pseudotuberculosis*

Overnight bacterial cultures were diluted 25-times in LB media and allowed to grow for 2 hours at 26°C. The medium was changed to T3SS-inducing conditions (5 mM EGTA, 20 mM MgCl_2_, pH 7.4 or 5) and incubated for 4 hours at 37°C under aerobic and anaerobic conditions. The culture supernatant was collected by centrifugation at 4000 rpm for 10 min and filtered using 0.45-μm filters. The secreted proteins were concentrated by TCA precipitation. Samples were loaded onto 12% SDS-PAGE according to the cultures’ OD_600_ values after 4 hours incubation.

### Visualization of flagella by atomic force microscopy

Bacterial cultures grown overnight were diluted 25-times in LB media and allowed to grow for 2 hours at 26°C to OD_600_ = 0.2. The growth conditions were then changed to T3SS-inducing conditions at 37°C. One milliliter was taken from the bacterial cultures every hour for analysis with atomic force microscopy. Each sample was centrifuged for 4 min at 1500 rpm, washed once with 2 mM MgCl_2_, and re-suspended in 50–200 μl of the same solution. Ten microliters of each sample was placed on freshly cleaved ruby red mica (Goodfellow Cambridge Ltd, Cambridge), incubated 5 min at room temperature, and blotted dry before being placed into a desiccator for a minimum of 2 hours. Images were collected by a Nanoscope V AFM (Bruker software) using ScanAsyst in air with ScanAsyst cantilevers at a scan rate of approximately 0.9–1 Hz. The final images were flattened and/or plane-fitted in both axes using Bruker software and presented in amplitude (error) mode.

### Statistical analysis

Windows Microsoft Excel 2011 and CLC Genomic Workbench were utilized for statistical tests and linear regression analysis of RNA-seq and infection data. Multiple RNA-seq were compared by the paired t-test on Gaussian data. Bonferroni correction was employed for multiple comparison analysis. Similarities between replicates were determined by Spearman and Pearson’s *R* value. One-tailed test were conducted to calculate *p*-values for difference in mutant infections clearance with 2×2 contingency table by Fisher’s exact test.

### Accession numbers

Kyoto Encyclopedia of Genes an Genomes (KEGG) accession numbers for the genes mentioned in this study are as follow; *arcA* (ypy:YPK_3606), *fnr* (ypy:YPK_1944), *rovA* (ypy:YPK_2381), *frdA* (ypy:YPK_3813), *hdeB* (ypy:YPK_1140), *uspA* (ypy:YPK_0120), *napA* (ypy:YPK_1387), *wrbA* (ypy:YPK_2363), *motB* (ypy:YPK_0802), *cheW* (ypy:YPK_1750), *csrA* (ypy:YPK_3372), *crp* (ypy:YPK_0248), *dnaG* (ypy:YPK_0635), *gyrB* (ypy:YPK_0004), *mdh* (ypy:YPK_3761), *rpoC* (ypy:YPK_0341), *fliC* (ypy:YPK_2381), *lpp* (ypy:YPK_1854), *ompF* (ypy:YPK_2649), *ompA* (ypy:YPK_2630), *ftn* (ypy:YPK_2438), *aspA* (ypy:YPK_3825), *yhbH* (ypy:YPK_3353), *pal* (ypy:YPK_2955), *flhC* (ypy:YPK_1746), *flhD* (ypy:YPK_1745), *fliA* (ypy:YPK_2380), *fliE* (ypy:YPK_2390), *fliK* (ypy:YPK_2396), *flgL* (ypy:YPK_2415), *flgH* (ypy:YPK_2419), *flgG* (ypy:YPK_2420), *flgB* (ypy:YPK_2425), *flgA* (ypy:YPK_2426), *invA* (ypy:YPK_2429), *uvrY* (ypy:YPK_2326), *hfq* (ypy:YPK_3799), *yopB* (pYV0055), *yopD* (pYV0054), *yopH* (pYV0094), *yopE* (pYV0025), *yopK* (pYV0040), *yopM* (pYV0047), *lcrF* (pYV0076).

## Supporting Information

S1 Fig Transcriptome coverage of other bacteria in cecal samples. The transcriptome coverage of each indicated species was obtained using reference genomes, if available, of species identified by 16SMicrobial database mapping of persistent infection samples. Columns indicate the percent coverage of the species in both early and persistent infection samples. RNA-seq analyses were performed on rRNA-depleted cDNA libraries with CLC Genomic Workbench.
(TIF)

S2 Fig Stability of flagella under T3SS-inducing conditions at 37°C. Visualization of *Y. pseudotuberculosis* flagella by atomic force microscopy. Wt *Y. pseudotuberculosis* were grown at exponential phase (26°C) and subjected to T3SS-inducing conditions (depleted Ca^2+^ at 37°C) and thereafter analysed every hour for 5 hours.
(TIF)

S3 Fig Similarities between up-regulated genes during anaerobic growth and persistent infection are independent of bacterial growth phase. Venn diagram illustrating the number of overlapping genes up-regulated in vivo during early versus persistent infection with up-regulated genes during logarithmic and stationary growth *in vitro*.
(TIF)

S4 Fig CsrA, Crp, and RovA influence gene expression during persistent infection. Venn diagram showing number of genes whose expression correlate with the CsrA, Crp, and RovA regulons [35] and during persistent infection (this study). Circle with centered dot indicates total number of the genes in RovA, Crp, and CsrA regulons. Circle indicates number of genes in RovA, Crp, and CsrA regulons whose expression pattern overlap with persistent infection. Diamond indicates total number of differentially expressed genes during persistent infection. Square indicates number of differentially expressed genes during persistent infection whose expression pattern overlaps with RovA, Crp, and CsrA regulons.
(TIF)

S5 Fig Survival chart of FVBn mice infected with *Y. pseudotuberculosis YPIII* wt and mutant strains. Low dose oral infection of FVBn mice with wt *Y. pseudotuberculosis* YPIII and the indicated mutant strain up to 42 dpi. Survival is given as a percentage of the total number of infected mice.
(TIF)

S6 Fig Analysis of T3SS effectors under different conditions. (A) Proteins secreted by *Y. pseudotuberculosis* wt and indicated mutant strains under T3SS-inducing conditions (depleted Ca^2+^ at 37°C) were analyzed in the presence and absence of oxygen and at normal and low pH. The secreted proteins were concentrated by TCA precipitation and loaded onto the gel according to each culture’s OD_600_ value. (B) The expression T3SS components in the presence and absence of oxygen in LB with normal (control) and low pH under T3SS-inducing conditions (depleted Ca+2 at 37°C) determined by qPCR.
(TIF)

S7 Fig The gene expression pattern of *gyrB* is stable under different *in vitro* growth conditions. The expression levels of *dnaG, gyrB, mdh*, and *rpoC* under different growth conditions and temperatures were analyzed by qPCR. Bacterial cultures were grown in BHI/LB medium at OD_600_:0.1 and 0.3 at 26°C and OD_600_:0.3 at 37°C under T3SS-inducing conditions. The same amount of cDNA template was used in qPCR analysis.
(TIF)

S1 Table Identified bacterial species in *in vivo* samples. Bacterial species identified by mapping cDNA sequencing reads from two uninfected mice, two infected (2 days), two persistently infected (42 days) cecal tissues to corresponding 16S rRNA sequence from NCBI 16SMicrobial database.
(XLSX)

S2 Table *In vitro* (26°C vs 37°C) differentially expressed genes of *Y. pseudotuberculosis* YPIII. The analyses were performed with CLC-Bio Genomics Workbench by using Transcriptomics Analysis Module. The data were obtained from two biological replicates from bacterial cultures grown at 26°C and 37°C virulence inducing conditions. The expression values are given as RPKM and differentially expressed genes were filtered with Log2 fold change ≥0,7. All data generated in this study were deposited in the Gene Expression Omnibus (GEO) database and are available under accession number GSE56477.
(PDF)

S3 Table *In vivo* (persistent infection vs early infection) differentially expressed genes of *Y. pseudotuberculosis* YPIII. The analyses were performed with CLC-Bio Genomics Workbench by using Transcriptomics Analysis Module. The data were obtained from two biological replicates from cecal tissue of two FVBn mice at early phase of infection and from cecal tissue of two FVBn mice at persistent infection. The expression values are given as RPKM and differentially expressed genes were filtered with Log2 fold change ≥0,7. All data generated in this study were deposited in the Gene Expression Omnibus (GEO) database and are available under accession number GSE56477.
(PDF)

S4 Table Comparison of *Y. pseudotuberculosis* YPIII gene expression at different conditions and in *rovA, csrA*, and *crp* mutant strains. Up- and downregulated genes in wt *Y. pseudotuberculosis* YPIII during persistent infection in mice, at 26°C *in vitro*, under anaerobic condition, and in *ΔrovA, ΔcsrA*, and *Δcrp* mutant strains. The list is sorted from highest to lowest value of upregulation during persistent infection.
*Indicates data obtained from Bucker et al., 2014.
(PDF)

S5 Table Anaerobic vs. Aerobic Microarray of *Y. pseudotuberculosis* YPIII. Differentially expressed *Y. pseudotuberculosis* YPIII (Log2 FC≥0.8 and p-value<0.05) genes under anaerobic and aerobic conditions at 26°C in exponential and stationary phase analyzed by microarray.
(XLS)

S6 Table Significantly higher clearance of *ΔarcA, Δfnr, ΔfrdA, ΔwrbA*, compared to the wt strain during later stages of infection. Differences (*p*-values; Fisher’s Exact Test) in clearance of infection between wt and indicated mutants at different time points (1–42 dpi) of infection
(DOCX)

S7 Table Strains used in this study. All the strains used in this study indicated with genotypes and references.
(DOCX)
