# Correction: Reprogramming of *Yersinia* from Virulent to Persistent Mode Revealed by Complex *In Vivo* RNA-seq Analysis

**DOI:** 10.1371/journal.ppat.1004769

**Published:** 2015-03-25

**Authors:** The PLOS Pathogens Staff

There are typographical errors in the published article.

There are three instances in the article that refer to “26S rRNAs”. This is incorrect. The correct text is “28S rRNAs”.

In the Abstract, “26oC” is incorrect. The celcius degree sign should be superscripted to read: “26°C”.

In the Fig. 1 legend, the following text has a typographical error: "Positive cells are brown (DAB) and the background is green. (methyl green)."

It correctly reads: "Positive cells are brown (DAB) and the background is green (methyl green)."
