# Supplementary figures and images for: Reprogramming of Yersinia from Virulent to Persistent Mode Revealed by Complex In Vivo RNA-seq Analysis
Source: PLoS Pathog. 2015 Jan 15;11(1):e1004600. doi: 10.1371/journal.ppat.1004600 (PMC4295882; doi:10.1371/journal.ppat.1004600)

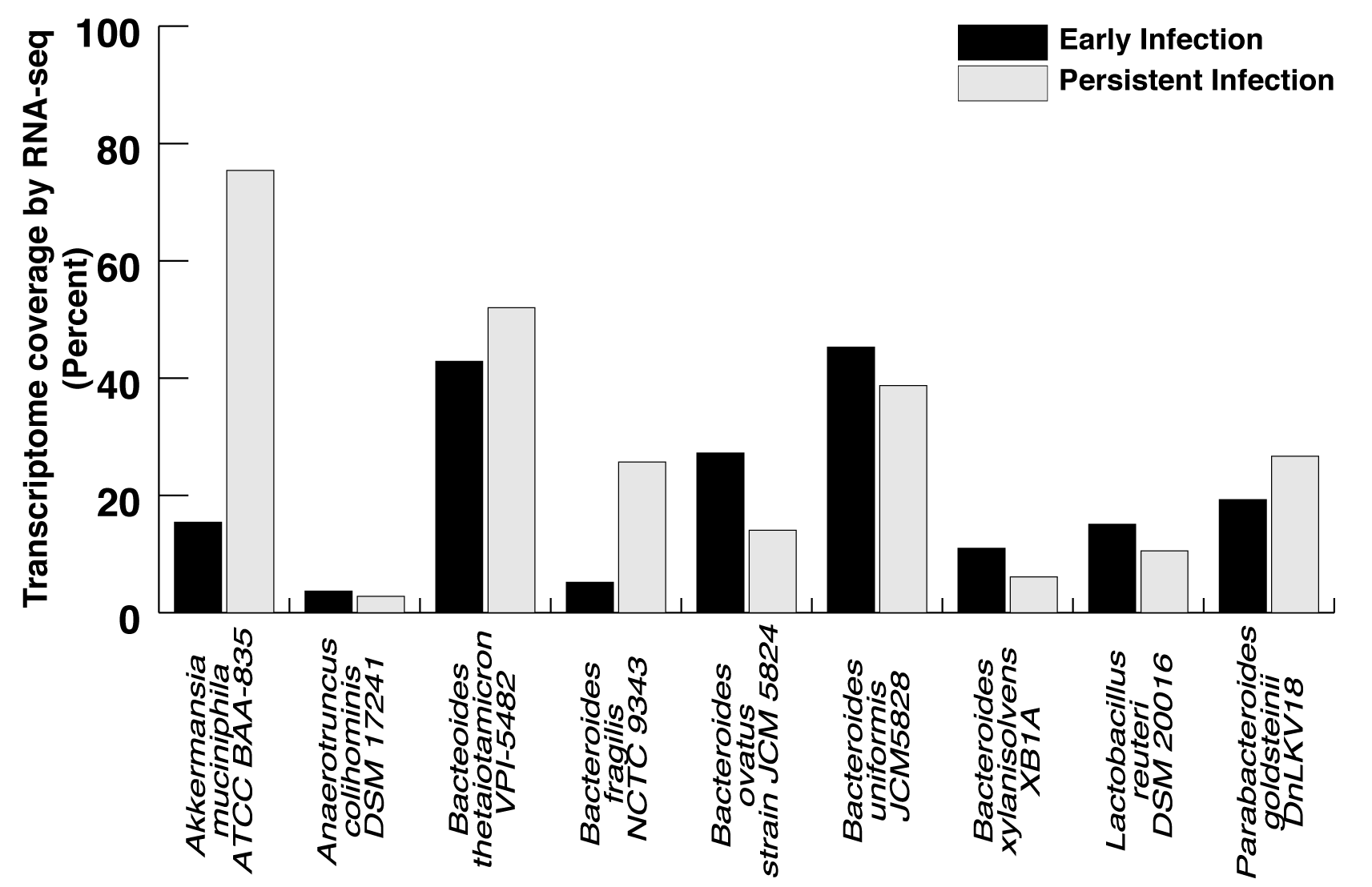

Supplement: S1 Fig — The transcriptome coverage of each indicated species was obtained using reference genomes, if available, of species identified by 16SMicrobial database mapping of persistent infection samples. Columns indicate the percent coverage of the species in both early and persistent infection samples. RNA-seq analyses were performed on rRNA-depleted cDNA libraries with CLC Genomic Workbench. (TIF) [file ppat.1004600.s001.tif]

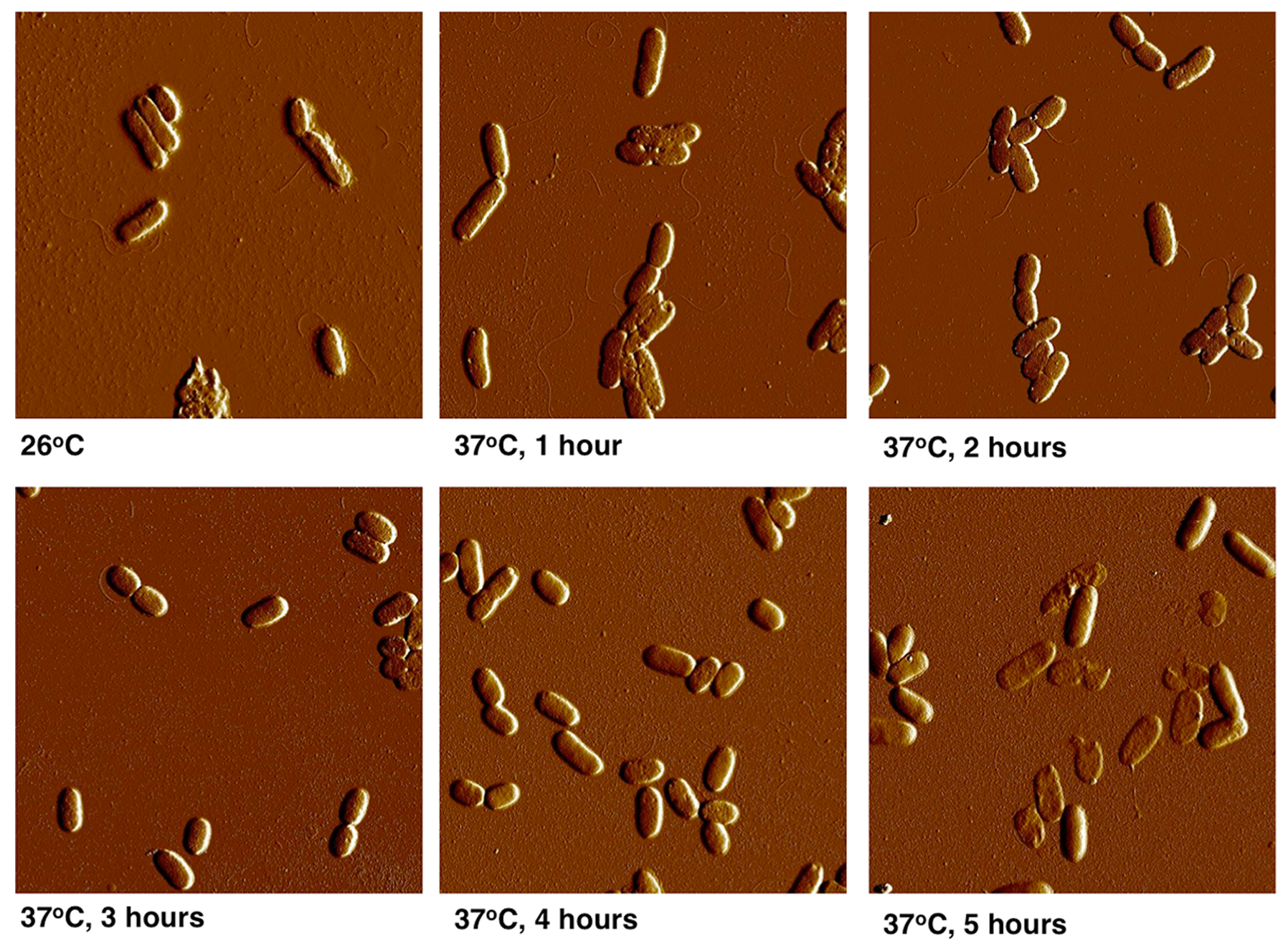

Supplement: S2 Fig — Visualization of Y. pseudotuberculosis flagella by atomic force microscopy. Wt Y. pseudotuberculosis were grown at exponential phase (26°C) and subjected to T3SS-inducing conditions (depleted Ca2+ at 37°C) and thereafter analysed every hour for 5 hours. (TIF) [file ppat.1004600.s002.tif]

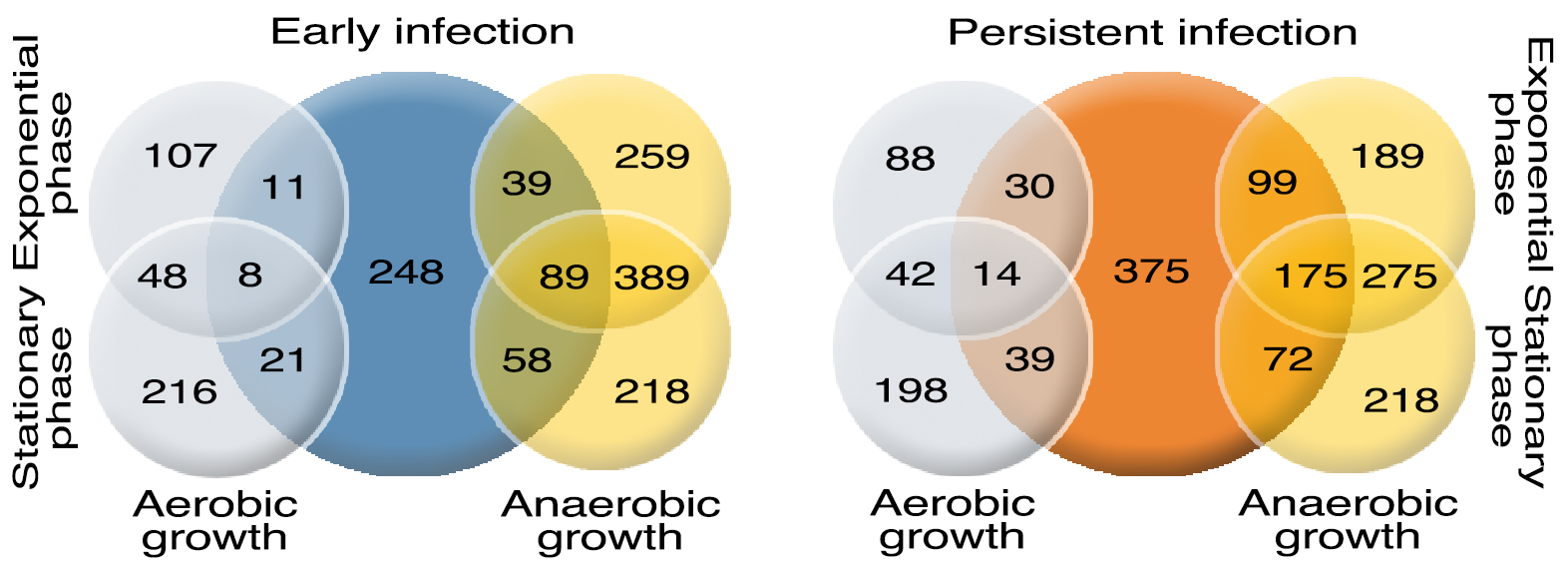

Supplement: S3 Fig — Venn diagram illustrating the number of overlapping genes up-regulated in vivo during early versus persistent infection with up-regulated genes during logarithmic and stationary growth in vitro. (TIF) [file ppat.1004600.s003.tif]

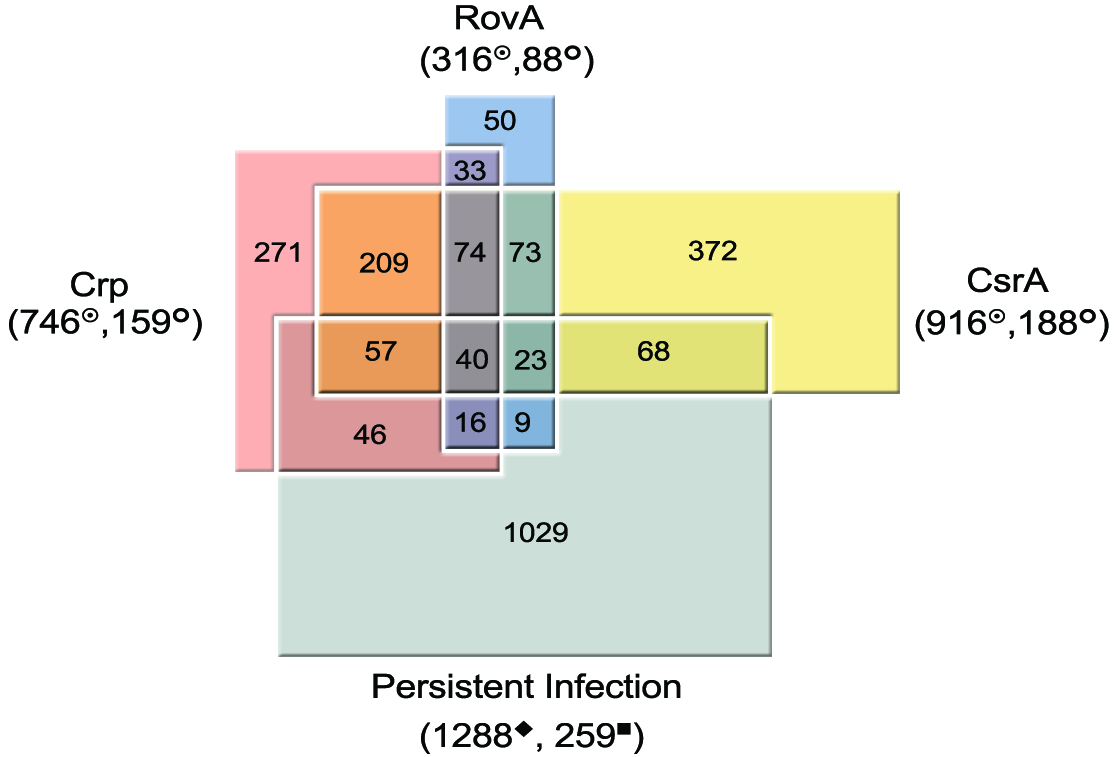

Supplement: S4 Fig — Venn diagram showing number of genes whose expression correlate with the CsrA, Crp, and RovA regulons [35] and during persistent infection (this study). Circle with centered dot indicates total number of the genes in RovA, Crp, and CsrA regulons. Circle indicates number of genes in RovA, Crp, and CsrA regulons whose expression pattern overlap with persistent infection. Diamond indicates total number of differentially expressed genes during persistent infection. Square indicates number of differentially expressed genes during persistent infection whose expression pattern overlaps with RovA, Crp, and CsrA regulons. (TIF) [file ppat.1004600.s004.tif]

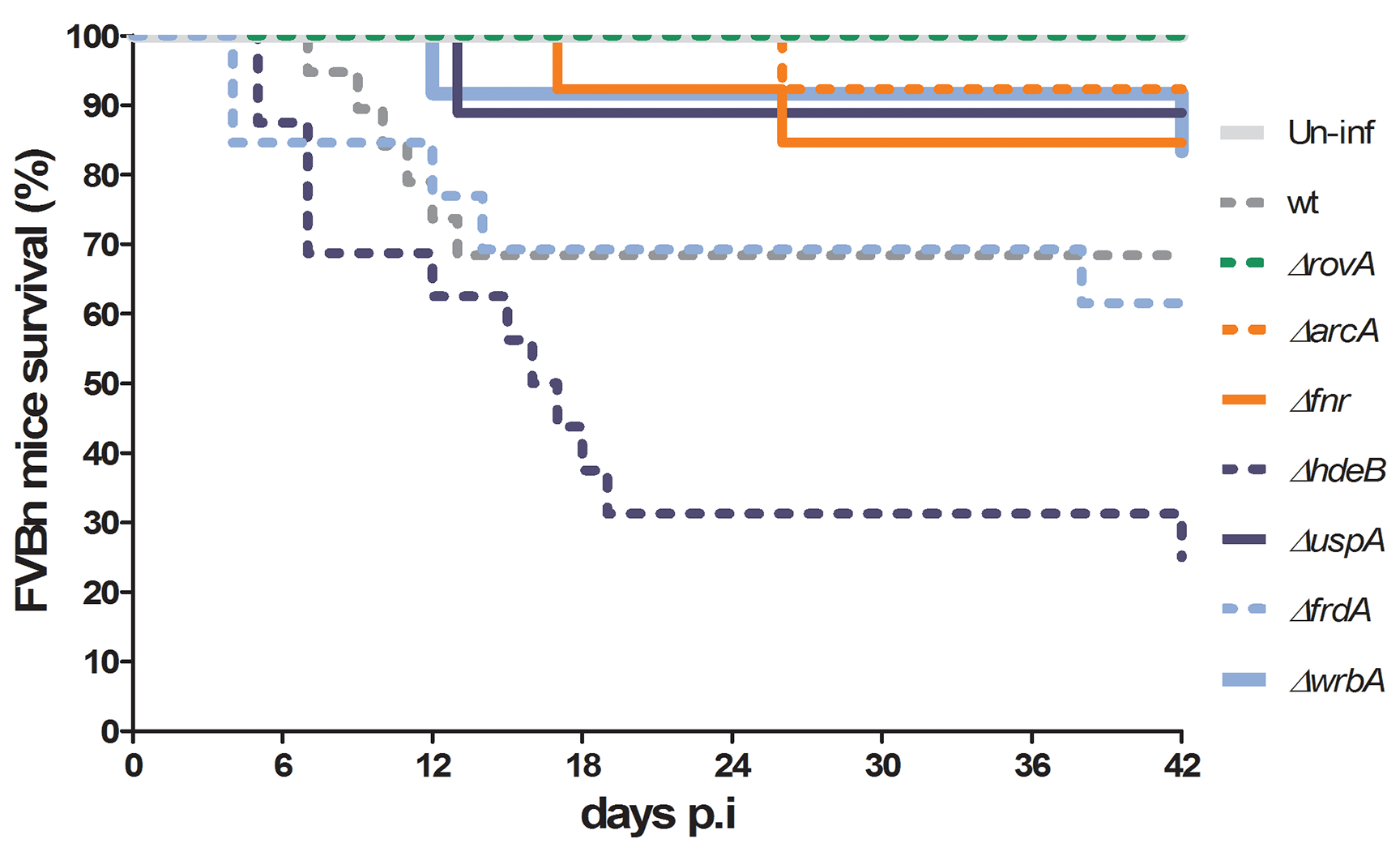

Supplement: S5 Fig — Low dose oral infection of FVBn mice with wt Y. pseudotuberculosis YPIII and the indicated mutant strain up to 42 dpi. Survival is given as a percentage of the total number of infected mice. (TIF) [file ppat.1004600.s005.tif]

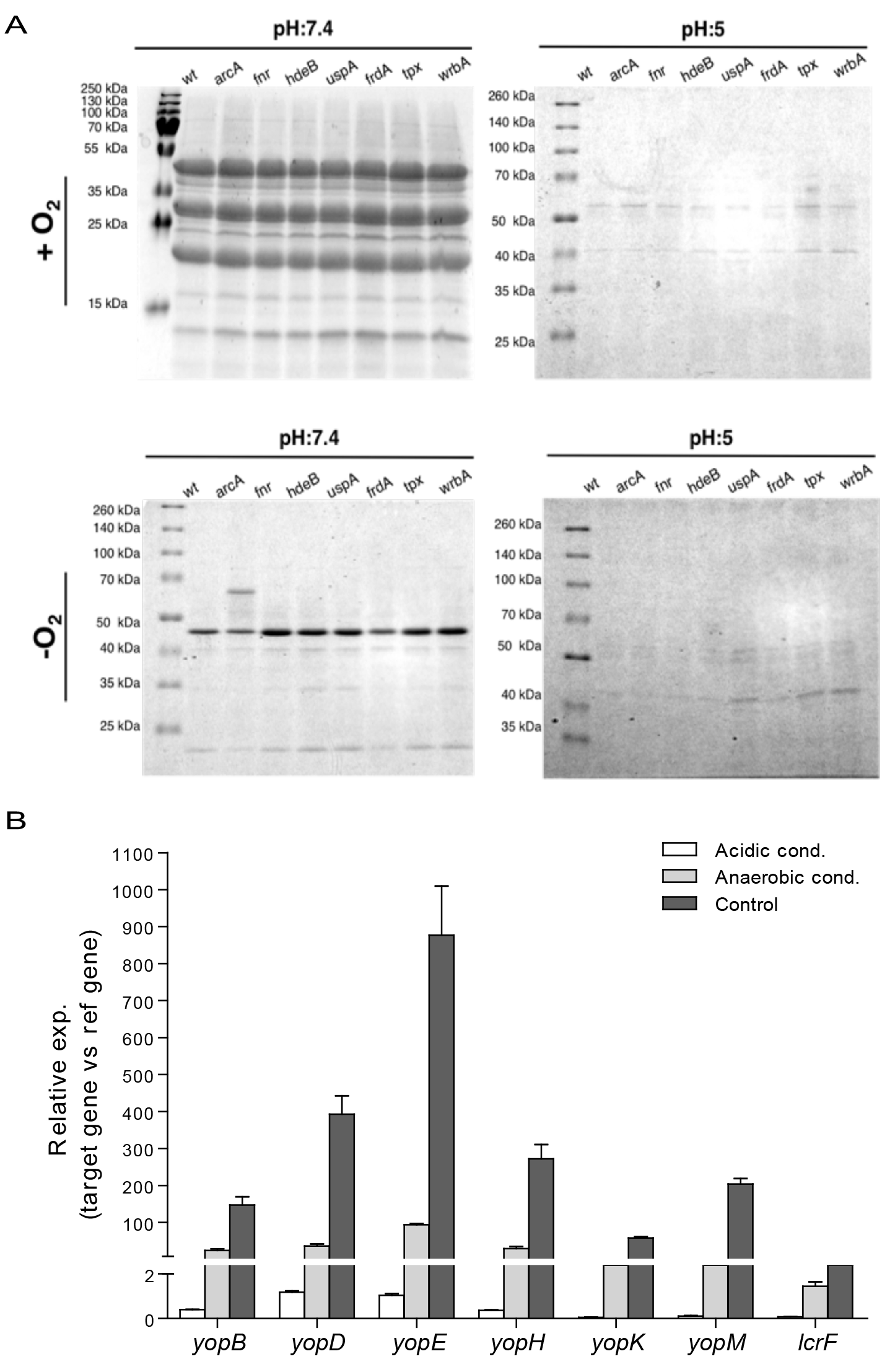

Supplement: S6 Fig — (A) Proteins secreted by Y. pseudotuberculosis wt and indicated mutant strains under T3SS-inducing conditions (depleted Ca2+ at 37°C) were analyzed in the presence and absence of oxygen and at normal and low pH. The secreted proteins were concentrated by TCA precipitation and loaded onto the gel according to each culture’s OD600 value. (B) The expression T3SS components in the presence and absence of oxygen in LB with normal (control) and low pH under T3SS-inducing conditions (depleted Ca+2 at 37°C) determined by qPCR. (TIF) [file ppat.1004600.s006.tif]

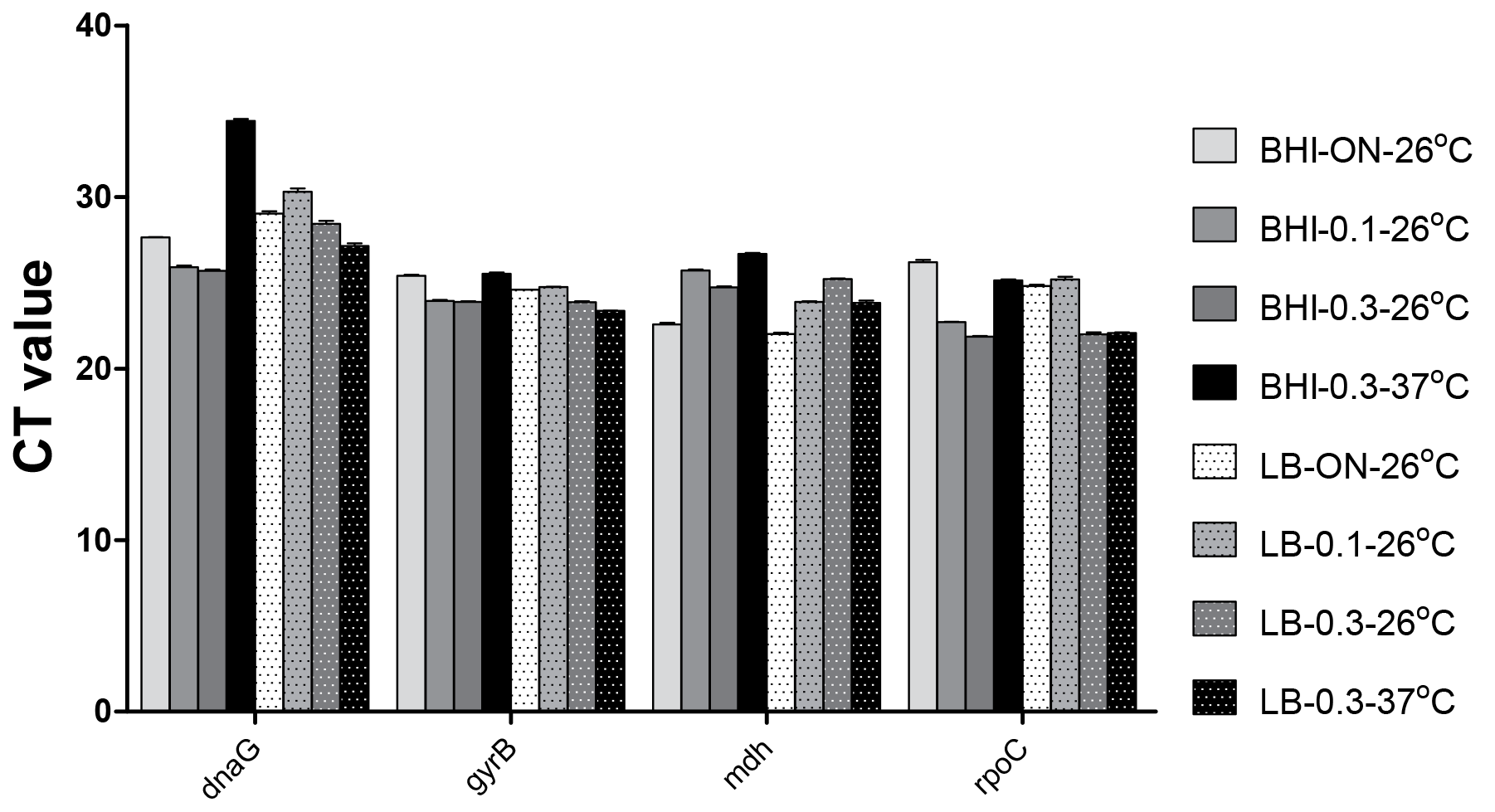

Supplement: S7 Fig — The expression levels of dnaG, gyrB, mdh, and rpoC under different growth conditions and temperatures were analyzed by qPCR. Bacterial cultures were grown in BHI/LB medium at OD600:0.1 and 0.3 at 26°C and OD600:0.3 at 37°C under T3SS-inducing conditions. The same amount of cDNA template was used in qPCR analysis. (TIF) [file ppat.1004600.s007.tif]
