# Supplementary material for: Reprogramming of Yersinia from Virulent to Persistent Mode Revealed by Complex In Vivo RNA-seq Analysis
Source: PLoS Pathog. 2015 Jan 15;11(1):e1004600. doi: 10.1371/journal.ppat.1004600 (PMC4295882; doi:10.1371/journal.ppat.1004600)
Supplement: S2 Table — The analyses were performed with CLC-Bio Genomics Workbench by using Transcriptomics Analysis Module. The data were obtained from two biological replicates from bacterial cultures grown at 26°C and 37°C virulence inducing conditions. The expression values are given as RPKM and differentially expressed genes were filtered with Log2 fold change ≥0,7. All data generated in this study were deposited in the Gene Expression Omnibus (GEO) database and are available under accession number GSE56477. (PDF) [file ppat.1004600.s009.pdf]

Table S2. *In vitro* (26ºC vs 37ºC) differentially expressed genes of *Yersinia pseudotuberculosis YPIII*. The analyses were performed with CLC-Bio Genomics Workbench by using Transcriptomics Analysis Module. The data were obtained from two biological replicates from bacterial cultures grown at 26ºC and 37ºC virulence inducing conditions. The expression values are given as RPKM and differentially expressed genes were filtered with Log2 fold change ≥0,7. All data generated in this study were deposited in the Gene Expression Omnibus (GEO) database and are available under accession number GSE56477.

Locus Tag	Definition	Log2-FC	26oC #1-RPKM	26oC #2-RPKM	26oC - RPKM Means	37oC #1-RPKM	37oC #2-RPKM	37oC - RPKM Means
YPK_2649	porin	7,38	53335,9	52992,2	53164,1	339,4	302,1	320,7
YPK_2438	ferroxidase	7,37	30614,1	31492,9	31053,5	189,6	184,8	187,2
YPK_2392	fliG flagellar motor switch protein G	7,33	95,8	92,9	94,3	0,4	1,7	1,0
YPK_0381	lamB maltoporin	7,26	721,7	630,2	676,0	5,6	3,5	4,6
YPK_2420	flgG flagellar basal body rod protein FlgG	6,70	217,4	208,2	212,8	1,4	4,3	2,8
YPK_1743	MgtC/SapB transporter	6,68	107,6	102,6	105,1	0,8	1,6	1,2
YPK_2425	flgB flagellar basal body rod protein FlgB	6,64	323,0	290,3	306,6	2,6	4,0	3,3
YPK_2383	fliS flagellar protein FliS	6,63	88,8	89,1	89,0	0,9	0,9	0,9
YPK_2384	flagellar biosynthesis protein FliT	6,52	71,3	74,2	72,8	0,5	2,0	1,2
YPK_2426	flgA flagellar basal body P-ring biosynthesis pro	6,45	159,8	161,1	160,5	4,6	1,1	2,9
YPK_0378	malE maltose ABC transporter periplasmic prot	6,05	364,6	337,2	350,9	9,9	3,5	6,7
YPK_2421	flgF flagellar basal body rod protein FlgF	6,02	63,9	58,6	61,3	0,7	1,5	1,1
YPK_2419	flgH flagellar basal body L-ring protein	6,02	49,7	44,3	47,0	0,5	1,3	0,9
YPK_2381	flagellin	6,01	2244,9	2142,2	2193,5	37,1	31,3	34,2
YPK_2429	invasin region 3	6,00	504,3	458,7	481,5	6,7	8,8	7,7
YPK_1954	hypothetical protein	5,93	143,2	130,5	136,9	2,9	1,8	2,3
YPK_2399	fliM flagellar motor switch protein FliM	5,93	72,5	70,9	71,7	1,2	1,1	1,2
YPK_2817	putative bacteriophage protein	5,89	127,5	110,4	118,9	1,7	2,4	2,1
YPK_2432	flhB flagellar biosynthesis protein FlhB	5,80	60,2	60,6	60,4	1,2	1,0	1,1
YPK_2400	fliN flagellar motor switch protein FliN	5,79	84,0	78,8	81,4	1,3	1,8	1,5
YPK_2393	fliH flagellar assembly protein H	5,79	47,5	51,1	49,3	0,5	3,4	1,9
YPK_2418	flgI flagellar basal body P-ring protein	5,78	61,1	57,4	59,2	0,6	3,9	2,2
YPK_2417	flgJ flagellar rod assembly protein/muramidase	5,77	79,1	77,3	78,2	1,1	2,0	1,6
YPK_1140	hdeB acid-resistance protein	5,76	57573,5	57403,3	57488,4	1125,0	1000,5	1062,7
YPK_2382	fliD flagellar capping protein	5,55	134,8	124,1	129,4	3,8	2,1	3,0
YPK_1744	magnesium-translocating P-type ATPase	5,24	119,4	113,4	116,4	3,8	2,6	3,2
YPK_1601	hypothetical protein	5,12	2781,7	2665,7	2723,7	83,9	73,1	78,5
YPK_2378	flagella biosynthesis protein FliZ	5,06	145,3	139,6	142,4	5,2	3,6	4,4
YPK_2390	fliE flagellar hook-basal body protein FliE	5,01	227,5	206,0	216,8	9,0	5,2	7,1
YPK_2809	putative bacteriophage tail fiber protein	4,97	88,4	86,5	87,5	6,0	1,8	3,9
YPK_2422	flgE flagellar hook protein FlgE	4,75	100,3	86,6	93,5	3,8	3,2	3,5
YPK_2395	fliJ flagellar biosynthesis chaperone	4,74	44,9	41,4	43,1	1,6	1,6	1,6
pYV0015		4,61	7,6	11,5	9,5	248,4	224,0	236,2
YPK_1953	putative virulence factor SrfB	4,55	121,8	108,1	114,9	4,5	5,5	5,0
YPK_2573	hypothetical protein	4,49	85,7	91,6	88,7	2,6	8,0	5,3
YPK_2761	transcriptional regulator CadC	4,38	475,3	506,4	490,8	18,7	31,2	25,0
YPK_0281	bacterioferritin	4,38	974,2	1018,1	996,1	47,0	48,9	47,9
YPK_2049	outer membrane protein W	4,37	2388,0	2252,6	2320,3	108,2	116,8	112,5
YPK_1742	methyl-accepting chemotaxis sensory transducer	4,36	26,1	30,3	28,2	1,3	1,4	1,4
YPK_2398	fliL flagellar basal body-associated protein FliL	4,32	43,0	40,2	41,6	1,9	2,3	2,1
YPK_0166	hypothetical protein	4,30	688,9	709,3	699,1	34,8	36,2	35,5
YPK_3393	dimethylsulfoxide reductase subunit B	4,09	35,3	37,2	36,3	1,4	3,9	2,7
YPK_0631	hypothetical protein	4,09	5039,9	5167,3	5103,6	298,9	301,3	300,1
YPK_1543	diguanylate phosphodiesterase	4,06	12,5	12,7	12,6	1,3	0,5	0,9
YPK_1659	hypothetical protein	4,04	243,4	222,7	233,1	17,6	11,7	14,7
YPK_1753	methyl-accepting chemotaxis sensory transducer	3,98	32,9	31,6	32,3	1,7	2,6	2,1
YPK_1876	transcriptional regulator SlyA	3,95	2719,5	2744,5	2732,0	203,9	157,1	180,5
YPK_0382	maltose regulon periplasmic protein	3,90	160,2	142,1	151,2	11,0	9,4	10,2
YPK_0792	autoinducer synthesis protein	3,88	356,4	340,4	348,4	22,4	25,0	23,7
YPK_2815	DNA circulation family protein	3,83	19,7	19,3	19,5	1,0	2,1	1,6
YPK_0077	histidine ammonia-lyase	3,83	61,0	55,3	58,1	3,5	5,0	4,2
YPK_2937	modA molybdate transporter periplasmic protein	3,81	189,3	194,2	191,8	12,8	14,7	13,8
YPK_2760	hypothetical protein	3,76	106,9	113,2	110,0	8,0	8,3	8,1
YPK_1141	voltage-gated potassium channel	3,70	305,2	313,9	309,5	23,9	23,7	23,8
YPK_0377	malF maltose transporter membrane protein	3,68	43,6	47,0	45,3	4,8	2,8	3,8
YPK_0633	hypothetical protein	3,64	144,8	130,5	137,7	10,8	11,3	11,0
YPK_2935	modC molybdate transporter ATP-binding protei	3,63	102,5	99,1	100,8	6,8	10,2	8,5
YPK_3632	hypothetical protein	3,62	183,4	193,6	188,5	13,9	17,1	15,5

YPK_2427		anti-sigma-28 factor FlgM	3,58	234,6	209,7	222,1	21,3	16,2	18,8
YPK_2185		hypothetical protein	3,48	4801,8	4712,5	4757,1	456,3	402,0	429,1
YPK_2820		putative bacteriophage protein	3,45	36,3	37,3	36,8	2,6	4,6	3,6
YPK_2962		cyd operon protein YbgT	3,41	1983,4	1876,1	1929,7	170,6	194,2	182,4
YPK_1863		superoxide dismutase	3,41	2307,6	2288,8	2298,2	241,6	195,7	218,6
YPK_2441		hypothetical protein	3,39	1170,7	1274,1	1222,4	115,1	118,1	116,6
YPK_2961		hypothetical protein	3,39	1456,7	1422,0	1439,3	120,6	160,4	140,5
YPK_2059		hypothetical protein	3,35	814,9	821,3	818,1	91,0	71,9	81,4
YPK_3229		helix-hairpin-helix repeat-containing competence	3,35	100,8	107,1	104,0	11,9	9,0	10,4
YPK_2415	flgL	flagellar hook-associated protein FlgL	3,33	140,0	129,8	134,9	14,6	12,3	13,4
YPK_2431	flhA	flagellar biosynthesis protein FlhA	3,33	36,7	38,9	37,8	2,9	5,1	4,0
YPK_1494		LemA family protein	3,32	51,4	54,8	53,1	4,9	5,8	5,3
YPK_0161		glycogen/starch/alpha-glucan phosphorylase	3,28	79,6	70,9	75,3	7,5	7,9	7,7
YPK_1748	motB	flagellar motor protein MotB	3,22	98,4	88,8	93,6	11,0	9,2	10,1
YPK_3816		fumarate reductase subunit D	3,20	1055,3	982,3	1018,8	95,5	134,0	114,7
YPK_2018		hypothetical protein	3,18	259,8	265,8	262,8	24,0	36,2	30,1
YPK_1585		hypothetical protein	3,18	240,2	221,0	230,6	21,5	31,8	26,6
YPK_3298		hypothetical protein	3,17	783,0	819,3	801,1	92,6	86,0	89,3
YPK_1105	mltD	membrane-bound lytic murein transglycos	3,10	321,9	295,3	308,6	36,4	35,7	36,1
YPK_2483		hypothetical protein	3,07	582,3	630,3	606,3	64,1	81,8	72,9
YPK_1129		ABC transporter-like protein	3,05	43,6	37,2	40,4	5,3	4,4	4,9
YPK_1454		putative anaerobic dimethyl sulfoxide reductases	3,02	29,6	32,7	31,1	3,4	4,3	3,9
YPK_1978		hypothetical protein	3,02	474,4	516,1	495,3	74,1	52,8	63,4
YPK_1153		hypothetical protein	2,98	6,0	5,5	5,8	0,7	0,7	0,7
YPK_2467		hypothetical protein	2,98	54,7	51,9	53,3	11,1	4,8	7,9
YPK_3163		hypothetical protein	2,97	151,3	154,8	153,1	20,0	19,2	19,6
YPK_0120		UspA domain-containing protein	2,96	8881,6	9198,1	9039,9	1218,3	1105,0	1161,6
YPK_2754		putative DNA-binding transcriptional regulator	2,94	99,2	91,4	95,3	10,4	15,5	13,0
YPK_3162		hypothetical protein	2,92	161,2	150,1	155,6	19,7	21,5	20,6
YPK_1125		extracellular solute-binding protein	2,90	34,0	29,4	31,7	5,3	3,4	4,4
YPK_2963		cytochrome d ubiquinol oxidase subunit II	2,89	1571,3	1423,8	1497,6	179,8	235,1	207,4
YPK_1750		purine-binding chemotaxis protein	2,88	160,1	164,0	162,0	19,2	25,5	22,3
YPK_3038		hypothetical protein	2,88	32,0	37,7	34,8	4,2	5,3	4,7
YPK_0200		hypothetical protein	2,88	245,4	263,8	254,6	38,7	31,5	35,1
YPK_3185		O-antigen biosynthesis protein Wxy	2,86	72,2	77,9	75,0	11,8	9,3	10,6
YPK_1673	bssS	biofilm formation regulatory protein BssS	2,85	1462,7	1545,6	1504,1	240,6	184,9	212,7
YPK_3359		anaerobic C4-dicarboxylate transporter	2,83	28,4	31,2	29,8	4,8	3,7	4,3
YPK_1772		hypothetical protein	2,81	334,4	353,3	343,8	52,7	45,8	49,3
YPK_0034		methyl-accepting chemotaxis sensory transducer	2,80	15,6	13,9	14,7	2,2	2,0	2,1
YPK_3010		glutamate and aspartate transporter subunit	2,79	2946,4	2790,1	2868,2	438,2	391,6	414,9
YPK_3590	rpsT	30S ribosomal protein S20	2,79	2716,6	2759,8	2738,2	423,1	372,0	397,6
YPK_2034		hypothetical protein	2,77	6,4	8,1	7,2	0,6	3,0	1,8
YPK_2439		DNA polymerase III subunit theta	2,75	194,9	214,7	204,8	30,7	30,4	30,6
YPK_1434		hypothetical protein	2,74	65,4	75,8	70,6	9,6	11,5	10,6
YPK_2879		hypothetical protein	2,74	330,4	297,5	314,0	57,2	39,1	48,2
YPK_2223		N-formylglutamate amidohydrolase	2,73	63,6	66,7	65,2	9,5	10,1	9,8
YPK_2448		hypothetical protein	2,72	155,4	133,9	144,6	29,6	16,8	23,2
YPK_2496		TRAP dicarboxylate transporter subunit DctP	2,71	89,9	85,1	87,5	11,0	17,4	14,2
YPK_3541		hypothetical protein	2,70	7,5	8,2	7,9	1,2	1,2	1,2
YPK_2385		AraC family transcriptional regulator	2,70	47,5	44,0	45,7	6,1	8,5	7,3
YPK_3061		hypothetical protein	2,68	58,0	66,9	62,5	8,3	11,5	9,9
YPK_0570		von Willebrand factor type A	2,68	145,4	150,4	147,9	20,3	26,6	23,5
YPK_1556		hypothetical protein	2,67	10,8	11,5	11,2	1,4	2,2	1,8
YPK_0121		universal stress protein UspB	2,67	54,0	53,5	53,7	10,4	7,1	8,7
YPK_3985	zntA	zinc/cadmium/mercury/lead-transporting A	2,65	32,7	31,8	32,3	5,2	5,1	5,1
YPK_2204		hypothetical protein	2,63	215,5	187,3	201,4	34,2	30,7	32,4
YPK_2218		chorismate binding-like protein	2,63	18,1	17,8	18,0	2,8	3,0	2,9
YPK_3281		hypothetical protein	2,62	4645,8	4552,7	4599,3	826,0	679,1	752,5
YPK_4108		hypothetical protein	2,62	1607,0	1709,0	1658,0	299,8	246,3	273,0
YPK_1617		hypothetical protein	2,62	14,7	13,5	14,1	4,4	1,5	2,9
YPK_4187		phosphatase	2,61	445,9	446,6	446,3	78,1	68,4	73,3
YPK_1747		flagellar motor protein MotA	2,61	60,4	54,8	57,6	9,9	9,0	9,4
YPK_1602		DNA starvation/stationary phase protectionprotei	2,60	1972,2	1940,6	1956,4	332,4	314,5	323,4
YPK_0569		stress protein	2,59	88,1	80,2	84,2	12,7	15,5	14,1
YPK_3412		arogenate dehydratase	2,57	73,4	72,1	72,7	13,3	11,4	12,3
YPK_3299		ApbE family lipoprotein	2,56	172,4	157,8	165,1	30,0	26,0	28,0
YPK_2694		cold-shock DNA-binding domain-containingprotei	2,55	2379,9	2284,7	2332,3	425,8	374,1	399,9
YPK_3060		Hcp1 family type VI secretion system effector	2,54	85,6	84,3	85,0	21,4	11,1	16,2
YPK_0057	xylF	D-xylose transporter subunit XylF	2,52	217,3	197,3	207,3	40,7	32,1	36,4
YPK_3631		hypothetical protein	2,52	47,7	42,0	44,9	9,4	6,5	8,0

YPK_2221	histidine utilization repressor	2,49	52,6	56,1	54,3	10,7	8,9	9,8
YPK_2484	AraC family transcriptional regulator	2,43	26,0	22,1	24,1	6,4	3,3	4,8
YPK_3184	group 1 glycosyl transferase	2,43	122,5	117,5	120,0	20,6	24,4	22,5
YPK_3303	Na(+)-translocating NADH-quinone reductasesut	2,43	337,4	306,3	321,8	56,3	64,3	60,3
YPK_2897	hypothetical protein	2,41	7,3	7,2	7,2	1,3	1,4	1,4
YPK_1759	chemotaxis regulator CheZ	2,40	167,8	151,6	159,7	30,6	29,8	30,2
YPK_1492	alkylphosphonate utilization operon proteinPhnA	2,39	258,2	230,0	244,1	45,7	47,5	46,6
YPK_0312	mscL large-conductance mechanosensitive cha	2,36	224,5	209,5	217,0	43,5	41,2	42,3
YPK_3192	CDP-6-deoxy-delta-3,4-glucoseen reductase	2,35	124,8	130,9	127,8	26,7	23,7	25,2
YPK_4040	PpiC-type peptidyl-prolyl cis-trans isomerase	2,35	137,6	131,8	134,7	24,1	29,4	26,7
YPK_2848	ribonucleotide-diphosphate reductase subunitalp	2,35	107,7	101,1	104,4	18,9	22,5	20,7
YPK_3070	hypothetical protein	2,34	22,0	23,1	22,6	4,0	5,0	4,5
YPK_3336	hypothetical protein	2,34	62,2	60,1	61,1	14,9	10,1	12,5
YPK_2964	cytochrome bd ubiquinol oxidase subunit I	2,33	913,0	877,6	895,3	160,9	199,4	180,1
YPK_0425	RelB antitoxin	2,33	49,7	53,3	51,5	11,0	9,6	10,3
YPK_1507	hypothetical protein	2,30	2001,8	2073,1	2037,4	407,7	417,0	412,3
YPK_0162	malQ 4-alpha-glucanotransferase	2,30	69,3	62,4	65,8	12,9	13,9	13,4
YPK_0215	XRE family transcriptional regulator	2,30	43,4	40,9	42,1	8,0	9,2	8,6
YPK_2471	hypothetical protein	2,30	65,7	70,7	68,2	15,0	13,0	14,0
YPK_3302	Na(+)-translocating NADH-quinone reductasesut	2,28	238,7	233,0	235,8	51,1	46,3	48,7
YPK_1482	hypothetical protein	2,27	3,5	4,1	3,8	0,6	1,0	0,8
YPK_3188	NAD-dependent epimerase/dehydratase	2,27	72,7	73,7	73,2	19,8	12,3	16,0
YPK_1001	carbonic anhydrase	2,27	1602,0	1468,5	1535,2	324,6	311,5	318,1
YPK_1826	ihfA integration host factor subunit alpha	2,26	3604,0	3343,8	3473,9	748,2	697,7	723,0
YPK_2643	hypothetical protein	2,25	141,5	145,0	143,2	36,6	25,7	31,2
YPK_3301	Na(+)-translocating NADH-quinone reductasesut	2,24	206,1	184,0	195,0	40,8	42,1	41,4
YPK_4216	flavodoxin	2,22	253,8	258,1	256,0	60,1	50,4	55,2
YPK_3578	folA dihydrofolate reductase	2,22	38,5	40,3	39,4	9,5	7,6	8,6
YPK_1128	ABC transporter-like protein	2,21	35,7	36,3	36,0	7,3	8,3	7,8
YPK_3738	hypothetical protein	2,21	497,0	468,9	483,0	104,6	103,7	104,1
YPK_0328	pantothenate kinase	2,21	120,1	111,6	115,8	31,8	20,3	26,0
YPK_3411	cytochrome-c peroxidase	2,20	86,5	80,3	83,4	22,0	15,3	18,6
YPK_2224	porin	2,20	11,3	11,6	11,4	2,8	2,2	2,5
YPK_1559	LysR family transcriptional regulator	2,18	452,8	489,7	471,2	112,2	97,7	105,0
YPK_2048	transport-associated	2,18	424,7	407,4	416,0	96,7	87,8	92,2
YPK_3353	sigma 54 modulation protein/30S ribosomalprote	2,17	17661,6	17291,3	17476,5	4234,9	3562,7	3898,8
YPK_2396	flagellar hook-length control protein	2,17	18,2	19,0	18,6	3,6	4,8	4,2
YPK_3368	S-ribosylhomocysteinase	2,17	2880,2	2767,0	2823,6	677,4	582,8	630,1
YPK_0268	FKBP-type peptidyl-prolyl cis-trans isomerase	2,17	476,5	464,2	470,4	94,0	118,3	106,1
YPK_0179	hypothetical protein	2,15	52,6	60,4	56,5	10,6	15,6	13,1
YPK_3942	twin arginine-targeting protein translocase	2,14	968,5	930,9	949,7	233,4	200,6	217,0
YPK_0122	phosphate transporter	2,13	70,0	68,8	69,4	13,4	19,4	16,4
YPK_1087	hypothetical protein	2,13	1023,7	965,0	994,3	228,5	225,1	226,8
YPK_2654	hypothetical protein	2,13	123,5	124,5	124,0	26,6	30,3	28,4
YPK_1395	hypothetical protein	2,13	5,0	4,2	4,6	1,3	0,9	1,1
YPK_2269	two component LuxR family transcriptionalregula	2,12	172,6	172,8	172,7	39,6	40,0	39,8
YPK_3638	diguanylate cyclase	2,12	75,5	71,2	73,4	17,5	16,4	16,9
YPK_1037	hypothetical protein	2,12	42,5	40,0	41,3	9,3	9,7	9,5
YPK_1676	antibiotic biosynthesis monooxygenase	2,11	713,1	722,0	717,5	180,6	155,0	167,8
YPK_0016	malS periplasmic alpha-amylase	2,10	7,6	8,8	8,2	1,4	2,8	2,1
YPK_0504	putative sigma(54) modulation protein	2,09	3801,6	3814,9	3808,2	970,8	830,9	900,9
YPK_2785	hypothetical protein	2,07	6,4	4,8	5,6	1,9	0,9	1,4
YPK_3372	carbon storage regulator	2,07	743,7	768,5	756,1	195,3	167,6	181,5
YPK_1749	chemotaxis protein CheA	2,07	73,6	67,4	70,5	16,3	17,5	16,9
YPK_3642	acriflavin resistance protein	2,06	42,3	41,8	42,1	9,9	10,2	10,1
YPK_1682	rpmF 50S ribosomal protein L32	2,04	3522,5	3609,8	3566,2	894,8	841,6	868,2
YPK_0447	3-hydroxyisobutyrate dehydrogenase	2,04	40,0	36,7	38,4	10,2	8,5	9,4
YPK_2881	hypothetical protein	2,03	127,4	123,3	125,3	34,4	27,7	31,1
YPK_1681	hypothetical protein	2,02	2552,8	2559,6	2556,2	660,9	599,0	629,9
YPK_1374	hypothetical protein	2,02	36,1	36,5	36,3	9,0	8,9	8,9
YPK_3546	DNA polymerase II	2,01	21,7	22,1	21,9	4,6	6,5	5,6
YPK_1733	AsnC family transcriptional regulator	2,00	148,0	146,0	147,0	32,6	42,0	37,3
YPK_4181	sodium/glutamate symporter	1,99	46,0	51,5	48,8	10,3	14,7	12,5
YPK_3358	putative two-component response-regulatoryprot	1,99	103,1	113,1	108,1	23,4	32,0	27,7
YPK_1174	autonomous glycyl radical cofactor GrcA	1,98	5952,0	6300,6	6126,3	1645,5	1466,0	1555,8
YPK_1495	hypothetical protein	1,98	21,0	16,0	18,5	7,6	3,1	5,3
YPK_3300	Na(+)-translocating NADH-quinone reductasesut	1,97	142,9	145,5	144,2	36,0	37,9	36,9
YPK_1792	oligogalacturonide lyase	1,96	118,2	117,9	118,0	33,0	28,2	30,6
YPK_3087	hypothetical protein	1,95	358,7	326,0	342,3	92,8	83,8	88,3
YPK_1144	PTS system N,N'-diacetylchitobiose-specifictrans	1,95	102,0	119,2	110,6	24,2	33,8	29,0

YPK_4177	rpoZ	DNA-directed RNA polymerase subunit om	1,95	549,5	582,6	566,1	152,8	141,1	147,0
YPK_3838		IS1 transposase	1,95	30,8	26,3	28,5	11,4	5,3	8,3
YPK_2678		glycosyl transferase family protein	1,94	30,0	28,6	29,3	7,1	8,2	7,7
YPK_3212		hypothetical protein	1,94	34,2	34,0	34,1	10,4	7,7	9,1
YPK_1024		diaminopimelate decarboxylase	1,94	23,6	19,2	21,4	7,1	4,4	5,8
YPK_4068		periplasmic binding protein/LacI transcriptionalre	1,93	97,3	99,5	98,4	28,4	23,9	26,1
YPK_0547		hypothetical protein	1,91	8303,4	8550,3	8426,8	2387,7	2116,0	2251,9
YPK_1952		putative virulence factor	1,90	15,0	14,7	14,8	5,0	3,3	4,2
YPK_0098		C4-dicarboxylate transporter DctA	1,90	118,8	125,8	122,3	33,1	32,6	32,8
YPK_0521		cytochrome d ubiquinol oxidase subunit III	1,90	1048,3	1057,3	1052,8	300,3	267,3	283,8
YPK_4096		transcriptional repressor protein MetJ	1,89	104,7	106,0	105,4	24,2	34,3	29,3
YPK_3792		transcriptional repressor NsrR	1,89	117,8	114,5	116,1	31,9	30,8	31,4
YPK_0354		transcriptional regulator HU subunit alpha	1,89	7527,8	7244,3	7386,1	2138,0	1867,4	2002,7
YPK_1819		hypothetical protein	1,89	436,2	440,3	438,3	136,1	105,2	120,6
YPK_3118		phage-related membrane protein	1,89	171,1	167,1	169,1	46,1	45,5	45,8
YPK_0159	glpE	thiosulfate sulfurtransferase	1,88	156,5	175,9	166,2	40,9	49,7	45,3
YPK_2628		competence-specific genes regulator	1,88	43,5	51,7	47,6	9,8	17,7	13,8
YPK_0998		outer membrane autotransporter	1,87	10,4	10,5	10,5	2,6	3,2	2,9
YPK_2482		hypothetical protein	1,86	184,2	203,6	193,9	49,8	57,1	53,5
YPK_0962		hypothetical protein	1,85	82,7	75,0	78,8	23,3	20,5	21,9
YPK_3202		primosomal replication priB and priC	1,85	51,4	52,6	52,0	13,9	15,1	14,5
YPK_3264	tgt	queuine tRNA-ribosyltransferase	1,85	49,8	46,6	48,2	12,7	14,2	13,5
YPK_1552		hypothetical protein	1,83	85,8	77,9	81,8	27,1	19,7	23,4
YPK_0638		putative glycerol-3-phosphate acyltransferasePls	1,83	141,9	146,7	144,3	42,9	38,6	40,8
YPK_3767		inorganic pyrophosphatase	1,83	1195,4	1268,5	1232,0	362,5	334,6	348,6
YPK_3286		YcgR family protein	1,82	48,4	47,6	48,0	12,4	14,9	13,7
YPK_3025		hypothetical protein	1,82	140,4	125,9	133,1	39,5	35,6	37,6
YPK_2802		colicin D	1,82	158,2	145,7	151,9	44,7	41,4	43,0
YPK_2561		cytidine deaminase	1,82	20,2	24,9	22,5	5,0	8,2	6,6
YPK_1645		CRISPR-associated helicase Cas3 family protein	1,81	26,1	25,6	25,9	6,9	7,9	7,4
YPK_3179		NAD-dependent epimerase/dehydratase	1,81	144,3	143,8	144,1	40,0	42,4	41,2
YPK_0446		beta-lactamase domain-containing protein	1,80	38,4	36,0	37,2	10,1	11,4	10,7
YPK_1746		transcriptional activator FlhC	1,79	283,2	312,2	297,7	85,1	86,9	86,0
YPK_3897		hypothetical protein	1,79	95,0	91,4	93,2	26,4	27,5	27,0
YPK_4219		F0F1 ATP synthase subunit I	1,79	191,0	186,7	188,9	55,0	54,3	54,7
YPK_2100		putative serine protein kinase PrkA	1,78	168,3	158,1	163,2	46,6	48,8	47,7
YPK_2574		hypothetical protein	1,77	1,7	1,7	1,7	0,5	0,5	0,5
YPK_2984		hypothetical protein	1,76	379,4	336,6	358,0	107,5	103,2	105,4
YPK_2309		hypothetical protein	1,76	180,3	157,7	169,0	54,0	46,0	50,0
YPK_3566		ImpA domain-containing protein	1,76	5,0	5,6	5,3	1,4	1,7	1,6
YPK_3775		adenosine-3'(2'),5'-bisphosphate nucleotidase	1,75	174,1	170,6	172,3	46,7	56,5	51,6
YPK_2308		hypothetical protein	1,75	57,3	58,8	58,0	17,6	16,9	17,2
YPK_3057		hypothetical protein	1,73	22,8	23,6	23,2	6,1	8,1	7,1
YPK_2239	hmsR	N-glycosyltransferase	1,72	11,8	11,6	11,7	3,2	3,9	3,6
YPK_3714		sterol-binding domain-containing protein	1,72	868,1	824,7	846,4	260,4	254,8	257,6
YPK_2733	grxA	glutaredoxin	1,70	576,4	594,9	585,6	204,3	161,0	182,7
YPK_3178		phosphomannomutase	1,70	191,2	187,9	189,6	59,6	56,8	58,2
YPK_3511		hypothetical protein	1,69	162,8	159,4	161,1	53,0	47,4	50,2
YPK_3078		hypothetical protein	1,68	26,0	24,2	25,1	8,6	7,1	7,9
YPK_1989		hypothetical protein	1,68	321,2	309,6	315,4	90,3	108,0	99,2
YPK_1529	truA	tRNA pseudouridine synthase A	1,68	34,4	41,2	37,8	8,3	18,1	13,2
YPK_2630		outer membrane protein A	1,68	10352,6	9589,2	9970,9	3154,4	3058,0	3106,2
YPK_1163		deoxyribodipyrimidine photolyase	1,67	47,5	43,2	45,4	17,2	12,0	14,6
YPK_2109		fatty acid metabolism regulator	1,67	337,1	332,6	334,9	110,1	100,3	105,2
YPK_3675		hypothetical protein	1,67	47,3	41,0	44,1	16,5	11,7	14,1
YPK_3821		hypothetical protein	1,67	676,4	661,1	668,7	200,5	221,2	210,8
YPK_1481		Hcp1 family type VI secretion system effector	1,67	50,3	54,7	52,5	16,5	16,5	16,5
YPK_3191		glucose-1-phosphate cytidylyltransferase	1,67	191,2	184,5	187,8	59,9	58,4	59,2
YPK_1138		urea transporter	1,66	25,6	26,8	26,2	7,9	8,6	8,3
YPK_1640		hypothetical protein	1,66	20,1	22,1	21,1	6,2	7,2	6,7
YPK_4075		hypothetical protein	1,66	554,6	565,3	559,9	200,9	158,1	179,5
YPK_2219		hypothetical protein	1,66	63,4	63,5	63,4	19,2	20,9	20,1
YPK_3181		mannose-1-phosphateguanylyltransferase/mann	1,65	94,6	92,6	93,6	34,5	26,2	30,3
YPK_1703		hypothetical protein	1,65	7,9	4,7	6,3	4,3	1,1	2,7
YPK_1669	tqsA	putative transport protein	1,65	34,6	31,4	33,0	11,7	9,5	10,6
YPK_2860		lysine decarboxylase	1,65	16,7	15,8	16,3	5,0	5,4	5,2
YPK_0225	aroK	shikimate kinase I	1,64	398,9	354,8	376,8	123,2	118,9	121,1
YPK_1811		fructosamine kinase	1,63	429,8	403,7	416,7	133,1	137,1	135,1
YPK_1544		phosphodiesterase	1,63	67,1	64,8	66,0	26,0	18,0	22,0
YPK_2108		SpoVR family protein	1,62	53,9	50,7	52,3	19,5	14,9	17,2

YPK_3285	hypothetical protein	1,62	121,6	138,0	129,8	36,8	48,9	42,8
YPK_2909	zraP zinc resistance protein	1,61	17,3	18,7	18,0	6,0	5,8	5,9
YPK_3725	lipoprotein NlpI	1,61	1568,3	1537,0	1552,6	535,9	484,9	510,4
YPK_3951	carboxymethylenebutenolidase	1,60	33,7	35,9	34,8	11,7	11,3	11,5
YPK_0248	cAMP-regulatory protein	1,59	953,1	961,4	957,3	333,7	303,0	318,3
YPK_2029	ribA GTP cyclohydrolase II	1,59	51,6	42,0	46,8	23,3	11,0	17,1
YPK_1725	hypothetical protein	1,58	974,3	990,5	982,4	364,5	297,9	331,2
YPK_3279	fructokinase	1,58	78,7	79,2	79,0	23,2	30,6	26,9
YPK_3263	yajC preprotein translocase subunit YajC	1,58	964,3	930,9	947,6	322,6	312,5	317,5
YPK_1674	DNA damage-inducible protein I	1,57	309,9	297,7	303,8	103,7	100,5	102,1
YPK_3425	RNA polymerase sigma factor RpoS	1,57	1082,5	1019,3	1050,9	338,1	371,7	354,9
YPK_1062	hypothetical protein	1,57	1106,6	1113,7	1110,1	421,2	336,0	378,6
YPK_3399	ABC transporter-like protein	1,57	10,5	10,1	10,3	3,2	3,8	3,5
YPK_0697	fimbrial protein	1,56	29,3	25,7	27,5	9,7	8,9	9,3
YPK_3237	transcriptional regulator BolA	1,55	239,3	234,9	237,1	78,0	83,9	81,0
YPK_4127	fieF ferrous iron efflux protein F	1,55	99,9	110,4	105,2	36,0	36,0	36,0
YPK_0082	sensory histidine kinase UhpB	1,55	32,8	34,5	33,6	10,0	13,5	11,7
YPK_2130	multidrug efflux system protein MdtI	1,55	14,3	16,4	15,3	4,2	6,6	5,4
YPK_2123	minE cell division topological specificity factorM	1,54	402,8	404,8	403,8	133,5	144,1	138,8
YPK_1996	DNA-binding transcriptional regulator AraC	1,54	68,8	68,1	68,5	20,9	27,1	24,0
YPK_4161	hypothetical protein	1,54	142,6	140,7	141,6	46,9	50,9	48,9
YPK_0317	DNA protecting protein DprA	1,53	42,7	44,5	43,6	13,7	16,6	15,2
YPK_2980	smpB SsrA-binding protein	1,53	485,2	477,0	481,1	167,4	165,0	166,2
YPK_2858	hypothetical protein	1,53	13,5	14,2	13,8	4,4	5,2	4,8
YPK_1657	hypothetical protein	1,53	6,8	6,8	6,8	2,3	2,4	2,4
YPK_1510	hypothetical protein	1,53	448,8	420,5	434,7	152,8	148,8	150,8
YPK_2070	extracellular solute-binding protein	1,52	832,4	833,8	833,1	291,6	287,8	289,7
YPK_1553	hypothetical protein	1,52	495,1	539,5	517,3	184,9	176,6	180,7
YPK_0732	hypothetical protein	1,51	23,0	26,8	24,9	7,7	9,9	8,8
YPK_3761	malate dehydrogenase	1,50	3557,2	3250,1	3403,7	1207,2	1192,4	1199,8
YPK_3820	hypothetical protein	1,50	141,0	151,4	146,2	53,9	50,0	51,9
YPK_4167	hypothetical protein	1,50	97,7	91,5	94,6	36,2	31,1	33,6
YPK_1931	hypothetical protein	1,49	76,4	75,8	76,1	28,8	25,5	27,2
YPK_1451	hypothetical protein	1,49	9,6	10,8	10,2	3,0	4,5	3,7
YPK_3326	ABC transporter-like protein	1,49	16,3	15,6	15,9	5,2	6,2	5,7
YPK_4155	radC DNA repair protein RadC	1,49	240,2	225,5	232,9	88,6	77,7	83,1
YPK_4033	undecaprenyl-phosphatealpha-N-acetylglucosam	1,48	52,4	51,9	52,2	18,2	19,2	18,7
YPK_2658	putative metallothionein SmtA	1,47	152,2	150,3	151,2	57,9	51,4	54,6
YPK_3183	GDP-mannose 4,6-dehydratase	1,47	162,5	160,3	161,4	57,3	59,4	58,3
YPK_0546	hypothetical protein	1,47	1615,5	1589,5	1602,5	590,7	567,8	579,2
YPK_4199	mobA molybdopterin-guanine dinucleotide biosy	1,46	30,2	29,5	29,9	10,0	11,9	10,9
YPK_1854	LPP repeat-containing protein	1,46	108055,7	110888,9	109472,3	41372,0	38390,4	39881,2
YPK_0505	RNA polymerase factor sigma-54	1,45	461,4	463,8	462,6	173,0	164,7	168,8
YPK_0322	hexapaptide repeat-containing transferase	1,45	195,4	199,2	197,3	71,8	72,6	72,2
YPK_2140	znuA high-affinity zinc transporter periplasmicpr	1,45	241,8	229,8	235,8	95,1	78,8	86,9
YPK_2960	acyl-CoA thioester hydrolase YbgC	1,45	226,2	197,1	211,6	86,8	69,3	78,0
YPK_4061	acetolactate synthase 2 catalytic subunit	1,43	16,1	16,2	16,1	6,6	5,4	6,0
YPK_3030	camphor resistance protein CrcB	1,43	5,8	6,4	6,1	1,8	2,9	2,3
YPK_2780	GntR family transcriptional regulator	1,42	53,2	55,0	54,1	17,7	23,4	20,6
YPK_4153	rpmG 50S ribosomal protein L33	1,42	1299,7	1355,6	1327,7	529,6	468,3	498,9
YPK_2955	peptidoglycan-associated outer membranelipopro	1,42	5839,6	5424,1	5631,8	2124,8	2087,1	2106,0
YPK_0558	serine/threonine transporter SstT	1,42	332,6	340,3	336,4	123,3	128,6	125,9
YPK_3955	hypothetical protein	1,41	140,5	126,4	133,4	51,2	49,1	50,1
YPK_1443	transmembrane pair domain-containing protein	1,40	14,7	14,3	14,5	5,2	5,8	5,5
YPK_2298	hypothetical protein	1,40	23,0	19,5	21,2	9,6	6,7	8,2
YPK_2623	hypothetical protein	1,40	37,4	41,1	39,2	13,3	16,6	15,0
YPK_1248	putative DNA-binding transcriptional regulator	1,40	13,2	11,8	12,5	5,7	4,0	4,9
YPK_1970	TnpA family transposase	1,40	12,9	11,8	12,4	5,3	4,2	4,7
YPK_1089	peptidyl-tRNA hydrolase domain-containingprotei	1,39	45,8	42,9	44,4	20,8	14,2	17,5
YPK_4215	DNA-binding transcriptional regulator AsnC	1,38	34,8	33,3	34,0	13,0	13,1	13,1
YPK_2849	nrdB ribonucleotide-diphosphate reductase sub	1,38	134,7	142,9	138,8	52,4	54,1	53,3
YPK_3177	ferric enterobactin transport protein FepE	1,38	109,4	114,1	111,7	43,4	42,6	43,0
YPK_3549	hypothetical protein	1,37	254,3	227,7	241,0	96,7	89,2	93,0
YPK_2388	death-on-curing family protein	1,37	195,4	197,6	196,5	79,4	72,9	76,1
YPK_3230	peptidyl-prolyl cis-trans isomerase D	1,37	301,4	297,4	299,4	130,0	104,1	117,1
YPK_0895	transcriptional activator Ogr/delta	1,37	80,1	77,3	78,7	29,3	31,9	30,6
YPK_2486	hypothetical protein	1,37	102,5	94,6	98,5	41,3	35,4	38,3
YPK_1536	3-octaprenyl-4-hydroxybenzoate carboxy-lyase	1,36	94,1	89,0	91,6	39,3	32,5	35,9
YPK_1793	IclR family transcriptional regulator	1,36	321,6	296,9	309,3	127,7	114,2	120,9
YPK_4175	ligB NAD-dependent DNA ligase LigB	1,36	13,9	13,6	13,8	5,0	5,9	5,4

YPK_3772	hypothetical protein	1,35	62,0	57,5	59,7	24,1	22,7	23,4
YPK_0894	hypothetical protein	1,34	27,6	25,2	26,4	12,0	9,1	10,6
YPK_4196	protein disulfide isomerase I	1,34	848,7	868,5	858,6	345,2	334,9	340,1
YPK_1994	hypothetical protein	1,33	25,5	26,6	26,0	9,6	11,2	10,4
YPK_3267	alkyl hydroperoxide reductase	1,33	7355,4	6840,5	7097,9	2948,4	2692,7	2820,5
YPK_2523	hypothetical protein	1,33	39,5	42,0	40,7	13,9	19,2	16,5
YPK_0056	xylose isomerase	1,32	13,7	14,2	14,0	5,8	5,4	5,6
YPK_0794	lipoprotein	1,32	12,5	9,4	11,0	5,8	3,3	4,6
YPK_4111	hypothetical protein	1,32	705,3	720,4	712,9	295,4	277,8	286,6
YPK_3646	O-antigen polymerase	1,31	51,4	47,6	49,5	19,8	20,1	19,9
YPK_1150	hypothetical protein	1,31	100,3	92,5	96,4	38,6	39,1	38,9
YPK_2667	ihfB integration host factor subunit beta	1,31	5537,0	5426,9	5482,0	2388,1	2054,1	2221,1
YPK_3102	XRE family transcriptional regulator	1,29	286,6	278,3	282,5	128,6	104,0	116,3
YPK_0660	LysR family transcriptional regulator	1,29	39,1	42,2	40,7	13,9	20,2	17,0
YPK_3503	coaE dephospho-CoA kinase	1,29	51,3	50,3	50,8	20,3	21,1	20,7
YPK_0650	hypothetical protein	1,29	6,7	8,9	7,8	2,5	4,0	3,3
YPK_0995	DeoR family transcriptional regulator	1,29	183,7	178,3	181,0	72,5	75,6	74,1
YPK_0635	dnaG DNA primase	1,28	50,9	51,4	51,1	21,3	20,8	21,0
YPK_0575	hypothetical protein	1,28	30,2	30,1	30,1	11,7	13,3	12,5
YPK_1423	RND family efflux transporter MFP subunit	1,28	12,9	9,3	11,1	6,1	3,4	4,7
YPK_2238	hmsS hemin storage system protein	1,28	10,8	18,9	14,9	3,4	11,4	7,4
YPK_3729	rbfA ribosome-binding factor A	1,27	195,3	175,7	185,5	79,8	73,6	76,7
YPK_0565	putative kinase protein	1,27	11,4	13,9	12,6	4,4	6,2	5,3
YPK_4158	slmA nucleoid occlusion protein	1,26	53,1	54,3	53,7	21,7	23,2	22,5
YPK_2835	putative transport protein	1,25	9,1	10,0	9,6	3,3	5,0	4,1
YPK_0636	rpsU 30S ribosomal protein S21	1,25	578,4	572,3	575,4	254,9	230,1	242,5
YPK_3241	cytochrome o ubiquinol oxidase subunit II	1,25	725,1	678,3	701,7	298,8	291,2	295,0
YPK_0536	hypothetical protein	1,25	233,5	230,7	232,1	96,5	99,0	97,7
YPK_2444	hypothetical protein	1,25	237,3	245,9	241,6	102,5	101,2	101,8
YPK_1716	putative lipoprotein	1,25	8,0	7,6	7,8	3,4	3,2	3,3
YPK_4098	rpmE 50S ribosomal protein L31	1,24	1581,9	1638,2	1610,0	684,8	675,3	680,1
YPK_2731	hypothetical protein	1,24	38,6	44,9	41,7	13,8	23,1	18,5
YPK_1825	pheT phenylalanyl-tRNA synthetase subunit bet	1,24	288,0	275,8	281,9	114,5	124,5	119,5
YPK_2079	hypothetical protein	1,24	90,9	79,6	85,3	44,5	29,6	37,1
YPK_1431	cell division protein ZipA	1,24	380,4	379,9	380,1	161,2	161,0	161,1
YPK_3272	proY putative proline-specific permease	1,24	49,2	49,4	49,3	22,2	19,8	21,0
YPK_1545	NUDIX hydrolase	1,23	51,7	51,0	51,3	20,7	23,2	21,9
YPK_3979	signal recognition particle-docking proteinFtsY	1,23	91,5	93,5	92,5	40,0	38,8	39,4
YPK_2747	glutathione S-transferase domain-containingprote	1,23	25,8	23,4	24,6	11,2	9,8	10,5
YPK_2178	N5-glutamine S-adenosyl-L-methionine-depende	1,22	66,0	57,6	61,8	29,8	23,4	26,6
YPK_4176	gmk guanylate kinase	1,22	130,7	125,9	128,3	56,7	53,6	55,1
YPK_1477	fimbrial biogenesis outer membrane usherproteir	1,22	2,8	3,6	3,2	1,1	1,7	1,4
YPK_2693	clpS ATP-dependent Clp protease adaptor prote	1,21	299,4	271,0	285,2	126,0	119,7	122,8
YPK_0334	nusG transcription antitermination protein NusG	1,21	454,4	439,0	446,7	206,9	179,6	193,3
YPK_2924	hypothetical protein	1,21	10,1	8,6	9,4	5,2	3,2	4,2
YPK_3289	DNA-binding transcriptional regulator Crl	1,20	544,6	558,3	551,4	251,2	230,1	240,6
YPK_3999	phospholipase A	1,20	200,7	194,6	197,7	86,9	85,5	86,2
YPK_0271	YheO domain-containing protein	1,20	145,4	144,0	144,7	65,8	60,7	63,2
YPK_3668	integrase family protein	1,20	6,3	9,8	8,1	2,2	5,7	4,0
YPK_2669	cmk cytidylate kinase	1,20	230,5	218,0	224,3	99,2	96,7	97,9
YPK_1869	lactoylglutathione lyase	1,19	183,3	168,4	175,8	81,7	72,3	77,0
YPK_1891	binding-protein-dependent transport system inne	1,19	18,1	19,0	18,5	7,6	8,7	8,1
YPK_1040	prepilin peptidase dependent protein-likeprotein	1,19	19,1	18,5	18,8	9,0	7,6	8,3
YPK_4200	molybdopterin-guanine dinucleotide biosynthesis	1,19	42,1	39,8	41,0	21,0	15,6	18,3
YPK_3495	regulatory protein AmpE	1,19	40,2	44,5	42,4	15,9	22,1	19,0
YPK_3494	amino acid permease-associated protein	1,18	26,3	26,9	26,6	11,2	12,4	11,8
YPK_2068	binding-protein-dependent transport system inne	1,17	117,2	110,8	114,0	51,5	50,0	50,7
YPK_0952	S-type pyocin domain-containing protein	1,16	7,0	10,3	8,6	2,7	5,5	4,1
YPK_3860	LexA repressor	1,16	99,0	88,3	93,6	44,6	39,1	41,9
YPK_0491	hypothetical protein	1,16	130,9	135,5	133,2	60,4	58,8	59,6
YPK_4149	3-deoxy-D-manno-octulosonic-acid transferase	1,16	74,9	73,8	74,3	31,6	35,1	33,4
YPK_3235	tig trigger factor	1,15	752,0	738,1	745,1	349,4	321,1	335,2
YPK_0517	BolA family protein	1,15	104,8	115,7	110,3	44,8	54,8	49,8
YPK_3527	cell division protein MraZ	1,15	248,6	259,5	254,0	109,0	120,1	114,5
YPK_2205	Sel1 domain-containing protein	1,15	6,8	8,0	7,4	2,6	4,3	3,5
YPK_0765	hypothetical protein	1,14	1,9	2,3	2,1	0,8	1,1	1,0
YPK_3146	hypothetical protein	1,14	252,1	255,7	253,9	122,7	108,2	115,5
YPK_4207	major facilitator transporter	1,14	24,6	24,7	24,6	11,1	11,3	11,2
YPK_1252	putative transglycosylase	1,14	22,8	21,1	21,9	10,9	9,1	10,0
YPK_0353	hypothetical protein	1,14	284,0	275,6	279,8	139,1	116,8	128,0

YPK_1033		dinucleoside polyphosphate hydrolase	1,14	98,0	108,6	103,3	43,6	50,6	47,1
YPK_1475		fimbrial protein	1,13	20,0	26,0	23,0	8,4	12,8	10,6
YPK_3159		multifunctional acyl-CoA thioesterase I/proteaseI/	1,13	8,9	9,1	9,0	4,3	3,9	4,1
YPK_2558		major facilitator transporter	1,13	14,2	14,9	14,5	6,8	6,5	6,6
YPK_0399		type VI secretion protein IcmF	1,13	3,9	4,6	4,2	1,7	2,2	2,0
YPK_1998	araG	L-arabinose transporter ATP-binding prote	1,13	8,8	10,2	9,5	3,5	5,5	4,5
YPK_2007		electron transport complex protein RnfB	1,13	44,6	46,9	45,8	18,5	24,0	21,2
YPK_3524		peptidoglycan glycosyltransferase	1,12	348,6	345,7	347,1	160,7	158,1	159,4
YPK_3499		hypothetical protein	1,12	9,0	9,0	9,0	4,4	3,9	4,2
YPK_2710	artP	arginine transporter ATP-binding subunit	1,12	8,1	9,2	8,6	3,7	4,3	4,0
YPK_3734	glmM	phosphoglucosamine mutase	1,12	382,5	355,4	369,0	178,2	161,5	169,8
YPK_0131		two component transcriptional regulator	1,12	38,2	37,0	37,6	16,4	18,3	17,4
YPK_1823	rplT	50S ribosomal protein L20	1,12	2733,1	2655,0	2694,1	1286,3	1197,4	1241,9
pYV0006		hypothetical protein	1,11	264,8	244,0	254,4	134,1	104,8	119,4
YPK_3034		hypothetical protein	1,10	13,4	12,5	13,0	5,9	6,2	6,0
YPK_2603		putative acyl carrier protein	1,10	58,7	68,2	63,4	27,2	31,9	29,5
YPK_4150		glycosyl transferase family protein	1,10	83,2	83,8	83,5	40,8	37,2	39,0
YPK_1822	rpmI	50S ribosomal protein L35	1,10	3575,7	3471,0	3523,3	1756,4	1539,7	1648,1
YPK_4190	glnL	nitrogen regulation protein NR(II)	1,10	20,6	25,3	23,0	9,2	12,4	10,8
YPK_0516		putative anti-sigma B factor antagonist	1,10	171,4	170,2	170,8	84,2	75,6	79,9
YPK_0332		elongation factor Tu	1,09	2256,8	2151,4	2204,1	1075,2	992,8	1034,0
YPK_3614		HAD superfamily ATPase	1,08	22,0	23,6	22,8	10,0	11,6	10,8
YPK_2798		sulfatase	1,08	26,7	27,6	27,1	11,8	14,1	13,0
YPK_2709		chorismate mutase	1,07	384,7	373,9	379,3	181,5	178,5	180,0
YPK_4229		glucosamine--fructose-6-phosphateaminotransfe	1,07	407,3	390,0	398,6	186,4	192,7	189,6
YPK_1821		translation initiation factor IF-3	1,07	5772,7	5505,4	5639,0	2751,4	2611,0	2681,2
YPK_2550		putative assembly protein	1,07	76,5	79,0	77,8	33,7	41,1	37,4
YPK_1687	acpP	acyl carrier protein	1,07	4870,2	4968,6	4919,4	2433,9	2267,1	2350,5
YPK_3189		DegT/DnrJ/EryC1/StrS aminotransferase	1,07	181,4	180,2	180,8	83,7	88,9	86,3
YPK_0320		putative ribosome maturation factor	1,06	206,2	192,5	199,4	98,2	92,4	95,3
YPK_1712		peptidase T	1,06	198,0	182,1	190,0	94,1	87,7	90,9
YPK_1180		methyltransferase small	1,06	48,6	59,3	54,0	22,1	30,0	26,1
YPK_2626		hypothetical protein	1,06	131,0	136,0	133,5	66,0	62,4	64,2
YPK_0313	trkA	potassium transporter peripheral membrane	1,05	28,6	30,0	29,3	13,8	14,5	14,1
YPK_0956		hypothetical protein	1,05	17,2	16,7	16,9	8,3	8,0	8,2
YPK_3956		hypothetical protein	1,05	112,2	114,3	113,2	52,7	57,0	54,8
YPK_3930		potassium transporter	1,04	20,6	22,5	21,6	10,3	10,7	10,5
YPK_1589		2-succinyl-5-enolpyruvyl-6-hydroxy-3-cyclohexen	1,04	21,8	25,0	23,4	9,9	13,0	11,5
YPK_3238		hypothetical protein	1,04	380,4	366,5	373,4	189,2	175,0	182,1
YPK_3898		hypothetical protein	1,04	47,2	44,1	45,6	22,7	21,8	22,2
YPK_0861		Z-ring-associated protein	1,03	506,1	519,5	512,8	256,5	246,2	251,4
YPK_3397		DeoR family transcriptional regulator	1,03	158,9	151,5	155,2	83,7	69,6	76,6
YPK_1394		putative acetyltransferase	1,03	42,5	31,9	37,2	25,3	13,3	19,3
YPK_3716		peptidase U32	1,02	34,9	40,0	37,5	16,7	20,5	18,6
pYV0007		replication protein	1,01	1057,1	1086,4	1071,8	555,4	508,6	532,0
YPK_1968		class I and II aminotransferase	1,01	38,0	33,6	35,8	19,6	16,1	17,8
YPK_1699		thiamine kinase	1,01	19,6	20,5	20,0	10,1	9,8	10,0
YPK_3739	greA	transcription elongation factor GreA	1,00	557,1	535,5	546,3	283,7	261,3	272,5
YPK_2050		hypothetical protein	1,00	53,5	61,8	57,7	25,2	32,9	29,1
YPK_3190		CDP-glucose 4,6-dehydratase	1,00	83,4	78,3	80,8	42,0	39,0	40,5
YPK_0411		hypothetical protein	1,00	25,5	31,0	28,2	12,4	16,1	14,2
YPK_1534		colicin V ion protein	1,00	52,3	47,9	50,1	27,8	22,7	25,3
YPK_2642		MOSC domain-containing protein	0,99	32,8	32,9	32,9	15,8	17,3	16,5
YPK_3569	djlA	Dna-J like membrane chaperone protein	0,99	42,3	39,2	40,8	22,0	19,1	20,6
YPK_0010		hypothetical protein	0,99	54,9	55,0	54,9	26,9	28,5	27,7
YPK_3278		SMC domain-containing protein	0,99	36,9	36,9	36,9	18,9	18,3	18,6
YPK_0128		hypothetical protein	0,98	14,9	11,4	13,2	8,1	5,4	6,7
YPK_0387		EvpB family type VI secretion protein	0,98	23,2	21,9	22,6	11,6	11,2	11,4
YPK_2139		hypothetical protein	0,98	208,5	216,3	212,4	109,6	105,7	107,6
YPK_2793		16S rRNA pseudouridylate synthase A	0,98	55,9	58,3	57,1	26,2	32,2	29,2
YPK_0304	rpmJ	50S ribosomal protein L36	0,98	807,9	812,6	810,2	430,9	393,2	412,0
YPK_3623		hypothetical protein	0,98	25,2	26,8	26,0	12,7	13,7	13,2
YPK_1768		patatin	0,98	36,0	35,8	35,9	18,2	18,2	18,2
YPK_4146	rfaD	ADP-L-glycero-D-manno-heptose-6-epimer	0,97	449,6	424,8	437,2	223,7	222,0	222,8
YPK_4014		hypothetical protein	0,97	63,9	70,0	67,0	31,1	37,5	34,3
YPK_0227		hypothetical protein	0,96	230,3	225,0	227,7	119,9	113,6	116,7
YPK_2355	uvrC	excinuclease ABC subunit C	0,95	88,9	86,4	87,6	45,8	44,7	45,3
YPK_3446	eno	phosphopyruvate hydratase	0,94	2147,0	2025,0	2086,0	1134,2	1035,3	1084,8
YPK_2480		hypothetical protein	0,94	76,5	83,2	79,8	41,0	42,0	41,5
YPK_2929		biotin biosynthesis protein BioC	0,94	2,4	3,4	2,9	1,1	2,1	1,6

YPK_2051		sulfate transporter	0,94	36,7	35,5	36,1	19,0	18,7	18,8
YPK_3997		threonine efflux system	0,94	30,6	26,0	28,3	16,6	13,1	14,8
YPK_3240		hypothetical protein	0,94	84,6	75,1	79,9	48,1	36,3	42,2
YPK_3634	rimI	ribosomal-protein-alanine N-acetyltransfera	0,94	25,8	31,6	28,7	13,1	17,0	15,0
YPK_2989		LexA regulated protein	0,94	43,9	43,4	43,6	23,6	22,0	22,8
YPK_2975		ATP-NAD/AcoX kinase	0,93	39,1	31,1	35,1	23,0	14,7	18,8
YPK_3105		hypothetical protein	0,93	239,0	234,5	236,8	128,1	120,8	124,5
YPK_3169		hypothetical protein	0,93	57,2	38,4	47,8	36,1	17,3	26,7
YPK_2499		two component LuxR family transcriptionalregula	0,93	9,7	12,0	10,8	4,8	6,7	5,8
YPK_4058		hypothetical protein	0,91	10,2	9,4	9,8	6,1	4,5	5,3
YPK_2498		TRAP dicarboxylate transporter subunit DctM	0,91	28,5	29,1	28,8	15,2	15,4	15,3
YPK_1504		cytochrome c-type biogenesis protein CcmI	0,91	68,6	63,3	65,9	36,6	33,7	35,2
YPK_0272		sulfur transfer complex subunit TusD	0,90	83,9	77,8	80,8	45,4	41,2	43,3
YPK_1987		hypothetical protein	0,90	395,5	386,8	391,2	211,4	207,9	209,6
YPK_2071		hypothetical protein	0,90	18,8	23,4	21,1	8,8	14,6	11,7
YPK_1904		mandelate racemase/muconate lactonizing prote	0,89	22,2	20,3	21,3	12,3	10,7	11,5
YPK_1351		phosphoribosylaminoimidazole synthetase	0,89	35,5	33,5	34,5	19,7	17,6	18,7
YPK_2242		putative endopeptidase	0,89	29,6	31,0	30,3	14,9	18,2	16,5
YPK_3176		inosine kinase	0,88	41,8	40,7	41,3	22,4	22,4	22,4
YPK_0822		hypothetical protein	0,88	251,2	235,7	243,4	132,9	132,0	132,5
YPK_0001	dnaA	chromosomal replication initiation protein	0,87	66,7	68,3	67,5	36,0	37,7	36,9
YPK_2375		polar amino acid ABC transporter inner membrar	0,87	13,1	16,7	14,9	6,4	10,3	8,4
YPK_4183		AsmA family protein	0,87	55,1	55,3	55,2	29,7	30,9	30,3
YPK_4086		TonB-dependent heme/hemoglobin receptor fami	0,86	1,4	1,4	1,4	0,8	0,8	0,8
YPK_3896		LysR family transcriptional regulator	0,86	11,3	11,2	11,3	6,2	6,2	6,2
YPK_1606	ompX	outer membrane protein X	0,86	238,1	218,1	228,1	131,6	120,0	125,8
YPK_2129		endoribonuclease L-PSP	0,86	214,1	194,7	204,4	122,1	104,2	113,1
YPK_1668		hypothetical protein	0,86	26,5	30,6	28,5	14,1	17,6	15,8
YPK_2243		tellurite resistance protein TehB	0,86	31,8	29,3	30,5	17,9	15,9	16,9
YPK_0682		type II secretion system protein E	0,85	1,8	1,2	1,5	1,1	0,6	0,8
YPK_4073	btuB	vitamin B12/cobalamin outer membranetra	0,85	22,7	21,0	21,9	13,3	11,0	12,2
YPK_2833		Cache sensor-containing methyl-acceptingchemo	0,85	8,5	8,8	8,7	4,9	4,7	4,8
YPK_2180	hemA	glutamyl-tRNA reductase	0,85	80,2	85,8	83,0	43,1	49,2	46,2
YPK_0133		pirin domain-containing protein	0,85	16,6	18,7	17,6	8,4	11,4	9,9
YPK_2397		hypothetical protein	0,85	11,4	12,5	12,0	0,0	0,0	0,0
YPK_1130		hypothetical protein	0,85	25,7	24,5	25,1	0,0	0,0	0,0
YPK_2380	fliA	flagellar biosynthesis sigma factor	0,85	260,5	246,9	253,7	0,5	0,0	0,2
YPK_0383		hypothetical protein	0,85	14,1	14,2	14,1	0,0	0,9	0,4
YPK_2379		hypothetical protein	0,85	38,2	39,2	38,7	2,2	0,0	1,1
YPK_2781		hypothetical protein	0,85	305,8	290,1	298,0	0,0	2,2	1,1
YPK_2401		flagellar biosynthesis protein FliO	0,85	41,4	37,9	39,6	0,0	0,0	0,0
YPK_1119		glutaredoxin-like protein NrdH	-0,85	0,0	0,0	0,0	24,1	22,0	23,1
YPK_3257	ribH	6,7-dimethyl-8-ribityllumazine synthase	-0,85	116,3	106,4	111,3	201,6	201,0	201,3
YPK_1055	xni	exonuclease IX	-0,86	21,8	22,0	21,9	39,6	39,7	39,7
YPK_4090		acetylornithine deacetylase	-0,89	15,1	19,5	17,3	29,1	35,2	32,1
YPK_2233		calcium/sodium:proton antiporter	-0,92	25,6	22,3	23,9	45,7	44,9	45,3
YPK_3633	prfC	peptide chain release factor 3	-0,93	44,5	49,6	47,0	84,0	94,8	89,4
YPK_3972	livM	leucine/isoleucine/valine transporter perme	-0,93	2,8	3,6	3,2	5,7	6,5	6,1
YPK_3474		aspartate alpha-decarboxylase	-0,93	104,6	97,8	101,2	195,8	189,6	192,7
YPK_1169		LamB/YcsF family protein	-0,94	9,6	9,9	9,7	18,8	18,3	18,6
YPK_1779		hypothetical protein	-0,94	18,9	18,4	18,6	35,4	35,9	35,7
YPK_1723		isocitrate dehydrogenase	-0,95	941,1	888,5	914,8	1788,0	1738,5	1763,3
YPK_1903	tpx	thiol peroxidase	-0,95	864,4	832,3	848,4	1704,0	1572,3	1638,1
YPK_1198		putative DNA-binding protein (Roi)	-0,95	2,2	3,0	2,6	4,5	5,4	5,0
YPK_4034	rho	transcription termination factor Rho	-0,95	112,5	120,1	116,3	216,1	234,0	225,1
YPK_1379		radical SAM domain-containing protein	-0,98	18,6	22,7	20,7	40,0	41,6	40,8
YPK_0726		flagellin domain-containing protein	-0,99	6,3	6,3	6,3	12,8	12,2	12,5
YPK_2095		methionine sulfoxide reductase B	-1,01	177,5	179,4	178,4	354,4	363,0	358,7
YPK_0459		acetyl-CoA carboxylase biotin carboxyl carrierpro	-1,04	26,6	30,5	28,5	54,7	62,0	58,4
YPK_2511		putative thiol-disulfide oxidoreductase DCC	-1,05	8,4	10,9	9,7	18,8	21,2	20,0
YPK_1066	rpsB	30S ribosomal protein S2	-1,13	325,9	289,9	307,9	666,7	680,0	673,3
YPK_1184	rseB	periplasmic negative regulator of sigmaE	-1,13	151,8	137,5	144,7	315,4	317,0	316,2
YPK_2770		xylulokinase	-1,13	1,7	1,8	1,7	3,9	3,8	3,8
YPK_3760		putative transcriptional regulator Nlp	-1,14	9,0	6,8	7,9	18,3	16,4	17,4
YPK_0970		binding-protein-dependent transport system inne	-1,14	2,9	3,6	3,2	6,9	7,4	7,1
YPK_3153		peptidyl-prolyl cis-trans isomerase B	-1,17	331,4	315,1	323,2	747,0	707,8	727,4
YPK_2788		binding-protein-dependent transport system inne	-1,18	29,8	29,9	29,8	65,7	69,4	67,5
YPK_3785		hypothetical protein	-1,19	13,1	10,1	11,6	28,7	24,0	26,4
YPK_3573	ksgA	dimethyladenosine transferase	-1,25	32,3	25,2	28,7	69,5	66,8	68,1
YPK_1693		putative metallodependent hydrolase	-1,25	10,0	18,1	14,1	29,5	36,2	32,8

YPK_0785	putative siderophore biosynthesis protein IucB	-1,26	1,7	2,6	2,2	4,9	5,5	5,2
YPK_0169	feoA ferrous iron transport protein A	-1,27	16,0	9,9	13,0	34,1	27,6	30,9
YPK_3122	Mu tail sheath family protein	-1,28	1,6	2,7	2,1	4,6	5,6	5,1
YPK_3763	hypothetical protein	-1,33	34,9	35,5	35,2	89,6	87,0	88,3
YPK_2252	iron permease FTR1	-1,37	48,4	43,8	46,1	117,6	120,8	119,2
YPK_1773	spore coat U domain-containing protein	-1,38	3,2	2,4	2,8	7,8	6,6	7,2
YPK_2778	mannonate dehydratase	-1,38	53,3	57,5	55,4	141,1	147,7	144,4
YPK_0781	sugar (glycoside-Pentoside-hexuronide)transport	-1,40	4,3	4,3	4,3	11,2	11,5	11,4
YPK_3994	putative sugar phosphatase	-1,41	33,7	41,2	37,5	91,6	107,6	99,6
YPK_3764	hypothetical protein	-1,42	21,6	14,1	17,8	52,5	41,9	47,2
YPK_3117	DNA circulation family protein	-1,42	2,4	1,1	1,7	5,0	4,0	4,5
YPK_1275	DNA-binding transcriptional regulator IscR	-1,43	43,4	47,5	45,4	119,5	125,9	122,7
YPK_2948	gpmA phosphoglyceromutase	-1,48	308,8	299,7	304,2	889,7	809,7	849,7
YPK_1894	phage shock protein PspA	-1,53	165,0	162,9	164,0	488,1	457,1	472,6
YPK_0780	hypothetical protein	-1,55	7,2	9,6	8,4	23,8	25,1	24,5
YPK_2787	binding-protein-dependent transport system inne	-1,61	32,2	33,2	32,7	95,3	104,8	100,0
YPK_2278	binding-protein-dependent transport system inne	-1,62	0,7	1,0	0,9	2,6	2,7	2,6
pYV0009	hypothetical protein	-1,64	29,2	26,3	27,7	85,3	87,6	86,5
YPK_2653	hypothetical protein	-1,64	66,9	74,1	70,5	214,8	224,7	219,7
YPK_2556	putative sugar ABC transporter	-1,68	3,8	3,3	3,6	11,1	11,7	11,4
YPK_2786	extracellular solute-binding protein	-1,70	34,6	34,4	34,5	106,5	117,9	112,2
YPK_3149	integrase family protein	-1,72	23,4	25,3	24,4	83,0	78,1	80,5
YPK_0967	ABC transporter-like protein	-1,75	12,5	14,1	13,3	41,6	47,9	44,7
YPK_2768	sugar ABC transporter substrate-binding protein	-1,76	2,4	1,9	2,1	7,2	7,2	7,2
YPK_0825	virulence determinant	-1,76	2,0	2,2	2,1	7,2	7,3	7,2
YPK_3823	groES co-chaperonin GroES	-1,81	728,0	639,6	683,8	2316,8	2488,6	2402,7
YPK_1847	sufA iron-sulfur cluster assembly scaffold protei	-1,86	23,5	23,3	23,4	85,6	84,0	84,8
YPK_2361	D-alanine/D-serine/glycine permease	-1,92	11,7	14,3	13,0	50,0	48,5	49,2
YPK_1858	cyclopropane-fatty-acyl-phospholipid synthase	-1,96	28,8	28,5	28,6	105,7	117,6	111,6
YPK_3322	putative aminotransferase	-1,97	1,2	0,5	0,8	3,4	2,9	3,1
YPK_1849	sufC cysteine desulfurase ATPase	-1,98	15,9	18,2	17,0	65,4	68,7	67,0
YPK_1268	virulence-related outer membrane protein	-1,99	8092,9	7611,2	7852,0	30663,9	31542,9	31103,4
YPK_1349	N-acetyltransferase GCN5	-2,00	91,3	80,8	86,1	368,0	320,4	344,2
YPK_1958	ABC transporter periplasmic substrate-bindingpro	-2,07	2,0	1,7	1,9	8,5	7,4	7,9
YPK_2831	outer membrane protease	-2,14	2,4	3,2	2,8	11,3	13,5	12,4
YPK_2487	ABC transporter-like protein	-2,18	10,3	8,6	9,4	43,2	42,0	42,6
pYV0051	hypothetical protein	-2,22	10,0	2,1	6,0	29,7	22,5	26,1
pYV0034	transposase remnant	-2,28	4,4	8,1	6,3	28,2	32,1	30,1
YPK_2885	pbpG D-alanyl-D-alanine endopeptidase	-2,30	9,9	11,8	10,8	51,5	54,7	53,1
pYV0035	hypothetical protein	-2,50	18,5	12,2	15,3	85,1	88,9	87,0
YPK_3008	transporter-associated protein	-2,53	18,5	19,6	19,0	114,8	105,8	110,3
YPK_2769	ribose 5-phosphate isomerase	-2,56	1,1	2,1	1,6	8,5	9,9	9,2
YPK_3923	actP acetate permease	-2,60	62,2	58,6	60,4	363,2	370,5	366,9
pYV0044	hypothetical protein	-2,66	18,0	14,2	16,1	108,8	94,7	101,8
pYV0023	transposase remnant	-2,71	13,9	14,9	14,4	94,8	93,1	93,9
YPK_1972	hypothetical protein	-2,72	10,7	9,1	9,9	68,3	62,3	65,3
YPK_2547	pyridoxal-dependent decarboxylase	-2,77	1,5	1,5	1,5	10,5	9,9	10,2
pYV0036	hypothetical protein	-2,81	9,5	6,2	7,8	54,6	55,4	55,0
YPK_2055	transporter	-2,85	13,4	15,3	14,4	101,7	105,3	103,5
YPK_3893	hmuV hemin importer ATP-binding subunit	-2,89	8,1	7,2	7,6	58,8	54,4	56,6
YPK_1407	putative sialic acid transporter	-2,91	7,2	6,9	7,0	50,0	56,2	53,1
YPK_2615	cytotoxic necrotizing factor	-3,04	440,2	417,1	428,6	3761,2	3320,9	3541,1
pYV0021	transposase	-3,11	2,1	6,3	4,2	30,6	38,2	34,4
YPK_0495	PTS system trehalose(maltose)-specifictransport	-3,18	18,5	17,9	18,2	157,4	174,2	165,8
pYV0073	yscT, type III secretion protein	-3,33	54,6	54,5	54,6	581,0	521,4	551,2
YPK_0964	periplasmic binding protein/LacI transcriptionalre	-3,37	11,0	10,0	10,5	108,1	108,5	108,3
pYV0013	hypothetical protein	-3,54	20,7	24,5	22,6	268,2	258,0	263,1
pYV0089	yscM, lcrQ, type III secretion regulatory	-3,66	141,4	126,3	133,8	1730,2	1654,4	1692,3
pYV0091	transposase	-3,68	25,3	30,5	27,9	372,9	347,3	360,1
pYV0064	tyeA, Yop secretion and targeting protein	-3,69	444,4	475,5	460,0	5622,7	6267,6	5945,1
YPK_0599	class I and II aminotransferase	-3,72	7,5	6,8	7,1	94,0	94,1	94,0
YPK_3888	hemin uptake protein	-3,73	5,3	6,2	5,7	81,4	72,5	76,9
YPK_2366	iron permease FTR1	-3,95	3,5	2,8	3,1	47,3	50,0	48,7
YPK_0280	bacterioferritin-associated ferredoxin	-4,00	71,9	84,1	78,0	1263,3	1237,1	1250,2
pYV0037		-4,07	12,3	18,5	15,4	235,7	278,2	257,0
YPK_2365	hypothetical protein	-4,12	11,3	10,5	10,9	183,1	195,8	189,5
YPK_0966	ABC transporter-like protein	-4,15	2,8	3,2	3,0	55,3	51,5	53,4
pYV0072	yscS, type III secretion protein	-4,19	80,7	71,3	76,0	1407,6	1373,7	1390,6
pYV0002	hypothetical protein	-4,24	165,6	175,1	170,3	3148,6	3271,3	3209,9
YPK_3885	coproporphyrinogen III oxidase	-4,31	5,3	3,7	4,5	87,3	91,9	89,6

pYV0024	sycE, yerA, yopE chaperone	-4,40	142,9	154,6	148,8	3308,9	3019,4	3164,1
pYV0084	yscH, yopR, lcrP, type III secretion protein	-4,45	322,0	312,1	317,0	7156,8	6732,9	6944,8
YPK_2839	outer membrane porin protein C	-4,45	164,7	164,8	164,7	3767,2	3461,9	3614,5
pYV0087	yscK, type III secretion protein	-4,51	29,7	26,7	28,2	608,4	682,4	645,4
pYV0085	yscI, lcrO, type III secretion protein	-4,60	274,3	240,9	257,6	6302,0	6226,3	6264,2
pYV0093	transposase	-4,65	1,6	5,1	3,4	75,1	87,9	81,5
pYV0077	hypothetical protein	-4,67	48,8	39,6	44,2	1171,4	1066,1	1118,7
pYV0061	yscY, type III secretion protein	-4,67	23,1	23,5	23,3	554,5	636,7	595,6
pYV0060	lcrD, yscV, membrane-bound Yop protein	-4,67	73,3	74,1	73,7	1905,4	1856,3	1880,9
pYV0069	yscP, type III secretion protein	-4,68	81,4	78,6	80,0	2020,8	2076,9	2048,9
pYV0070	type III secretion system protein	-4,83	59,1	65,8	62,4	1756,6	1783,2	1769,9
pYV0074	yscU, type III secretion protein	-4,91	38,6	37,6	38,1	1189,9	1101,2	1145,5
pYV0065	yopN, lcrE, membrane-bound Yop targetingprotei	-4,93	298,2	286,9	292,6	8711,1	9093,8	8902,4
pYV0086	yscJ, ylpB, type III secretion lipoprotein	-4,95	128,9	130,9	129,9	4052,7	3978,7	4015,7
YPK_2203	type 12 methyltransferase	-5,05	9,8	8,7	9,2	310,2	300,6	305,4
YPK_2291	transposase	-5,15	7,4	2,8	5,1	184,2	173,7	179,0
pYV0075	virG, Yop targeting lipoprotein	-5,16	4,1	3,8	3,9	143,2	139,2	141,2
pYV0071	type III secretion system protein	-5,21	34,1	27,2	30,7	1140,9	1122,9	1131,9
pYV0067	type III secretion system ATPase	-5,21	73,3	65,3	69,3	2407,8	2777,6	2592,7
pYV0083	yscG, type III secretion protein	-5,27	245,0	246,7	245,8	9769,5	9187,4	9478,5
pYV0076	lcrF, virF, thermoregulatory protein	-5,43	9,2	9,8	9,5	435,1	388,2	411,6
pYV0079	yscC, type III secretion protein	-5,47	80,6	70,7	75,6	3455,9	3246,1	3351,0
pYV0068	yscO, type III secretion protein	-5,49	74,6	60,6	67,6	3075,4	3013,8	3044,6
pYV0092	transposase	-5,62	9,6	6,1	7,8	394,9	373,2	384,0
pYV0053	hypothetical protein	-5,74	39,3	46,1	42,7	2298,0	2254,8	2276,4
pYV0039	transposase	-5,79	36,2	30,3	33,2	1972,5	1701,2	1836,8
YPK_3389	cytochrome b562	-5,86	63,3	69,9	66,6	3940,9	3787,3	3864,1
pYV0080	yscD, type III secretion protein	-5,90	41,8	40,3	41,0	2525,4	2379,9	2452,7
pYV0081	yscE, type III secretion protein	-5,91	72,8	81,1	76,9	4812,6	4455,5	4634,1
YPK_2198	hypothetical protein	-5,98	17,9	16,4	17,1	1144,6	1023,0	1083,8
YPK_2199	hypothetical protein	-5,99	35,1	34,2	34,6	2374,0	2057,7	2215,9
pYV0082	yscF, type III secretion protein	-6,07	156,4	167,0	161,7	11548,6	10305,6	10927,1
YPK_2200	hypothetical protein	-6,12	515,2	584,3	549,8	40077,0	36900,9	38488,9
pYV0059	hypothetical protein	-6,13	20,9	18,5	19,7	1434,7	1325,7	1380,2
YPK_2197	hypothetical protein	-6,17	17,9	16,2	17,1	1316,2	1140,9	1228,5
pYV0078	hypothetical protein	-6,33	19,4	39,0	29,2	2587,0	2253,6	2420,3
YPK_3390	cytochrome b561	-6,43	9,0	4,6	6,8	581,8	599,9	590,9
pYV0098	yopP, yopJ, targeted effector protein	-6,77	28,8	25,2	27,0	3119,5	2775,6	2947,5
pYV0094	yopH, protein-tyrosine phosphatase Yop effector	-7,06	598,2	559,3	578,8	80035,1	74485,3	77260,2
pYV0058	lcrG, Yop regulator	-7,15	49,5	51,8	50,6	7490,3	6955,8	7223,0
pYV0054	yopD, Yop negative regulation/targetingcomponer	-7,24	726,2	706,0	716,1	113245,6	103968,2	108606,9
pYV0088	type III secretion system protein	-7,59	10,9	5,2	8,0	1557,6	1524,3	1540,9
pYV0057	lcrV, V antigen, antihost protein/regulator	-8,30	39,7	28,8	34,2	11318,9	10190,1	10754,5
pYV0040	yop targeting protein yopK, yopQ	-8,41	9,2	12,2	10,7	3990,1	3415,9	3703,0
pYV0055	yopB, Yop targeting protein	-9,05	96,4	85,8	91,1	48408,6	48450,4	48429,5
pYV0056	lcrH, sycD, low calcium response protein H	-9,49	39,1	28,2	33,7	23815,2	24802,3	24308,7
pYV0047	yopM, targeted effector protein	-9,55	4,4	4,3	4,4	3512,3	3070,3	3291,3
