# Supplementary material for: Reprogramming of Yersinia from Virulent to Persistent Mode Revealed by Complex In Vivo RNA-seq Analysis
Source: PLoS Pathog. 2015 Jan 15;11(1):e1004600. doi: 10.1371/journal.ppat.1004600 (PMC4295882; doi:10.1371/journal.ppat.1004600)
Supplement: S3 Table — The analyses were performed with CLC-Bio Genomics Workbench by using Transcriptomics Analysis Module. The data were obtained from two biological replicates from cecal tissue of two FVBn mice at early phase of infection and from cecal tissue of two FVBn mice at persistent infection. The expression values are given as RPKM and differentially expressed genes were filtered with Log2 fold change ≥0,7. All data generated in this study were deposited in the Gene Expression Omnibus (GEO) database and are available under accession number GSE56477. (PDF) [file ppat.1004600.s010.pdf]

Table S3. ***In vivo*** **(Persistent infection vs Early infection) differentially expressed genes of** ***Yersinia pseudotuberculosis YPIII.*** The analyses were performed with CLC-Bio Genomics Workbench by using Transcriptomics Analysis Module. The data were obtained from two biological replicates from cecal tissue of two FVBn mice at early phase of infection and from cecal tissue of two FVBn mice at persistent infection. The expression values are given as RPKM and differentially expressed genes were filtered with Log2 fold change ≥0,7. All data generated in this study were deposited in the Gene Expression Omnibus (GEO) database and are available under accession number GSE56477.

Locus Tag	Definition	Log2 FC	Early #1 RPKMO	Early #2 RPKMO	Early Means	Persistence #1 RPKMO	Persistence #2 RPKMO	Persistence means
YPK_0304	rpmJ 50S ribosomal protein L36	13,35	0,2	0,3	0,2	3288,8	1963,2	2626,0
YPK_0120	UspA domain-containing protein	12,54	0,8	1,0	0,9	3046,6	9002,1	6024,3
YPK_0631	hypothetical protein	12,54	0,6	0,7	0,6	5673,9	2344,8	4009,3
YPK_2475	hypothetical protein	12,06	0,2	0,3	0,2	1096,3	981,6	1039,0
YPK_0504	putative sigma(54) modulation protein	11,79	0,5	0,6	0,6	1401,6	2928,4	2165,0
YPK_3799	hfq RNA-binding protein Hfq	11,62	0,6	0,7	0,6	3084,2	1183,5	2133,8
YPK_2961	hypothetical protein	11,59	0,6	0,7	0,7	1623,6	2544,2	2083,9
YPK_4198	hypothetical protein	11,06	0,5	0,6	0,5	994,5	1335,7	1165,1
YPK_3361	rplS 50S ribosomal protein L19	10,96	0,6	0,7	0,7	777,8	2437,6	1607,7
YPK_1740	cold-shock DNA-binding domain-containingprotein	10,93	0,4	0,5	0,4	1248,7	559,0	903,9
YPK_2389	hypothetical protein	10,89	0,3	0,4	0,4	732,8	656,1	694,5
YPK_1087	hypothetical protein	10,59	0,4	0,4	0,4	659,8	590,8	625,3
YPK_0132	hypothetical protein	10,43	0,4	0,5	0,4	624,3	559,0	591,7
YPK_1602	DNA starvation/stationary phase protectionprotein D	10,42	0,9	1,1	1,0	1083,5	1697,8	1390,6
YPK_2733	grxA glutaredoxin	10,33	0,5	0,6	0,5	508,1	910,0	709,0
YPK_4035	thioredoxin	10,23	0,6	0,7	0,7	413,1	1479,7	946,4
YPK_2881	hypothetical protein	10,11	0,6	0,7	0,7	791,1	708,3	749,7
YPK_2390	fliE flagellar hook-basal body protein FliE	9,87	0,6	0,7	0,6	864,8	387,2	626,0
YPK_2438	ferroxidase	9,67	0,9	401,6	201,3	21151,9	11747,3	16449,6
YPK_1876	transcriptional regulator SlyA	9,45	0,8	0,9	0,9	1260,0	282,0	771,0
YPK_0605	hypothetical protein	9,45	0,6	0,7	0,6	444,8	398,3	421,6
YPK_3729	rbfA ribosome-binding factor A	9,38	0,8	0,9	0,8	330,7	888,4	609,5
YPK_4171	hypothetical protein	9,37	0,7	0,8	0,7	364,5	652,7	508,6
YPK_2049	outer membrane protein W	9,35	1,2	1,3	1,3	431,0	1543,8	987,4
YPK_4013	cyaY frataxin-like protein	9,29	0,6	0,7	0,6	420,6	376,6	398,6
YPK_0652	hypothetical protein	9,22	1,2	1,5	1,3	1198,5	536,6	867,5
YPK_2381	flagellin	9,22	2,0	2,3	2,2	1491,7	1113,1	1302,4
YPK_3244	cytochrome o ubiquinol oxidase subunit IV	9,18	0,6	0,7	0,7	405,9	363,5	384,7
YPK_0300	rpsE 30S ribosomal protein S5	9,18	0,9	1,1	1,0	270,9	1212,7	741,8
YPK_0528	hypothetical protein	9,12	0,9	1,0	1,0	1122,8	251,3	687,1
YPK_3937	rfaH transcriptional activator RfaH	9,12	0,9	1,0	1,0	280,7	1005,4	643,0
YPK_4216	flavodoxin	8,89	0,8	0,9	0,9	308,7	552,8	430,8
YPK_1277	scaffold protein	8,76	0,7	0,8	0,8	350,7	314,0	332,4
YPK_0012	heat shock chaperone IbpB	8,74	0,8	1,0	0,9	293,1	524,9	409,0
YPK_1069	frr ribosome recycling factor	8,73	1,0	1,2	1,1	490,2	439,0	464,6
YPK_2310	XRE family transcriptional regulator	8,72	0,7	0,8	0,8	345,5	309,3	327,4
YPK_0037	molybdopterin oxidoreductase Fe4S4 region	8,58	1,1	1,2	1,2	465,6	416,9	441,3
YPK_2980	smpB SsrA-binding protein	8,57	0,9	1,1	1,0	275,7	493,7	384,7
YPK_1750	purine-binding chemotaxis protein	8,55	0,9	1,1	1,0	274,1	490,8	382,4
YPK_0248	cAMP-regulatory protein	8,53	1,1	1,3	1,2	216,5	969,4	593,0
YPK_3714	sterol-binding domain-containing protein	8,51	1,1	1,3	1,2	454,3	406,7	430,5
YPK_3512	lpxC UDP-3-O-[3-hydroxymyristoyl] N-acetylgluco:	8,45	1,7	1,9	1,8	596,3	667,4	631,8
YPK_3238	hypothetical protein	8,41	1,1	1,2	1,1	236,4	635,0	435,7
YPK_1295	hypothetical protein	8,35	1,2	1,3	1,3	431,0	386,0	408,5
YPK_3781	rplI 50S ribosomal protein L9	8,32	0,8	1,0	0,9	300,7	269,3	285,0
YPK_2969	sdhB succinate dehydrogenase iron-sulfur subunit	8,31	1,3	1,5	1,4	574,5	343,0	458,7

YPK_1172	pyrrolidone-carboxylate peptidase	8,30	1,2	1,4	1,3	423,2	378,9	401,1
YPK_0334	nusG transcription antitermination protein NusG	8,29	1,0	1,2	1,1	500,8	224,2	362,5
YPK_2380	fliA flagellar biosynthesis sigma factor	8,28	1,3	1,5	1,4	569,8	340,2	455,0
YPK_1071	undecaprenyl pyrophosphate synthase	8,14	1,4	1,6	1,5	543,2	324,2	433,7
YPK_4187	phosphatase	8,06	1,1	1,3	1,2	231,7	414,9	323,3
YPK_3397	DeoR family transcriptional regulator	7,96	1,5	1,7	1,6	509,4	304,1	406,7
YPK_0773	hypothetical protein	7,95	0,9	1,1	1,0	264,7	237,0	250,8
YPK_3775	adenosine-3'(2'),5'-bisphosphate nucleotidase	7,92	1,3	1,6	1,5	185,4	664,0	424,7
YPK_2175	2-dehydro-3-deoxyphosphooctonate aldolase	7,80	1,5	1,8	1,7	161,0	864,7	512,8
YPK_0322	hexapaptide repeat-containing transferase	7,80	1,0	1,2	1,1	251,8	225,4	238,6
YPK_3814	fumarate reductase iron-sulfur subunit	7,74	1,3	1,6	1,4	560,6	167,3	364,0
YPK_0381	lamB maltoporin	7,67	2,3	2,7	2,5	325,8	777,9	551,9
YPK_0163	putative DNA uptake protein	7,64	1,0	1,2	1,1	237,6	212,7	225,2
YPK_4113	glycerol kinase	7,60	2,7	3,2	3,0	453,8	731,4	592,6
YPK_2954	tol-pal system protein YbgF	7,46	1,5	1,7	1,6	169,8	456,1	312,9
YPK_1529	truA tRNA pseudouridine synthase A	7,42	1,6	1,9	1,7	312,2	279,5	295,8
YPK_0642	putative signal transduction protein	7,42	1,1	1,3	1,2	220,6	197,6	209,1
YPK_0057	xylF D-xylose transporter subunit XylF	7,37	1,8	2,1	1,9	276,8	371,8	324,3
YPK_1104	hypothetical protein	7,34	1,4	1,6	1,5	180,3	323,0	251,7
YPK_2761	transcriptional regulator CadC	7,31	1,2	1,4	1,3	212,6	190,3	201,4
YPK_1682	rpmF 50S ribosomal protein L32	7,26	0,3	1173,3	586,8	3128,7	2801,5	2965,1
YPK_1609	hypothetical protein	7,24	1,2	1,4	1,3	206,9	185,2	196,1
YPK_2568	folE GTP cyclohydrolase I	7,24	1,2	1,4	1,3	206,9	185,2	196,1
YPK_1036	thyA thymidylate synthase	7,22	1,4	1,7	1,6	173,0	309,7	241,3
YPK_3187	glycosyl transferase family protein	7,17	1,7	2,0	1,9	286,2	256,2	271,2
YPK_3773	hypothetical protein	7,13	1607,6	0,5	804,1	3746,0	3913,3	3829,7
YPK_3735	folP dihydropteroate synthase	7,08	1,5	1,8	1,6	329,9	147,7	238,8
YPK_0158	intramembrane serine protease GlpG	7,07	1,5	1,8	1,6	328,8	147,2	238,0
YPK_4112	MIP family channel protein	7,03	1,5	1,8	1,7	162,1	290,3	226,2
YPK_0378	malE maltose ABC transporter periplasmic protein	7,01	2,2	2,5	2,4	455,7	204,0	329,9
YPK_1681	hypothetical protein	6,96	1,0	390,4	195,7	3122,9	1864,2	2493,5
YPK_3590	rpsT 30S ribosomal protein S20	6,95	0,5	1524,5	762,5	3556,9	3184,9	3370,9
YPK_1811	fructosamine kinase	6,94	1,6	1,8	1,7	314,3	140,7	227,5
YPK_3779	opacity-associated protein A	6,92	1,3	1,6	1,5	185,4	166,0	175,7
YPK_2185	hypothetical protein	6,90	0,5	1476,0	738,3	3443,8	3083,6	3263,7
YPK_1190	era GTP-binding protein Era	6,83	1,6	1,9	1,8	151,0	270,4	210,7
YPK_2649	porin	6,80	328,0	191,1	259,5	31081,0	25206,9	28144,0
YPK_3300	Na(+)-translocating NADH-quinone reductasesubur	6,78	2,2	2,6	2,4	338,5	202,1	270,3
YPK_3264	tgt queuine tRNA-ribosyltransferase	6,73	2,0	2,4	2,2	245,3	219,7	232,5
YPK_3281	hypothetical protein	6,71	0,5	1401,7	701,1	2803,2	2928,4	2865,8
YPK_0001	dnaA chromosomal replication initiation protein	6,70	2,4	2,8	2,6	408,6	182,9	295,8
YPK_3526	mraW S-adenosyl-methyltransferase MraW	6,67	1,7	2,0	1,9	143,1	256,2	199,7
YPK_2187	GTP-dependent nucleic acid-binding protein EngD	6,60	2,0	2,3	2,1	379,0	113,1	246,1
YPK_1431	cell division protein ZipA	6,60	1,8	2,1	1,9	279,3	125,0	202,2
YPK_3826	anaerobic C4-dicarboxylate transporter	6,60	2,3	2,7	2,5	318,4	190,0	254,2
YPK_0667	dkgA 2,5-diketo-D-gluconate reductase A	6,58	1,5	1,8	1,6	165,0	147,7	156,3
YPK_0854	mechanosensitive ion channel MscS	6,46	1,6	1,8	1,7	158,2	141,7	149,9
YPK_1507	hypothetical protein	6,43	583,9	0,6	292,3	1360,5	2030,4	1695,5
YPK_1141	voltage-gated potassium channel	6,39	2,1	2,5	2,3	352,2	105,1	228,6
YPK_1822	rpmI 50S ribosomal protein L35	6,33	1723,6	0,4	862,0	2677,5	1798,1	2237,8
YPK_1532	bifunctional folylpolyglutamate synthase/dihydrofola	6,30	2,4	2,7	2,5	211,8	189,6	200,7
YPK_2441	hypothetical protein	6,29	0,2	0,3	0,2	0,2	2010,7	1005,5
YPK_1276	cysteine desulfurase	6,26	2,2	2,6	2,4	336,8	100,5	218,7
YPK_4153	rpmG 50S ribosomal protein L33	6,26	0,3	1173,3	586,8	1564,4	1400,7	1482,5
YPK_3772	hypothetical protein	6,24	2,4	2,8	2,6	207,5	185,8	196,6
YPK_4221	F0F1 ATP synthase subunit C	6,22	1434,0	0,5	717,3	1670,7	2493,3	2082,0
YPK_1056	hypothetical protein	6,17	2,5	2,9	2,7	101,3	362,7	232,0
YPK_1535	amidophosphoribosyltransferase	6,16	2,7	3,2	3,0	182,2	244,8	213,5
YPK_2508	mandelate racemase/muconate lactonizing protein	6,15	2,3	2,7	2,5	324,3	96,8	210,5

YPK_3263	yajC preprotein translocase subunit YajC	6,09	518,1	0,7	259,4	1207,2	1441,2	1324,2
YPK_3993	hypothetical protein	6,04	2,2	2,5	2,3	230,1	103,0	166,6
YPK_3767	inorganic pyrophosphatase	5,96	1,0	776,4	388,7	1552,7	1853,8	1703,2
YPK_3178	phosphomannomutase	5,95	2,5	2,9	2,7	100,6	270,2	185,4
YPK_4051	ketol-acid reductoisomerase	5,94	2,7	3,1	2,9	187,0	167,4	177,2
YPK_0465	rod shape-determining protein MreB	5,94	1,9	2,2	2,0	132,1	118,3	125,2
YPK_3010	glutamate and aspartate transporter subunit	5,89	579,5	1,9	290,7	2250,4	1746,4	1998,4
YPK_0546	hypothetical protein	5,86	0,7	514,4	257,6	1028,8	1228,2	1128,5
YPK_3240	hypothetical protein	5,79	0,2	0,3	0,2	1122,8	0,2	561,5
YPK_1826	ihfA integration host factor subunit alpha	5,77	0,5	680,3	340,4	2721,1	406,1	1563,6
YPK_2405	hypothetical protein	5,76	0,3	0,4	0,3	0,2	1400,7	700,5
YPK_2963	cytochrome d ubiquinol oxidase subunit II	5,74	2,1	363,2	182,6	1089,6	1951,3	1520,4
YPK_3369	glutamate--cysteine ligase	5,72	2,9	3,4	3,1	173,1	155,0	164,0
YPK_1863	superoxide dismutase	5,69	304,3	1,2	152,8	1181,9	846,6	1014,3
YPK_2691	infA translation initiation factor IF-1	5,69	0,4	0,5	0,4	1823,9	0,3	912,1
YPK_0292	rpsQ 30S ribosomal protein S17	5,69	0,5	0,6	0,5	0,4	1881,7	941,1
YPK_1601	hypothetical protein	5,67	2,0	183,5	92,8	1101,1	876,4	988,7
YPK_3943	ubiB putative ubiquinone biosynthesis protein UbiE	5,66	2,9	3,4	3,2	169,6	151,8	160,7
YPK_1725	hypothetical protein	5,65	0,5	0,6	0,5	2055,0	0,3	1027,7
YPK_3404	tartrate/fumarate subfamily Fe-S typehydro-lyase su	5,64	3,0	3,4	3,2	84,2	301,5	192,8
YPK_3372	carbon storage regulator	5,62	0,3	0,4	0,4	0,3	1272,1	636,2
YPK_0305	rpsM 30S ribosomal protein S13	5,62	0,7	1138,2	569,4	758,7	2377,8	1568,3
YPK_3507	hypothetical protein	5,60	0,4	0,4	0,4	0,3	1252,9	626,6
YPK_4170	hypothetical protein	5,60	0,4	0,4	0,4	0,3	1252,9	626,6
YPK_3231	transcriptional regulator HU subunit beta	5,59	633,4	0,6	317,0	983,9	881,0	932,5
YPK_1593	O-succinylbenzoic acid--CoA ligase	5,57	2,6	3,0	2,8	195,3	87,4	141,3
YPK_3035	hypothetical protein	5,56	0,4	0,4	0,4	0,3	1216,2	608,3
YPK_3230	peptidyl-prolyl cis-trans isomerase D	5,54	3,4	4,0	3,7	220,2	131,4	175,8
YPK_0293	rplN 50S ribosomal protein L14	5,53	1407,7	0,8	704,3	728,9	3263,5	1996,2
YPK_4249	rpmH 50S ribosomal protein L34	5,50	0,3	0,3	0,3	0,2	825,6	412,9
YPK_3295	aminoacyl-histidine dipeptidase	5,48	2,6	3,1	2,8	189,3	84,7	137,0
YPK_3756	rpmA 50S ribosomal protein L27	5,46	0,5	0,6	0,5	1558,5	0,3	779,4
YPK_0276	30S ribosomal protein S7	5,41	745,3	1,0	373,2	578,9	2332,6	1455,8
YPK_3838	IS1 transposase	5,37	0,5	0,6	0,6	1460,4	0,4	730,4
YPK_2534	hypothetical protein	5,36	0,3	0,3	0,3	838,7	0,2	419,5
YPK_3203	hypothetical protein	5,36	0,3	0,3	0,3	0,2	751,0	375,6
YPK_0287	rpsS 30S ribosomal protein S19	5,35	0,5	0,6	0,6	0,4	1294,1	647,3
YPK_0146	signal transduction histidine kinase LytS	5,34	3,1	3,6	3,3	81,5	219,0	150,2
YPK_1886	tppB putative tripeptide transporter permease	5,34	2,8	3,2	3,0	180,1	80,6	130,4
YPK_2933	6-phosphogluconolactonase	5,33	176,6	2,1	89,4	823,0	736,9	780,0
YPK_4111	hypothetical protein	5,27	717,0	0,5	358,8	1113,8	498,7	806,2
YPK_1717	hypothetical protein	5,26	0,3	0,4	0,3	782,2	0,2	391,2
YPK_3932	proline dipeptidase	5,24	2,4	2,8	2,6	103,7	92,9	98,3
YPK_1745	transcriptional activator FlhD	5,24	0,6	0,8	0,7	1542,7	0,4	771,6
YPK_1767	hypothetical protein	5,22	0,5	0,5	0,5	0,4	962,7	481,5
YPK_3061	hypothetical protein	5,20	0,5	0,5	0,5	1062,8	0,3	531,6
YPK_1673	bssS biofilm formation regulatory protein BssS	5,19	0,5	788,1	394,3	525,4	940,9	733,1
YPK_2048	transport-associated	5,18	0,6	0,7	0,6	1285,2	0,4	642,8
YPK_2621	heat shock protein HspQ	5,17	0,6	0,7	0,6	1273,4	0,4	636,9
YPK_3028	tatE twin arginine translocase protein A	5,17	0,5	0,6	0,5	0,4	930,3	465,4
YPK_2425	flgB flagellar basal body rod protein FlgB	5,17	0,8	0,9	0,8	1641,8	0,5	821,2
YPK_1183	anti-RNA polymerase sigma factor SigE	5,15	537,5	1,4	269,4	626,2	1495,2	1060,7
YPK_2059	hypothetical protein	5,14	0,5	0,6	0,5	0,4	910,0	455,2
YPK_4227	atpC F0F1 ATP synthase subunit epsilon	5,14	0,8	0,9	0,8	0,6	1439,6	720,1
YPK_3837	hypothetical protein	5,13	822,3	0,9	411,6	638,7	1429,7	1034,2
YPK_3474	aspartate alpha-decarboxylase	5,12	0,7	0,8	0,8	0,5	1275,4	637,9
YPK_3509	preprotein translocase subunit SecA	5,10	4,9	5,7	5,3	102,2	320,2	211,2
YPK_2977	hypothetical protein	5,07	0,6	0,7	0,7	0,5	1062,5	531,5
YPK_1877	17 kDa surface antigen	5,04	1125,0	1,0	563,0	1165,1	1043,2	1104,2

YPK_2802	colicin D	5,03	0,5	0,6	0,6	943,9	0,4	472,1
YPK_2489	hypothetical protein	5,03	0,5	0,6	0,6	0,4	845,2	422,8
YPK_4075	hypothetical protein	5,03	428,8	0,9	214,9	666,2	596,5	631,3
YPK_2692	clpA ATP-dependent Clp protease ATP-binding sul	5,00	4,1	4,8	4,4	121,8	163,5	142,6
YPK_3738	hypothetical protein	4,99	0,5	0,6	0,6	916,0	0,4	458,2
YPK_1163	deoxyribodipyrimidine photolyase	4,97	2,6	3,1	2,9	94,5	84,6	89,5
YPK_2474	cold shock-like protein CspC	4,96	0,4	0,5	0,4	632,9	0,3	316,6
YPK_2695	macrolide transporter ATP-binding /permease	4,94	3,5	4,1	3,8	213,1	63,6	138,4
YPK_3151	hypothetical protein	4,94	0,4	0,5	0,4	624,3	0,3	312,3
YPK_3234	clpP ATP-dependent Clp protease proteolytic subu	4,93	1,1	329,4	165,3	439,2	786,5	612,9
YPK_0286	rplB 50S ribosomal protein L2	4,91	1073,4	1,7	537,6	667,0	2538,1	1602,5
YPK_3237	transcriptional regulator BolA	4,87	0,6	0,7	0,6	841,3	0,4	420,8
YPK_3782	rpsR 30S ribosomal protein S18	4,84	0,4	0,5	0,5	0,3	523,8	262,1
YPK_1140	hdeB acid-resistance protein	4,84	527,2	1842,8	1185,0	33988,0	23466,5	28727,2
YPK_4063	hypothetical protein	4,79	0,6	0,7	0,7	0,5	714,4	357,4
YPK_1492	alkylphosphonate utilization operon proteinPhnA	4,78	0,6	0,7	0,7	0,5	708,3	354,4
YPK_3686	extracellular solute-binding protein	4,77	2,8	3,3	3,1	88,2	79,0	83,6
YPK_2482	hypothetical protein	4,77	0,4	0,5	0,5	0,3	498,7	249,5
YPK_3087	hypothetical protein	4,75	0,5	0,5	0,5	0,4	492,7	246,5
YPK_3364	rpsP 30S ribosomal protein S16	4,72	0,5	0,5	0,5	537,6	0,3	268,9
YPK_1355	arsenate reductase	4,72	0,7	0,8	0,7	0,5	679,4	339,9
YPK_3852	hypothetical protein	4,70	0,7	0,8	0,7	0,5	673,9	337,2
YPK_1818	hypothetical protein	4,70	0,5	0,5	0,5	531,4	0,3	265,9
YPK_2804	hypothetical protein	4,69	0,7	0,8	0,7	746,5	0,5	373,5
YPK_1367	hypothetical protein	4,67	0,8	1,0	0,9	0,6	807,8	404,2
YPK_2448	hypothetical protein	4,67	0,5	0,6	0,5	519,5	0,3	259,9
YPK_2158	integral membrane protein MviN	4,67	2,9	3,4	3,2	84,9	76,1	80,5
YPK_3527	cell division protein MraZ	4,65	0,8	1,0	0,9	0,7	797,4	399,0
YPK_2846	DNA gyrase subunit A	4,63	4,8	5,6	5,2	51,5	322,7	187,1
YPK_3942	twin arginine-targeting protein translocase	4,62	0,5	0,6	0,5	502,6	0,3	251,5
YPK_0539	LppC family lipoprotein	4,62	3,5	4,1	3,8	140,4	62,8	101,6
YPK_3850	single-stranded DNA-binding protein	4,61	1,0	1,2	1,1	0,8	892,1	446,4
YPK_0200	hypothetical protein	4,61	0,5	0,6	0,5	497,2	0,3	248,8
YPK_3897	hypothetical protein	4,61	0,5	0,6	0,5	0,4	445,2	222,8
YPK_0492	cytochrome b562	4,60	0,7	0,8	0,8	0,6	628,0	314,3
YPK_0868	glycine cleavage system protein H	4,60	0,7	0,8	0,8	0,6	628,0	314,3
YPK_2437	copper resistance protein CopC	4,60	0,7	0,8	0,8	0,6	628,0	314,3
YPK_1062	hypothetical protein	4,59	0,7	0,8	0,8	696,1	0,5	348,3
YPK_0306	30S ribosomal protein S11	4,59	448,1	0,8	224,5	348,1	623,3	485,7
YPK_3785	hypothetical protein	4,56	0,5	0,6	0,6	481,8	0,4	241,1
YPK_2471	hypothetical protein	4,56	0,5	0,6	0,6	0,4	431,4	215,9
YPK_3821	hypothetical protein	4,56	0,7	0,9	0,8	0,6	609,6	305,1
YPK_2177	invasion gene expression up-regulator SirB	4,55	0,7	0,9	0,8	675,9	0,5	338,2
YPK_2562	malate dehydrogenase	4,55	3,1	3,6	3,3	81,5	73,0	77,3
YPK_1676	antibiotic biosynthesis monooxygenase	4,52	0,5	0,6	0,6	0,4	418,3	209,4
YPK_4146	rfaD ADP-L-glycero-D-manno-heptose-6-epimeras	4,51	1,7	221,5	111,6	295,3	661,0	478,1
YPK_0828	hypothetical protein	4,50	0,5	0,6	0,6	462,6	0,4	231,5
YPK_2294	hypothetical protein	4,50	0,5	0,6	0,6	462,6	0,4	231,5
YPK_2363	TrpR binding protein WrbA	4,48	1,1	1,3	1,2	0,8	817,5	409,2
YPK_0836	Holliday junction resolvase-like protein	4,48	0,8	0,9	0,8	643,1	0,5	321,8
YPK_2025	hypothetical protein	4,46	0,6	0,6	0,6	0,4	402,1	201,3
YPK_2958	colicin uptake protein TolR	4,46	0,8	0,9	0,8	634,3	0,5	317,4
YPK_2018	hypothetical protein	4,43	0,6	0,7	0,6	440,6	0,4	220,5
YPK_2500	hypothetical protein	4,42	1,0	1,1	1,1	0,8	680,0	340,4
YPK_1352	upp uracil phosphoribosyltransferase	4,42	1,1	1,3	1,2	0,9	782,8	391,9
YPK_3680	DNA polymerase III subunit chi	4,39	0,8	1,0	0,9	605,3	0,6	303,0
YPK_0829	hypothetical protein	4,39	1,0	369,7	185,3	739,3	220,6	480,0
YPK_3019	hypothetical protein	4,38	0,6	0,7	0,6	424,5	0,4	212,4
YPK_4096	transcriptional repressor protein MetJ	4,38	0,6	0,7	0,6	0,5	380,1	190,3

YPK_3362	trmD tRNA (guanine-N(1)-)-methyltransferase	4,34	1,3	1,6	1,5	1,0	830,0	415,5
YPK_2781	hypothetical protein	4,34	0,6	0,7	0,7	413,1	0,4	206,8
YPK_4248	rnpA ribonuclease P	4,34	0,6	0,7	0,7	0,5	369,9	185,2
YPK_0445	17 kDa surface antigen	4,33	0,9	1,0	0,9	0,7	521,6	261,1
YPK_0736	17 kDa surface antigen	4,33	0,9	1,0	0,9	0,7	521,6	261,1
YPK_3146	hypothetical protein	4,33	0,6	0,7	0,7	0,5	366,7	183,6
YPK_2264	putative lipoprotein	4,32	0,9	1,0	0,9	575,3	0,6	288,0
YPK_1490	type VI secretion system lysozyme-like protein	4,30	0,9	1,0	0,9	0,7	508,8	254,8
YPK_0010	hypothetical protein	4,29	0,6	0,7	0,7	398,9	0,4	199,7
YPK_0283	rplC 50S ribosomal protein L3	4,28	840,2	1,3	420,8	217,5	1947,9	1082,7
YPK_2760	hypothetical protein	4,27	0,9	1,0	1,0	558,0	0,6	279,3
YPK_2129	endoribonuclease L-PSP	4,26	0,6	0,7	0,7	392,2	0,4	196,3
YPK_3481	hypothetical protein	4,25	0,6	0,7	0,7	388,9	0,4	194,7
YPK_3759	MuA-transposase/repressor protein CIDNA-binding	4,25	0,6	0,7	0,7	388,9	0,4	194,7
YPK_0992	PTS system mannose/fructose/sorbose familytrans	4,25	0,9	1,1	1,0	0,7	490,8	245,8
YPK_3606	two-component response regulator	4,23	1,3	1,5	1,4	1,0	685,9	343,5
YPK_0538	hypothetical protein	4,23	0,6	0,8	0,7	0,5	342,5	171,5
YPK_3549	hypothetical protein	4,23	0,6	0,8	0,7	0,5	342,5	171,5
YPK_0172	ompR osmolarity response regulator	4,22	1,3	1,5	1,4	1,0	683,1	342,1
YPK_3513	cell division protein FtsZ	4,21	2,1	179,7	90,9	239,6	536,4	388,0
YPK_1426	PTS system glucose-specific transporter	4,21	0,9	1,1	1,0	0,7	479,5	240,1
YPK_1221	XRE family transcriptional regulator	4,20	0,7	0,8	0,7	0,5	336,9	168,7
YPK_0527	ClpXP protease specificity-enhancing factor	4,20	0,9	1,1	1,0	0,7	474,0	237,4
YPK_2552	uridine kinase	4,18	1,2	1,4	1,3	0,9	573,6	287,3
YPK_3586	FKBP-type peptidylprolyl isomerase	4,17	1,0	1,1	1,0	520,5	0,7	260,6
YPK_3155	phosphoribosylaminoimidazole carboxylasecatalytic	4,17	1,0	1,1	1,0	0,7	466,0	233,4
YPK_3723	hypothetical protein	4,17	0,7	0,8	0,7	367,4	0,5	183,9
YPK_1151	replication initiation regulator SeqA	4,16	1,0	1,1	1,0	517,6	0,7	259,1
YPK_2990	flavodoxin FldA	4,16	1,0	1,1	1,0	517,6	0,7	259,1
YPK_1033	dinucleoside polyphosphate hydrolase	4,16	1,0	1,1	1,0	0,7	463,4	232,1
YPK_2523	hypothetical protein	4,14	1,0	1,1	1,1	0,8	455,8	228,3
YPK_1701	hypothetical protein	4,12	1,0	1,2	1,1	503,5	0,7	252,1
YPK_3808	oligoribonuclease	4,12	1,0	1,2	1,1	500,8	0,7	250,8
YPK_1568	NADH dehydrogenase subunit J	4,12	1,0	1,2	1,1	0,8	448,4	224,6
YPK_3363	rimM 16S rRNA-processing protein RimM	4,11	1,0	1,2	1,1	0,8	446,0	223,4
YPK_3813	fumarate reductase flavoprotein subunit	4,10	3,3	341,6	172,5	303,7	1087,6	695,6
YPK_0549	hypothetical protein	4,09	0,7	0,8	0,8	348,1	0,5	174,3
YPK_2709	chorismate mutase	4,08	1,0	1,2	1,1	487,7	0,7	244,2
YPK_0448	carboxymuconolactone decarboxylase	4,07	0,7	0,8	0,8	342,9	0,5	171,7
YPK_1563	NADH dehydrogenase subunit E	4,07	1,0	1,2	1,1	0,8	434,4	217,6
YPK_2669	cmk cytidylate kinase	4,07	1,3	1,5	1,4	594,1	0,9	297,5
YPK_1097	D,D-heptose 1,7-bisphosphate phosphatase	4,06	1,0	1,2	1,1	0,8	432,1	216,5
YPK_3573	ksgA dimethyladenosine transferase	4,04	1,5	1,7	1,6	1,2	601,5	301,3
YPK_3225	cof family hydrolase	4,03	1,5	1,7	1,6	1,2	599,4	300,3
YPK_0249	hypothetical protein	4,03	0,7	0,9	0,8	0,6	298,2	149,4
YPK_0268	FKBP-type peptidyl-prolyl cis-trans isomerase	4,03	302,8	1,2	152,0	470,4	210,6	340,5
YPK_2006	Na(+)-translocating NADH-quinone reductasesubur	4,03	1,1	1,2	1,1	0,8	421,2	211,0
YPK_2551	dcd deoxycytidine triphosphate deaminase	4,03	1,1	1,2	1,1	0,8	421,2	211,0
YPK_2855	catalase	4,03	123,6	3,0	63,3	192,0	515,9	353,9
YPK_1296	outer membrane protein assembly complex subunit	4,02	2,1	175,2	88,7	233,6	418,3	326,0
YPK_0581	hypothetical protein	4,00	0,8	0,9	0,8	326,1	0,5	163,3
YPK_3301	Na(+)-translocating NADH-quinone reductasesubur	3,99	1,1	1,3	1,2	458,7	0,8	229,7
YPK_4210	D-ribose pyranase	3,99	0,8	0,9	0,8	0,6	289,9	145,3
YPK_3746	putative phage terminase, small subunit	3,98	1,1	1,3	1,2	0,9	406,7	203,8
YPK_1883	glutathionine S-transferase	3,97	291,0	1,3	146,1	226,0	404,8	315,4
YPK_2126	Slp family outer membrane lipoprotein	3,97	1,1	1,3	1,2	0,9	404,8	202,8
YPK_0656	hypothetical protein	3,97	0,8	0,9	0,8	319,3	0,5	159,9
YPK_3683	hypothetical protein	3,95	0,8	0,9	0,9	315,0	0,5	157,8
YPK_0855	arginine exporter protein	3,94	1,1	1,3	1,2	0,9	397,0	198,9

YPK_1007	general secretion pathway protein G	3,93	0,8	0,9	0,9	310,8	0,6	155,7
YPK_2913	DNA-binding transcriptional activator XapR	3,91	1,6	1,9	1,8	1,3	551,6	276,4
YPK_3302	Na(+)-translocating NADH-quinone reductasesubur	3,91	1,1	1,3	1,2	435,1	0,8	217,9
YPK_3191	glucose-1-phosphate cytidylyltransferase	3,91	1,4	1,6	1,5	1,1	477,0	239,1
YPK_3562	type VI secretion system lysozyme-like protein	3,91	0,8	0,9	0,9	306,7	0,6	153,6
YPK_2309	hypothetical protein	3,89	1,2	1,4	1,3	429,1	0,8	214,9
YPK_3163	hypothetical protein	3,89	0,8	1,0	0,9	0,6	271,0	135,8
YPK_1656	autoinducer synthesis protein	3,88	1,2	1,4	1,3	0,9	380,7	190,8
YPK_2632	hypothetical protein	3,87	0,8	1,0	0,9	298,8	0,6	149,7
YPK_0638	putative glycerol-3-phosphate acyltransferasePlsY	3,87	1,2	1,4	1,3	421,3	0,8	211,0
YPK_0792	autoinducer synthesis protein	3,87	1,2	1,4	1,3	421,3	0,8	211,0
YPK_3291	xanthine-guanine phosphoribosyltransferase	3,86	0,8	1,0	0,9	296,9	0,6	148,7
YPK_0649	ribB 3,4-dihydroxy-2-butanone 4-phosphate syntha	3,86	1,2	1,4	1,3	0,9	375,5	188,2
YPK_3941	twin-arginine translocation protein subunitTatB	3,84	1,2	1,4	1,3	0,9	370,5	185,7
YPK_0373	pgi glucose-6-phosphate isomerase	3,83	108,2	3,5	55,8	252,1	300,9	276,5
YPK_3978	cell division protein FtsE	3,83	1,2	1,4	1,3	0,9	367,2	184,1
YPK_3579	hypothetical protein	3,83	0,9	1,0	0,9	0,7	259,2	129,9
YPK_4232	pstC phosphate transporter permease subunit Pst	3,82	1,7	2,0	1,9	1,3	515,7	258,5
YPK_0281	bacterioferritin	3,82	0,9	1,0	0,9	0,7	257,6	129,1
YPK_1948	nucleotidase	3,82	1,2	1,4	1,3	406,5	0,9	203,7
YPK_0864	2-octaprenyl-6-methoxyphenyl hydroxylase	3,81	2,1	2,5	2,3	1,7	629,1	315,4
YPK_2959	colicin uptake protein TolQ	3,81	1,2	1,4	1,3	1,0	362,4	181,7
YPK_2388	death-on-curing family protein	3,81	0,9	1,0	0,9	285,9	0,6	143,2
YPK_0451	hypothetical protein	3,81	0,9	1,0	0,9	0,7	256,0	128,3
YPK_3270	short chain dehydrogenase	3,80	1,2	1,4	1,3	1,0	360,8	180,9
YPK_3032	cold-shock DNA-binding domain-containingprotein	3,80	0,9	1,0	0,9	284,1	0,6	142,4
YPK_3060	Hcp1 family type VI secretion system effector	3,80	0,9	1,0	0,9	284,1	0,6	142,4
YPK_1090	hypothetical protein	3,80	1,2	1,4	1,3	1,0	359,3	180,1
YPK_1817	multiple drug resistance protein MarC	3,80	1,2	1,4	1,3	1,0	359,3	180,1
YPK_2429	invasin region 3	3,79	5,2	6,1	5,7	143,0	42,7	92,9
YPK_1173	uracil-DNA glycosylase	3,79	1,2	1,5	1,3	1,0	357,7	179,3
YPK_2423	flgD flagellar basal body rod modification protein	3,79	1,2	1,5	1,3	1,0	357,7	179,3
YPK_1566	NADH dehydrogenase subunit H	3,79	1,8	422,8	212,3	281,8	504,7	393,3
YPK_0463	quinone oxidoreductase	3,79	1,8	2,1	1,9	1,4	504,7	253,0
YPK_2419	flgH flagellar basal body L-ring protein	3,78	1,3	1,5	1,4	397,8	0,9	199,3
YPK_4106	ribonuclease activity regulator protein RraA	3,78	0,9	1,0	1,0	0,7	251,3	126,0
YPK_1743	MgtC/SapB transporter	3,77	1,3	1,5	1,4	1,0	351,7	176,3
YPK_3935	fre FMN reductase	3,76	1,3	1,5	1,4	1,0	350,2	175,6
YPK_0696	pili assembly chaperone	3,72	1,3	1,5	1,4	381,4	0,9	191,2
YPK_3617	OmpA/MotB domain-containing protein	3,72	0,9	1,1	1,0	0,7	241,1	120,9
YPK_2159		3,72	2,9	127,8	65,4	426,0	152,6	289,3
YPK_3245	protoheme IX farnesyltransferase	3,72	1,6	232,6	117,1	155,0	416,5	285,8
YPK_3524	peptidoglycan glycosyltransferase	3,71	303,1	3,7	153,4	392,4	491,9	442,2
YPK_3587	lspA lipoprotein signal peptidase	3,71	0,9	1,1	1,0	267,7	0,6	134,2
YPK_2952	nicotinamide mononucleotide transporter PnuC	3,71	1,3	1,5	1,4	378,3	0,9	189,6
YPK_0316	def peptide deformylase	3,70	0,9	1,1	1,0	0,7	238,4	119,5
YPK_3874	integral membrane protein TerC	3,70	1,9	398,6	200,2	531,4	237,9	384,7
YPK_3858	tRNA-dihydrouridine synthase A	3,70	1,9	2,2	2,0	1,5	475,8	238,6
YPK_3440	phosphoadenosine phosphosulfate reductase	3,69	1,3	1,6	1,4	373,8	0,9	187,3
YPK_3563	hypothetical protein	3,69	0,9	1,1	1,0	263,2	0,7	131,9
YPK_1247	hypothetical protein	3,69	0,9	1,1	1,0	0,7	235,7	118,2
YPK_3015	leuS leucyl-tRNA synthetase	3,69	69,1	5,4	37,3	322,2	192,3	257,2
YPK_1600	glnH glutamine ABC transporter periplasmic protei	3,68	1,3	1,6	1,5	1,0	330,7	165,9
YPK_0775	hypothetical protein	3,67	1,0	1,1	1,0	260,2	0,7	130,5
YPK_1606	ompX outer membrane protein X	3,67	1,0	1,1	1,0	260,2	0,7	130,5
YPK_3511	hypothetical protein	3,67	1,0	1,1	1,0	0,7	233,0	116,9
YPK_1165	putative hydrolase-oxidase	3,67	1,4	1,6	1,5	367,8	0,9	184,4
YPK_3000	UMP phosphatase	3,66	1,4	1,6	1,5	364,9	0,9	182,9
YPK_1944	fumarate/nitrate reduction transcriptionalregulator	3,66	1,4	1,6	1,5	1,1	326,8	163,9

YPK_3945	ubiE ubiquinone/menaquinone biosynthesismethyl	3,65	1,4	1,6	1,5	1,1	325,5	163,3
YPK_1735	hypothetical protein	3,65	1,0	1,1	1,1	255,9	0,7	128,3
YPK_2153	copper homeostasis protein CutC	3,64	1,4	1,6	1,5	1,1	321,7	161,4
YPK_3611	NTPase	3,62	1,0	1,2	1,1	251,8	0,7	126,2
YPK_1567	NADH dehydrogenase subunit I	3,62	1,0	1,2	1,1	0,8	225,4	113,1
YPK_0002	DNA polymerase III subunit beta	3,62	2,0	2,3	2,2	501,3	1,4	251,3
YPK_1475	fimbrial protein	3,62	1,0	1,2	1,1	250,4	0,7	125,6
YPK_0995	DeoR family transcriptional regulator	3,61	1,4	1,6	1,5	353,8	1,0	177,4
YPK_3160	short chain dehydrogenase	3,61	1,4	1,6	1,5	353,8	1,0	177,4
YPK_1088	YaeQ family protein	3,61	1,0	1,2	1,1	0,8	223,0	111,9
YPK_0380	maltose/maltodextrin transporter ATP-bindingprotein	3,61	2,0	2,3	2,2	497,2	1,4	249,3
YPK_1544	phosphodiesterase	3,60	1,0	1,2	1,1	0,8	221,8	111,3
YPK_3515	cell division protein FtsQ	3,59	1,4	1,7	1,6	1,1	310,9	156,0
YPK_3796	FtsH protease regulator HflK	3,58	2,3	2,7	2,5	1,8	489,7	245,7
YPK_2465	PTS system mannose/fructose/sorbose familytransp	3,58	1,4	1,7	1,6	1,1	308,6	154,9
YPK_2415	flgL flagellar hook-associated protein FlgL	3,57	1,8	2,1	1,9	1,4	377,4	189,4
YPK_2376	cystine transporter subunit	3,57	1,5	1,7	1,6	1,1	307,4	154,3
YPK_0509	3-deoxy-D-manno-octulosonate 8-phosphatephosph	3,57	1,0	1,2	1,1	0,8	217,2	109,0
YPK_0227	hypothetical protein	3,56	1,8	2,1	1,9	417,7	1,2	209,5
YPK_1128	ABC transporter-like protein	3,56	1,5	1,7	1,6	340,8	1,0	170,9
YPK_2223	N-formylglutamate amidohydrolase	3,56	1,5	1,7	1,6	340,8	1,0	170,9
YPK_2426	flgA flagellar basal body P-ring biosynthesis protei	3,56	1,5	1,7	1,6	1,1	304,1	152,6
YPK_1536	3-octaprenyl-4-hydroxybenzoate carboxy-lyase	3,55	1,0	1,2	1,1	238,8	0,7	119,8
YPK_1698	membrane lipoprotein lipid attachment site	3,54	1,0	1,2	1,1	237,6	0,7	119,2
YPK_3872	stress protein	3,54	1,0	1,2	1,1	237,6	0,7	119,2
YPK_0645	bifunctional heptose 7-phosphate kinase/heptose1-	3,53	2,6	3,0	2,8	2,0	519,1	260,5
YPK_4136	cysE serine acetyltransferase	3,53	1,5	1,7	1,6	1,2	298,6	149,9
YPK_2178	N5-glutamine S-adenosyl-L-methionine-dependentr	3,52	1,5	1,8	1,6	1,2	296,5	148,8
YPK_2211	hypothetical protein	3,52	1,1	1,2	1,2	234,0	0,7	117,4
YPK_4144	tdh L-threonine 3-dehydrogenase	3,51	1,9	2,2	2,0	403,2	1,3	202,2
YPK_2038	cob(I)yrinic acid a,c-diamideadenosyltransferase	3,50	1,1	1,3	1,2	231,7	0,7	116,2
YPK_1080	rnhB ribonuclease HII	3,49	1,1	1,3	1,2	0,8	205,4	103,1
YPK_0437	putative glutathione S-transferase YghU	3,48	1,5	1,8	1,7	321,9	1,1	161,5
YPK_1591	naphthoate synthase	3,47	1,6	1,8	1,7	320,8	1,1	160,9
YPK_2676	formate transporter	3,47	1,6	1,8	1,7	320,8	1,1	160,9
YPK_3188	NAD-dependent epimerase/dehydratase	3,47	1,6	1,8	1,7	320,8	1,1	160,9
YPK_1625	hypothetical protein	3,47	1,9	2,2	2,1	1,5	350,8	176,2
YPK_4072	glutamate racemase	3,46	1,6	1,8	1,7	1,2	285,3	143,2
YPK_4161	hypothetical protein	3,46	1,6	1,8	1,7	1,2	285,3	143,2
YPK_4147	ADP-heptose--LPS heptosyltransferase	3,46	1,9	2,2	2,1	1,5	347,9	174,7
YPK_3841	transcriptional activator RhaR	3,45	1,6	1,8	1,7	315,4	1,1	158,2
YPK_3632	hypothetical protein	3,45	1,1	1,3	1,2	222,8	0,8	111,8
YPK_2144	ruvA Holliday junction DNA helicase RuvA	3,45	1,1	1,3	1,2	0,9	199,5	100,2
YPK_0510	D-arabinose 5-phosphate isomerase	3,45	1,9	2,3	2,1	385,3	1,3	193,3
YPK_3008	transporter-associated protein	3,44	1,6	1,9	1,7	1,2	280,5	140,8
YPK_3716	peptidase U32	3,44	1,6	1,9	1,7	1,2	280,5	140,8
YPK_1784	heat shock protein HtpX	3,43	1,6	1,9	1,7	1,2	279,5	140,4
YPK_4242	hypothetical protein	3,43	1,1	1,3	1,2	220,6	0,8	110,7
YPK_2007	electron transport complex protein RnfB	3,43	1,1	1,3	1,2	219,6	0,8	110,2
YPK_0447	3-hydroxyisobutyrate dehydrogenase	3,42	1,6	1,9	1,7	1,3	276,7	139,0
YPK_0990	PTS system mannose/fructose/sorbose familytransp	3,42	1,6	1,9	1,7	1,3	276,7	139,0
YPK_3233	clpX ATP-dependent protease ATP-binding subuni	3,41	2,3	2,7	2,5	434,4	1,6	218,0
YPK_0665	repressor protein for FtsI	3,41	2,6	3,0	2,8	2,0	434,4	218,2
YPK_0655	nudF ADP-ribose pyrophosphatase NudF	3,41	1,1	1,3	1,2	216,5	0,8	108,7
YPK_2237	N-acetyltransferase GCN5	3,41	1,6	1,9	1,8	306,0	1,1	153,6
YPK_1362	dihydrodipicolinate synthase	3,41	1,6	1,9	1,8	1,3	274,0	137,6
YPK_2068	binding-protein-dependent transport system innerm	3,40	1,6	1,9	1,8	304,0	1,1	152,6
YPK_0569	stress protein	3,39	1,2	1,4	1,3	213,5	0,8	107,2
YPK_2111	disulfide bond formation protein B	3,39	1,2	1,4	1,3	213,5	0,8	107,2

YPK_3280	rdgC recombination associated protein	3,39	1,6	1,9	1,8	1,3	270,4	135,9
YPK_1759	chemotaxis regulator CheZ	3,38	1,2	1,4	1,3	0,9	190,3	95,6
YPK_1372	succinyl-diaminopimelate desuccinylase	3,37	2,0	2,4	2,2	1,6	328,6	165,1
YPK_3571	peptidyl-prolyl cis-trans isomerase SurA	3,37	2,4	2,7	2,5	1,8	379,2	190,5
YPK_3616	diguanylate cyclase	3,37	2,4	158,1	80,2	210,8	188,7	199,8
YPK_3944	sterol-binding domain-containing protein	3,37	1,2	1,4	1,3	210,6	0,8	105,7
YPK_0534	isoprenoid biosynthesis protein withamidotransferas	3,36	1,2	1,4	1,3	0,9	187,7	94,3
YPK_3603	homoserine kinase	3,36	1,7	2,0	1,8	1,3	265,2	133,3
YPK_1513	N5-glutamine S-adenosyl-L-methionine-dependentr	3,35	1,7	2,0	1,8	1,3	264,4	132,9
YPK_2356	response regulator	3,35	1,2	1,4	1,3	208,7	0,8	104,8
YPK_1451	hypothetical protein	3,35	1,2	1,4	1,3	0,9	186,9	93,9
YPK_0134	LacI family transcriptional regulator	3,34	1,7	2,0	1,8	1,3	262,7	132,0
YPK_0865	hypothetical protein	3,33	2,4	2,8	2,6	1,9	368,3	185,1
YPK_3297	glycerophosphoryl diester phosphodiesterase	3,33	1,7	2,0	1,9	1,3	260,3	130,8
YPK_4002	magnesium/nickel/cobalt transporter CorA	3,33	1,7	2,0	1,9	289,8	1,2	145,5
YPK_0458	acetyl-CoA carboxylase biotin carboxylasesubunit	3,33	2,4	2,8	2,6	409,5	1,7	205,6
YPK_0114	glutathione reductase	3,32	2,4	2,8	2,6	1,9	365,9	183,9
YPK_1298	auxin efflux carrier	3,32	1,7	2,0	1,9	1,3	257,8	129,6
YPK_1923	DeoR family transcriptional regulator	3,32	1,7	2,0	1,9	1,3	257,8	129,6
YPK_3882	hypothetical protein	3,31	1,7	2,0	1,9	1,3	257,0	129,2
YPK_0453	tRNA-dihydrouridine synthase B	3,30	1,7	2,0	1,9	285,3	1,2	143,3
YPK_3027	lipoyl synthase	3,30	1,7	2,0	1,9	1,4	255,5	128,4
YPK_1868	ribonuclease T	3,30	1,2	1,4	1,3	201,5	0,9	101,2
YPK_2623	hypothetical protein	3,30	1,2	1,4	1,3	201,5	0,9	101,2
YPK_1815	periplasmic solute binding protein	3,30	1,8	213,3	107,5	284,4	127,3	205,9
YPK_1444	glk glucokinase	3,30	1,8	2,0	1,9	1,4	253,9	127,6
YPK_3621	DNA repair protein RadA	3,29	2,5	2,9	2,7	1,9	358,0	180,0
YPK_4006	TetR family transcriptional regulator	3,29	1,2	1,5	1,3	199,8	0,9	100,3
YPK_4206	GntR family transcriptional regulator	3,28	1,3	1,5	1,4	198,9	0,9	99,9
YPK_0633	hypothetical protein	3,28	1,3	1,5	1,4	1,0	178,1	89,5
YPK_3856	quinone oxidoreductase	3,28	1,8	2,1	1,9	1,4	250,8	126,1
YPK_1906	extracellular solute-binding protein	3,27	330,5	3,4	167,0	684,6	153,2	418,9
YPK_2664	lpxK tetraacyldisaccharide 4'-kinase	3,27	1,8	2,1	1,9	279,3	1,2	140,3
YPK_0543	putative glutathione S-transferase	3,27	1,8	2,1	1,9	1,4	250,1	125,7
YPK_4197	serine/threonine protein kinase	3,27	1,8	2,1	1,9	1,4	250,1	125,7
YPK_1358	hypothetical protein	3,27	1,3	1,5	1,4	197,2	0,9	99,0
YPK_2936	modB molybdate ABC transporter permease	3,27	1,3	1,5	1,4	1,0	176,6	88,8
YPK_0531	putative radical SAM protein	3,27	1,8	2,1	1,9	278,5	1,2	139,8
YPK_3192	CDP-6-deoxy-delta-3,4-glucoseen reductase	3,27	1,8	2,1	1,9	1,4	249,3	125,4
YPK_0900	small terminase subunit	3,27	1,3	1,5	1,4	1,0	175,8	88,4
YPK_2242	putative endopeptidase	3,27	2,2	2,6	2,4	340,1	1,5	170,8
YPK_1918	NAD-binding D-isomer specific 2-hydroxyaciddehyc	3,26	178,7	2,1	90,4	138,8	248,6	193,7
YPK_1418	putative peptidase	3,26	2,2	2,6	2,4	1,7	303,8	152,8
YPK_0559	oxidoreductase domain-containing protein	3,26	1,8	2,1	1,9	1,4	247,8	124,6
YPK_1105	mltD membrane-bound lytic murein transglycosyla	3,25	2,6	3,0	2,8	2,0	349,0	175,5
YPK_2414	DeoR family transcriptional regulator	3,25	1,8	2,1	2,0	1,4	246,4	123,9
YPK_2690	aat leucyl/phenylalanyl-tRNA--protein transferase	3,24	1,3	1,5	1,4	1,0	172,9	87,0
YPK_0641	cca multifunctional tRNA nucleotidyltransferase/2'3	3,24	2,2	2,6	2,4	334,4	1,6	168,0
YPK_2246	ABC transporter-like protein	3,24	1,3	1,5	1,4	1,0	172,2	86,6
YPK_0646	inner membrane protein YjeH	3,23	2,3	2,6	2,4	1,8	296,6	149,2
YPK_3653	autoinducer AI-2 ABC transporter periplasmicAI-2-b	3,23	1,8	2,1	2,0	1,4	242,1	121,7
YPK_0025	putative lipoprotein	3,22	1,3	1,5	1,4	190,7	0,9	95,8
YPK_2702	HCP oxidoreductase	3,22	1,8	2,2	2,0	1,4	241,4	121,4
YPK_0263	glycosyl transferase family protein	3,22	1,9	2,2	2,0	268,8	1,3	135,0
YPK_1893	pspF phage shock protein operon transcriptionala	3,21	1,9	2,2	2,0	1,4	240,0	120,7
YPK_3013	ABC transporter-like protein	3,21	1,3	1,5	1,4	189,2	0,9	95,0
YPK_0506	putative ABC transporter ATP-binding proteinYhbG	3,21	1,3	1,5	1,4	1,0	169,4	85,2
YPK_1191	recO DNA repair protein RecO	3,21	1,3	1,5	1,4	1,0	169,4	85,2
YPK_1683	putative glycerol-3-phosphate acyltransferasePlsX	3,21	1,9	2,2	2,0	1,5	238,6	120,0

YPK_0663		1-acyl-sn-glycerol-3-phosphate acyltransferase	3,20	1,3	1,5	1,4	187,6	0,9	94,3
YPK_2089		selenophosphate synthetase	3,19	1,9	2,2	2,0	1,5	235,9	118,7
YPK_2563		hypothetical protein	3,19	1,3	1,6	1,4	186,1	0,9	93,5
YPK_0131		two component transcriptional regulator	3,19	1,3	1,6	1,4	1,0	166,7	83,9
YPK_2498		TRAP dicarboxylate transporter subunit DctM	3,18	2,3	2,7	2,5	1,8	287,0	144,4
YPK_2780		GntR family transcriptional regulator	3,18	1,3	1,6	1,5	184,7	0,9	92,8
YPK_3296		DNA polymerase IV	3,17	1,9	2,2	2,1	1,5	233,2	117,4
YPK_3326		ABC transporter-like protein	3,17	1,4	1,6	1,5	183,9	0,9	92,4
YPK_3207		RND family efflux transporter MFP subunit	3,17	2,1	348,7	175,4	116,2	520,3	318,3
YPK_2953		quinolinate synthetase	3,17	1,9	2,2	2,1	1,5	232,6	117,0
YPK_1258		hypothetical protein	3,16	1,9	2,2	2,1	1,5	231,3	116,4
YPK_3518	murG	undecaprenyldiphospho-muramoylpentapep	3,16	1,9	2,3	2,1	257,6	1,3	129,5
YPK_3925		Integrase catalytic subunit	3,15	1,4	1,6	1,5	181,8	1,0	91,4
YPK_2421	flgF	flagellar basal body rod protein FlgF	3,15	1,4	1,6	1,5	1,1	162,7	81,9
YPK_0976	lacI	lac repressor	3,15	1,9	2,3	2,1	1,5	230,0	115,8
YPK_3190		CDP-glucose 4,6-dehydratase	3,15	1,9	2,3	2,1	1,5	230,0	115,8
YPK_0157		DNA-binding transcriptional repressor GlpR	3,15	1,4	1,6	1,5	1,1	162,1	81,6
YPK_1180		methyltransferase small	3,15	1,4	1,6	1,5	1,1	162,1	81,6
YPK_3005		(dimethylallyl)adenosine tRNAmethylthiotransferase	3,15	116,4	3,2	59,8	180,8	161,9	171,4
YPK_3299		ApbE family lipoprotein	3,14	1,9	2,3	2,1	1,5	228,7	115,1
YPK_2028		phosphatidylglycerophosphatase B	3,14	1,4	1,6	1,5	180,3	1,0	90,7
YPK_3950		uridine phosphorylase	3,14	1,4	1,6	1,5	1,1	161,5	81,3
YPK_3492	pdhR	transcriptional regulator PdhR	3,14	1,4	1,6	1,5	1,1	160,9	81,0
YPK_2221		histidine utilization repressor	3,13	1,4	1,6	1,5	179,0	1,0	90,0
YPK_2155	argS	arginyl-tRNA synthetase	3,13	3,1	3,6	3,4	2,4	358,0	180,2
YPK_3734	glmM	phosphoglucosamine mutase	3,13	2,4	2,8	2,6	309,2	1,7	155,4
YPK_1464		murein hydrolase B	3,13	2,0	2,3	2,1	252,0	1,4	126,7
YPK_1060		tRNA pseudouridine synthase C	3,12	1,4	1,6	1,5	177,6	1,0	89,3
YPK_1274		RNA methyltransferase	3,12	1,4	1,6	1,5	177,6	1,0	89,3
YPK_2233		calcium/sodium:proton antiporter	3,12	2,0	2,3	2,2	250,6	1,4	126,0
YPK_2880		hypothetical protein	3,11	2,0	2,3	2,2	1,5	223,8	112,7
YPK_2937	modA	molybdate transporter periplasmic protein	3,11	1,4	1,6	1,5	176,2	1,0	88,6
YPK_2906		NADH:flavin oxidoreductase	3,11	2,0	2,3	2,2	248,6	1,4	125,0
YPK_0650		hypothetical protein	3,10	1,4	1,7	1,5	175,6	1,0	88,3
YPK_2420	flgG	flagellar basal body rod protein FlgG	3,10	1,4	1,7	1,5	175,6	1,0	88,3
YPK_2593		hypothetical protein	3,10	1,4	1,7	1,5	1,1	157,2	79,2
YPK_3736	hflB	ATP-dependent metalloprotease	3,09	3,5	4,1	3,8	2,7	382,8	192,8
YPK_3067		cupin 4 family protein	3,09	2,0	2,4	2,2	1,6	220,3	110,9
YPK_1527		NAD-binding D-isomer specific 2-hydroxyaciddehyc	3,08	2,0	2,4	2,2	244,7	1,4	123,1
YPK_1293	ispG	4-hydroxy-3-methylbut-2-en-1-yl diphosphate	3,08	2,0	2,4	2,2	1,6	219,1	110,3
YPK_0552		DNA-binding transcriptional repressor ExuR	3,08	1,4	1,7	1,6	173,0	1,0	87,0
YPK_3483		S-adenosylmethionine decarboxylase	3,08	1,4	1,7	1,6	1,1	154,9	78,0
YPK_4026		TDP-4-oxo-6-deoxy-D-glucose transaminase	3,08	2,0	2,4	2,2	1,6	218,5	110,1
YPK_2148	aspS	aspartyl-tRNA synthetase	3,08	3,2	3,8	3,5	2,5	345,0	173,7
YPK_1227		hypothetical protein	3,07	2,0	2,4	2,2	1,6	217,9	109,8
YPK_2047	trpA	tryptophan synthase subunit alpha	3,06	1,5	1,7	1,6	170,4	1,0	85,7
YPK_2506	lldD	L-lactate dehydrogenase	3,06	2,1	2,4	2,2	1,6	215,7	108,6
YPK_1953		putative virulence factor SrfB	3,05	2,1	2,4	2,3	1,6	214,6	108,1
YPK_1888		ABC transporter-like protein	3,04	1,5	1,7	1,6	1,1	150,9	76,0
YPK_4046		pili assembly chaperone	3,04	1,5	1,7	1,6	1,2	150,4	75,8
YPK_0027		mannitol-1-phosphate 5-dehydrogenase	3,04	2,1	2,4	2,3	1,6	212,4	107,0
YPK_2091		protease 4	3,03	3,3	3,9	3,6	2,6	334,9	168,8
YPK_0036		formate dehydrogenase accessory protein	3,03	1,5	1,7	1,6	1,2	149,3	75,2
YPK_3740		D-alanyl-D-alanine carboxypeptidase	3,02	2,6	3,0	2,8	2,0	256,3	129,2
YPK_0061		integrase family protein	3,00	2,1	2,5	2,3	1,7	207,6	104,6
YPK_2778		mannonate dehydratase	3,00	2,2	2,5	2,3	1,7	207,1	104,4
YPK_2572		S-formylglutathione hydrolase	3,00	1,5	1,8	1,7	163,2	1,1	82,1
YPK_1014		prepilin peptidase	3,00	1,5	1,8	1,7	1,2	146,1	73,7
YPK_3448	mazG	nucleoside triphosphate pyrophosphohydro	3,00	1,5	1,8	1,7	1,2	146,1	73,7

YPK_3519	cell division protein FtsW	2,99	2,2	2,5	2,3	1,7	205,5	103,6
YPK_3470	pcnB poly(A) polymerase I	2,99	2,7	3,1	2,9	281,1	1,8	141,5
YPK_2740	serine-type D-Ala-D-Ala carboxypeptidase	2,99	2,2	171,8	87,0	114,5	205,0	159,8
YPK_1864	NLP/P60 protein	2,98	1,5	1,8	1,7	1,2	144,6	72,9
YPK_0502	hypothetical protein	2,98	1,5	1,8	1,7	161,0	1,1	81,0
YPK_2866	rhodanese domain-containing protein	2,98	1,5	1,8	1,7	1,2	144,1	72,7
YPK_3898	hypothetical protein	2,98	2,2	2,6	2,4	1,7	203,5	102,6
YPK_4229	glucosamine--fructose-6-phosphateaminotransferas	2,97	3,3	227,0	115,2	227,0	203,3	215,1
YPK_0254	phosphoribulokinase	2,95	1,6	1,8	1,7	1,2	141,7	71,4
YPK_3304	Na(+)-translocating NADH-quinone reductasesubur	2,94	143,2	2,6	72,9	222,4	99,6	161,0
YPK_3654	aldolase	2,94	1,6	1,8	1,7	1,2	140,7	71,0
YPK_3999	phospholipase A	2,94	1,6	1,9	1,7	1,2	140,2	70,7
YPK_3467	sugar fermentation stimulation protein A	2,93	1,6	1,9	1,7	156,1	1,1	78,6
YPK_3708	binding-protein-dependent transport system innerm	2,93	1,6	1,9	1,7	1,2	139,3	70,3
YPK_2072	bifunctional acetaldehyde-CoA/alcoholdehydrogena	2,93	4,8	77,8	41,3	51,8	417,7	234,8
YPK_1546	PTS system ascorbate-specific transportersubunit I	2,93	2,3	2,6	2,5	1,8	196,8	99,3
YPK_1747	flagellar motor protein MotA	2,92	1,6	1,9	1,7	155,0	1,1	78,1
YPK_4030	wecC UDP-N-acetyl-D-mannosamine dehydrogen	2,92	2,3	2,7	2,5	1,8	195,9	98,8
YPK_0215	XRE family transcriptional regulator	2,92	1,6	1,9	1,7	1,3	138,4	69,8
YPK_1324	lipid kinase	2,92	1,6	1,9	1,7	1,3	138,4	69,8
YPK_2009	AraC family transcriptional regulator	2,91	1,6	1,9	1,8	154,0	1,1	77,6
YPK_2125	long-chain-fatty-acid--CoA ligase	2,91	3,0	245,9	124,4	81,9	513,6	297,8
YPK_2578	molybdopterin biosynthesis protein MoeA	2,91	2,3	2,7	2,5	1,8	194,0	97,9
YPK_2670	3-phosphoshikimate 1-carboxyvinyltransferase	2,89	2,3	2,7	2,5	214,7	1,6	108,2
YPK_4036	ATP-dependent RNA helicase RhlB	2,89	2,3	2,7	2,5	1,8	192,2	97,0
YPK_3497	quinolinate phosphoribosyltransferase	2,89	1,6	1,9	1,8	1,3	135,7	68,5
YPK_3602	threonine synthase	2,89	137,9	2,7	70,3	107,1	191,8	149,4
YPK_0382	maltose regulon periplasmic protein	2,89	1,6	1,9	1,8	1,3	135,2	68,2
YPK_0660	LysR family transcriptional regulator	2,89	1,6	1,9	1,8	1,3	135,2	68,2
YPK_3248	2-dehydropantoate 2-reductase	2,89	1,6	1,9	1,8	1,3	135,2	68,2
YPK_1983	major facilitator transporter	2,88	2,3	2,7	2,5	1,8	190,5	96,1
YPK_2826	Hrp-dependent type III effector protein	2,87	2,4	2,7	2,5	1,8	189,6	95,7
YPK_3176	inosine kinase	2,87	2,4	2,7	2,5	1,8	189,6	95,7
YPK_1508	fadI 3-ketoacyl-CoA thiolase	2,87	2,4	2,8	2,6	1,8	188,7	95,3
YPK_3189	DegT/DnrJ/EryC1/StrS aminotransferase	2,86	2,4	2,8	2,6	210,3	1,6	106,0
YPK_3920	gltP glutamate/aspartate:proton symporter	2,86	2,4	2,8	2,6	1,8	187,9	94,9
YPK_1045	N-acetylglutamate synthase	2,85	2,4	2,8	2,6	1,9	186,6	94,2
YPK_1638	hypothetical protein	2,84	1,7	2,0	1,8	1,3	131,4	66,3
YPK_2863	ABC transporter-like protein	2,84	1,7	2,0	1,8	1,3	131,4	66,3
YPK_3382	transketolase domain-containing protein	2,84	1,7	2,0	1,8	1,3	130,5	65,9
YPK_0119	glutamate dehydrogenase	2,83	2,4	154,2	78,3	102,8	184,1	143,5
YPK_2184	ribose-phosphate pyrophosphokinase	2,83	1,7	2,0	1,9	1,3	130,1	65,7
YPK_0438	hypothetical protein	2,83	2,4	2,8	2,6	1,9	183,7	92,8
YPK_0328	pantothenate kinase	2,83	1,7	2,0	1,9	144,9	1,2	73,0
YPK_1168	urea amidolyase-like protein	2,83	1,7	2,0	1,9	144,9	1,2	73,0
YPK_3170		2,83	3,0	3,5	3,2	2,3	224,6	113,5
YPK_0071	periplasmic binding protein	2,82	1,7	2,0	1,9	1,3	129,3	65,3
YPK_1748	motB flagellar motor protein MotB	2,82	2,4	2,8	2,6	1,9	182,5	92,2
YPK_3855	replicative DNA helicase	2,82	2,4	2,8	2,6	1,9	182,5	92,2
YPK_0838	glutathione synthetase	2,81	1,7	2,0	1,9	1,3	128,5	64,9
YPK_1678	23S rRNA pseudouridylate synthase C	2,81	1,7	2,0	1,9	143,1	1,2	72,2
YPK_2728	putrescine ABC transporter membrane protein	2,80	1,7	2,0	1,9	1,4	127,7	64,5
YPK_4087	argininosuccinate lyase	2,80	2,5	2,9	2,7	201,2	1,7	101,5
YPK_4192	coproporphyrinogen III oxidase	2,80	2,5	2,9	2,7	201,2	1,7	101,5
YPK_2477	amino acid permease-associated protein	2,80	129,5	2,9	66,2	201,2	90,1	145,6
YPK_1856	multidrug efflux protein	2,80	2,5	2,9	2,7	1,9	180,2	91,0
YPK_1126	binding-protein-dependent transport system innerm	2,80	1,8	2,0	1,9	142,2	1,2	71,7
YPK_0313	trkA potassium transporter peripheral membranepı	2,80	2,5	2,9	2,7	200,8	1,7	101,2
YPK_3758	octaprenyl diphosphate synthase	2,80	1,8	2,0	1,9	1,4	127,0	64,2

YPK_0076	urocanate hydratase	2,79	3,0	3,5	3,3	2,4	219,7	111,1
YPK_3411	cytochrome-c peroxidase	2,79	2,5	2,9	2,7	1,9	179,4	90,7
YPK_3728	truB tRNA pseudouridine synthase B	2,79	1,8	2,1	1,9	1,4	126,6	64,0
YPK_0262	glycosyl transferase family protein	2,78	1,8	2,1	1,9	140,5	1,2	70,9
YPK_4021	putative transport protein YifK	2,78	2,5	2,9	2,7	1,9	177,8	89,9
YPK_3896	LysR family transcriptional regulator	2,78	1,8	2,1	1,9	1,4	125,4	63,4
YPK_0588	LacI family transcriptional regulator	2,77	1,8	2,1	1,9	1,4	125,0	63,2
YPK_2648	asnC asparaginyl-tRNA synthetase	2,77	2,5	2,9	2,7	197,3	1,8	99,6
YPK_3588	ileS isoleucyl-tRNA synthetase	2,77	5,1	73,9	39,5	98,5	176,4	137,4
YPK_2496	TRAP dicarboxylate transporter subunit DctP	2,76	1,8	2,1	1,9	138,8	1,2	70,0
YPK_4214	asparagine synthetase AsnA	2,76	1,8	2,1	1,9	138,8	1,2	70,0
YPK_1437	gltX glutamyl-tRNA synthetase	2,76	2,6	3,0	2,8	195,3	1,8	98,5
YPK_2014	NAD-dependent epimerase/dehydratase	2,76	1,8	2,1	2,0	138,0	1,3	69,6
YPK_2766	monosaccharide-transporting ATPase	2,76	1,8	2,1	2,0	138,0	1,3	69,6
YPK_3819	lysine 2,3-aminomutase YodO family protein	2,75	1,8	2,1	2,0	137,2	1,3	69,2
YPK_0563	NADH:flavin oxidoreductase	2,75	3,6	4,2	3,9	2,8	245,4	124,1
YPK_2564	mglC beta-methylgalactoside transporter innermer	2,74	1,8	2,1	2,0	136,4	1,3	68,8
YPK_1737	PIG3 family NAD(P)H quinone oxidoreductase	2,74	1,8	2,1	2,0	1,4	122,1	61,8
YPK_3184	group 1 glycosyl transferase	2,73	1,8	2,1	2,0	136,0	1,3	68,6
YPK_0779	LacI family transcriptional regulator	2,73	1,8	2,1	2,0	135,2	1,3	68,2
YPK_1463	extracellular solute-binding protein	2,73	1,8	2,1	2,0	1,4	121,0	61,2
YPK_1967	Na+/H+ antiporter NhaC	2,72	2,6	3,1	2,8	190,1	1,8	95,9
YPK_1302	inosine 5'-monophosphate dehydrogenase	2,71	2,6	3,1	2,9	2,0	169,2	85,6
YPK_0486	succinate-semialdehyde dehydrogenase	2,70	2,6	3,1	2,9	2,1	168,5	85,3
YPK_1164	cyclic-AMP phosphodiesterase	2,70	1,9	2,2	2,0	132,9	1,3	67,1
YPK_1841	phospho-2-dehydro-3-deoxyheptonate aldolase	2,69	1,9	2,2	2,0	1,5	117,9	59,7
YPK_1757	chemotaxis-specific methylesterase	2,68	1,9	2,2	2,1	1,5	117,6	59,5
YPK_3809	ribosome-associated GTPase	2,68	1,9	2,2	2,1	131,0	1,3	66,1
YPK_3679	leucyl aminopeptidase	2,66	2,7	137,2	70,0	183,0	81,9	132,4
YPK_1668	hypothetical protein	2,66	1,9	2,2	2,1	129,2	1,3	65,2
YPK_3357	phospho-2-dehydro-3-deoxyheptonate aldolase	2,65	1,9	2,3	2,1	128,1	1,4	64,7
YPK_1986	mannose-6-phosphate isomerase	2,64	1,9	2,3	2,1	1,5	114,4	57,9
YPK_4033	undecaprenyl-phosphatealpha-N-acetylglucosaminy	2,64	2,0	2,3	2,1	127,4	1,4	64,4
YPK_1645	CRISPR-associated helicase Cas3 family protein	2,63	5,9	63,3	34,6	126,6	113,4	120,0
YPK_3208	hydrophobe/amphiphile efflux-1 (HAE1) familyprote	2,63	226,7	6,6	116,6	132,1	433,6	282,8
YPK_1954	hypothetical protein	2,62	2,8	3,3	3,0	2,2	159,4	80,8
YPK_0697	fimbrial protein	2,61	2,0	2,3	2,2	1,5	111,9	56,7
YPK_1844	hypothetical protein	2,61	2,0	2,3	2,2	1,5	111,9	56,7
YPK_1054	putative RNA 2'-O-ribose methyltransferase	2,61	2,0	2,3	2,2	124,6	1,4	63,0
YPK_3040	xylose isomerase domain-containing protein	2,61	2,0	2,3	2,2	124,6	1,4	63,0
YPK_1387	nitrate reductase catalytic subunit	2,61	4,5	5,2	4,8	3,5	249,0	126,3
YPK_2696	macrolide transporter subunit MacA	2,60	2,0	2,3	2,2	124,0	1,4	62,7
YPK_1303	guaA GMP synthase	2,60	2,8	3,3	3,1	2,2	157,0	79,6
YPK_2409	ABC transporter-like protein	2,60	2,8	3,3	3,1	2,2	157,0	79,6
YPK_1672	solA N-methyltryptophan oxidase	2,60	2,0	2,3	2,2	123,6	1,4	62,5
YPK_0586	ABC transporter-like protein	2,60	2,0	2,3	2,2	1,6	110,7	56,1
YPK_1661	mdoG glucan biosynthesis protein G	2,59	2,9	3,3	3,1	2,2	156,1	79,2
YPK_4018	putative uroporphyrinogen IIIC-methyltransferase	2,59	2,0	2,4	2,2	1,6	109,8	55,7
YPK_1799	ABC transporter-like protein	2,58	2,0	2,4	2,2	122,3	1,4	61,9
YPK_2849	nrdB ribonucleotide-diphosphate reductase subun	2,58	2,0	2,4	2,2	122,0	1,4	61,7
YPK_2811	baseplate J family protein	2,57	2,1	2,4	2,2	121,4	1,4	61,4
YPK_2848	ribonucleotide-diphosphate reductase subunitalpha	2,57	4,1	4,8	4,5	3,2	216,9	110,0
YPK_2571	S-(hydroxymethyl)glutathione dehydrogenase/class	2,57	2,1	2,4	2,2	121,1	1,4	61,2
YPK_3593	chaperone protein DnaJ	2,57	2,1	2,4	2,2	121,1	1,4	61,2
YPK_3128	integrase family protein	2,55	2,1	2,4	2,3	119,5	1,4	60,5
YPK_2841	major facilitator transporter	2,55	2,1	2,4	2,3	1,6	107,0	54,3
YPK_0533	aerobic respiration control sensor protein ArcB	2,54	4,2	4,9	4,5	237,3	2,9	120,1
YPK_3991	glpA sn-glycerol-3-phosphate dehydrogenase sub	2,53	3,0	3,5	3,2	2,3	149,7	76,0
YPK_2026	hypothetical protein	2,53	2,1	2,5	2,3	118,0	1,5	59,7

YPK_0013		hypothetical protein	2,53	3,0	3,5	3,2	166,8	2,1	84,5
YPK_4119		LysR family transcriptional regulator	2,52	2,1	2,5	2,3	1,7	104,8	53,3
YPK_0562		rRNA (guanine-N(2)-)-methyltransferase	2,51	2,1	2,5	2,3	116,2	1,5	58,9
YPK_1012		general secretion pathway protein L	2,50	2,1	2,5	2,3	115,9	1,5	58,7
YPK_3647		lipopolysaccharide heptosyltransferase III	2,50	2,2	2,5	2,3	1,7	103,5	52,6
YPK_1290		ribosomal RNA large subunit methyltransferase N	2,50	2,2	2,5	2,3	115,4	1,5	58,4
YPK_1070		1-deoxy-D-xylulose 5-phosphate reductoisomerase	2,50	2,2	2,5	2,3	1,7	103,3	52,5
YPK_2762		PTS system fructose-specific transporter subunitIIB	2,49	3,1	3,6	3,3	2,4	145,7	74,1
YPK_1589		2-succinyl-5-enolpyruvyl-6-hydroxy-3-cyclohexene-	2,49	3,1	3,6	3,3	2,4	145,5	73,9
YPK_1423		RND family efflux transporter MFP subunit	2,49	2,2	2,5	2,4	114,5	1,5	58,0
YPK_2792		bicyclomycin/multidrug efflux system protein	2,49	2,2	2,5	2,4	114,5	1,5	58,0
YPK_0162	malQ	4-alpha-glucanotransferase	2,49	3,8	4,4	4,1	2,9	177,5	90,2
YPK_1346		phosphate ABC transporter permease	2,48	3,1	3,6	3,3	161,6	2,1	81,9
YPK_3068		transposase mutator type	2,48	2,2	2,5	2,4	1,7	102,3	52,0
YPK_0096		pectate lyase	2,48	3,1	3,6	3,3	2,4	144,2	73,3
YPK_3550		type VI secretion protein IcmF	2,47	6,3	7,3	6,8	39,7	35,6	37,6
YPK_0922		ssDNA exonuclease RecJ	2,47	3,1	3,6	3,4	2,4	143,0	72,7
YPK_1413		hypothetical protein	2,46	2,2	2,6	2,4	1,7	100,3	51,0
YPK_1712		peptidase T	2,45	2,2	2,6	2,4	111,7	1,6	56,6
YPK_3081		alpha/beta hydrolase fold protein	2,44	3,2	3,7	3,4	2,5	140,5	71,5
YPK_0072		hypothetical protein	2,44	2,2	2,6	2,4	110,9	1,6	56,2
YPK_3222		putative multidrug transportermembrane ATP-bindir	2,44	3,2	3,7	3,4	2,5	140,3	71,4
YPK_1709		outer membrane-specific lipoprotein transportersub	2,44	2,3	2,6	2,4	110,7	1,6	56,1
YPK_1768		patatin	2,43	2,3	2,6	2,5	109,9	1,6	55,7
YPK_3514	ftsA	cell division protein FtsA	2,43	2,3	2,6	2,5	1,8	98,4	50,1
YPK_3287	proA	gamma-glutamyl phosphate reductase	2,42	2,3	2,6	2,5	109,6	1,6	55,6
YPK_0820		adenine DNA glycosylase	2,42	2,3	2,6	2,5	1,8	98,2	50,0
YPK_3791		exoribonuclease R	2,42	4,6	164,1	84,3	109,4	195,9	152,7
YPK_3998		ATP-dependent DNA helicase RecQ	2,42	3,2	3,8	3,5	2,5	138,2	70,4
YPK_0112		gluconate transporter	2,41	2,3	2,7	2,5	1,8	97,5	49,6
YPK_0577		putative adhesin	2,41	3,2	3,8	3,5	153,8	2,3	78,0
YPK_3532		AMP-dependent synthetase and ligase	2,41	3,2	3,8	3,5	153,3	2,3	77,8
YPK_3630		patatin	2,41	2,3	2,7	2,5	1,8	97,0	49,4
YPK_1979		putative voltage-gated ClC-type chloride channelClc	2,40	2,3	2,7	2,5	107,8	1,6	54,7
YPK_0098		C4-dicarboxylate transporter DctA	2,39	2,3	2,7	2,5	107,1	1,6	54,4
YPK_1555		citrate transporter	2,39	3,3	3,8	3,6	151,1	2,3	76,7
YPK_0553		d-galactonate transporter	2,37	2,4	2,7	2,5	105,9	1,6	53,8
YPK_4057		dihydroxy-acid dehydratase	2,37	3,3	3,9	3,6	2,6	134,0	68,3
YPK_0400		ImpA domain-containing protein	2,37	2,4	2,7	2,6	1,8	94,6	48,2
YPK_2015		major facilitator transporter	2,37	2,4	2,8	2,6	105,4	1,6	53,5
YPK_3253		1-deoxy-D-xylulose-5-phosphate synthase	2,37	3,3	3,9	3,6	148,9	2,3	75,6
YPK_4091		phosphoenolpyruvate carboxylase	2,36	4,7	5,5	5,1	3,7	188,4	96,0
YPK_2526	hisD	histidinol dehydrogenase	2,34	2,4	2,8	2,6	1,9	92,9	47,4
YPK_4217		tRNA uridine 5-carboxymethylaminomethylmodifica	2,34	3,4	4,0	3,7	2,6	131,2	66,9
YPK_1332		multidrug efflux system subunit MdtA	2,34	2,4	2,8	2,6	103,5	1,7	52,6
YPK_1259		two component, sigma54 specific, Fis familytranscri	2,34	2,4	2,8	2,6	103,3	1,7	52,5
YPK_3377		major facilitator transporter	2,33	2,4	2,8	2,6	103,1	1,7	52,4
YPK_3359		anaerobic C4-dicarboxylate transporter	2,33	2,4	2,8	2,6	102,8	1,7	52,3
YPK_0464		regulatory protein CsrD	2,32	3,4	4,0	3,7	144,5	2,4	73,5
YPK_2396		flagellar hook-length control protein	2,32	2,4	2,8	2,6	1,9	91,5	46,7
YPK_3305		Na(+)-translocating NADH-quinone reductasesubur	2,31	2,5	2,9	2,7	1,9	90,9	46,4
YPK_2016		exoribonuclease II	2,31	3,5	4,1	3,8	2,7	128,2	65,4
YPK_2100		putative serine protein kinase PrkA	2,31	3,5	4,1	3,8	2,7	128,2	65,4
YPK_4045		hypothetical protein	2,31	2,5	2,9	2,7	1,9	90,5	46,2
YPK_1718		adenylosuccinate lyase	2,30	2,5	2,9	2,7	1,9	90,3	46,1
YPK_3048		thiamine pyrophosphate binding domain-containing	2,30	3,5	4,1	3,8	142,3	2,4	72,4
YPK_4133	cpxA	two-component sensor protein	2,30	2,5	2,9	2,7	1,9	89,9	45,9
YPK_1084	tilS	tRNA(Ile)-lysidine synthetase	2,29	2,5	2,9	2,7	99,9	1,7	50,8
YPK_0004	gyrB	DNA gyrase subunit B	2,28	4,3	5,1	4,7	172,2	3,0	87,6

YPK_3198	DNA polymerase III subunits gamma and tau	2,28	3,6	4,1	3,8	2,8	125,5	64,1
YPK_1898	hypothetical protein	2,28	2,5	2,9	2,7	98,9	1,8	50,3
YPK_4173	putative transcriptional regulator	2,27	3,6	4,2	3,9	2,8	124,5	63,7
YPK_3353	sigma 54 modulation protein/30S ribosomalprotein S	2,26	3844,6	1679,8	2762,2	13064,3	11363,6	12213,9
YPK_4191	glnG nitrogen regulation protein NR(I)	2,26	2,5	3,0	2,8	97,8	1,8	49,8
YPK_2993	outer membrane porin	2,25	2,6	3,0	2,8	97,2	1,8	49,5
YPK_2240	hmsF outer membrane N-deacetylase	2,25	3,6	4,2	3,9	2,8	122,7	62,8
YPK_2805	glycoside hydrolase family protein	2,24	2,6	3,0	2,8	2,0	86,2	44,1
YPK_3458	chloride channel protein	2,24	2,6	3,0	2,8	2,0	86,2	44,1
YPK_3165	copA copper exporting ATPase	2,23	5,2	6,0	5,6	4,0	172,2	88,1
YPK_0600	Na+/H+ antiporter NhaC	2,22	2,6	3,0	2,8	95,2	1,8	48,5
YPK_1519	mnmC 5-methylaminomethyl-2-thiouridinemethyltr	2,21	3,7	4,3	4,0	2,9	119,9	61,4
YPK_4081	dihydrolipoamide dehydrogenase	2,21	2,6	3,1	2,9	94,5	1,8	48,1
YPK_4180	ATP-dependent DNA helicase RecG	2,20	3,7	4,4	4,1	2,9	119,2	61,0
YPK_0469	ribonuclease G	2,20	2,6	3,1	2,9	2,1	84,2	43,1
YPK_1677	rne ribonuclease E	2,19	49,0	7,6	28,3	76,1	102,2	89,1
YPK_0556	altronate dehydratase	2,18	2,7	3,1	2,9	2,1	83,1	42,6
YPK_2001	L-arabinose isomerase	2,17	2,7	3,2	2,9	92,0	1,9	47,0
YPK_3946	hypothetical protein	2,17	2,7	3,2	2,9	91,8	1,9	46,9
YPK_0178	intracellular growth attenuator IgaA	2,16	3,9	4,5	4,2	3,0	115,5	59,3
YPK_3453	dgt deoxyguanosinetriphosphate triphosphohydrol	2,15	2,7	3,2	3,0	90,9	1,9	46,4
YPK_3936	3-octaprenyl-4-hydroxybenzoate carboxy-lyase	2,15	2,7	3,2	3,0	90,9	1,9	46,4
YPK_3343	N-acetyltransferase GCN5	2,15	4,7	5,5	5,1	157,4	3,3	80,4
YPK_2835	putative transport protein	2,15	2,7	3,2	3,0	2,1	81,1	41,6
YPK_3009	lnt apolipoprotein N-acyltransferase	2,13	2,8	3,2	3,0	2,2	80,0	41,1
YPK_0748	AbgT transporter	2,12	2,8	3,3	3,0	88,8	1,9	45,4
YPK_1181	L-aspartate oxidase	2,05	2,9	3,4	3,2	84,5	2,1	43,3
YPK_0711	fliF flagellar MS-ring protein	2,05	3,0	3,4	3,2	2,3	75,5	38,9
YPK_1152	phosphoglucomutase	2,04	3,0	3,4	3,2	84,2	2,1	43,1
YPK_1892	extracellular solute-binding protein	2,04	3,0	3,4	3,2	84,2	2,1	43,1
YPK_1835	arnT 4-amino-4-deoxy-L-arabinose transferase	2,02	3,0	3,5	3,2	2,3	74,4	38,4
YPK_1753	methyl-accepting chemotaxis sensory transducer	2,02	3,0	3,5	3,3	2,3	74,0	38,2
YPK_0170	RNA-binding S1 domain-containing protein	2,01	4,3	5,0	4,6	3,3	104,5	53,9
YPK_3394	anaerobic dimethyl sulfoxide reductase subunitA	1,97	4,4	5,1	4,8	113,1	3,1	58,1
YPK_0802	OmpA/MotB domain-containing protein	1,96	3,1	3,6	3,4	2,4	71,4	36,9
YPK_3770	surface antigen (D15)	1,96	3,1	3,6	3,4	2,4	71,4	36,9
YPK_1742	methyl-accepting chemotaxis sensory transducer	1,96	3,1	3,6	3,4	79,6	2,2	40,9
YPK_2051	sulfate transporter	1,96	3,1	3,6	3,4	79,6	2,2	40,9
YPK_1845	FAD linked oxidase domain-containing protein	1,94	5,5	6,4	5,9	136,2	3,8	70,0
YPK_3271	note_PFAM:_alpha_amylase_catalytic_region,SMAF	1,89	3,3	3,8	3,6	2,6	67,8	35,2
YPK_3311	putative accessory processing protein	1,87	3,3	3,9	3,6	2,6	66,9	34,7
YPK_2654	hypothetical protein	1,87	3,3	3,9	3,6	74,6	2,3	38,4
YPK_4211	trkD potassium transport protein Kup	1,86	3,4	3,9	3,6	2,6	66,3	34,5
YPK_0659	DNA topoisomerase IV subunit B	1,84	3,4	4,0	3,7	2,6	65,4	34,0
YPK_1744	magnesium-translocating P-type ATPase	1,83	4,8	5,7	5,3	102,8	3,4	53,1
YPK_2640	ABC transporter ATPase	1,82	3,4	4,0	3,7	2,7	64,8	33,7
YPK_3802	N-acetylmuramoyl-L-alanine amidase	1,82	3,4	4,0	3,7	2,7	64,8	33,7
YPK_1823	rplT 50S ribosomal protein L20	1,77	976,9	569,1	773,0	3793,7	1698,4	2746,1
YPK_2645	pepN aminopeptidase N	1,77	68,3	79,5	73,9	265,1	237,4	251,2
YPK_3831	putative oxidoreductase Fe-S binding subunit	1,75	3,6	4,2	3,9	2,8	61,5	32,2
YPK_1422	ABC transporter-like protein	1,74	3,7	4,3	4,0	2,8	60,9	31,9
YPK_3144	SMC (structural maintenance of chromosomes)fam	1,73	3,7	4,3	4,0	2,9	60,5	31,7
YPK_1368	hypothetical protein	1,72	3,7	4,3	4,0	2,9	60,5	31,7
YPK_2442	oligopeptidase B	1,72	3,7	4,3	4,0	2,9	60,5	31,7
YPK_2630	outer membrane protein A	1,71	2341,0	2337,9	2339,4	7143,2	8256,8	7700,0
YPK_2017	carbon starvation protein CstA	1,71	3,7	4,3	4,0	67,0	2,6	34,8
YPK_2493	glycoside hydrolase clan GH-D	1,67	3,8	4,5	4,1	3,0	58,3	30,7
YPK_2002	type I secretion system ATPase	1,67	3,8	4,5	4,2	64,8	2,7	33,7
YPK_2627	hypothetical protein	1,66	3,8	4,5	4,2	3,0	58,0	30,5

YPK_1027	bifunctional acyl-[acyl carrier protein]synthetase/2-a	1,65	3,9	4,5	4,2	3,0	57,5	30,3
YPK_1345	binding-protein-dependent transport system innerm	1,65	3,9	4,5	4,2	3,0	57,4	30,2
YPK_2207	Sel1 domain-containing protein	1,65	3,9	4,5	4,2	3,0	57,4	30,2
YPK_1212	virulence-associated E family protein	1,64	3,9	4,6	4,2	63,6	2,7	33,2
YPK_2856	dinG ATP-dependent DNA helicase DinG	1,64	3,9	4,6	4,2	63,5	2,7	33,1
YPK_0094	biofilm formation regulator HmsP	1,63	3,9	4,6	4,3	63,4	2,7	33,1
YPK_3426	nlpD lipoprotein NlpD	1,61	541,0	210,1	375,6	700,3	1505,0	1102,7
YPK_2667	ihfB integration host factor subunit beta	1,61	1822,9	1416,0	1619,4	4719,6	5071,1	4895,4
YPK_3769	hypothetical protein	1,60	7,0	8,1	7,5	107,5	4,8	56,2
YPK_0470	hypothetical protein	1,59	7,0	8,2	7,6	106,2	4,9	55,5
YPK_0662	DNA topoisomerase IV subunit A	1,58	4,1	4,8	4,4	3,2	54,6	28,9
YPK_0583	outer membrane autotransporter	1,57	4,1	4,8	4,4	3,2	54,3	28,7
YPK_2098	MltA-interacting MipA family protein	1,53	229,5	267,4	248,5	356,5	1436,6	896,6
YPK_1041	recC exonuclease V subunit gamma	1,51	6,1	7,1	6,6	4,7	73,7	39,2
YPK_3072	glycoside hydrolase family 3	1,51	4,3	5,0	4,6	58,2	3,0	30,6
YPK_1288	beta and gamma crystallin	1,49	4,4	5,1	4,7	3,4	51,3	27,3
YPK_1456	anaerobic dimethyl sulfoxide reductase subunitA	1,48	4,4	5,1	4,7	3,4	51,2	27,3
YPK_0370		1,46	4,4	5,2	4,8	3,4	50,4	26,9
YPK_2451	L-serine dehydratase 1	1,45	130,4	151,9	141,1	405,0	362,7	383,8
YPK_0052	fimbrial biogenesis outer membrane usherprotein	1,44	4,5	5,2	4,9	55,5	3,1	29,3
YPK_0219	mrcA peptidoglycan synthetase	1,41	4,6	5,4	5,0	54,3	3,2	28,7
YPK_2274	fimbrial biogenesis outer membrane usherprotein	1,39	4,7	5,4	5,0	53,5	3,2	28,4
YPK_0341	DNA-directed RNA polymerase subunit beta	1,37	254,2	641,7	448,0	756,9	1443,8	1100,3
YPK_0804	type VI secretion ATPase	1,36	4,7	5,5	5,1	3,7	47,0	25,3
YPK_2074	global DNA-binding transcriptional dualregulator H-I	1,35	428,8	999,3	714,1	2331,6	1193,0	1762,3
YPK_0354	transcriptional regulator HU subunit alpha	1,24	626,8	1460,5	1043,6	5841,7	871,8	3356,8
YPK_4118	tpiA triosephosphate isomerase	1,24	230,4	536,9	383,6	715,8	961,4	838,6
YPK_4225	F0F1 ATP synthase subunit gamma	1,24	205,1	239,0	222,0	159,3	1711,7	935,5
YPK_0295	rplE 50S ribosomal protein L5	1,24	977,7	379,7	678,7	759,4	2720,0	1739,7
YPK_2677	formate acetyltransferase	1,24	547,2	91,1	319,1	364,3	761,2	562,7
YPK_3547	ATP-dependent helicase HepA	1,22	5,2	6,1	5,7	47,7	3,6	25,7
YPK_3267	alkyl hydroperoxide reductase	1,21	1169,7	2725,7	1947,7	3634,0	4677,5	4155,8
YPK_3152	cysS cysteinyl-tRNA synthetase	1,15	7319,0	4488,3	5903,7	13264,5	12145,1	12704,8
YPK_1001	carbonic anhydrase	1,14	468,0	272,7	370,3	1272,3	488,2	880,3
YPK_3642	acriflavin resistance protein	1,14	5,5	6,5	6,0	4,3	40,3	22,3
YPK_2955	peptidoglycan-associated outer membranelipoprote	1,11	2773,7	1615,9	2194,8	4578,1	4581,5	4579,8
YPK_2566	periplasmic binding protein/LacI transcriptionalregul	1,11	714,9	208,2	461,5	1110,5	621,5	866,0
YPK_0505	RNA polymerase factor sigma-54	1,11	248,3	144,6	196,5	385,7	431,7	408,7
YPK_3446	eno phosphopyruvate hydratase	1,10	960,8	799,6	880,2	1279,3	2768,2	2023,8
YPK_3825	aspA aspartate ammonia-lyase	1,10	1610,4	1154,7	1382,6	2020,6	4221,6	3121,1
YPK_0107	outer membrane autotransporter	1,08	5,8	6,7	6,2	43,2	4,0	23,6
YPK_3147	hypothetical protein	1,05	5,9	6,8	6,4	4,6	38,0	21,3
YPK_0547	hypothetical protein	1,03	1134,5	3304,6	2219,6	4405,9	3550,6	3978,3
YPK_3726	polynucleotide phosphorylase/polyadenylase	1,03	168,5	98,1	133,3	196,3	351,5	273,9
YPK_3242	cytochrome o ubiquinol oxidase subunit I	1,03	179,1	104,3	141,7	139,1	560,4	349,8
YPK_1854	LPP repeat-containing protein	1,01	34108,8	36359,1	35233,9	73841,5	68137,1	70989,3
YPK_0332	elongation factor Tu	0,97	1050,0	1048,6	1049,3	1165,1	3651,3	2408,2
YPK_0634	RNA polymerase sigma factor RpoD	0,95	96,9	225,9	161,4	301,2	269,7	285,4
YPK_0289	rpsC 30S ribosomal protein S3	0,90	1011,3	294,6	652,9	392,7	2637,5	1515,1
YPK_0296	rpsN 30S ribosomal protein S14	0,85	567,3	0,7	284,0	0,4	2761,6	1381,0
YPK_0340	rpoB DNA-directed RNA polymerase subunit beta	0,84	310,7	310,3	310,5	344,7	895,1	619,9
YPK_1485	type VI secretion protein IcmF	0,83	6,9	8,0	7,4	5,3	32,5	18,9
YPK_1253	phosphoribosylformylglycinamidine synthase	0,80	7,0	8,1	7,6	5,4	32,0	18,7
YPK_1821	translation initiation factor IF-3	0,80	1489,6	2892,7	2191,2	5399,4	2417,3	3908,4
YPK_0869	glycine dehydrogenase	0,74	186,1	72,3	129,2	96,4	388,2	242,3
YPK_2997	nagB glucosamine-6-phosphate deaminase	0,74	221,0	1,7	111,4	1,1	922,3	461,7
YPK_4059	branched-chain amino acid aminotransferase	-0,85	1,7	668,7	335,2	297,2	1,2	149,2
pYV0072	yscS, type III secretion protein	-0,85	1941,4	0,6	971,0	1005,3	0,3	502,8
YPK_1360	bcp thioredoxin-dependent thiol peroxidase	-0,85	0,9	1302,6	651,7	0,7	518,4	259,5

YPK_0160	transcriptional regulator MalT	-0,85	197,6	5,7	101,6	3,8	91,6	47,7
YPK_1073	zinc metallopeptidase RseP	-0,85	393,7	2,8	198,3	1,9	182,5	92,2
YPK_4005	hypothetical protein	-0,85	432,6	2,6	217,6	1,7	200,6	101,2
YPK_4079	DNA-binding transcriptional regulator OxyR	-0,85	579,5	1,9	290,7	1,3	268,7	135,0
YPK_3424	DNA mismatch repair protein MutS	-0,85	209,6	5,4	107,5	3,6	97,2	50,4
YPK_3921	acetyl-CoA synthetase	-1,00	546,2	4,1	275,2	70,7	126,6	98,7
YPK_0853	fructose-bisphosphate aldolase	-1,02	1644,5	2299,3	1971,9	894,1	1029,3	961,7
pYV0061	yscY, type III secretion protein	-1,05	0,6	1176,6	588,6	392,2	0,4	196,3
YPK_2135	DNA-binding transcriptional regulator HexR	-1,05	1,6	456,0	228,8	152,0	1,1	76,6
YPK_3298	hypothetical protein	-1,05	0,4	1755,1	877,7	585,0	0,3	292,6
YPK_3881	hypothetical protein	-1,05	1,9	383,2	192,6	127,7	1,4	64,5
YPK_1782	putative solute/DNA competence effector	-1,05	495,2	1,5	248,3	192,3	0,9	96,6
YPK_1192	pyridoxine 5'-phosphate synthase	-1,05	483,2	1,5	242,4	187,6	0,9	94,3
YPK_1034	fused phosphoenolpyruvate-proteinphosphotransfe	-1,05	158,8	4,7	81,8	61,7	2,8	32,2
YPK_1925	ATP-dependent RNA helicase HrpA	-1,05	92,0	8,1	50,1	35,7	4,9	20,3
YPK_1150	hypothetical protein	-1,05	945,9	0,8	473,4	367,4	0,5	183,9
YPK_0849	peptidase M48 Ste24p	-1,05	469,9	1,6	235,7	182,5	0,9	91,7
YPK_2537	UTP-glucose-1-phosphate uridylyltransferase	-1,05	397,9	1,9	199,9	154,5	1,1	77,8
YPK_1910	hypothetical protein	-1,05	353,2	2,1	177,7	137,2	1,3	69,2
YPK_1995	putative oxidoreductase	-1,05	338,2	2,2	170,2	131,3	1,3	66,3
YPK_2998	nagA N-acetylglucosamine-6-phosphate deacetyla	-1,05	310,1	2,4	156,3	120,4	1,4	60,9
YPK_3104	hypothetical protein	-1,05	947,2	275,9	611,5	367,8	164,7	266,3
YPK_4126	6-phosphofructokinase	-1,05	360,7	840,5	600,6	280,1	250,8	265,5
YPK_1076	lpxD UDP-3-O-[3-hydroxymyristoyl] glucosamineN	-1,05	347,1	404,4	375,7	134,8	241,4	188,1
YPK_0043	selenocysteinyl-tRNA-specific translationfactor	-1,05	3,5	210,5	107,0	2,8	62,8	32,8
YPK_0271	YheO domain-containing protein	-1,05	1,3	569,9	285,6	1,0	170,1	85,5
YPK_3479	hypoxanthine phosphoribosyltransferase	-1,05	1,0	751,3	376,1	0,8	224,2	112,5
YPK_3584	dihydrodipicolinate reductase	-1,05	1,5	502,1	251,8	1,2	149,8	75,5
YPK_2139	hypothetical protein	-1,05	2,4	314,8	158,6	1,8	93,9	47,9
YPK_2650	aromatic amino acid aminotransferase	-1,05	2,1	347,8	175,0	1,7	103,8	52,7
YPK_0654	tolC outer membrane channel protein	-1,05	255,2	2,9	129,0	2,0	88,7	45,3
YPK_4044	fimbrial biogenesis outer membrane usherprotein	-1,05	134,7	5,6	70,1	3,7	46,8	25,3
YPK_1610	alcohol dehydrogenase	-1,05	289,8	2,6	146,2	1,7	100,8	51,2
YPK_3766	fructose-1	-1,05	317,5	2,4	159,9	1,6	110,4	56,0
YPK_1548	putative PTS IIA-like nitrogen-regulatoryprotein PtsI	-1,05	789,7	0,9	395,3	0,6	274,6	137,6
YPK_0151	glycogen/starch/alpha-glucan phosphorylase	-1,05	583,4	5,1	294,3	3,4	202,9	103,1
YPK_1297	engA GTP-binding protein EngA	-1,05	239,3	3,1	121,2	2,1	83,2	42,7
YPK_0039	formate dehydrogenase subunit beta	-1,05	365,1	2,0	183,6	1,4	127,0	64,2
YPK_3810	psd phosphatidylserine decarboxylase	-1,05	401,9	1,9	201,9	1,2	139,8	70,5
YPK_2685	cell division protein FtsK	-1,05	90,9	8,2	49,6	5,5	31,6	18,5
YPK_0101	ribokinase-like domain-containing protein	-1,05	375,4	2,0	188,7	1,3	130,5	65,9
YPK_2150	hypothetical protein	-1,05	883,0	0,8	441,9	0,6	307,1	153,8
YPK_2096	glyceraldehyde-3-phosphate dehydrogenase	-1,09	2316,4	1868,5	2092,4	553,6	1734,9	1144,2
YPK_0825	virulence determinant	-1,15	38,4	19,5	28,9	13,0	11,6	12,3
YPK_0335	rplK 50S ribosomal protein L11	-1,22	1225,0	2378,7	1801,9	634,3	851,9	743,1
YPK_1903	tpx thiol peroxidase	-1,23	2789,9	1,1	1395,5	541,7	485,1	513,4
YPK_2967	dihydrolipoamide succinyltransferase	-1,24	1307,4	677,0	992,2	225,7	707,2	466,4
YPK_3739	greA transcription elongation factor GreA	-1,35	0,9	1286,6	643,7	285,9	0,6	143,2
YPK_1066	rpsB 30S ribosomal protein S2	-1,35	1461,3	567,5	1014,4	756,7	169,4	463,0
YPK_3873	stress protein	-1,35	304,3	1063,8	684,1	236,4	211,7	224,0
YPK_4104	hslU ATP-dependent protease ATP-binding subuni	-1,35	400,7	311,3	356,0	207,5	92,9	150,2
YPK_3862	glycerol-3-phosphate acyltransferase	-1,35	4,5	250,9	127,7	3,5	49,9	26,7
YPK_1881	tyrosyl-tRNA synthetase	-1,35	2,3	975,2	488,7	1,8	194,0	97,9
YPK_1561	NADH dehydrogenase subunit B	-1,35	1,2	910,7	456,0	1,0	181,2	91,1
YPK_1363	lipoprotein	-1,35	503,0	2,2	252,6	1,5	116,6	59,1
YPK_0558	serine/threonine transporter SstT	-1,36	539,1	2,8	270,9	104,7	93,7	99,2
YPK_2966	sucC succinyl-CoA synthetase subunit beta	-1,40	761,5	1597,0	1179,2	236,6	741,4	489,0
YPK_2996	PTS system N-acetylglucosamine-specifictransporte	-1,51	438,5	306,5	372,5	68,1	244,0	156,0
YPK_2973	gltA type II citrate synthase	-1,55	2,3	647,1	324,7	107,8	1,6	54,7

YPK_2118	hypothetical protein	-1,55	1075,0	1,4	538,2	208,7	0,8	104,8
YPK_1783	carboxy-terminal protease	-1,55	343,2	100,0	221,6	66,6	59,7	63,2
pYV0005	replication protein	-1,55	1,6	952,6	477,1	1,2	142,2	71,7
YPK_1042	peptidase M16 domain-containing protein	-1,64	556,5	6,0	281,3	4,0	86,0	45,0
YPK_0564	acid-resistance membrane protein	-1,69	1854,3	1440,4	1647,4	240,0	1074,7	657,4
YPK_0478	virulence plasmid 65kDa B protein	-1,71	40,0	9,3	24,7	6,2	5,6	5,9
YPK_1184	rseB periplasmic negative regulator of sigmaE	-1,72	370,7	1079,9	725,3	288,0	128,9	208,4
YPK_3599	transaldolase B	-1,72	929,7	1949,9	1439,8	433,3	388,0	410,6
YPK_2655	mukB cell division protein MukB	-1,72	40,1	9,3	24,7	6,2	5,6	5,9
YPK_1043	recB exonuclease V subunit beta	-2,00	48,8	7,7	28,2	5,1	4,6	4,8
YPK_3582	carB carbamoyl phosphate synthase large subunit	-2,18	5,8	64,4	35,1	4,5	4,0	4,3
YPK_1739	lacZ beta-D-galactosidase	-2,19	55,8	6,7	31,3	4,5	4,0	4,2
YPK_1268	virulence-related outer membrane protein	-2,23	28354,5	27720,5	28037,5	3037,7	11786,6	7412,1
YPK_2350	TP901 family phage tail tape measure protein	-2,33	5,2	71,4	38,3	4,1	3,6	3,9
YPK_2270		-2,37	7,2	104,2	55,7	5,6	5,0	5,3
YPK_2030	aconitate hydratase	-2,45	4,8	77,8	41,3	3,7	3,3	3,5
pYV0067	type III secretion system ATPase	-2,47	2291,2	1570,3	1930,8	418,7	281,2	350,0
YPK_0124	multi-sensor hybrid histidine kinase	-2,52	4,6	81,4	43,0	3,6	3,2	3,4
YPK_2574	hypothetical protein	-2,63	75,6	4,9	40,3	3,3	3,0	3,1
YPK_2839	outer membrane porin protein C	-2,73	2547,1	3524,2	3035,6	123,6	1660,7	892,2
YPK_0190	hypothetical protein	-2,73	4,0	94,2	49,1	3,1	2,8	2,9
YPK_1117	ribonucleotide-diphosphate reductase subunitalpha	-2,77	82,9	4,5	43,7	3,0	2,7	2,8
YPK_2033	DNA topoisomerase I	-2,78	204,8	79,5	142,2	106,0	3,3	54,7
YPK_0702	flhA flagellar biosynthesis protein FlhA	-2,80	85,2	4,4	44,8	2,9	2,6	2,8
YPK_0023	glyS glycyl-tRNA synthetase subunit beta	-2,82	3,7	100,4	52,1	2,9	2,6	2,7
YPK_3711	heparinase II/III family protein	-2,86	3,6	102,9	53,3	2,8	2,5	2,7
YPK_0473	YD repeat-containing protein	-2,86	5,1	145,6	75,4	4,0	3,6	3,8
YPK_0644	bifunctional glutamine-synthetaseadenylyltransferas	-2,86	5,1	145,7	75,4	4,0	3,6	3,8
YPK_1044	recD exonuclease V subunit alpha	-2,89	90,3	4,1	47,2	2,8	2,5	2,6
YPK_2615	cytotoxic necrotizing factor	-2,90	2523,0	1230,6	1876,8	227,9	244,8	236,4
YPK_1280	hscA chaperone protein HscA	-2,93	93,0	4,0	48,5	2,7	2,4	2,5
YPK_0595	fusaric acid resistance protein region	-2,94	3,4	109,2	56,3	2,7	2,4	2,5
YPK_3666	Type I site-specific deoxyribonuclease	-2,97	135,0	5,5	70,3	3,7	3,3	3,5
YPK_1376	binding-protein-dependent transport system innerm	-3,05	3,2	117,3	60,3	2,5	2,2	2,3
YPK_1091	prolyl-tRNA synthetase	-3,09	103,7	3,6	53,6	2,4	2,2	2,3
YPK_3175	putative cation:proton antiport protein	-3,11	3,0	122,7	62,9	2,4	2,1	2,2
YPK_1843	phosphoenolpyruvate synthase	-3,12	149,7	174,4	162,0	116,3	3,0	59,6
YPK_3204	potassium efflux protein KefA	-3,12	212,6	7,0	109,8	4,7	4,2	4,4
YPK_1950	acetolactate synthase catalytic subunit	-3,13	3,0	124,5	63,7	2,3	2,1	2,2
YPK_2699	hypothetical protein	-3,13	106,8	3,5	55,2	2,3	2,1	2,2
YPK_3232	DNA-binding ATP-dependent protease La	-3,14	151,6	4,9	78,3	3,3	2,9	3,1
YPK_1947	hypothetical protein	-3,15	4,2	178,2	91,2	3,3	2,9	3,1
YPK_1421	FAD-dependent pyridine nucleotide-disulfideoxidore	-3,15	3,0	126,0	64,5	2,3	2,1	2,2
YPK_3447	pyrG CTP synthetase	-3,16	108,8	3,4	56,1	2,3	2,1	2,2
YPK_1222	hypothetical protein	-3,19	111,0	3,4	57,2	2,2	2,0	2,1
YPK_3633	prfC peptide chain release factor 3	-3,20	112,0	3,3	57,7	2,2	2,0	2,1
YPK_3650	ABC transporter-like protein	-3,21	2,9	131,0	66,9	2,2	2,0	2,1
YPK_0377	malF maltose transporter membrane protein	-3,21	2,8	131,5	67,2	2,2	2,0	2,1
YPK_1407	putative sialic acid transporter	-3,25	2,8	135,4	69,1	2,1	1,9	2,0
YPK_1571	NADH dehydrogenase subunit M	-3,25	2,8	135,4	69,1	2,1	1,9	2,0
YPK_3385	ABC transporter-like protein	-3,26	2,7	136,1	69,4	2,1	1,9	2,0
YPK_0966	ABC transporter-like protein	-3,27	118,0	3,2	60,6	2,1	1,9	2,0
YPK_2008	electron transport complex protein RnfC	-3,30	3,8	198,2	101,0	2,9	2,6	2,8
YPK_1933		-3,30	120,3	140,2	130,3	46,7	3,7	25,2
YPK_1420	Na+/solute symporter	-3,32	121,9	284,0	202,9	2,0	169,5	85,8
YPK_1344	polyphosphate kinase	-3,33	3,7	201,4	102,6	2,9	2,6	2,7
pYV0024	sycE, yerA, yopE chaperone	-3,35	2668,8	4146,0	3407,4	345,5	309,3	327,4
YPK_0150	glgA glycogen synthase	-3,35	2,6	144,9	73,8	2,0	1,8	1,9
YPK_0614	integrase family protein	-3,35	2,6	144,9	73,8	2,0	1,8	1,9

YPK_2361		D-alanine/D-serine/glycine permease	-3,36	124,9	3,0	64,0	2,0	1,8	1,9
YPK_4207		major facilitator transporter	-3,36	124,9	3,0	64,0	2,0	1,8	1,9
YPK_0495		PTS system trehalose(maltose)-specifictransporter	-3,37	125,7	3,0	64,3	2,0	1,8	1,9
YPK_2748		putative outer membrane receptor	-3,37	3,6	208,0	105,8	2,8	2,5	2,6
YPK_3717		RND efflux system outer membrane lipoprotein	-3,38	126,5	3,0	64,7	2,0	1,8	1,9
YPK_2446		ATP-dependent RNA helicase DbpA	-3,40	128,7	2,9	65,8	1,9	1,7	1,8
YPK_4228	glmU	bifunctional N-acetylglucosamine-1-phospha	-3,41	129,8	2,9	66,3	1,9	1,7	1,8
YPK_4023		putative common antigen polymerase	-3,42	2,5	151,9	77,2	1,9	1,7	1,8
YPK_0264		putative ABC transporter ATP-binding protein	-3,43	185,5	4,0	94,7	2,7	2,4	2,5
YPK_3145		hypothetical protein	-3,43	185,5	4,0	94,7	2,7	2,4	2,5
YPK_0863		proline aminopeptidase P II	-3,47	2,4	157,7	80,1	1,8	1,6	1,7
YPK_3570		organic solvent tolerance protein	-3,49	4,0	276,9	140,5	3,1	2,8	3,0
YPK_3487		bifunctional aconitate hydratase2/2-methylisocitrate	-3,49	343,7	640,7	492,2	3,6	478,0	240,8
YPK_3219		ammonium transporter	-3,50	137,6	2,7	70,1	1,8	1,6	1,7
YPK_1353		uracil transporter	-3,50	138,2	2,7	70,5	1,8	1,6	1,7
YPK_2636		3-oxoacyl-(acyl carrier protein) synthase II	-3,52	139,5	2,7	71,1	1,8	1,6	1,7
YPK_0685		type II and III secretion system protein	-3,52	139,8	162,9	151,4	1,8	97,2	49,5
YPK_4162		integrase family protein	-3,52	2,3	163,3	82,8	1,8	1,6	1,7
YPK_0859		D-3-phosphoglycerate dehydrogenase	-3,55	143,2	2,6	72,9	1,7	1,6	1,6
YPK_0427		transposase mutator type	-3,59	2,2	171,3	86,8	1,7	1,5	1,6
YPK_1707		outer membrane-specific lipoprotein transportersub	-3,60	2,2	172,2	87,2	1,7	1,5	1,6
YPK_1565		NADH dehydrogenase subunit G	-3,61	195,2	530,7	362,9	3,8	181,0	92,4
YPK_2620		PUA domain-containing protein	-3,61	149,3	2,5	75,9	1,7	1,5	1,6
YPK_3876		stress protein	-3,62	150,0	2,5	76,2	1,7	1,5	1,6
YPK_0440		hypothetical protein	-3,63	151,1	2,5	76,8	1,6	1,5	1,6
YPK_2751		periplasmic binding protein	-3,64	151,9	2,5	77,2	1,6	1,5	1,6
YPK_3705		hypothetical protein	-3,64	2,1	177,4	89,8	1,6	1,5	1,5
YPK_0852	pgk	phosphoglycerate kinase	-3,65	152,7	1423,2	787,9	1,6	849,5	425,6
YPK_0651		glutathionylspermidine synthase	-3,65	153,1	2,4	77,8	1,6	1,5	1,5
YPK_3924		hypothetical protein	-3,65	153,5	2,4	78,0	1,6	1,5	1,5
YPK_3177		ferric enterobactin transport protein FepE	-3,66	2,1	179,7	90,9	1,6	1,4	1,5
YPK_0519		serine endoprotease	-3,66	154,3	2,4	78,3	1,6	1,4	1,5
YPK_4031		UDP-N-acetylglucosamine 2-epimerase	-3,69	2,0	183,0	92,5	1,6	1,4	1,5
YPK_2549		integral membrane protein TerC	-3,70	2,9	261,6	132,2	2,2	2,0	2,1
YPK_3421		alcohol dehydrogenase	-3,71	159,2	2,3	80,8	1,6	1,4	1,5
YPK_1878	anmK	anhydro-N-acetylmuramic acid kinase	-3,71	2,0	186,0	94,0	1,6	1,4	1,5
YPK_1292		cytoskeletal protein RodZ	-3,72	2,0	187,0	94,5	1,6	1,4	1,5
YPK_0967		ABC transporter-like protein	-3,72	160,5	2,3	81,4	1,6	1,4	1,5
YPK_2547		pyridoxal-dependent decarboxylase	-3,74	2,8	268,1	135,4	2,2	1,9	2,1
YPK_0226	aroB	3-dehydroquinate synthase	-3,74	163,1	2,3	82,7	1,5	1,4	1,4
YPK_2108		SpoVR family protein	-3,75	2,8	270,2	136,5	2,1	1,9	2,0
YPK_2179	prfA	peptide chain release factor 1	-3,75	164,0	2,3	83,1	1,5	1,4	1,4
YPK_2196		hypothetical protein	-3,76	165,4	2,3	83,8	1,5	1,3	1,4
YPK_1974		aldehyde dehydrogenase	-3,76	2,7	272,8	137,8	2,1	1,9	2,0
YPK_0152	glpD	glycerol-3-phosphate dehydrogenase	-3,77	235,1	3,2	119,1	2,1	1,9	2,0
YPK_2528		imidazole glycerol-phosphatedehydratase/histidinol	-3,77	166,3	2,2	84,3	1,5	1,3	1,4
YPK_1899		hypothetical protein	-3,78	1,9	194,8	98,4	1,5	1,3	1,4
YPK_3403		carbohydrate kinase FGGY	-3,79	2,7	277,2	139,9	2,1	1,9	2,0
YPK_4167		hypothetical protein	-3,79	168,6	2,2	85,4	1,5	1,3	1,4
YPK_4067		ABC transporter-like protein	-3,79	238,8	3,1	121,0	2,1	1,9	2,0
YPK_3501		guanosine 5'-monophosphate oxidoreductase	-3,80	170,1	2,2	86,1	1,5	1,3	1,4
YPK_2542		transport system permease	-3,81	170,6	2,2	86,4	1,5	1,3	1,4
YPK_2674	ansB	L-asparaginase II	-3,81	171,0	398,6	284,8	1,5	237,9	119,7
YPK_1572		NADH dehydrogenase subunit N	-3,82	243,2	3,1	123,1	2,0	1,8	1,9
YPK_3938		delta-aminolevulinic acid dehydratase	-3,83	1,8	202,2	102,0	1,4	1,3	1,4
YPK_4137	gpsA	NAD(P)H-dependent glycerol-3-phosphatede	-3,84	1,8	202,8	102,3	1,4	1,3	1,4
YPK_2092	ansA	asparaginase	-3,84	1,8	203,4	102,6	1,4	1,3	1,4
YPK_2943		UDP-galactose-4-epimerase	-3,84	174,5	2,1	88,3	1,4	1,3	1,4
YPK_4114	glpX	fructose 1,6-bisphosphatase II	-3,85	175,6	2,1	88,8	1,4	1,3	1,3

YPK_4122	anion transporter	-3,85	2,6	290,5	146,5	2,0	1,8	1,9
YPK_2891	helicase domain-containing protein	-3,86	1,8	205,8	103,8	1,4	1,3	1,3
YPK_4089	argC N-acetyl-gamma-glutamyl-phosphate reducta	-3,86	176,6	2,1	89,4	1,4	1,3	1,3
YPK_0964	periplasmic binding protein/LacI transcriptionalregul	-3,86	1,8	206,4	104,1	1,4	1,3	1,3
YPK_1985	fumC fumarate hydratase	-3,88	2,5	296,7	149,6	2,0	1,8	1,9
YPK_1116	nrdF ribonucleotide-diphosphate reductase subuni	-3,90	1,8	212,7	107,2	1,4	1,2	1,3
YPK_1592	O-succinylbenzoate synthase	-3,90	1,8	212,7	107,2	1,4	1,2	1,3
YPK_2152	methyltransferase	-3,90	182,5	2,0	92,3	1,4	1,2	1,3
YPK_3193	hemH ferrochelatase	-3,92	184,2	2,0	93,1	1,4	1,2	1,3
YPK_3552	type VI secretion protein	-3,93	262,4	2,8	132,6	1,9	1,7	1,8
YPK_0496	treR trehalose repressor	-3,93	185,9	2,0	94,0	1,3	1,2	1,3
YPK_0315	fmt methionyl-tRNA formyltransferase	-3,94	187,1	2,0	94,6	1,3	1,2	1,3
YPK_1179	ATP-dependent RNA helicase SrmB	-3,96	2,4	312,7	157,5	1,9	1,7	1,8
YPK_2865	glycine betaine ABC transportersubstrate-binding pı	-3,96	190,1	2,0	96,0	1,3	1,2	1,2
YPK_3626	deoA thymidine phosphorylase	-3,96	268,9	2,8	135,9	1,9	1,7	1,8
YPK_1611	monosaccharide-transporting ATPase	-3,99	193,2	225,1	209,1	150,0	1,2	75,6
YPK_0725	putative transcriptional regulator CadC	-3,99	1,7	225,1	113,4	1,3	1,2	1,2
YPK_3797	putative GTPase HflX	-4,00	276,4	483,1	379,7	322,0	1,6	161,8
YPK_2639	PqiA family integral membrane protein	-4,00	276,4	2,7	139,6	1,8	1,6	1,7
YPK_3262	secD preprotein translocase subunit SecD	-4,01	98,2	457,8	278,0	76,3	2,3	39,3
YPK_0376	malG maltose transporter permease	-4,03	1,6	231,8	116,7	1,3	1,1	1,2
YPK_2436	copper resistance D domain-containing protein	-4,04	200,3	1,9	101,1	1,2	1,1	1,2
YPK_4186	rbn ribonuclease BN	-4,04	200,3	1,9	101,1	1,2	1,1	1,2
YPK_3402	putative L-xylulose 5-phosphate 3-epimerase	-4,04	1,6	234,1	117,9	1,2	1,1	1,2
YPK_1445	hypothetical protein	-4,05	2,3	332,0	167,1	1,7	1,6	1,7
YPK_3290	frsA fermentation/respiration switch protein	-4,05	285,0	2,6	143,8	1,7	1,6	1,7
YPK_3625	phosphopentomutase	-4,07	2,2	338,5	170,4	1,7	1,5	1,6
YPK_1727	NmrA family protein	-4,08	206,5	1,8	104,2	1,2	1,1	1,1
YPK_3523	murE UDP-N-acetylmuramoylalanyl-D-glutamate--	-4,09	2,7	418,3	210,5	2,1	1,9	2,0
YPK_3495	regulatory protein AmpE	-4,09	1,5	241,4	121,5	1,2	1,1	1,1
YPK_2488	hypothetical protein	-4,09	207,9	1,8	104,9	1,2	1,1	1,1
YPK_2080	purU formyltetrahydrofolate deformylase	-4,10	208,7	1,8	105,2	1,2	1,1	1,1
YPK_3891	periplasmic binding protein	-4,11	210,9	1,8	106,3	1,2	1,1	1,1
YPK_1968	class I and II aminotransferase	-4,13	301,5	2,5	152,0	1,7	1,5	1,6
YPK_3583	carbamoyl phosphate synthase small subunit	-4,13	2,1	352,2	177,2	1,6	1,5	1,6
YPK_2902	ABC-2 type transporter	-4,13	302,3	2,5	152,4	1,6	1,5	1,6
YPK_3923	actP acetate permease	-4,14	215,2	501,4	358,3	167,1	2,1	84,6
YPK_4189	glnA glutamine synthetase	-4,16	378,7	3,0	190,8	2,0	1,8	1,9
YPK_3900	type III secretion protein SpaR/YscT/HrcT	-4,19	222,7	1,7	112,2	1,1	1,0	1,1
YPK_0113	transcriptional regulator CdaR	-4,19	2,0	367,1	184,5	1,6	1,4	1,5
pYV0065	yopN, lcrE, membrane-bound Yop targetingprotein	-4,20	8641,1	6790,1	7715,6	312,2	559,0	435,6
YPK_1078	UDP-N-acetylglucosamine acyltransferase	-4,20	1,4	261,4	131,4	1,1	1,0	1,1
YPK_2235	hypothetical protein	-4,20	1,4	261,4	131,4	1,1	1,0	1,1
YPK_3974	extracellular ligand-binding receptor	-4,21	318,4	2,3	160,4	1,6	1,4	1,5
YPK_1526	AraC family transcriptional regulator	-4,21	225,2	1,7	113,4	1,1	1,0	1,0
YPK_0513	hypothetical protein	-4,21	1,4	263,4	132,4	1,1	1,0	1,0
YPK_2548	ferric hydroximate transport ferric ironreductase	-4,22	226,9	1,6	114,3	1,1	1,0	1,0
YPK_3344	pssA phosphatidylserine synthase	-4,22	2,4	458,7	230,6	1,9	1,7	1,8
YPK_2787	binding-protein-dependent transport system innerm	-4,23	322,7	2,3	162,5	1,5	1,4	1,5
YPK_3307	glutamine amidotransferase	-4,23	228,6	1,6	115,1	1,1	1,0	1,0
YPK_2010	rnfD electron transport complex protein RnfD	-4,23	324,4	2,3	163,4	1,5	1,4	1,5
YPK_2750	transport system permease	-4,25	328,0	2,3	165,1	1,5	1,4	1,4
YPK_3006	PhoH family protein	-4,26	330,7	2,3	166,5	1,5	1,3	1,4
YPK_0351	hemE uroporphyrinogen decarboxylase	-4,27	332,6	2,2	167,4	1,5	1,3	1,4
YPK_2168	ABC transporter-like protein	-4,27	332,6	2,2	167,4	1,5	1,3	1,4
YPK_1357	hypothetical protein	-4,27	333,5	2,2	167,9	1,5	1,3	1,4
YPK_3180	glycosyl transferase family protein	-4,27	1,4	274,8	138,1	1,1	0,9	1,0
YPK_3687	binding-protein-dependent transport system innerm	-4,29	1,9	394,1	198,0	1,5	1,3	1,4
YPK_1533	hypothetical protein	-4,30	239,6	1,6	120,6	1,0	0,9	1,0

YPK_3636	rsmC 16S ribosomal RNA m2G1207 methyltransfe	-4,30	340,1	2,2	171,2	1,5	1,3	1,4
YPK_0824	hypothetical protein	-4,30	1,3	280,3	140,8	1,0	0,9	1,0
YPK_0231	tryptophanyl-tRNA synthetase	-4,31	341,1	2,2	171,6	1,5	1,3	1,4
YPK_3352	outer membrane protein assembly complex subunit	-4,31	241,6	1,5	121,6	1,0	0,9	1,0
YPK_4080	glutaredoxin family protein	-4,31	241,6	1,5	121,6	1,0	0,9	1,0
YPK_0672	tonB-system energizer ExbB	-4,31	1,9	398,6	200,2	1,5	1,3	1,4
YPK_0326	murB UDP-N-acetylenolpyruvoylglucosamine redu	-4,31	342,1	2,2	172,1	1,5	1,3	1,4
YPK_2656	condesin subunit E	-4,31	1,3	282,6	142,0	1,0	0,9	1,0
YPK_0535	mtgA monofunctional biosynthetic peptidoglycantra	-4,32	1,3	283,8	142,5	1,0	0,9	1,0
YPK_1068	pyrH uridylate kinase	-4,32	243,5	1,5	122,5	1,0	0,9	1,0
YPK_2788	binding-protein-dependent transport system innerm	-4,32	345,0	2,2	173,6	1,4	1,3	1,4
YPK_2377	D-cysteine desulfhydrase	-4,34	348,1	2,1	175,1	1,4	1,3	1,4
YPK_4235	transcriptional regulator PhoU	-4,34	246,6	1,5	124,0	1,0	0,9	1,0
YPK_2078	response regulator of RpoS	-4,34	1,8	406,7	204,3	1,4	1,3	1,4
YPK_1614	LacI family transcriptional regulator	-4,36	353,2	2,1	177,7	1,4	1,3	1,3
YPK_2066	oligopeptide/dipeptide ABC transporter ATPase	-4,36	354,3	206,4	280,3	1,4	123,2	62,3
YPK_0568	von Willebrand factor type A	-4,37	251,8	1,5	126,6	1,0	0,9	0,9
YPK_3995	lysophospholipase L2	-4,37	1,8	415,2	208,5	1,4	1,3	1,3
YPK_0431	carbohydrate ABC transporter periplasmic-bindingp	-4,38	358,5	2,1	180,3	1,4	1,2	1,3
YPK_1134	ureE urease accessory protein UreE	-4,38	253,9	1,5	127,7	1,0	0,9	0,9
YPK_2782	cobalamin synthesis protein P47K	-4,39	360,7	2,1	181,4	1,4	1,2	1,3
YPK_3351	rluD 23S rRNA pseudouridine synthase D	-4,40	362,9	2,1	182,5	1,4	1,2	1,3
YPK_3158	putative ABC transporter ATP-binding proteinYbbA	-4,40	1,2	299,6	150,4	1,0	0,9	0,9
YPK_1984	DNA replication terminus site-binding protein	-4,40	1,8	424,1	212,9	1,4	1,2	1,3
YPK_3417	DeoR family transcriptional regulator	-4,40	1,8	425,4	213,6	1,4	1,2	1,3
YPK_4129	putative transposase YhgA family protein	-4,42	1,7	430,6	216,2	1,3	1,2	1,3
YPK_1940	hypothetical protein	-4,43	371,9	2,0	187,0	1,3	1,2	1,3
YPK_2259	LysR family transcriptional regulator	-4,44	1,7	437,4	219,5	1,3	1,2	1,3
YPK_0083	regulatory protein UhpC	-4,45	2,4	622,5	312,5	1,9	1,7	1,8
YPK_4179	tRNA guanosine-2'-O-methyltransferase	-4,46	267,5	1,4	134,5	0,9	0,8	0,9
YPK_1167	allophanate hydrolase subunit 1	-4,46	268,7	1,4	135,1	0,9	0,8	0,9
YPK_0923	prfB peptide chain release factor 2	-4,46	380,2	2,0	191,1	1,3	1,2	1,2
YPK_0355	hypothetical protein	-4,47	270,0	1,4	135,7	0,9	0,8	0,9
YPK_4184	thioesterase domain-containing protein	-4,48	383,8	1,9	192,9	1,3	1,2	1,2
YPK_2069	oppB oligopeptide transporter permease	-4,48	1,7	448,7	225,2	1,3	1,2	1,2
YPK_2093	nicotinamidase/pyrazinamidase	-4,48	272,4	1,4	136,9	0,9	0,8	0,9
YPK_3737	rrmJ 23S rRNA methyltransferase J	-4,52	1,1	326,3	163,7	0,9	0,8	0,8
YPK_2536	UTP-glucose-1-phosphate uridylyltransferase	-4,52	396,6	1,9	199,2	1,3	1,1	1,2
YPK_2858	hypothetical protein	-4,53	1,6	463,6	232,6	1,3	1,1	1,2
YPK_4034	rho transcription termination factor Rho	-4,53	564,6	2,6	283,6	1,8	1,6	1,7
YPK_0035	superoxide dismutase	-4,54	565,5	329,4	447,4	439,2	0,8	220,0
YPK_0931	putative bacteriophage protein	-4,54	1,1	329,4	165,3	0,9	0,8	0,8
YPK_2970	sdhA succinate dehydrogenase flavoprotein subur	-4,55	201,7	470,1	335,9	2,5	70,2	36,3
YPK_3555	hypothetical protein	-4,55	285,4	1,3	143,4	0,9	0,8	0,8
YPK_1366	hypothetical protein	-4,56	1,6	474,7	238,1	1,2	1,1	1,2
YPK_3161	thioredoxin domain-containing protein	-4,56	1,6	474,7	238,1	1,2	1,1	1,2
pYV0074	yscU, type III secretion protein	-4,57	1,9	582,8	292,4	1,5	1,3	1,4
YPK_2292	transposase mutator type	-4,58	1,6	479,6	240,6	1,2	1,1	1,1
YPK_3392	DMSO reductase anchor subunit DmsC	-4,58	1,6	479,6	240,6	1,2	1,1	1,1
pYV0037		-4,58	1,1	339,0	170,1	0,9	0,8	0,8
YPK_2036	putative periplasmic protease	-4,59	1,9	592,8	297,3	1,5	1,3	1,4
YPK_1390	NUDIX hydrolase	-4,60	295,3	1,3	148,3	0,8	0,8	0,8
YPK_0661	NAD(P)H dehydrogenase (quinone)	-4,63	1,1	351,0	176,0	0,8	0,7	0,8
YPK_1695	shikimate 5-dehydrogenase	-4,65	432,5	1,7	217,1	1,2	1,0	1,1
YPK_3196	recR recombination protein RecR	-4,65	305,9	1,2	153,6	0,8	0,7	0,8
pYV0076	lcrF, virF, thermoregulatory protein	-4,65	434,0	1,7	217,9	1,1	1,0	1,1
YPK_2163	hypothetical protein	-4,68	312,3	1,2	156,7	0,8	0,7	0,8
YPK_1275	DNA-binding transcriptional regulator IscR	-4,69	1,0	367,7	184,4	0,8	0,7	0,7
YPK_3366	cytochrome c assembly protein	-4,70	447,0	1,7	224,3	1,1	1,0	1,1

YPK_0991	PTS sorbose-specific transporter subunit IIC	-4,70	448,7	1,7	225,2	1,1	1,0	1,1
YPK_3406	methionine aminopeptidase	-4,71	450,4	1,7	226,0	1,1	1,0	1,0
YPK_4150	glycosyl transferase family protein	-4,71	1,4	526,7	264,1	1,1	1,0	1,0
YPK_0489	hypothetical protein	-4,72	320,7	1,2	160,9	0,8	0,7	0,7
YPK_1349	N-acetyltransferase GCN5	-4,73	322,4	1,2	161,8	0,8	0,7	0,7
YPK_2055	transporter	-4,73	459,0	1,6	230,3	1,1	1,0	1,0
YPK_2037	short chain dehydrogenase	-4,75	464,4	1,6	233,0	1,1	1,0	1,0
YPK_2293	hypothetical protein	-4,76	329,8	384,3	357,0	128,1	1,4	64,7
YPK_0557	hypothetical protein	-4,76	1,0	386,1	193,5	0,8	0,7	0,7
YPK_3753	hypothetical protein	-4,81	1,6	688,5	345,1	1,3	1,1	1,2
YPK_2847	3-demethylubiquinone-9 3-methyltransferase	-4,81	1,3	565,2	283,3	1,0	0,9	1,0
YPK_4121	hypothetical protein	-4,84	1,3	576,9	289,1	1,0	0,9	1,0
YPK_2245	redoxin domain-containing protein	-4,86	352,9	1,1	177,0	0,7	0,6	0,7
YPK_2793	16S rRNA pseudouridylate synthase A	-4,86	1,3	581,8	291,5	1,0	0,9	0,9
YPK_3725	lipoprotein NlpI	-4,91	1001,4	933,4	967,4	1,2	835,7	418,5
YPK_0177	HAD family hydrolase	-4,91	518,8	1,4	260,1	1,0	0,9	0,9
YPK_1189	rnc ribonuclease III	-4,91	518,8	1,4	260,1	1,0	0,9	0,9
YPK_1511	phosphohistidine phosphatase	-4,92	368,1	1,0	184,5	0,7	0,6	0,6
YPK_0147	glycogen branching protein	-4,93	81,7	95,2	88,4	3,0	2,7	2,9
YPK_3020	rRNA large subunit methyltransferase	-4,93	0,9	434,2	217,5	0,7	0,6	0,6
YPK_1405	hypothetical protein	-4,93	372,7	1,0	186,8	0,7	0,6	0,6
YPK_0459	acetyl-CoA carboxylase biotin carboxyl carrierprotei	-4,95	377,4	1,0	189,2	0,7	0,6	0,6
YPK_3223	AsnC family transcriptional regulator	-4,96	379,8	1,0	190,4	0,7	0,6	0,6
YPK_1685	malonyl CoA-acyl carrier protein transacylase	-4,97	381,4	444,4	412,9	1,3	132,6	67,0
YPK_3890	hemin-degrading family protein	-4,97	855,2	2,2	428,7	1,5	1,3	1,4
YPK_0856	hypothetical protein	-4,97	663,1	1,7	332,4	1,1	1,0	1,1
YPK_4159	pyrE orotate phosphoribosyltransferase	-4,98	1,2	634,8	318,0	0,9	0,8	0,9
YPK_2739	serine transporter	-4,99	273,3	636,7	455,0	106,1	1,6	53,9
YPK_1694	PTS system glucose-specific transporter subunitIIB	-5,01	993,1	723,2	858,1	2,0	345,3	173,7
YPK_4076	DNA-binding transcriptional repressor FabR	-5,01	1,2	646,6	323,9	0,9	0,8	0,8
YPK_0602	YheO domain-containing protein	-5,02	1,1	649,6	325,4	0,9	0,8	0,8
YPK_1384	two component LuxR family transcriptionalregulator	-5,02	560,2	1,3	280,7	0,9	0,8	0,8
YPK_1313		-5,05	402,8	0,9	201,9	0,6	0,6	0,6
YPK_4140	rhodanese domain-containing protein	-5,05	402,8	0,9	201,9	0,6	0,6	0,6
YPK_0730	MotA/TolQ/ExbB proton channel	-5,08	582,0	1,3	291,6	0,9	0,8	0,8
YPK_3877	stress protein	-5,11	593,6	1,3	297,4	0,8	0,8	0,8
pYV0025	outer membrane virulence protein	-5,12	63407,2	60162,3	61784,7	415,6	7628,8	4022,2
YPK_0658	esterase YqiA	-5,13	605,6	1,2	303,4	0,8	0,7	0,8
YPK_0242	peptidyl-prolyl cis-trans isomerase A	-5,16	618,1	1,2	309,7	0,8	0,7	0,8
YPK_2121	minC septum formation inhibitor	-5,19	1,2	898,9	450,1	1,0	0,9	0,9
pYV0073	yscT, type III secretion protein	-5,21	900,8	1,7	451,2	1,1	1,0	1,0
YPK_2643	hypothetical protein	-5,21	1,0	743,3	372,1	0,8	0,7	0,7
YPK_4220	F0F1 ATP synthase subunit A	-5,22	644,0	750,4	697,2	333,5	1,0	167,3
YPK_0507	lipopolysaccharide transport periplasmic proteinLpt/	-5,23	644,8	1,2	323,0	0,8	0,7	0,7
YPK_3319	acireductone dioxygenase ARD	-5,23	1,0	755,3	378,2	0,8	0,7	0,7
YPK_2094	hypothetical protein	-5,26	465,6	0,8	233,2	0,5	0,5	0,5
YPK_1921	hypothetical protein	-5,27	0,7	546,7	273,7	0,5	0,5	0,5
YPK_2251	hypothetical protein	-5,27	1,0	776,4	388,7	0,7	0,7	0,7
YPK_1976	3-hydroxy acid dehydrogenase	-5,27	943,4	1,6	472,5	1,1	0,9	1,0
pYV0062	yscX, type III secretion protein	-5,28	473,0	0,8	236,9	0,5	0,5	0,5
YPK_1945	methylated-DNA--protein-cysteinemethyltransferase	-5,30	677,7	1,1	339,4	0,7	0,7	0,7
YPK_0771	hypothetical protein	-5,30	0,7	559,9	280,3	0,5	0,5	0,5
YPK_2198	hypothetical protein	-5,37	1,7	1550,3	776,0	1,3	1,2	1,2
YPK_3247	putative nucleotide-binding protein	-5,37	714,2	1,0	357,6	0,7	0,6	0,7
YPK_2291	transposase	-5,40	0,6	598,4	299,5	0,5	0,4	0,5
YPK_0031	hypothetical protein	-5,42	0,9	857,7	429,3	0,7	0,6	0,6
YPK_2759	pH 6 antigen	-5,42	0,9	857,7	429,3	0,7	0,6	0,6
YPK_4138	preprotein translocase subunit SecB	-5,42	0,9	857,7	429,3	0,7	0,6	0,6
YPK_2023	translation initiation factor Sui1	-5,45	531,9	0,7	266,3	0,5	0,4	0,4

YPK_4215	DNA-binding transcriptional regulator AsnC	-5,46	0,8	885,0	442,9	0,7	0,6	0,6
YPK_3209	transposase IS200-family protein	-5,47	764,4	1,0	382,7	0,7	0,6	0,6
YPK_4194	engB ribosome biogenesis GTP-binding protein Ys	-5,48	1,2	1263,9	632,5	0,9	0,8	0,9
YPK_2164	cytoplasmic chaperone TorD family protein	-5,48	1,0	1097,3	549,2	0,8	0,7	0,8
YPK_2027	hypothetical protein	-5,51	0,6	648,6	324,6	0,4	0,4	0,4
YPK_3390	cytochrome b561	-5,56	994,0	1,1	497,6	0,8	0,7	0,7
YPK_3463	mrcB penicillin-binding protein 1b	-5,57	143,9	167,7	155,8	3,5	3,1	3,3
YPK_3120	putative bacteriophage protein	-5,57	578,2	0,6	289,4	0,4	0,4	0,4
pYV0094	yopH, protein-tyrosine phosphatase Yop effector	-5,57	57937,6	57482,6	57710,1	982,6	1495,6	1239,1
pYV0091	transposase	-5,59	0,8	964,7	482,7	0,6	0,5	0,6
YPK_0274	sulfur transfer complex subunit TusB	-5,63	601,5	0,6	301,1	0,4	0,4	0,4
YPK_0647	hypothetical protein	-5,66	0,5	715,3	357,9	0,4	0,4	0,4
YPK_4177	rpoZ DNA-directed RNA polymerase subunit omeg	-5,69	0,5	730,3	365,4	0,4	0,4	0,4
YPK_0525	rpsI 30S ribosomal protein S9	-5,69	889,6	518,2	703,9	345,5	0,5	173,0
YPK_0501	phosphohistidinoprotein-hexosephosphotransferase	-5,70	0,5	738,0	369,3	0,4	0,4	0,4
pYV0090	transposase	-5,73	0,5	754,0	377,2	0,4	0,3	0,4
YPK_0909	hypothetical protein	-5,73	647,1	0,6	323,8	0,4	0,3	0,4
YPK_3929	hemG protoporphyrinogen oxidase	-5,76	1318,0	1,1	659,6	0,8	0,7	0,7
YPK_1847	sufA iron-sulfur cluster assembly scaffold protein	-5,77	938,5	0,8	469,6	0,5	0,5	0,5
YPK_2295	hypothetical protein	-5,81	684,2	0,5	342,4	0,4	0,3	0,3
YPK_3486	hypothetical protein	-5,81	968,9	0,8	484,9	0,5	0,5	0,5
YPK_2879	hypothetical protein	-5,83	0,5	806,4	403,4	0,4	0,3	0,3
YPK_2971	sdhD succinate dehydrogenase cytochrome b556	-5,86	1001,4	0,7	501,1	0,5	0,4	0,5
YPK_3108	DinI family protein	-5,86	708,5	0,5	354,5	0,4	0,3	0,3
pYV0079	yscC, type III secretion protein	-5,86	1075,1	2733,1	1904,1	2,5	339,9	171,2
YPK_3153	peptidyl-prolyl cis-trans isomerase B	-5,86	709,9	1654,3	1182,1	0,7	493,7	247,2
YPK_3457	iron-sulfur cluster insertion protein ErpA	-5,87	1009,9	0,7	505,3	0,5	0,4	0,5
pYV0082	yscF, type III secretion protein	-5,90	36635,5	44972,1	40803,8	508,1	910,0	709,0
YPK_3722	XRE family transcriptional regulator	-6,01	0,6	1297,2	648,9	0,4	0,4	0,4
pYV0049	hypothetical protein	-6,03	793,2	0,5	396,8	0,3	0,3	0,3
pYV0069	yscP, type III secretion protein	-6,06	2341,5	1970,3	2155,9	1,9	542,8	272,4
pYV0017	resolvase	-6,07	956,9	4087,9	2522,4	1238,7	0,7	619,7
YPK_3655	autoinducer-2 (AI-2) modifying protein LsrG	-6,11	1191,0	0,6	595,8	0,4	0,4	0,4
YPK_0284	rplD 50S ribosomal protein L4	-6,16	2618,9	1017,1	1818,0	0,9	607,1	304,0
YPK_1765	hypothetical protein	-6,17	0,4	1018,8	509,6	0,3	0,3	0,3
YPK_0280	bacterioferritin-associated ferredoxin	-6,17	874,4	0,4	437,4	0,3	0,3	0,3
YPK_1807	transposase	-6,19	0,4	1033,9	517,1	0,3	0,3	0,3
YPK_0494	trehalose-6-phosphate hydrolase	-6,22	107,2	249,8	178,5	2,3	2,1	2,2
YPK_3236	hypothetical protein	-6,23	914,6	0,4	457,5	0,3	0,2	0,3
pYV0039	transposase	-6,25	928,8	0,4	464,6	0,3	0,2	0,3
YPK_0301	rpmD 50S ribosomal protein L30	-6,28	943,4	0,4	471,9	0,3	0,2	0,2
YPK_2088	DNA topoisomerase III	-6,29	184,9	215,4	200,2	2,7	2,4	2,6
YPK_3757	rplU 50S ribosomal protein L21	-6,31	0,6	1945,8	973,2	0,4	0,4	0,4
YPK_0299	rplR 50S ribosomal protein L18	-6,34	1969,8	2295,1	2132,5	0,5	1370,0	685,2
pYV0060	lcrD, yscV, membrane-bound Yop protein	-6,37	1265,3	1867,4	1566,4	131,0	2,6	66,8
YPK_3885	coproporphyrinogen III oxidase	-6,39	135,4	157,7	146,6	1,8	1,6	1,7
YPK_4154	rpmB 50S ribosomal protein L28	-6,40	1451,4	845,6	1148,5	0,3	504,7	252,5
YPK_2651	hypothetical protein	-6,58	0,3	1356,2	678,2	0,2	0,2	0,2
pYV0007	replication protein	-6,59	0,5	2364,4	1182,4	0,4	0,3	0,3
pYV0054	yopD, Yop negative regulation/targetingcomponent	-6,61	95113,6	80759,6	87936,6	747,7	1071,2	909,5
YPK_0554	glucuronate isomerase	-6,69	252,5	147,1	199,8	2,0	1,8	1,9
YPK_4078	soluble pyridine nucleotide transhydrogenase	-6,71	127,0	296,0	211,5	2,0	1,8	1,9
YPK_1810	hypothetical protein	-6,71	0,4	2099,0	1049,7	0,3	0,2	0,3
YPK_1525	hypothetical protein	-6,73	1289,6	0,3	644,9	0,2	0,2	0,2
YPK_1194	iron-sulfur cluster-binding protein	-6,73	0,3	2131,2	1065,8	0,3	0,2	0,3
YPK_3445	superoxide dismutase	-6,77	1746,0	2373,3	2059,7	0,9	404,8	202,8
YPK_3889	TonB-dependent heme/hemoglobin receptor familyp	-6,80	175,7	511,6	343,6	2,8	2,5	2,7
YPK_2252	iron permease FTR1	-6,80	185,8	432,9	309,3	2,7	2,4	2,5
YPK_2789	ABC transporter-like protein	-6,84	223,6	260,6	242,1	2,2	2,0	2,1

pYV0001	ypkA, targeted effector protein kinase	-6,86	2921,3	3214,6	3067,9	3,1	225,7	114,4
YPK_0269	hypothetical protein	-7,01	3131,0	0,5	1565,8	0,3	0,3	0,3
YPK_0520	protease Do	-7,06	129,5	452,7	291,1	1,9	1,7	1,8
pYV0002	hypothetical protein	-7,08	4676,2	1362,1	3019,1	0,6	542,0	271,3
YPK_1858	cyclopropane-fatty-acyl-phospholipid synthase	-7,27	154,3	359,5	256,9	1,6	1,4	1,5
YPK_2831	outer membrane protease	-7,36	189,5	220,8	205,1	1,3	1,2	1,2
YPK_1688	3-oxoacyl-(acyl carrier protein) synthase II	-7,55	286,3	333,6	310,0	1,7	1,6	1,6
YPK_1031	tas putative aldo-keto reductase	-7,56	170,6	397,4	284,0	1,5	1,3	1,4
YPK_3795	FtsH protease regulator HflC	-7,66	176,6	411,5	294,1	1,4	1,3	1,3
pYV0080	yscD, type III secretion protein	-7,67	3105,2	2302,3	2703,7	1,8	98,2	50,0
YPK_3338	efflux pump membrane protein	-7,72	303,0	353,1	328,1	1,6	1,5	1,6
YPK_2565	galactose/methyl galaxtoside transporterATP-bindin	-7,76	234,2	818,5	526,3	2,1	1,9	2,0
YPK_3388	catalase/peroxidase HPI	-7,90	4594,2	4319,8	4457,0	3,1	112,1	57,6
YPK_3949	protein tyrosine/serine phosphatase	-8,15	209,4	488,0	348,7	1,2	1,1	1,1
YPK_1177	ankyrin	-8,37	268,7	313,1	290,9	0,9	0,8	0,9
YPK_3452	serine endoprotease	-8,41	492,5	860,7	676,6	2,0	1,8	1,9
YPK_1375	extracellular solute-binding protein	-8,43	326,2	760,2	543,2	1,5	1,4	1,4
YPK_1364	phosphoribosylaminoimidazole-succinocarboxamid	-8,63	247,6	576,9	412,3	1,0	0,9	1,0
YPK_0320	putative ribosome maturation factor	-8,76	307,5	358,2	332,9	0,8	0,7	0,8
pYV0083	yscG, type III secretion protein	-8,78	6008,5	11084,4	8546,4	0,5	696,5	348,5
pYV0087	yscK, type III secretion protein	-8,90	542,4	316,0	429,2	0,9	0,8	0,9
YPK_3194	adk adenylate kinase	-8,92	547,3	318,9	433,1	0,9	0,8	0,9
YPK_2634	3-hydroxydecanoyl-ACP dehydratase	-9,00	281,4	655,7	468,6	0,9	0,8	0,8
pYV0057	lcrV, V antigen, antihost protein/regulator	-9,10	10491,1	9905,3	10198,2	1,4	251,6	126,5
pYV0070	type III secretion system protein	-9,56	959,6	894,4	927,0	1,3	1,2	1,2
YPK_2197	hypothetical protein	-9,62	782,8	1140,0	961,4	1,3	1,1	1,2
YPK_3713	N-acetyltransferase GCN5	-9,62	348,7	812,7	580,7	0,7	0,6	0,7
YPK_2199	hypothetical protein	-9,72	1305,7	869,3	1087,5	1,3	1,2	1,3
pYV0055	yopB, Yop targeting protein	-9,97	34052,1	30744,0	32398,1	1,7	615,1	308,4
YPK_2974	heat shock protein GrpE	-10,23	608,7	1418,4	1013,5	0,8	0,7	0,8
pYV0092	transposase	-10,26	870,2	506,9	688,5	0,6	0,5	0,5
YPK_0272	sulfur transfer complex subunit TusD	-10,30	883,0	514,4	698,7	0,6	0,5	0,5
pYV0047	yopM, targeted effector protein	-10,51	3233,7	3139,7	3186,7	2,3	2,1	2,2
pYV0098	yopP, yopJ, targeted effector protein	-10,53	2043,9	1428,9	1736,4	1,2	1,1	1,2
pYV0071	type III secretion system protein	-10,58	809,9	2201,8	1505,8	0,9	0,8	0,9
YPK_2795	50S ribosomal protein L25	-10,73	607,6	708,0	657,8	0,4	0,4	0,4
pYV0020	sycH, yopH targeting protein	-10,89	1233,4	958,0	1095,7	0,6	0,5	0,6
YPK_0521	cytochrome d ubiquinol oxidase subunit III	-11,03	1295,8	1006,5	1151,2	0,6	0,5	0,5
YPK_1569	NADH dehydrogenase subunit K	-11,06	572,7	1334,5	953,6	0,4	0,4	0,4
YPK_4139	glutaredoxin 3	-11,10	692,1	806,4	749,2	0,4	0,3	0,3
YPK_2200	hypothetical protein	-11,16	36083,3	50179,1	43131,2	0,2	1619,0	809,6
pYV0088	type III secretion system protein	-11,49	3624,1	1624,1	2624,1	0,9	0,8	0,8
YPK_0636	rpsU 30S ribosomal protein S21	-11,50	793,2	924,1	858,6	0,3	0,3	0,3
pYV0040	yop targeting protein yopK, yopQ	-11,58	1924,0	2615,3	2269,7	0,8	0,7	0,7
pYV0086	yscJ, ylpB, type III secretion lipoprotein	-11,89	4090,3	3364,1	3727,2	1,0	0,9	1,0
pYV0063	sycN, type III secretion protein	-12,07	1407,7	3280,4	2344,1	0,5	0,5	0,5
pYV0015		-12,14	990,3	1153,9	1072,1	0,3	0,2	0,2
pYV0068	yscO, type III secretion protein	-12,25	2641,6	3517,5	3079,6	0,7	0,6	0,6
pYV0078	hypothetical protein	-12,26	2536,6	2955,5	2746,0	0,6	0,5	0,6
pYV0089	yscM, lcrQ, type III secretion regulatory	-12,36	3504,9	1750,2	2627,6	0,5	0,4	0,5
YPK_3389	cytochrome b562	-12,64	2274,8	4770,8	3522,8	0,5	0,5	0,5
pYV0084	yscH, yopR, lcrP, type III secretion protein	-12,72	4940,0	4111,3	4525,7	0,7	0,6	0,7
pYV0085	yscI, lcrO, type III secretion protein	-12,83	2503,5	4667,1	3585,3	0,5	0,4	0,5
pYV0058	lcrG, Yop regulator	-13,10	2406,1	4906,0	3656,0	0,4	0,4	0,4
pYV0053	hypothetical protein	-13,19	1007,0	4693,3	2850,2	0,2	0,2	0,2
pYV0064	tyeA, Yop secretion and targeting protein	-13,58	4962,0	4336,1	4649,0	0,4	0,4	0,4
pYV0081	yscE, type III secretion protein	-13,65	2548,6	4949,1	3748,9	0,3	0,3	0,3
pYV0056	lcrH, sycD, low calcium response protein H	-14,94	22189,9	20602,6	21396,2	0,7	0,6	0,7
