# Supplementary material for: Reprogramming of Yersinia from Virulent to Persistent Mode Revealed by Complex In Vivo RNA-seq Analysis
Source: PLoS Pathog. 2015 Jan 15;11(1):e1004600. doi: 10.1371/journal.ppat.1004600 (PMC4295882; doi:10.1371/journal.ppat.1004600)
Supplement: S4 Table — Up- and downregulated genes in wt Y. pseudotuberculosis YPIII during persistent infection in mice, at 26°C in vitro, under anaerobic condition, and in ΔrovA, ΔcsrA, and Δcrp mutant strains. The list is sorted from highest to lowest value of upregulation during persistent infection. *Indicates data obtained from Bucker et al., 2014. (PDF) [file ppat.1004600.s011.pdf]

Table S4. Comparison of *Y. pseudotuberculosis* YPIII gene expression at different conditions and in *rovA, csrA,* and *crp* mutants. Up- and downregulation of the genes in wt *Y. pseudotuberculosis* YPIII during persistent infection in mice, at 26°C *in vitro*, under anaerobic condition, in *ΔrovA*, *ΔcsrA*, and *Δcrp* strains. The list is sorted with the value of upregulation during persistent infection.
*Indicates data obtained from Bucker et al., 2014.

Locus Tag	Definition	Persistent infection	26°C	Anaerobic condition	*ΔrovA**	*ΔcsrA**	*Δcrp**
YPK_0304	rpmJ 50S ribosomal protein L36	UP	UP	UP	-	-	-
YPK_0120	UspA domain-containing protein	UP	UP	DOWN	DOWN	-	DOWN
YPK_0631	hypothetical protein	UP	UP	DOWN	-	DOWN	DOWN
YPK_2475	hypothetical protein	UP	-	UP	DOWN	DOWN	DOWN
YPK_0504	putative sigma(54) modulation protein	UP	UP	DOWN	-	-	UP
YPK_3799	hfq RNA-binding protein Hfq	UP	-	UP	-	-	-
YPK_2961	hypothetical protein	UP	UP	DOWN	DOWN	-	DOWN
YPK_4198	hypothetical protein	UP	-	UP	-	-	-
YPK_3361	rplS 50S ribosomal protein L19	UP	-	UP	-	-	-
YPK_1740	cold-shock DNA-binding domain-containingprotein	UP	-	UP	DOWN	DOWN	DOWN
YPK_2389	hypothetical protein	UP	-	-	DOWN	DOWN	-
YPK_1087	hypothetical protein	UP	UP	-	DOWN	DOWN	DOWN
YPK_0132	hypothetical protein	UP	-	DOWN	-	DOWN	DOWN
YPK_1602	DNA starvation/stationary phase protectionprotein Dps	UP	UP	DOWN	DOWN	-	UP
YPK_2733	grxA glutaredoxin	UP	UP	UP	DOWN	-	DOWN
YPK_4035	thioredoxin	UP	-	UP	DOWN	DOWN	DOWN
YPK_2881	hypothetical protein	UP	UP	-	-	-	-
YPK_2390	fliE flagellar hook-basal body protein FliE	UP	UP	UP	-	DOWN	DOWN
YPK_2438	ferroxidase	UP	UP	UP	DOWN	-	UP
YPK_1876	transcriptional regulator SlyA	UP	UP	DOWN	DOWN	UP	DOWN
YPK_0605	hypothetical protein	UP	-	-	-	-	-
YPK_3729	rbfA ribosome-binding factor A	UP	UP	UP	-	-	-
YPK_4171	hypothetical protein	UP	-	-	-	-	-
YPK_2049	outer membrane protein W	UP	UP	-	-	-	-
YPK_4013	cyaY frataxin-like protein	UP	-	-	-	DOWN	-
YPK_0652	hypothetical protein	UP	-	UP	-	-	-
YPK_2381	flagellin	UP	UP	UP	DOWN	DOWN	DOWN
YPK_3244	cytochrome o ubiquinol oxidase subunit IV	UP	-	UP	-	-	-
YPK_0300	rpsE 30S ribosomal protein S5	UP	-	UP	UP	UP	-
YPK_0528	hypothetical protein	UP	-	UP	-	UP	-
YPK_3937	rfaH transcriptional activator RfaH	UP	-	-	-	-	-
YPK_4216	flavodoxin	UP	UP	UP	-	DOWN	-
YPK_1277	scaffold protein	UP	-	-	-	-	-
YPK_0012	heat shock chaperone IbpB	UP	-	DOWN	DOWN	-	DOWN
YPK_1069	frr ribosome recycling factor	UP	-	UP	-	-	-
YPK_2310	XRE family transcriptional regulator	UP	-	-	-	-	-
YPK_0037	molybdopterin oxidoreductase Fe4S4 region	UP	-	-	-	-	-
YPK_2980	smpB SsrA-binding protein	UP	UP	-	-	-	-
YPK_1750	purine-binding chemotaxis protein	UP	UP	UP	-	DOWN	DOWN
YPK_0248	cAMP-regulatory protein	UP	UP	UP	-	-	DOWN
YPK_3714	sterol-binding domain-containing protein	UP	UP	UP	-	-	-
YPK_3512	lpxC UDP-3-O-[3-hydroxymyristoyl] N-acetylglucosaminedeacetylas	UP	-	-	-	UP	UP
YPK_3238	hypothetical protein	UP	UP	-	DOWN	DOWN	DOWN
YPK_1295	hypothetical protein	UP	-	UP	-	-	UP
YPK_3781	rplI 50S ribosomal protein L9	UP	-	UP	-	-	-
YPK_2969	sdhB succinate dehydrogenase iron-sulfur subunit	UP	-	-	UP	UP	-
YPK_1172	pyrrolidone-carboxyPersistency peptidase	UP	-	UP	-	-	-
YPK_0334	nusG transcription antitermination protein NusG	UP	UP	UP	-	-	-
YPK_2380	fliA flagellar biosynthesis sigma factor	UP	UP	UP	-	DOWN	DOWN
YPK_1071	undecaprenyl pyrophosphate synthase	UP	-	UP	-	-	-
YPK_4187	phosphatase	UP	UP	-	-	-	UP
YPK_3397	DeoR family transcriptional regulator	UP	UP	DOWN	-	-	-
YPK_0773	hypothetical protein	UP	-	-	-	-	-
YPK_3775	adenosine-3'(2'),5'-bisphosphate nucleotidase	UP	UP	-	-	DOWN	-
YPK_2175	2-dehydro-3-deoxyphosphooctonate aldolase	UP	-	UP	-	-	-
YPK_0322	hexapaptide repeat-containing transferase	UP	UP	UP	-	-	-
YPK_3814	fumarate reductase iron-sulfur subunit	UP	-	UP	-	-	-
YPK_0381	lamB maltoporin	UP	UP	-	-	UP	DOWN
YPK_0163	putative DNA uptake protein	UP	-	-	-	-	-
YPK_4113	glycerol kinase	UP	-	-	UP	UP	DOWN
YPK_2954	tol-pal system protein YbgF	UP	-	UP	-	UP	UP
YPK_1529	truA tRNA pseudouridine synthase A	UP	UP	UP	-	-	-
YPK_0642	putative signal transduction protein	UP	-	UP	-	UP	-
YPK_0057	xylF D-xylose transporter subunit XylF	UP	UP	DOWN	-	-	-
YPK_1104	hypothetical protein	UP	-	-	-	-	-
YPK_2761	transcriptional regulator CadC	UP	UP	DOWN	DOWN	-	DOWN
YPK_1682	rpmF 50S ribosomal protein L32	UP	UP	UP	DOWN	DOWN	DOWN
YPK_1609	hypothetical protein	UP	-	-	-	-	-
YPK_2568	folE GTP cyclohydrolase I	UP	-	UP	-	-	-
YPK_1036	thyA thymidyPersistency synthase	UP	-	UP	-	-	-

YPK_3187	glycosyl transferase family protein	UP	-	-	DOWN	DOWN	-
YPK_3773	hypothetical protein	UP	-	DOWN	-	UP	-
YPK_3735	folP dihydropteroate synthase	UP	-	-	-	-	-
YPK_0158	intramembrane serine protease GlpG	UP	-	UP	-	DOWN	DOWN
YPK_4112	MIP family channel protein	UP	-	-	-	-	DOWN
YPK_0378	malE maltose ABC transporter periplasmic protein	UP	UP	UP	-	UP	-
YPK_1681	hypothetical protein	UP	UP	UP	DOWN	DOWN	DOWN
YPK_3590	rpsT 30S ribosomal protein S20	UP	UP	UP	DOWN	DOWN	DOWN
YPK_1811	fructosamine kinase	UP	UP	DOWN	-	DOWN	UP
YPK_3779	opacity-associated protein A	UP	-	-	-	-	-
YPK_2185	hypothetical protein	UP	UP	DOWN	-	UP	-
YPK_1190	era GTP-binding protein Era	UP	-	UP	-	-	-
YPK_2649	porin	UP	UP	UP	-	DOWN	DOWN
YPK_3300	Na(+)-translocating NADH-quinone reductasesubunit F	UP	UP	UP	-	-	-
YPK_3264	tgt queuine tRNA-ribosyltransferase	UP	UP	UP	-	-	-
YPK_3281	hypothetical protein	UP	UP	UP	-	DOWN	DOWN
YPK_0001	dnaA chromosomal replication initiation protein	UP	UP	UP	-	-	-
YPK_3526	mraW S-adenosyl-methyltransferase MraW	UP	-	UP	-	-	-
YPK_2187	GTP-dependent nucleic acid-binding protein EngD	UP	-	UP	-	-	-
YPK_1431	cell division protein ZipA	UP	UP	-	DOWN	DOWN	DOWN
YPK_3826	anaerobic C4-dicarboxyPersistency transporter	UP	-	UP	-	-	-
YPK_0667	dkgA 2,5-diketo-D-gluconate reductase A	UP	-	-	-	-	-
YPK_0854	mechanosensitive ion channel MscS	UP	-	UP	-	-	-
YPK_1507	hypothetical protein	UP	UP	-	DOWN	DOWN	-
YPK_1141	voltage-gated potassium channel	UP	UP	DOWN	-	-	-
YPK_1822	rpmI 50S ribosomal protein L35	UP	UP	UP	-	-	-
YPK_1532	bifunctional folylpolyglutamate synthase/dihydrofoPersistency syntha	UP	-	UP	-	UP	UP
YPK_2441	hypothetical protein	UP	UP	DOWN	DOWN	DOWN	UP
YPK_1276	cysteine desulfurase	UP	-	UP	-	-	-
YPK_4153	rpmG 50S ribosomal protein L33	UP	UP	UP	DOWN	DOWN	DOWN
YPK_3772	hypothetical protein	UP	UP	-	-	-	-
YPK_4221	F0F1 ATP synthase subunit C	UP	-	UP	-	-	DOWN
YPK_1056	hypothetical protein	UP	-	-	-	UP	-
YPK_1535	amidophosphoribosyltransferase	UP	-	UP	-	-	-
YPK_2508	mandePersistency racemase/muconate lactonizing protein	UP	-	DOWN	-	UP	UP
YPK_3263	yajC preprotein translocase subunit YajC	UP	UP	UP	-	DOWN	DOWN
YPK_3993	hypothetical protein	UP	-	-	-	-	-
YPK_3767	inorganic pyrophosphatase	UP	UP	UP	DOWN	DOWN	DOWN
YPK_3178	phosphomannomutase	UP	UP	-	-	-	-
YPK_4051	ketol-acid reductoisomerase	UP	-	UP	-	-	-
YPK_0465	rod shape-determining protein MreB	UP	-	UP	-	-	-
YPK_3010	glutamate and aspartate transporter subunit	UP	UP	DOWN	-	-	DOWN
YPK_0546	hypothetical protein	UP	UP	-	-	-	-
YPK_3240	hypothetical protein	UP	UP	-	-	DOWN	DOWN
YPK_1826	ihfA integration host factor subunit alpha	UP	UP	-	-	-	-
YPK_2405	hypothetical protein	UP	-	-	DOWN	-	-
YPK_2963	cytochrome d ubiquinol oxidase subunit II	UP	UP	UP	-	UP	-
YPK_3369	glutamate--cysteine ligase	UP	-	UP	-	-	UP
YPK_1863	superoxide dismutase	UP	UP	DOWN	-	-	-
YPK_2691	infA translation initiation factor IF-1	UP	-	UP	-	-	DOWN
YPK_0292	rpsQ 30S ribosomal protein S17	UP	-	UP	-	-	-
YPK_1601	hypothetical protein	UP	UP	UP	-	UP	-
YPK_3943	ubiB putative ubiquinone biosynthesis protein UbiB	UP	-	UP	-	-	-
YPK_1725	hypothetical protein	UP	UP	-	DOWN	DOWN	DOWN
YPK_3404	tartrate/fumarate subfamily Fe-S typehydro-lyase subunit beta	UP	-	UP	UP	UP	-
YPK_3372	carbon storage regulator	UP	UP	UP	DOWN	DOWN	-
YPK_0305	rpsM 30S ribosomal protein S13	UP	-	UP	UP	UP	UP
YPK_3507	hypothetical protein	UP	-	-	-	-	-
YPK_4170	hypothetical protein	UP	-	-	-	-	-
YPK_3231	transcriptional regulator HU subunit beta	UP	-	UP	DOWN	DOWN	DOWN
YPK_1593	O-succinylbenzoic acid--CoA ligase	UP	-	-	-	-	-
YPK_3035	hypothetical protein	UP	-	DOWN	DOWN	DOWN	DOWN
YPK_3230	peptidyl-prolyl cis-trans isomerase D	UP	UP	UP	-	-	-
YPK_0293	rplN 50S ribosomal protein L14	UP	-	UP	-	-	-
YPK_4249	rpmH 50S ribosomal protein L34	UP	-	UP	DOWN	DOWN	-
YPK_3295	aminoacyl-histidine dipeptidase	UP	-	UP	-	-	-
YPK_3756	rpmA 50S ribosomal protein L27	UP	-	UP	-	DOWN	DOWN
YPK_0276	30S ribosomal protein S7	UP	-	UP	-	-	-
YPK_3838	IS1 transposase	UP	UP	-	-	DOWN	-
YPK_2534	hypothetical protein	UP	-	-	-	-	-
YPK_3203	hypothetical protein	UP	-	-	DOWN	-	DOWN
YPK_0287	rpsS 30S ribosomal protein S19	UP	-	UP	UP	UP	-
YPK_0146	signal transduction histidine kinase LytS	UP	-	-	-	-	-
YPK_1886	tppB putative tripeptide transporter permease	UP	-	UP	-	-	DOWN
YPK_2933	6-phosphogluconolactonase	UP	-	UP	-	-	-
YPK_4111	hypothetical protein	UP	UP	UP	DOWN	-	-
YPK_1717	hypothetical protein	UP	-	-	-	-	-
YPK_3932	proline dipeptidase	UP	-	UP	-	-	-
YPK_1745	transcriptional activator FlhD	UP	-	UP	-	DOWN	DOWN
YPK_1767	hypothetical protein	UP	-	-	-	DOWN	-
YPK_3061	hypothetical protein	UP	UP	-	-	UP	-
YPK_1673	bssS biofilm formation regulatory protein BssS	UP	UP	DOWN	-	-	-
YPK_2048	transport-associated	UP	UP	DOWN	-	UP	UP
YPK_2621	heat shock protein HspQ	UP	-	-	-	-	-
YPK_3028	tatE twin arginine translocase protein A	UP	-	-	-	UP	UP

YPK_2425	flgB flagellar basal body rod protein FlgB	UP	UP	UP	-	DOWN	DOWN
YPK_1183	anti-RNA polymerase sigma factor SigE	UP	-	UP	-	-	UP
YPK_2059	hypothetical protein	UP	UP	-	-	-	DOWN
YPK_4227	atpC F0F1 ATP synthase subunit epsilon	UP	-	UP	-	-	DOWN
YPK_3837	hypothetical protein	UP	-	-	DOWN	DOWN	DOWN
YPK_3474	aspartate alpha-decarboxylase	UP	DOWN	UP	-	DOWN	UP
YPK_3509	preprotein translocase subunit SecA	UP	-	UP	-	-	-
YPK_2977	hypothetical protein	UP	-	UP	-	-	-
YPK_1877	17 kDa surface antigen	UP	-	DOWN	-	-	-
YPK_2802	colicin D	UP	UP	-	-	UP	-
YPK_2489	hypothetical protein	UP	-	-	DOWN	DOWN	-
YPK_4075	hypothetical protein	UP	UP	-	-	-	-
YPK_2692	clpA ATP-dependent Clp protease ATP-binding subunit	UP	-	DOWN	UP	UP	UP
YPK_3738	hypothetical protein	UP	UP	UP	DOWN	DOWN	DOWN
YPK_1163	deoxyribodipyrimidine photolyase	UP	UP	-	-	-	-
YPK_2474	cold shock-like protein CspC	UP	-	UP	DOWN	DOWN	DOWN
YPK_2695	macrolide transporter ATP-binding /permease	UP	-	-	-	UP	-
YPK_3151	hypothetical protein	UP	-	UP	-	-	-
YPK_3234	clpP ATP-dependent Clp protease proteolytic subunit	UP	-	UP	UP	UP	-
YPK_0286	rplB 50S ribosomal protein L2	UP	-	UP	UP	UP	-
YPK_3237	transcriptional regulator BolA	UP	UP	-	-	-	-
YPK_3782	rpsR 30S ribosomal protein S18	UP	-	UP	-	-	-
YPK_1140	hdeB acid-resistance protein	UP	UP	DOWN	-	-	-
YPK_4063	hypothetical protein	UP	-	UP	DOWN	DOWN	-
YPK_1492	alkylphosphonate utilization operon proteinPhnA	UP	UP	-	DOWN	-	DOWN
YPK_3686	extracellular solute-binding protein	UP	-	-	-	-	UP
YPK_2482	hypothetical protein	UP	UP	-	DOWN	DOWN	DOWN
YPK_3087	hypothetical protein	UP	UP	-	-	-	-
YPK_3364	rpsP 30S ribosomal protein S16	UP	-	UP	-	-	-
YPK_1355	arsenate reductase	UP	-	-	UP	UP	UP
YPK_3852	hypothetical protein	UP	-	-	-	-	-
YPK_1818	hypothetical protein	UP	-	UP	-	DOWN	DOWN
YPK_2804	hypothetical protein	UP	-	DOWN	-	UP	DOWN
YPK_1367	hypothetical protein	UP	-	-	-	-	-
YPK_2448	hypothetical protein	UP	UP	-	DOWN	DOWN	-
YPK_2158	integral membrane protein MviN	UP	-	-	-	-	-
YPK_3527	cell division protein MraZ	UP	UP	UP	-	-	-
YPK_2846	DNA gyrase subunit A	UP	-	UP	-	UP	-
YPK_3942	twin arginine-targeting protein translocase	UP	UP	-	-	-	-
YPK_0539	LppC family lipoprotein	UP	-	-	-	-	-
YPK_3850	single-stranded DNA-binding protein	UP	-	UP	-	DOWN	-
YPK_0200	hypothetical protein	UP	UP	-	-	-	-
YPK_3897	hypothetical protein	UP	UP	-	-	-	-
YPK_0492	cytochrome b562	UP	-	DOWN	-	DOWN	-
YPK_0868	glycine cleavage system protein H	UP	-	UP	-	DOWN	DOWN
YPK_2437	copper resistance protein CopC	UP	-	-	-	-	-
YPK_1062	hypothetical protein	UP	UP	-	-	DOWN	-
YPK_0306	30S ribosomal protein S11	UP	-	UP	UP	UP	UP
YPK_3785	hypothetical protein	UP	DOWN	-	-	-	-
YPK_2471	hypothetical protein	UP	UP	-	DOWN	DOWN	DOWN
YPK_3821	hypothetical protein	UP	UP	UP	-	DOWN	-
YPK_2177	invasion gene expression up-regulator SirB	UP	-	UP	-	-	-
YPK_2562	maPersistency dehydrogenase	UP	-	-	-	UP	-
YPK_1676	antibiotic biosynthesis monooxygenase	UP	UP	DOWN	DOWN	DOWN	DOWN
YPK_4146	rfaD ADP-L-glycero-D-manno-heptose-6-epimerase	UP	UP	UP	-	-	-
YPK_0828	hypothetical protein	UP	-	UP	-	-	-
YPK_2294	hypothetical protein	UP	-	-	-	-	-
YPK_2363	TrpR binding protein WrbA	UP	-	-	-	-	UP
YPK_0836	Holliday junction resolvase-like protein	UP	-	UP	-	-	-
YPK_2025	hypothetical protein	UP	-	DOWN	DOWN	DOWN	-
YPK_2958	colicin uptake protein TolR	UP	-	UP	-	-	-
YPK_2018	hypothetical protein	UP	UP	DOWN	-	-	-
YPK_2500	hypothetical protein	UP	-	UP	-	DOWN	-
YPK_1352	upp uracil phosphoribosyltransferase	UP	-	UP	-	-	-
YPK_3680	DNA polymerase III subunit chi	UP	-	-	-	-	-
YPK_0829	hypothetical protein	UP	-	UP	-	-	-
YPK_3019	hypothetical protein	UP	-	UP	-	-	-
YPK_4096	transcriptional repressor protein MetJ	UP	UP	UP	-	DOWN	DOWN
YPK_3362	trmD tRNA (guanine-N(1)-)-methyltransferase	UP	-	UP	-	-	-
YPK_2781	hypothetical protein	UP	UP	UP	DOWN	UP	DOWN
YPK_4248	rnpA ribonuclease P	UP	-	UP	-	-	-
YPK_0445	17 kDa surface antigen	UP	-	-	-	DOWN	-
YPK_0736	17 kDa surface antigen	UP	-	UP	-	-	-
YPK_3146	hypothetical protein	UP	UP	UP	-	-	-
YPK_2264	putative lipoprotein	UP	-	-	-	-	UP
YPK_1490	type VI secretion system lysozyme-like protein	UP	-	DOWN	-	-	-
YPK_0010	hypothetical protein	UP	UP	-	-	-	-
YPK_0283	rplC 50S ribosomal protein L3	UP	-	UP	UP	UP	-
YPK_2760	hypothetical protein	UP	UP	DOWN	DOWN	-	DOWN
YPK_2129	endoribonuclease L-PSP	UP	UP	UP	DOWN	DOWN	DOWN
YPK_3481	hypothetical protein	UP	-	-	-	DOWN	DOWN
YPK_3759	MuA-transposase/repressor protein CIDNA-binding	UP	-	-	-	-	-
YPK_0992	PTS system mannose/fructose/sorbose familytransporter subunit IIB	UP	-	-	-	-	-
YPK_3606	two-component response regulator	UP	-	UP	-	DOWN	-
YPK_0538	hypothetical protein	UP	-	UP	-	-	-

YPK_3549	hypothetical protein	UP	UP	-	-	-	UP
YPK_0172	ompR osmolarity response regulator	UP	-	UP	-	UP	-
YPK_3513	cell division protein FtsZ	UP	-	UP	UP	UP	UP
YPK_1426	PTS system glucose-specific transporter	UP	-	UP	-	-	-
YPK_1221	XRE family transcriptional regulator	UP	-	DOWN	-	-	-
YPK_0527	ClpXP protease specificity-enhancing factor	UP	-	UP	-	-	-
YPK_2552	uridine kinase	UP	-	UP	-	-	-
YPK_3586	FKBP-type peptidylprolyl isomerase	UP	-	UP	-	-	-
YPK_3155	phosphoribosylaminoimidazole carboxylasecatalytic subunit	UP	-	-	-	DOWN	DOWN
YPK_3723	hypothetical protein	UP	-	-	-	-	-
YPK_1151	replication initiation regulator SeqA	UP	-	-	-	-	-
YPK_2990	flavodoxin FldA	UP	-	UP	DOWN	DOWN	DOWN
YPK_1033	dinucleoside polyphosphate hydrolase	UP	UP	UP	-	-	-
YPK_2523	hypothetical protein	UP	UP	-	-	DOWN	-
YPK_1701	hypothetical protein	UP	-	-	-	-	-
YPK_3808	oligoribonuclease	UP	-	UP	-	-	-
YPK_1568	NADH dehydrogenase subunit J	UP	-	UP	-	-	-
YPK_3363	rimM 16S rRNA-processing protein RimM	UP	-	UP	-	-	-
YPK_3813	fumarate reductase flavoprotein subunit	UP	-	UP	-	-	-
YPK_0549	hypothetical protein	UP	-	-	-	-	-
YPK_2709	chorismate mutase	UP	UP	-	-	UP	UP
YPK_0448	carboxymuconolactone decarboxylase	UP	-	-	-	-	-
YPK_1563	NADH dehydrogenase subunit E	UP	-	UP	UP	UP	UP
YPK_2669	cmk cytidyPersistency kinase	UP	UP	UP	-	UP	UP
YPK_1097	D,D-heptose 1,7-bisphosphate phosphatase	UP	-	-	-	DOWN	-
YPK_3573	ksgA dimethyladenosine transferase	UP	DOWN	UP	-	-	-
YPK_3225	cof family hydrolase	UP	-	-	-	-	-
YPK_0249	hypothetical protein	UP	-	UP	-	-	-
YPK_0268	FKBP-type peptidyl-prolyl cis-trans isomerase	UP	UP	UP	-	DOWN	DOWN
YPK_2006	Na(+)-translocating NADH-quinone reductasesubunit E	UP	-	-	-	-	-
YPK_2551	dcd deoxycytidine triphosphate deaminase	UP	-	UP	-	-	-
YPK_2855	catalase	UP	-	UP	-	UP	UP
YPK_1296	outer membrane protein assembly complex subunitYfgL	UP	-	UP	-	-	UP
YPK_0581	hypothetical protein	UP	-	DOWN	-	-	-
YPK_3301	Na(+)-translocating NADH-quinone reductasesubunit E	UP	UP	UP	-	-	-
YPK_4210	D-ribose pyranase	UP	-	-	-	-	-
YPK_3746	putative phage terminase, small subunit	UP	-	-	-	-	UP
YPK_1883	glutathionine S-transferase	UP	-	DOWN	-	-	UP
YPK_2126	Slp family outer membrane lipoprotein	UP	-	DOWN	-	-	-
YPK_0656	hypothetical protein	UP	-	UP	-	-	-
YPK_3683	hypothetical protein	UP	-	UP	UP	-	UP
YPK_0855	arginine exporter protein	UP	-	-	-	-	-
YPK_1007	general secretion pathway protein G	UP	-	-	-	-	-
YPK_2913	DNA-binding transcriptional activator XapR	UP	-	-	-	-	-
YPK_3302	Na(+)-translocating NADH-quinone reductasesubunit D	UP	UP	UP	-	UP	-
YPK_3191	glucose-1-phosphate cytidylyltransferase	UP	UP	UP	-	UP	-
YPK_3562	type VI secretion system lysozyme-like protein	UP	-	-	UP	UP	UP
YPK_2309	hypothetical protein	UP	UP	-	-	UP	UP
YPK_3163	hypothetical protein	UP	UP	-	-	-	-
YPK_1656	autoinducer synthesis protein	UP	-	UP	DOWN	DOWN	DOWN
YPK_2632	hypothetical protein	UP	-	DOWN	-	-	-
YPK_0638	putative glycerol-3-phosphate acyltransferasePlsY	UP	UP	-	DOWN	DOWN	DOWN
YPK_0792	autoinducer synthesis protein	UP	UP	DOWN	DOWN	-	DOWN
YPK_3291	xanthine-guanine phosphoribosyltransferase	UP	-	UP	DOWN	DOWN	DOWN
YPK_0649	ribB 3,4-dihydroxy-2-butanone 4-phosphate synthase	UP	-	UP	-	-	UP
YPK_3941	twin-arginine translocation protein subunitTatB	UP	-	UP	-	-	-
YPK_0373	pgi glucose-6-phosphate isomerase	UP	-	UP	-	-	-
YPK_3978	cell division protein FtsE	UP	-	UP	-	-	-
YPK_3579	hypothetical protein	UP	-	-	-	-	-
YPK_4232	pstC phosphate transporter permease subunit PstC	UP	-	-	-	-	-
YPK_0281	bacterioferritin	UP	UP	DOWN	-	-	UP
YPK_1948	nucleotidase	UP	-	UP	-	-	-
YPK_0864	2-octaprenyl-6-methoxyphenyl hydroxylase	UP	-	UP	-	-	-
YPK_2959	colicin uptake protein TolQ	UP	-	UP	-	-	-
YPK_2388	death-on-curing family protein	UP	UP	-	DOWN	DOWN	-
YPK_0451	hypothetical protein	UP	-	-	-	-	-
YPK_3270	short chain dehydrogenase	UP	-	-	-	-	-
YPK_3032	cold-shock DNA-binding domain-containingprotein	UP	-	UP	DOWN	DOWN	DOWN
YPK_3060	Hcp1 family type VI secretion system effector	UP	UP	-	-	UP	-
YPK_1090	hypothetical protein	UP	-	-	-	UP	-
YPK_1817	multiple drug resistance protein MarC	UP	-	-	-	-	-
YPK_2429	invasin region 3	UP	UP	-	-	UP	-
YPK_1173	uracil-DNA glycosylase	UP	-	UP	-	-	-
YPK_2423	flgD flagellar basal body rod modification protein	UP	-	UP	-	DOWN	DOWN
YPK_1566	NADH dehydrogenase subunit H	UP	-	UP	-	-	-
YPK_0463	quinone oxidoreductase	UP	-	UP	-	-	-
YPK_2419	flgH flagellar basal body L-ring protein	UP	UP	UP	-	DOWN	DOWN
YPK_4106	ribonuclease activity regulator protein RraA	UP	-	UP	-	-	-
YPK_1743	MgtC/SapB transporter	UP	UP	DOWN	-	-	-
YPK_3935	fre FMN reductase	UP	-	UP	-	-	-
YPK_0696	pili assembly chaperone	UP	-	-	-	-	UP
YPK_3617	OmpA/MotB domain-containing protein	UP	-	-	-	-	-
YPK_2159		UP	-	-	-	-	-
YPK_3245	protoheme IX farnesyltransferase	UP	-	UP	-	-	-
YPK_3524	peptidoglycan glycosyltransferase	UP	UP	-	-	-	-

YPK_3587	lspA lipoprotein signal peptidase	UP	-	UP	-	-	-
YPK_2952	nicotinamide mononucleotide transporter PnuC	UP	-	-	-	UP	-
YPK_0316	def peptide deformylase	UP	-	UP	DOWN	DOWN	DOWN
YPK_3874	integral membrane protein TerC	UP	-	UP	-	UP	-
YPK_3858	tRNA-dihydrouridine synthase A	UP	-	UP	-	-	-
YPK_3440	phosphoadenosine phosphosulfate reductase	UP	-	-	-	-	-
YPK_3563	hypothetical protein	UP	-	UP	UP	UP	UP
YPK_1247	hypothetical protein	UP	-	-	-	-	-
YPK_3015	leuS leucyl-tRNA synthetase	UP	-	UP	UP	UP	UP
YPK_1600	glnH glutamine ABC transporter periplasmic protein	UP	-	DOWN	-	UP	-
YPK_0775	hypothetical protein	UP	-	-	-	-	-
YPK_1606	ompX outer membrane protein X	UP	UP	UP	DOWN	UP	-
YPK_3511	hypothetical protein	UP	UP	-	-	-	-
YPK_1165	putative hydrolase-oxidase	UP	-	-	-	-	-
YPK_3000	UMP phosphatase	UP	-	-	-	-	-
YPK_1944	fumarate/nitrate reduction transcriptionalregulator	UP	-	UP	-	-	-
YPK_3945	ubiE ubiquinone/menaquinone biosynthesismethyltransferase	UP	-	UP	-	-	-
YPK_1735	hypothetical protein	UP	-	-	DOWN	DOWN	-
YPK_2153	copper homeostasis protein CutC	UP	-	-	-	-	-
YPK_3611	NTPase	UP	-	-	-	-	-
YPK_1567	NADH dehydrogenase subunit I	UP	-	UP	-	-	-
YPK_0002	DNA polymerase III subunit beta	UP	-	UP	-	-	-
YPK_1475	fimbrial protein	UP	UP	-	-	UP	DOWN
YPK_0995	DeoR family transcriptional regulator	UP	UP	DOWN	-	DOWN	DOWN
YPK_3160	short chain dehydrogenase	UP	-	-	-	-	-
YPK_1088	YaeQ family protein	UP	-	-	-	-	-
YPK_0380	maltose/maltodextrin transporter ATP-bindingprotein	UP	-	UP	-	UP	DOWN
YPK_1544	phosphodiesterase	UP	UP	-	-	-	-
YPK_3515	cell division protein FtsQ	UP	-	UP	-	-	-
YPK_3796	FtsH protease regulator HflK	UP	-	UP	UP	UP	UP
YPK_2465	PTS system mannose/fructose/sorbose familytransporter subunit IIC	UP	-	UP	-	-	-
YPK_2415	flgL flagellar hook-associated protein FlgL	UP	UP	UP	-	DOWN	DOWN
YPK_2376	cystine transporter subunit	UP	-	UP	-	-	DOWN
YPK_0509	3-deoxy-D-manno-octulosonate 8-phosphatephosphatase	UP	-	-	-	UP	UP
YPK_0227	hypothetical protein	UP	UP	UP	-	-	-
YPK_1128	ABC transporter-like protein	UP	UP	UP	-	-	UP
YPK_2223	N-formylglutamate amidohydrolase	UP	UP	DOWN	-	-	-
YPK_2426	flgA flagellar basal body P-ring biosynthesis proteinFlgA	UP	UP	UP	-	DOWN	DOWN
YPK_1536	3-octaprenyl-4-hydroxybenzoate carboxy-lyase	UP	UP	-	-	-	-
YPK_1698	membrane lipoprotein lipid attachment site	UP	-	UP	-	-	-
YPK_3872	stress protein	UP	-	UP	-	-	-
YPK_0645	bifunctional heptose 7-phosphate kinase/heptose1-phosphate adenyl	UP	-	UP	-	-	-
YPK_4136	cysE serine acetyltransferase	UP	-	-	-	-	-
YPK_2178	N5-glutamine S-adenosyl-L-methionine-dependentmethyltransferase	UP	UP	UP	-	-	-
YPK_2211	hypothetical protein	UP	-	-	-	-	-
YPK_4144	tdh L-threonine 3-dehydrogenase	UP	-	UP	-	UP	-
YPK_2038	cob(I)yrinic acid a,c-diamideadenosyltransferase	UP	-	UP	-	-	UP
YPK_1080	rnhB ribonuclease HII	UP	-	UP	-	-	-
YPK_0437	putative glutathione S-transferase YghU	UP	-	-	-	-	UP
YPK_1591	naphthoate synthase	UP	-	-	-	-	-
YPK_2676	formate transporter	UP	-	UP	DOWN	DOWN	DOWN
YPK_3188	NAD-dependent epimerase/dehydratase	UP	UP	-	-	-	-
YPK_1625	hypothetical protein	UP	-	-	-	-	-
YPK_4072	glutamate racemase	UP	-	-	-	-	-
YPK_4161	hypothetical protein	UP	UP	UP	-	-	-
YPK_4147	ADP-heptose--LPS heptosyltransferase	UP	-	UP	-	-	-
YPK_3841	transcriptional activator RhaR	UP	-	-	-	-	DOWN
YPK_3632	hypothetical protein	UP	UP	DOWN	-	UP	UP
YPK_2144	ruvA Holliday junction DNA helicase RuvA	UP	-	-	-	-	-
YPK_0510	D-arabinose 5-phosphate isomerase	UP	-	-	-	-	-
YPK_3008	transporter-associated protein	UP	DOWN	UP	-	-	-
YPK_3716	peptidase U32	UP	UP	UP	-	-	-
YPK_1784	heat shock protein HtpX	UP	-	-	-	-	-
YPK_4242	hypothetical protein	UP	-	-	-	DOWN	DOWN
YPK_2007	electron transport complex protein RnfB	UP	UP	-	-	-	-
YPK_0447	3-hydroxyisobutyrate dehydrogenase	UP	UP	-	-	-	UP
YPK_0990	PTS system mannose/fructose/sorbose familytransporter subunit IID	UP	-	UP	-	-	-
YPK_3233	clpX ATP-dependent protease ATP-binding subunit ClpX	UP	-	UP	-	-	-
YPK_0665	repressor protein for FtsI	UP	-	-	-	-	-
YPK_0655	nudF ADP-ribose pyrophosphatase NudF	UP	-	UP	-	-	-
YPK_2237	N-acetyltransferase GCN5	UP	-	-	-	-	-
YPK_1362	dihydrodipicolinate synthase	UP	-	UP	-	-	-
YPK_2068	binding-protein-dependent transport system innermembrane protein	UP	UP	-	-	-	-
YPK_0569	stress protein	UP	UP	UP	-	-	-
YPK_2111	disulfide bond formation protein B	UP	-	-	-	-	-
YPK_3280	rdgC recombination associated protein	UP	-	UP	-	-	-
YPK_1759	chemotaxis regulator CheZ	UP	UP	-	-	DOWN	DOWN
YPK_1372	succinyl-diaminopimePersistency desuccinylase	UP	-	UP	-	-	-
YPK_3571	peptidyl-prolyl cis-trans isomerase SurA	UP	-	UP	-	-	-
YPK_3616	diguanyPersistency cyclase	UP	-	-	-	-	-
YPK_3944	sterol-binding domain-containing protein	UP	-	UP	-	-	-
YPK_0534	isoprenoid biosynthesis protein withamidotransferase-like domain	UP	-	-	-	UP	UP
YPK_3603	homoserine kinase	UP	-	-	-	-	-
YPK_1513	N5-glutamine S-adenosyl-L-methionine-dependentmethyltransferase	UP	-	UP	-	-	-
YPK_2356	response regulator	UP	-	-	-	-	UP

YPK_1451	hypothetical protein	UP	UP	-	-	DOWN	-
YPK_0134	LacI family transcriptional regulator	UP	-	-	-	-	-
YPK_0865	hypothetical protein	UP	-	-	-	-	-
YPK_3297	glycerophosphoryl diester phosphodiesterase	UP	-	-	-	UP	-
YPK_4002	magnesium/nickel/cobalt transporter CorA	UP	-	UP	-	-	-
YPK_0458	acetyl-CoA carboxylase biotin carboxylasesubunit	UP	-	UP	-	-	-
YPK_0114	glutathione reductase	UP	-	UP	-	-	-
YPK_1298	auxin efflux carrier	UP	-	-	-	-	-
YPK_1923	DeoR family transcriptional regulator	UP	-	-	-	-	-
YPK_3882	hypothetical protein	UP	-	-	-	-	UP
YPK_0453	tRNA-dihydrouridine synthase B	UP	-	UP	-	DOWN	DOWN
YPK_3027	lipoyl synthase	UP	-	UP	-	-	-
YPK_1868	ribonuclease T	UP	-	UP	-	-	-
YPK_2623	hypothetical protein	UP	UP	-	-	UP	-
YPK_1815	periplasmic solute binding protein	UP	-	-	-	-	-
YPK_1444	glk glucokinase	UP	-	UP	-	-	DOWN
YPK_3621	DNA repair protein RadA	UP	-	UP	-	-	-
YPK_4006	TetR family transcriptional regulator	UP	-	-	-	-	-
YPK_4206	GntR family transcriptional regulator	UP	-	-	-	-	-
YPK_0633	hypothetical protein	UP	UP	-	DOWN	DOWN	DOWN
YPK_3856	quinone oxidoreductase	UP	-	-	-	-	-
YPK_1906	extracellular solute-binding protein	UP	-	-	-	UP	UP
YPK_2664	lpxK tetraacyldisaccharide 4'-kinase	UP	-	-	-	-	-
YPK_0543	putative glutathione S-transferase	UP	-	-	-	-	-
YPK_4197	serine/threonine protein kinase	UP	-	UP	-	-	-
YPK_1358	hypothetical protein	UP	-	-	-	-	-
YPK_2936	modB molybdate ABC transporter permease	UP	-	-	-	-	-
YPK_0531	putative radical SAM protein	UP	-	UP	-	-	-
YPK_3192	CDP-6-deoxy-delta-3,4-glucoseen reductase	UP	UP	-	-	-	-
YPK_0900	small terminase subunit	UP	-	-	-	-	-
YPK_2242	putative endopeptidase	UP	UP	-	-	-	-
YPK_1918	NAD-binding D-isomer specific 2-hydroxyaciddehydrogenase	UP	-	UP	-	-	-
YPK_1418	putative peptidase	UP	-	-	-	-	-
YPK_0559	oxidoreductase domain-containing protein	UP	-	UP	-	-	-
YPK_1105	mltD membrane-bound lytic murein transglycosylase D	UP	UP	UP	-	-	-
YPK_2414	DeoR family transcriptional regulator	UP	-	-	-	-	-
YPK_2690	aat leucyl/phenylalanyl-tRNA--protein transferase	UP	-	-	-	UP	-
YPK_0641	cca multifunctional tRNA nucleotidyltransferase/2'3'-cyclicphosphod	UP	-	-	-	UP	UP
YPK_2246	ABC transporter-like protein	UP	-	-	-	-	UP
YPK_0646	inner membrane protein YjeH	UP	-	-	-	-	-
YPK_3653	autoinducer AI-2 ABC transporter periplasmicAI-2-binding protein	UP	-	DOWN	-	-	-
YPK_0025	putative lipoprotein	UP	-	UP	-	-	UP
YPK_2702	HCP oxidoreductase	UP	-	-	-	-	-
YPK_0263	glycosyl transferase family protein	UP	-	-	-	-	-
YPK_1893	pspF phage shock protein operon transcriptionalactivator	UP	-	-	-	-	-
YPK_3013	ABC transporter-like protein	UP	-	DOWN	-	-	-
YPK_0506	putative ABC transporter ATP-binding proteinYhbG	UP	-	UP	-	-	-
YPK_1191	recO DNA repair protein RecO	UP	-	-	-	-	-
YPK_1683	putative glycerol-3-phosphate acyltransferasePlsX	UP	-	UP	-	-	-
YPK_0663	1-acyl-sn-glycerol-3-phosphate acyltransferase	UP	-	-	-	-	-
YPK_2089	selenophosphate synthetase	UP	-	-	-	DOWN	-
YPK_2563	hypothetical protein	UP	-	-	-	-	-
YPK_0131	two component transcriptional regulator	UP	UP	-	-	-	-
YPK_2498	TRAP dicarboxyPersistency transporter subunit DctM	UP	UP	DOWN	-	-	-
YPK_2780	GntR family transcriptional regulator	UP	UP	-	-	UP	-
YPK_3296	DNA polymerase IV	UP	-	-	-	-	-
YPK_3326	ABC transporter-like protein	UP	UP	-	-	-	-
YPK_3207	RND family efflux transporter MFP subunit	UP	-	-	-	-	-
YPK_2953	quinolinate synthetase	UP	-	-	-	UP	-
YPK_1258	hypothetical protein	UP	-	UP	-	-	-
YPK_3518	murG undecaprenyldiphospho-muramoylpentapeptidebeta-N- acety	UP	-	-	-	-	-
YPK_3925	Integrase catalytic subunit	UP	-	-	-	-	UP
YPK_2421	flgF flagellar basal body rod protein FlgF	UP	UP	UP	UP	DOWN	DOWN
YPK_0976	lacI lac repressor	UP	-	-	-	-	-
YPK_3190	CDP-glucose 4,6-dehydratase	UP	UP	UP	UP	UP	UP
YPK_0157	DNA-binding transcriptional repressor GlpR	UP	-	UP	-	-	-
YPK_1180	methyltransferase small	UP	UP	-	-	-	-
YPK_3005	(dimethylallyl)adenosine tRNAmethylthiotransferase	UP	-	DOWN	-	UP	UP
YPK_3299	ApbE family lipoprotein	UP	UP	UP	-	-	-
YPK_2028	phosphatidylglycerophosphatase B	UP	-	-	-	-	-
YPK_3950	uridine phosphorylase	UP	-	DOWN	UP	-	-
YPK_3492	pdhR transcriptional regulator PdhR	UP	-	UP	-	-	DOWN
YPK_2221	histidine utilization repressor	UP	UP	-	-	-	-
YPK_2155	argS arginyl-tRNA synthetase	UP	-	UP	-	-	-
YPK_3734	glmM phosphoglucosamine mutase	UP	UP	UP	-	-	-
YPK_1464	murein hydrolase B	UP	-	-	-	-	-
YPK_1060	tRNA pseudouridine synthase C	UP	-	UP	-	-	-
YPK_1274	RNA methyltransferase	UP	-	-	-	DOWN	DOWN
YPK_2233	calcium/sodium:proton antiporter	UP	DOWN	-	-	DOWN	DOWN
YPK_2880	hypothetical protein	UP	-	-	-	UP	-
YPK_2937	modA molybdate transporter periplasmic protein	UP	UP	-	-	-	-
YPK_2906	NADH:flavin oxidoreductase	UP	-	-	-	UP	-
YPK_0650	hypothetical protein	UP	UP	-	-	-	-
YPK_2420	flgG flagellar basal body rod protein FlgG	UP	UP	UP	-	DOWN	DOWN
YPK_2593	hypothetical protein	UP	-	-	-	-	-

YPK_3736	hflB ATP-dependent metalloprotease	UP	-	UP	-	-	-
YPK_3067	cupin 4 family protein	UP	-	UP	-	-	-
YPK_1527	NAD-binding D-isomer specific 2-hydroxyaciddehydrogenase	UP	-	UP	-	-	-
YPK_1293	ispG 4-hydroxy-3-methylbut-2-en-1-yl diphosphatesynthase	UP	-	UP	-	-	-
YPK_0552	DNA-binding transcriptional repressor ExuR	UP	-	-	-	-	-
YPK_3483	S-adenosylmethionine decarboxylase	UP	-	UP	-	-	-
YPK_4026	TDP-4-oxo-6-deoxy-D-glucose transaminase	UP	-	UP	-	-	-
YPK_2148	aspS aspartyl-tRNA synthetase	UP	-	UP	-	-	-
YPK_1227	hypothetical protein	UP	-	-	-	-	-
YPK_2047	trpA tryptophan synthase subunit alpha	UP	-	-	-	-	-
YPK_2506	lldD L-lactate dehydrogenase	UP	-	-	-	UP	UP
YPK_1953	putative virulence factor SrfB	UP	UP	UP	-	DOWN	DOWN
YPK_1888	ABC transporter-like protein	UP	-	-	-	-	-
YPK_4046	pili assembly chaperone	UP	-	-	-	-	-
YPK_0027	mannitol-1-phosphate 5-dehydrogenase	UP	-	-	-	-	-
YPK_2091	protease 4	UP	-	UP	-	-	-
YPK_0036	formate dehydrogenase accessory protein	UP	-	-	-	-	-
YPK_3740	D-alanyl-D-alanine carboxypeptidase	UP	-	-	-	-	-
YPK_0061	integrase family protein	UP	-	-	-	-	-
YPK_2778	mannonate dehydratase	UP	DOWN	-	-	-	-
YPK_2572	S-formylglutathione hydrolase	UP	-	UP	-	UP	-
YPK_1014	prepilin peptidase	UP	-	-	-	-	-
YPK_3448	mazG nucleoside triphosphate pyrophosphohydrolase	UP	-	-	-	-	-
YPK_3519	cell division protein FtsW	UP	-	-	-	-	-
YPK_3470	pcnB poly(A) polymerase I	UP	-	UP	-	-	-
YPK_2740	serine-type D-Ala-D-Ala carboxypeptidase	UP	-	UP	-	UP	UP
YPK_1864	NLP/P60 protein	UP	-	-	-	-	-
YPK_0502	hypothetical protein	UP	-	UP	-	UP	-
YPK_2866	rhodanese domain-containing protein	UP	-	-	-	UP	-
YPK_3898	hypothetical protein	UP	UP	-	-	-	-
YPK_4229	glucosamine--fructose-6-phosphateaminotransferase	UP	UP	UP	-	-	UP
YPK_0254	phosphoribulokinase	UP	-	-	-	-	-
YPK_3304	Na(+)-translocating NADH-quinone reductasesubunit B	UP	-	UP	-	-	-
YPK_3654	aldolase	UP	-	DOWN	-	-	-
YPK_3999	phospholipase A	UP	UP	UP	-	-	-
YPK_3467	sugar fermentation stimulation protein A	UP	-	-	-	-	-
YPK_3708	binding-protein-dependent transport system innermembrane protein	UP	-	-	-	-	-
YPK_2072	bifunctional acetaldehyde-CoA/alcoholdehydrogenase	UP	-	UP	-	DOWN	DOWN
YPK_1546	PTS system ascorbate-specific transportersubunit IIC	UP	-	UP	-	DOWN	DOWN
YPK_1747	flagellar motor protein MotA	UP	UP	-	-	DOWN	DOWN
YPK_4030	wecC UDP-N-acetyl-D-mannosamine dehydrogenase	UP	-	UP	-	UP	-
YPK_0215	XRE family transcriptional regulator	UP	UP	-	-	-	-
YPK_1324	lipid kinase	UP	-	-	-	UP	-
YPK_2009	AraC family transcriptional regulator	UP	-	-	-	DOWN	-
YPK_2125	long-chain-fatty-acid--CoA ligase	UP	-	DOWN	-	-	-
YPK_2578	molybdopterin biosynthesis protein MoeA	UP	-	UP	-	-	-
YPK_2670	3-phosphoshikimate 1-carboxyvinyltransferase	UP	-	UP	UP	UP	-
YPK_4036	ATP-dependent RNA helicase RhlB	UP	-	UP	-	-	-
YPK_3497	quinolinate phosphoribosyltransferase	UP	-	UP	-	DOWN	-
YPK_3602	threonine synthase	UP	-	-	-	-	-
YPK_0382	maltose regulon periplasmic protein	UP	UP	-	-	-	-
YPK_0660	LysR family transcriptional regulator	UP	UP	-	-	-	-
YPK_3248	2-dehydropantoate 2-reductase	UP	-	-	-	-	-
YPK_1983	major facilitator transporter	UP	-	-	-	UP	-
YPK_2826	Hrp-dependent type III effector protein	UP	-	-	-	-	-
YPK_3176	inosine kinase	UP	UP	-	-	DOWN	-
YPK_1508	fadI 3-ketoacyl-CoA thiolase	UP	-	DOWN	-	UP	-
YPK_3189	DegT/DnrJ/EryC1/StrS aminotransferase	UP	UP	UP	-	UP	-
YPK_3920	gltP glutamate/aspartate:proton symporter	UP	-	-	-	DOWN	DOWN
YPK_1045	N-acetylglutamate synthase	UP	-	-	-	-	-
YPK_1638	hypothetical protein	UP	-	-	-	-	-
YPK_2863	ABC transporter-like protein	UP	-	-	-	UP	-
YPK_3382	transketolase domain-containing protein	UP	-	-	-	-	-
YPK_0119	glutamate dehydrogenase	UP	-	UP	-	-	-
YPK_2184	ribose-phosphate pyrophosphokinase	UP	-	UP	-	-	UP
YPK_0438	hypothetical protein	UP	-	-	-	-	-
YPK_0328	pantothenate kinase	UP	UP	UP	DOWN	DOWN	DOWN
YPK_1168	urea amidolyase-like protein	UP	-	UP	-	-	-
YPK_3170		UP	-	-	-	-	-
YPK_0071	periplasmic binding protein	UP	-	-	-	-	-
YPK_1748	motB flagellar motor protein MotB	UP	UP	-	-	DOWN	DOWN
YPK_3855	replicative DNA helicase	UP	-	UP	-	-	-
YPK_0838	glutathione synthetase	UP	-	UP	-	-	-
YPK_1678	23S rRNA pseudouridyPersistency synthase C	UP	-	UP	-	DOWN	DOWN
YPK_2728	putrescine ABC transporter membrane protein	UP	-	-	-	-	-
YPK_4087	argininosuccinate lyase	UP	-	-	-	-	-
YPK_4192	coproporphyrinogen III oxidase	UP	-	-	-	-	-
YPK_2477	amino acid permease-associated protein	UP	-	UP	-	-	DOWN
YPK_1856	multidrug efflux protein	UP	-	-	-	-	-
YPK_1126	binding-protein-dependent transport system innermembrane protein	UP	-	-	-	-	-
YPK_0313	trkA potassium transporter peripheral membraneprotein	UP	UP	-	-	-	-
YPK_3758	octaprenyl diphosphate synthase	UP	-	UP	-	DOWN	-
YPK_0076	urocanate hydratase	UP	-	DOWN	-	-	-
YPK_3411	cytochrome-c peroxidase	UP	UP	-	-	-	-
YPK_3728	truB tRNA pseudouridine synthase B	UP	-	UP	-	-	-

YPK_0262	glycosyl transferase family protein	UP	-	UP	-	-	DOWN
YPK_4021	putative transport protein YifK	UP	-	-	-	-	-
YPK_3896	LysR family transcriptional regulator	UP	UP	-	-	-	-
YPK_0588	LacI family transcriptional regulator	UP	-	-	-	-	-
YPK_2648	asnC asparaginyl-tRNA synthetase	UP	-	UP	-	UP	-
YPK_3588	ileS isoleucyl-tRNA synthetase	UP	-	UP	-	-	-
YPK_2496	TRAP dicarboxyPersistency transporter subunit DctP	UP	UP	-	-	-	-
YPK_4214	asparagine synthetase AsnA	UP	-	UP	-	DOWN	DOWN
YPK_1437	gltX glutamyl-tRNA synthetase	UP	-	UP	-	-	-
YPK_2014	NAD-dependent epimerase/dehydratase	UP	-	-	-	-	UP
YPK_2766	monosaccharide-transporting ATPase	UP	-	-	-	-	-
YPK_3819	lysine 2,3-aminomutase YodO family protein	UP	-	UP	-	-	UP
YPK_0563	NADH:flavin oxidoreductase	UP	-	-	-	-	-
YPK_2564	mglC beta-methylgalactoside transporter innermembrane protein	UP	-	-	-	-	DOWN
YPK_1737	PIG3 family NAD(P)H quinone oxidoreductase	UP	-	-	UP	UP	UP
YPK_3184	group 1 glycosyl transferase	UP	UP	-	-	-	-
YPK_0779	LacI family transcriptional regulator	UP	-	-	-	-	-
YPK_1463	extracellular solute-binding protein	UP	-	-	-	-	-
YPK_1967	Na+/H+ antiporter NhaC	UP	-	-	-	-	-
YPK_1302	inosine 5'-monophosphate dehydrogenase	UP	-	UP	UP	-	UP
YPK_0486	succinate-semialdehyde dehydrogenase	UP	-	DOWN	-	-	UP
YPK_1164	cyclic-AMP phosphodiesterase	UP	-	-	-	-	-
YPK_1841	phospho-2-dehydro-3-deoxyheptonate aldolase	UP	-	UP	-	-	-
YPK_1757	chemotaxis-specific methylesterase	UP	-	-	-	-	-
YPK_3809	ribosome-associated GTPase	UP	-	UP	-	-	-
YPK_3679	leucyl aminopeptidase	UP	-	UP	-	-	-
YPK_1668	hypothetical protein	UP	UP	-	-	-	-
YPK_3357	phospho-2-dehydro-3-deoxyheptonate aldolase	UP	-	-	-	DOWN	-
YPK_1986	mannose-6-phosphate isomerase	UP	-	UP	-	-	-
YPK_4033	undecaprenyl-phosphatealpha-N-acetylglucosaminyl 1-phosphatetra	UP	UP	-	-	-	-
YPK_1645	CRISPR-associated helicase Cas3 family protein	UP	UP	DOWN	-	UP	-
YPK_3208	hydrophobe/amphiphile efflux-1 (HAE1) familyprotein	UP	-	UP	-	-	-
YPK_1954	hypothetical protein	UP	UP	UP	-	DOWN	DOWN
YPK_0697	fimbrial protein	UP	UP	-	-	-	-
YPK_1844	hypothetical protein	UP	-	-	-	-	-
YPK_1054	putative RNA 2'-O-ribose methyltransferase	UP	-	-	-	-	-
YPK_3040	xylose isomerase domain-containing protein	UP	-	DOWN	-	-	-
YPK_1387	nitrate reductase catalytic subunit	UP	-	-	-	-	-
YPK_2696	macrolide transporter subunit MacA	UP	-	-	-	UP	-
YPK_1303	guaA GMP synthase	UP	-	UP	-	-	UP
YPK_2409	ABC transporter-like protein	UP	-	DOWN	-	-	-
YPK_1672	solA N-methyltryptophan oxidase	UP	-	-	-	-	-
YPK_0586	ABC transporter-like protein	UP	-	DOWN	-	-	-
YPK_1661	mdoG glucan biosynthesis protein G	UP	-	-	-	UP	UP
YPK_4018	putative uroporphyrinogen IIIC-methyltransferase	UP	-	UP	-	UP	-
YPK_1799	ABC transporter-like protein	UP	-	-	-	-	-
YPK_2849	nrdB ribonucleotide-diphosphate reductase subunitbeta	UP	UP	UP	-	-	-
YPK_2811	basepPersistency J family protein	UP	-	DOWN	-	UP	-
YPK_2848	ribonucleotide-diphosphate reductase subunitalpha	UP	UP	UP	-	-	-
YPK_2571	S-(hydroxymethyl)glutathione dehydrogenase/classIII alcohol dehydr	UP	-	UP	UP	UP	-
YPK_3593	chaperone protein DnaJ	UP	-	UP	UP	UP	-
YPK_3128	integrase family protein	UP	-	-	-	-	-
YPK_2841	major facilitator transporter	UP	-	-	-	-	-
YPK_0533	aerobic respiration control sensor protein ArcB	UP	-	-	-	-	-
YPK_3991	glpA sn-glycerol-3-phosphate dehydrogenase subunit A	UP	-	UP	-	-	-
YPK_2026	hypothetical protein	UP	-	-	-	-	-
YPK_0013	hypothetical protein	UP	-	DOWN	-	-	-
YPK_4119	LysR family transcriptional regulator	UP	-	-	-	-	-
YPK_0562	rRNA (guanine-N(2)-)-methyltransferase	UP	-	-	-	-	-
YPK_1012	general secretion pathway protein L	UP	-	-	-	-	-
YPK_3647	lipopolysaccharide heptosyltransferase III	UP	-	-	-	-	-
YPK_1290	ribosomal RNA large subunit methyltransferase N	UP	-	UP	-	-	-
YPK_1070	1-deoxy-D-xylulose 5-phosphate reductoisomerase	UP	-	UP	-	UP	UP
YPK_2762	PTS system fructose-specific transporter subunitIIBC	UP	-	UP	-	-	-
YPK_1589	2-succinyl-5-enolpyruvyl-6-hydroxy-3-cyclohexene-1-carboxyPersiste	UP	UP	-	-	-	-
YPK_1423	RND family efflux transporter MFP subunit	UP	UP	-	-	-	-
YPK_2792	bicyclomycin/multidrug efflux system protein	UP	-	-	-	-	-
YPK_0162	malQ 4-alpha-glucanotransferase	UP	UP	UP	-	UP	-
YPK_1346	phosphate ABC transporter permease	UP	-	-	-	-	-
YPK_3068	transposase mutator type	UP	-	-	-	-	-
YPK_0096	pectate lyase	UP	-	-	-	-	-
YPK_3550	type VI secretion protein IcmF	UP	-	-	-	UP	UP
YPK_0922	ssDNA exonuclease RecJ	UP	-	UP	-	-	-
YPK_1413	hypothetical protein	UP	-	-	DOWN	DOWN	DOWN
YPK_1712	peptidase T	UP	UP	UP	-	-	-
YPK_3081	alpha/beta hydrolase fold protein	UP	-	-	-	-	-
YPK_0072	hypothetical protein	UP	-	-	-	-	-
YPK_3222	putative multidrug transportermembrane ATP-binding components	UP	-	-	-	-	-
YPK_1709	outer membrane-specific lipoprotein transportersubunit LolE	UP	-	-	-	-	-
YPK_1768	patatin	UP	UP	-	-	-	-
YPK_3514	ftsA cell division protein FtsA	UP	-	UP	-	-	-
YPK_3287	proA gamma-glutamyl phosphate reductase	UP	-	UP	-	-	-
YPK_0820	adenine DNA glycosylase	UP	-	-	-	-	-
YPK_3791	exoribonuclease R	UP	-	UP	-	-	-
YPK_3998	ATP-dependent DNA helicase RecQ	UP	-	-	-	-	-

YPK_0112	gluconate transporter	UP	-	-	-	-	-
YPK_0577	putative adhesin	UP	-	-	-	-	-
YPK_3532	AMP-dependent synthetase and ligase	UP	-	-	-	-	-
YPK_3630	patatin	UP	-	-	-	UP	-
YPK_1979	putative voltage-gated ClC-type chloride channelClcB	UP	-	-	-	UP	-
YPK_0098	C4-dicarboxyPersistency transporter DctA	UP	UP	DOWN	-	UP	-
YPK_1555	citrate transporter	UP	-	UP	-	DOWN	-
YPK_0553	d-galactonate transporter	UP	-	-	-	-	-
YPK_4057	dihydroxy-acid dehydratase	UP	-	-	UP	-	-
YPK_0400	ImpA domain-containing protein	UP	-	-	-	-	UP
YPK_2015	major facilitator transporter	UP	-	-	-	-	-
YPK_3253	1-deoxy-D-xylulose-5-phosphate synthase	UP	-	-	-	-	UP
YPK_4091	phosphoenolpyruvate carboxylase	UP	-	UP	-	-	-
YPK_2526	hisD histidinol dehydrogenase	UP	-	UP	-	UP	-
YPK_4217	tRNA uridine 5-carboxymethylaminomethylmodification protein GidA	UP	-	-	-	-	-
YPK_1332	multidrug efflux system subunit MdtA	UP	-	-	-	-	-
YPK_1259	two component, sigma54 specific, Fis familytranscriptional regulator	UP	-	-	-	-	-
YPK_3377	major facilitator transporter	UP	-	-	-	-	-
YPK_3359	anaerobic C4-dicarboxyPersistency transporter	UP	UP	UP	-	-	-
YPK_0464	regulatory protein CsrD	UP	-	-	-	-	-
YPK_2396	flagellar hook-length control protein	UP	UP	UP	-	DOWN	DOWN
YPK_3305	Na(+)-translocating NADH-quinone reductasesubunit A	UP	-	UP	-	-	-
YPK_2016	exoribonuclease II	UP	-	UP	-	-	-
YPK_2100	putative serine protein kinase PrkA	UP	UP	DOWN	-	UP	UP
YPK_4045	hypothetical protein	UP	-	-	-	-	-
YPK_1718	adenylosuccinate lyase	UP	-	UP	UP	-	-
YPK_3048	thiamine pyrophosphate binding domain-containingprotein	UP	-	-	-	-	-
YPK_4133	cpxA two-component sensor protein	UP	-	UP	-	-	-
YPK_1084	tilS tRNA(Ile)-lysidine synthetase	UP	-	-	-	-	-
YPK_0004	gyrB DNA gyrase subunit B	UP	-	UP	UP	UP	UP
YPK_3198	DNA polymerase III subunits gamma and tau	UP	-	-	-	-	-
YPK_1898	hypothetical protein	UP	-	-	-	-	-
YPK_4173	putative transcriptional regulator	UP	-	-	-	-	-
YPK_3353	sigma 54 modulation protein/30S ribosomalprotein S30EA	UP	UP	DOWN	-	-	-
YPK_4191	glnG nitrogen regulation protein NR(I)	UP	-	-	-	-	-
YPK_2993	outer membrane porin	UP	-	-	-	-	-
YPK_2240	hmsF outer membrane N-deacetylase	UP	-	-	-	-	-
YPK_2805	glycoside hydrolase family protein	UP	-	-	-	UP	-
YPK_3458	chloride channel protein	UP	-	-	-	-	-
YPK_3165	copA copper exporting ATPase	UP	-	DOWN	-	-	-
YPK_0600	Na+/H+ antiporter NhaC	UP	-	DOWN	-	-	-
YPK_1519	mnmC 5-methylaminomethyl-2-thiouridinemethyltransferase	UP	-	-	-	-	-
YPK_4081	dihydrolipoamide dehydrogenase	UP	-	-	-	-	-
YPK_4180	ATP-dependent DNA helicase RecG	UP	-	-	-	-	-
YPK_0469	ribonuclease G	UP	-	-	-	-	-
YPK_1677	rne ribonuclease E	UP	-	UP	-	-	-
YPK_0556	altronate dehydratase	UP	-	-	-	-	-
YPK_2001	L-arabinose isomerase	UP	-	-	-	-	-
YPK_3946	hypothetical protein	UP	-	-	-	-	-
YPK_0178	intracellular growth attenuator IgaA	UP	-	-	-	-	-
YPK_3453	dgt deoxyguanosinetriphosphate triphosphohydrolase	UP	-	-	-	UP	UP
YPK_3936	3-octaprenyl-4-hydroxybenzoate carboxy-lyase	UP	-	UP	-	-	-
YPK_3343	N-acetyltransferase GCN5	UP	-	-	-	-	-
YPK_2835	putative transport protein	UP	UP	-	-	-	-
YPK_3009	lnt apolipoprotein N-acyltransferase	UP	-	-	-	UP	-
YPK_0748	AbgT transporter	UP	-	-	-	DOWN	-
YPK_1181	L-aspartate oxidase	UP	-	-	-	-	-
YPK_0711	fliF flagellar MS-ring protein	UP	-	-	-	-	-
YPK_1152	phosphoglucomutase	UP	-	UP	-	-	-
YPK_1892	extracellular solute-binding protein	UP	-	-	-	-	-
YPK_1835	arnT 4-amino-4-deoxy-L-arabinose transferase	UP	-	-	-	-	-
YPK_1753	methyl-accepting chemotaxis sensory transducer	UP	UP	UP	-	DOWN	DOWN
YPK_0170	RNA-binding S1 domain-containing protein	UP	-	UP	-	-	-
YPK_3394	anaerobic dimethyl sulfoxide reductase subunitA	UP	-	UP	-	-	-
YPK_0802	OmpA/MotB domain-containing protein	UP	-	-	-	-	-
YPK_3770	surface antigen (D15)	UP	-	-	-	-	-
YPK_1742	methyl-accepting chemotaxis sensory transducer	UP	UP	-	-	-	-
YPK_2051	sulfate transporter	UP	UP	-	-	-	-
YPK_1845	FAD linked oxidase domain-containing protein	UP	-	-	-	-	-
YPK_3271	note_PFAM:_alpha_amylase_catalytic_region,SMART:_alpha_amylas	UP	-	UP	-	-	-
YPK_3311	putative accessory processing protein	UP	-	-	-	-	-
YPK_2654	hypothetical protein	UP	UP	-	-	-	-
YPK_4211	trkD potassium transport protein Kup	UP	-	-	-	-	-
YPK_0659	DNA topoisomerase IV subunit B	UP	-	UP	-	-	-
YPK_1744	magnesium-translocating P-type ATPase	UP	UP	DOWN	DOWN	-	-
YPK_2640	ABC transporter ATPase	UP	-	UP	-	-	-
YPK_3802	N-acetylmuramoyl-L-alanine amidase	UP	-	-	-	-	-
YPK_1823	rplT 50S ribosomal protein L20	UP	UP	UP	-	-	-
YPK_2645	pepN aminopeptidase N	UP	-	UP	-	-	-
YPK_3831	putative oxidoreductase Fe-S binding subunit	UP	-	-	-	-	-
YPK_1422	ABC transporter-like protein	UP	-	-	-	-	-
YPK_3144	SMC (structural maintenance of chromosomes)family protein	UP	-	-	-	UP	-
YPK_1368	hypothetical protein	UP	-	-	-	-	-
YPK_2442	oligopeptidase B	UP	-	UP	-	UP	UP
YPK_2630	outer membrane protein A	UP	UP	UP	UP	UP	UP

YPK_2017	carbon starvation protein CstA	UP	-	DOWN	-	UP	-
YPK_2493	glycoside hydrolase clan GH-D	UP	-	-	-	-	-
YPK_2002	type I secretion system ATPase	UP	-	-	-	-	-
YPK_2627	hypothetical protein	UP	-	-	-	-	-
YPK_1027	bifunctional acyl-[acyl carrier protein]synthetase/2-acylglycerophosph	UP	-	-	-	-	-
YPK_1345	binding-protein-dependent transport system innermembrane protein	UP	-	-	-	-	-
YPK_2207	Sel1 domain-containing protein	UP	-	-	-	-	-
YPK_1212	virulence-associated E family protein	UP	-	-	-	-	-
YPK_2856	dinG ATP-dependent DNA helicase DinG	UP	-	-	-	-	-
YPK_0094	biofilm formation regulator HmsP	UP	-	-	-	-	-
YPK_3426	nlpD lipoprotein NlpD	UP	-	UP	-	-	-
YPK_2667	ihfB integration host factor subunit beta	UP	UP	UP	-	UP	UP
YPK_3769	hypothetical protein	UP	-	-	-	-	-
YPK_0470	hypothetical protein	UP	-	-	-	-	-
YPK_0662	DNA topoisomerase IV subunit A	UP	-	UP	-	-	-
YPK_0583	outer membrane autotransporter	UP	-	-	-	-	-
YPK_2098	MltA-interacting MipA family protein	UP	-	-	-	-	-
YPK_1041	recC exonuclease V subunit gamma	UP	-	-	-	-	-
YPK_3072	glycoside hydrolase family 3	UP	-	-	-	UP	UP
YPK_1288	beta and gamma crystallin	UP	-	-	-	-	-
YPK_1456	anaerobic dimethyl sulfoxide reductase subunitA	UP	-	UP	-	-	-
YPK_0370		UP	-	-	-	-	-
YPK_2451	L-serine dehydratase 1	UP	-	-	-	UP	-
YPK_0052	fimbrial biogenesis outer membrane usherprotein	UP	-	-	-	-	-
YPK_0219	mrcA peptidoglycan synthetase	UP	-	-	-	-	-
YPK_2274	fimbrial biogenesis outer membrane usherprotein	UP	-	-	-	-	-
YPK_0341	DNA-directed RNA polymerase subunit beta	UP	-	UP	-	UP	UP
YPK_0804	type VI secretion ATPase	UP	-	-	-	-	-
YPK_2074	global DNA-binding transcriptional dualregulator H-NS	UP	-	UP	-	-	-
YPK_0354	transcriptional regulator HU subunit alpha	UP	UP	UP	-	-	-
YPK_4118	tpiA triosephosphate isomerase	UP	-	UP	-	-	-
YPK_4225	F0F1 ATP synthase subunit gamma	UP	-	UP	UP	UP	DOWN
YPK_0295	rplE 50S ribosomal protein L5	UP	-	UP	-	-	-
YPK_2677	formate acetyltransferase	UP	-	UP	-	UP	-
YPK_3547	ATP-dependent helicase HepA	UP	-	-	-	-	-
YPK_3267	alkyl hydroperoxide reductase	UP	UP	UP	-	DOWN	-
YPK_3152	cysS cysteinyl-tRNA synthetase	UP	-	UP	-	-	-
YPK_1001	carbonic anhydrase	UP	UP	DOWN	-	UP	-
YPK_3642	acriflavin resistance protein	UP	UP	-	-	DOWN	-
YPK_2955	peptidoglycan-associated outer membranelipoprotein	UP	UP	UP	-	UP	UP
YPK_2566	periplasmic binding protein/LacI transcriptionalregulator	UP	-	-	-	UP	DOWN
YPK_0505	RNA polymerase factor sigma-54	UP	UP	-	-	-	-
YPK_3446	eno phosphopyruvate hydratase	UP	UP	UP	-	-	-
YPK_3825	aspA aspartate ammonia-lyase	UP	-	UP	-	UP	-
YPK_0107	outer membrane autotransporter	UP	-	-	-	-	-
YPK_3147	hypothetical protein	UP	-	-	-	DOWN	DOWN
YPK_0547	hypothetical protein	UP	UP	-	-	-	-
YPK_3242	cytochrome o ubiquinol oxidase subunit I	UP	-	UP	-	-	-
YPK_3726	polynucleotide phosphorylase/polyadenylase	UP	-	UP	-	-	-
YPK_1854	LPP repeat-containing protein	UP	UP	-	DOWN	DOWN	DOWN
YPK_0332	elongation factor Tu	UP	UP	UP	-	-	-
YPK_0634	RNA polymerase sigma factor RpoD	UP	-	UP	-	-	-
YPK_0289	rpsC 30S ribosomal protein S3	UP	-	UP	UP	UP	-
YPK_0296	rpsN 30S ribosomal protein S14	UP	-	UP	UP	UP	-
YPK_0340	rpoB DNA-directed RNA polymerase subunit beta	UP	-	UP	-	-	-
YPK_1485	type VI secretion protein IcmF	UP	-	-	-	-	-
YPK_1253	phosphoribosylformylglycinamidine synthase	UP	-	UP	-	-	-
YPK_1821	translation initiation factor IF-3	UP	UP	-	-	-	-
YPK_0869	glycine dehydrogenase	UP	-	UP	-	-	-
pYV0006	hypothetical protein	UP	UP	-	-	-	-
pYV0026	hypothetical protein	UP	-	UP	-	DOWN	-
pYV0031	plasmid-partitioning protein	UP	-	-	-	-	-
pYV0045	hypothetical protein	UP	-	-	-	-	-
YPK_2997	nagB glucosamine-6-phosphate deaminase	UP	-	UP	-	-	-
YPK_4059	branched-chain amino acid aminotransferase	DOWN	-	UP	-	-	-
pYV0072	yscS, type III secretion protein	DOWN	DOWN	-	-	-	-
YPK_1360	bcp thioredoxin-dependent thiol peroxidase	DOWN	-	UP	-	DOWN	-
YPK_0160	transcriptional regulator MalT	DOWN	-	UP	-	-	DOWN
YPK_1073	zinc metallopeptidase RseP	DOWN	-	UP	-	UP	-
YPK_4005	hypothetical protein	DOWN	-	-	-	-	-
YPK_4079	DNA-binding transcriptional regulator OxyR	DOWN	-	UP	-	-	-
YPK_3424	DNA mismatch repair protein MutS	DOWN	-	-	-	-	-
YPK_3921	acetyl-CoA synthetase	DOWN	-	DOWN	-	UP	-
YPK_0853	fructose-bisphosphate aldolase	DOWN	-	UP	-	-	-
pYV0061	yscY, type III secretion protein	DOWN	DOWN	UP	-	-	-
YPK_2135	DNA-binding transcriptional regulator HexR	DOWN	-	UP	-	-	-
YPK_3298	hypothetical protein	DOWN	UP	-	-	-	-
YPK_3881	hypothetical protein	DOWN	-	-	-	-	-
YPK_1782	putative solute/DNA competence effector	DOWN	-	UP	-	-	-
YPK_1192	pyridoxine 5'-phosphate synthase	DOWN	-	-	-	-	-
YPK_1034	fused phosphoenolpyruvate-proteinphosphotransferase PtsP/GAF dc	DOWN	-	UP	-	-	-
YPK_1925	ATP-dependent RNA helicase HrpA	DOWN	-	-	-	-	-
YPK_0849	peptidase M48 Ste24p	DOWN	-	UP	-	DOWN	DOWN
YPK_1150	hypothetical protein	DOWN	UP	-	-	-	DOWN
YPK_1910	hypothetical protein	DOWN	-	-	-	-	-

YPK_1995	putative oxidoreductase	DOWN	-	-	-	-	UP
YPK_2537	UTP-glucose-1-phosphate uridylyltransferase	DOWN	-	-	-	UP	UP
YPK_2998	nagA N-acetylglucosamine-6-phosphate deacetylase	DOWN	-	UP	-	-	-
YPK_3104	hypothetical protein	DOWN	-	-	-	-	-
YPK_1076	lpxD UDP-3-O-[3-hydroxymyristoyl] glucosamineN-acyltransferase	DOWN	-	UP	-	UP	UP
YPK_4126	6-phosphofructokinase	DOWN	-	UP	-	-	-
YPK_0043	selenocysteinyl-tRNA-specific translationfactor	DOWN	-	-	-	-	-
YPK_0271	YheO domain-containing protein	DOWN	UP	-	-	-	-
YPK_3479	hypoxanthine phosphoribosyltransferase	DOWN	-	UP	-	-	-
YPK_3584	dihydrodipicolinate reductase	DOWN	-	UP	-	DOWN	-
YPK_2139	hypothetical protein	DOWN	UP	-	-	-	-
YPK_2650	aromatic amino acid aminotransferase	DOWN	-	UP	-	-	-
YPK_0654	tolC outer membrane channel protein	DOWN	-	UP	-	UP	UP
YPK_1548	putative PTS IIA-like nitrogen-regulatoryprotein PtsN	DOWN	-	UP	-	DOWN	DOWN
YPK_1610	alcohol dehydrogenase	DOWN	-	DOWN	-	-	-
YPK_3766	fructose-1	DOWN	-	-	-	-	-
YPK_4044	fimbrial biogenesis outer membrane usherprotein	DOWN	-	-	-	-	-
YPK_0039	formate dehydrogenase subunit beta	DOWN	-	-	-	-	-
YPK_0151	glycogen/starch/alpha-glucan phosphorylase	DOWN	-	DOWN	UP	UP	UP
YPK_1297	engA GTP-binding protein EngA	DOWN	-	UP	-	-	-
YPK_3810	psd phosphatidylserine decarboxylase	DOWN	-	UP	-	-	-
YPK_0101	ribokinase-like domain-containing protein	DOWN	-	UP	-	-	-
YPK_2150	hypothetical protein	DOWN	-	DOWN	-	-	-
YPK_2685	cell division protein FtsK	DOWN	-	UP	-	UP	-
YPK_2096	glyceraldehyde-3-phosphate dehydrogenase	DOWN	-	UP	-	-	-
YPK_0825	virulence determinant	DOWN	DOWN	-	-	-	-
YPK_0335	rplK 50S ribosomal protein L11	DOWN	-	UP	-	-	DOWN
YPK_1903	tpx thiol peroxidase	DOWN	DOWN	UP	-	-	UP
YPK_2967	dihydrolipoamide succinyltransferase	DOWN	-	UP	UP	UP	-
YPK_3739	greA transcription elongation factor GreA	DOWN	UP	UP	-	DOWN	DOWN
YPK_1066	rpsB 30S ribosomal protein S2	DOWN	DOWN	UP	-	-	-
YPK_3873	stress protein	DOWN	-	UP	-	-	-
YPK_4104	hslU ATP-dependent protease ATP-binding subunit HslU	DOWN	-	UP	-	-	-
YPK_3862	glycerol-3-phosphate acyltransferase	DOWN	-	UP	-	-	-
YPK_1561	NADH dehydrogenase subunit B	DOWN	-	UP	-	-	-
YPK_1881	tyrosyl-tRNA synthetase	DOWN	-	UP	-	-	-
YPK_1363	lipoprotein	DOWN	-	UP	-	-	-
YPK_0558	serine/threonine transporter SstT	DOWN	UP	DOWN	-	-	-
YPK_2966	sucC succinyl-CoA synthetase subunit beta	DOWN	-	UP	UP	UP	-
YPK_2996	PTS system N-acetylglucosamine-specifictransporter subunit IIBC	DOWN	-	UP	-	-	-
YPK_2973	gltA type II citrate synthase	DOWN	-	UP	UP	UP	-
YPK_2118	hypothetical protein	DOWN	-	-	-	-	-
YPK_1783	carboxy-terminal protease	DOWN	-	UP	UP	UP	UP
pYV0005	replication protein	DOWN	-	-	-	-	-
YPK_1042	peptidase M16 domain-containing protein	DOWN	-	-	-	UP	UP
YPK_0564	acid-resistance membrane protein	DOWN	-	-	-	-	-
YPK_0478	virulence plasmid 65kDa B protein	DOWN	-	-	-	-	-
YPK_1184	rseB periplasmic negative regulator of sigmaE	DOWN	DOWN	-	-	-	UP
YPK_3599	transaldolase B	DOWN	-	UP	-	-	-
YPK_2655	mukB cell division protein MukB	DOWN	-	UP	-	UP	-
YPK_1043	recB exonuclease V subunit beta	DOWN	-	-	-	UP	UP
YPK_3582	carB carbamoyl phosphate synthase large subunit	DOWN	-	UP	UP	-	-
YPK_1739	lacZ beta-D-galactosidase	DOWN	-	-	-	-	-
YPK_1268	virulence-related outer membrane protein	DOWN	DOWN	-	UP	-	UP
YPK_2350	TP901 family phage tail tape measure protein	DOWN	-	-	-	-	-
YPK_2270		DOWN	-	-	-	-	-
YPK_2030	aconitate hydratase	DOWN	-	DOWN	UP	UP	UP
pYV0067	type III secretion system ATPase	DOWN	DOWN	-	-	DOWN	-
YPK_0124	multi-sensor hybrid histidine kinase	DOWN	-	-	-	-	-
YPK_2574	hypothetical protein	DOWN	-	-	-	-	-
YPK_2839	outer membrane porin protein C	DOWN	DOWN	UP	-	UP	-
YPK_0190	hypothetical protein	DOWN	-	-	-	-	-
YPK_1117	ribonucleotide-diphosphate reductase subunitalpha	DOWN	-	-	-	-	-
YPK_2033	DNA topoisomerase I	DOWN	-	UP	-	-	-
YPK_0702	flhA flagellar biosynthesis protein FlhA	DOWN	-	-	-	-	-
YPK_0023	glyS glycyl-tRNA synthetase subunit beta	DOWN	-	UP	UP	-	-
YPK_3711	heparinase II/III family protein	DOWN	-	-	-	-	-
YPK_0473	YD repeat-containing protein	DOWN	-	-	-	-	-
YPK_0644	bifunctional glutamine-synthetaseadenylyltransferase/deadenyltransf	DOWN	-	-	-	-	-
YPK_1044	recD exonuclease V subunit alpha	DOWN	-	-	-	-	-
YPK_2615	cytotoxic necrotizing factor	DOWN	DOWN	UP	-	-	-
YPK_1280	hscA chaperone protein HscA	DOWN	-	UP	-	-	-
YPK_0595	fusaric acid resistance protein region	DOWN	-	-	-	-	-
YPK_3666	Type I site-specific deoxyribonuclease	DOWN	-	-	-	-	-
YPK_1376	binding-protein-dependent transport system innermembrane protein	DOWN	-	DOWN	UP	UP	-
YPK_1091	prolyl-tRNA synthetase	DOWN	-	UP	-	-	-
YPK_3175	putative cation:proton antiport protein	DOWN	-	-	-	-	-
YPK_1843	phosphoenolpyruvate synthase	DOWN	-	DOWN	-	UP	UP
YPK_3204	potassium efflux protein KefA	DOWN	-	-	-	-	-
YPK_1950	acetolactate synthase catalytic subunit	DOWN	-	-	-	UP	-
YPK_2699	hypothetical protein	DOWN	-	-	-	-	-
YPK_3232	DNA-binding ATP-dependent protease La	DOWN	-	UP	UP	UP	-
YPK_1947	hypothetical protein	DOWN	-	-	-	-	-
YPK_1421	FAD-dependent pyridine nucleotide-disulfideoxidoreductase	DOWN	-	-	-	-	-
YPK_3447	pyrG CTP synthetase	DOWN	-	UP	-	-	-

YPK_1222	hypothetical protein	DOWN	-	-	-	-	-
YPK_3633	prfC peptide chain release factor 3	DOWN	DOWN	UP	-	-	-
YPK_3650	ABC transporter-like protein	DOWN	-	DOWN	-	-	-
YPK_0377	malF maltose transporter membrane protein	DOWN	UP	UP	-	-	-
YPK_1407	putative sialic acid transporter	DOWN	DOWN	-	-	-	-
YPK_1571	NADH dehydrogenase subunit M	DOWN	-	UP	-	-	-
YPK_3385	ABC transporter-like protein	DOWN	-	-	-	-	-
YPK_0966	ABC transporter-like protein	DOWN	DOWN	-	-	-	-
YPK_2008	electron transport complex protein RnfC	DOWN	-	-	-	-	-
YPK_1933		DOWN	-	-	-	-	-
YPK_1420	Na+/solute symporter	DOWN	-	-	-	-	-
YPK_1344	polyphosphate kinase	DOWN	-	-	-	-	-
pYV0024	sycE, yerA, yopE chaperone	DOWN	DOWN	-	-	DOWN	DOWN
YPK_0150	glgA glycogen synthase	DOWN	-	-	-	UP	-
YPK_0614	integrase family protein	DOWN	-	-	-	-	-
YPK_2361	D-alanine/D-serine/glycine permease	DOWN	DOWN	-	-	-	-
YPK_4207	major facilitator transporter	DOWN	UP	-	-	-	-
YPK_0495	PTS system trehalose(maltose)-specifictransporter subunit IIBC	DOWN	DOWN	-	-	-	UP
YPK_2748	putative outer membrane receptor	DOWN	-	-	-	-	-
YPK_3717	RND efflux system outer membrane lipoprotein	DOWN	-	-	-	-	-
YPK_2446	ATP-dependent RNA helicase DbpA	DOWN	-	UP	-	-	-
YPK_4228	glmU bifunctional N-acetylglucosamine-1-phosphateuridyltransferas	DOWN	-	UP	-	-	UP
YPK_4023	putative common antigen polymerase	DOWN	-	-	-	-	-
YPK_0264	putative ABC transporter ATP-binding protein	DOWN	-	-	-	-	-
YPK_3145	hypothetical protein	DOWN	-	-	-	-	-
YPK_0863	proline aminopeptidase P II	DOWN	-	UP	-	-	-
YPK_3570	organic solvent tolerance protein	DOWN	-	UP	-	-	-
YPK_3487	bifunctional aconitate hydratase2/2-methylisocitrate dehydratase	DOWN	-	UP	UP	UP	UP
YPK_3219	ammonium transporter	DOWN	-	-	-	-	-
YPK_1353	uracil transporter	DOWN	-	-	-	-	-
YPK_2636	3-oxoacyl-(acyl carrier protein) synthase II	DOWN	-	-	-	UP	UP
YPK_0685	type II and III secretion system protein	DOWN	-	-	-	-	-
YPK_4162	integrase family protein	DOWN	-	-	-	-	-
YPK_0859	D-3-phosphoglycerate dehydrogenase	DOWN	-	UP	-	DOWN	-
YPK_0427	transposase mutator type	DOWN	-	-	-	UP	-
YPK_1707	outer membrane-specific lipoprotein transportersubunit LolC	DOWN	-	UP	-	-	-
YPK_1565	NADH dehydrogenase subunit G	DOWN	-	UP	-	-	-
YPK_2620	PUA domain-containing protein	DOWN	-	UP	-	-	-
YPK_3876	stress protein	DOWN	-	UP	UP	UP	-
YPK_0440	hypothetical protein	DOWN	-	-	-	-	-
YPK_2751	periplasmic binding protein	DOWN	-	-	-	-	DOWN
YPK_3705	hypothetical protein	DOWN	-	-	-	-	-
YPK_0852	pgk phosphoglycerate kinase	DOWN	-	UP	-	-	-
YPK_0651	glutathionylspermidine synthase	DOWN	-	UP	-	-	-
YPK_3924	hypothetical protein	DOWN	-	-	-	-	-
YPK_3177	ferric enterobactin transport protein FepE	DOWN	UP	-	-	-	-
YPK_0519	serine endoprotease	DOWN	-	-	-	-	-
YPK_4031	UDP-N-acetylglucosamine 2-epimerase	DOWN	-	UP	-	UP	-
YPK_2549	integral membrane protein TerC	DOWN	-	-	-	-	-
YPK_3421	alcohol dehydrogenase	DOWN	-	-	-	-	-
YPK_1878	anmK anhydro-N-acetylmuramic acid kinase	DOWN	-	-	-	UP	UP
YPK_1292	cytoskeletal protein RodZ	DOWN	-	UP	-	-	-
YPK_0967	ABC transporter-like protein	DOWN	DOWN	UP	-	-	-
YPK_2547	pyridoxal-dependent decarboxylase	DOWN	DOWN	-	-	-	-
YPK_0226	aroB 3-dehydroquinate synthase	DOWN	-	UP	-	-	-
YPK_2108	SpoVR family protein	DOWN	UP	DOWN	-	UP	UP
YPK_2179	prfA peptide chain release factor 1	DOWN	-	UP	-	DOWN	-
YPK_2196	hypothetical protein	DOWN	-	-	-	-	-
YPK_1974	aldehyde dehydrogenase	DOWN	-	DOWN	-	UP	-
YPK_0152	glpD glycerol-3-phosphate dehydrogenase	DOWN	-	DOWN	-	-	DOWN
YPK_2528	imidazole glycerol-phosphatedehydratase/histidinol phosphatase	DOWN	-	-	UP	UP	-
YPK_1899	hypothetical protein	DOWN	-	-	-	-	UP
YPK_3403	carbohydrate kinase FGGY	DOWN	-	DOWN	-	-	-
YPK_4167	hypothetical protein	DOWN	UP	-	-	-	-
YPK_4067	ABC transporter-like protein	DOWN	-	-	-	-	-
YPK_3501	guanosine 5'-monophosphate oxidoreductase	DOWN	-	UP	-	DOWN	DOWN
YPK_2542	transport system permease	DOWN	-	-	-	-	-
YPK_2674	ansB L-asparaginase II	DOWN	-	-	-	-	-
YPK_1572	NADH dehydrogenase subunit N	DOWN	-	UP	-	-	-
YPK_3938	delta-aminolevulinic acid dehydratase	DOWN	-	UP	-	-	-
YPK_4137	gpsA NAD(P)H-dependent glycerol-3-phosphatedehydrogenase	DOWN	-	UP	-	-	-
YPK_2092	ansA asparaginase	DOWN	-	-	-	-	-
YPK_2943	UDP-galactose-4-epimerase	DOWN	-	-	-	-	-
YPK_4114	glpX fructose 1,6-bisphosphatase II	DOWN	-	UP	-	-	-
YPK_4122	anion transporter	DOWN	-	-	-	-	-
YPK_2891	helicase domain-containing protein	DOWN	-	UP	-	-	-
YPK_4089	argC N-acetyl-gamma-glutamyl-phosphate reductase	DOWN	-	-	-	DOWN	-
YPK_0964	periplasmic binding protein/LacI transcriptionalregulator	DOWN	DOWN	DOWN	-	-	-
YPK_1985	fumC fumarate hydratase	DOWN	-	DOWN	-	-	-
YPK_1116	nrdF ribonucleotide-diphosphate reductase subunitbeta	DOWN	-	-	-	-	-
YPK_1592	O-succinylbenzoate synthase	DOWN	-	UP	-	-	-
YPK_2152	methyltransferase	DOWN	-	-	-	-	-
YPK_3193	hemH ferrochelatase	DOWN	-	UP	-	-	-
YPK_3552	type VI secretion protein	DOWN	-	-	UP	UP	UP
YPK_0496	treR trehalose repressor	DOWN	-	-	-	-	-

YPK_0315	fmt methionyl-tRNA formyltransferase	DOWN	-	UP	-	-	-
YPK_1179	ATP-dependent RNA helicase SrmB	DOWN	-	UP	-	-	-
YPK_2865	glycine betaine ABC transportersubstrate-binding protein	DOWN	-	-	-	UP	UP
YPK_3626	deoA thymidine phosphorylase	DOWN	-	DOWN	UP	-	UP
YPK_1611	monosaccharide-transporting ATPase	DOWN	-	DOWN	-	-	-
YPK_0725	putative transcriptional regulator CadC	DOWN	-	-	-	-	UP
YPK_3797	putative GTPase HflX	DOWN	-	UP	-	-	-
YPK_2639	PqiA family integral membrane protein	DOWN	-	-	-	UP	-
YPK_3262	secD preprotein translocase subunit SecD	DOWN	-	UP	-	-	-
YPK_0376	malG maltose transporter permease	DOWN	-	-	-	-	-
YPK_2436	copper resistance D domain-containing protein	DOWN	-	-	-	-	-
YPK_4186	rbn ribonuclease BN	DOWN	-	-	-	-	-
YPK_3402	putative L-xylulose 5-phosphate 3-epimerase	DOWN	-	DOWN	-	-	-
YPK_1445	hypothetical protein	DOWN	-	DOWN	-	-	-
YPK_3290	frsA fermentation/respiration switch protein	DOWN	-	UP	-	-	-
YPK_3625	phosphopentomutase	DOWN	-	DOWN	UP	-	UP
YPK_1727	NmrA family protein	DOWN	-	DOWN	-	UP	UP
YPK_3523	murE UDP-N-acetylmuramoylalanyl-D-glutamate--2,6-diaminopimel	DOWN	-	UP	-	-	-
YPK_3495	regulatory protein AmpE	DOWN	UP	-	-	-	-
YPK_2488	hypothetical protein	DOWN	-	-	-	-	UP
YPK_2080	purU formyltetrahydrofolate deformylase	DOWN	-	UP	-	DOWN	-
YPK_3891	periplasmic binding protein	DOWN	-	-	-	UP	-
YPK_1968	class I and II aminotransferase	DOWN	UP	-	-	-	-
YPK_3583	carbamoyl phosphate synthase small subunit	DOWN	-	UP	-	-	-
YPK_2902	ABC-2 type transporter	DOWN	-	-	-	-	-
YPK_3923	actP acetate permease	DOWN	DOWN	DOWN	-	UP	-
YPK_4189	glnA glutamine synthetase	DOWN	-	UP	-	-	-
YPK_3900	type III secretion protein SpaR/YscT/HrcT	DOWN	-	-	-	-	-
YPK_0113	transcriptional regulator CdaR	DOWN	-	-	-	-	-
pYV0065	yopN, lcrE, membrane-bound Yop targetingprotein	DOWN	DOWN	UP	UP	UP	UP
YPK_1078	UDP-N-acetylglucosamine acyltransferase	DOWN	-	UP	-	-	-
YPK_2235	hypothetical protein	DOWN	-	DOWN	-	UP	UP
YPK_3974	extracellular ligand-binding receptor	DOWN	-	-	-	-	UP
YPK_1526	AraC family transcriptional regulator	DOWN	-	-	-	-	-
YPK_0513	hypothetical protein	DOWN	-	-	-	-	UP
YPK_2548	ferric hydroximate transport ferric ironreductase	DOWN	-	-	-	DOWN	DOWN
YPK_3344	pssA phosphatidylserine synthase	DOWN	-	UP	-	-	-
YPK_2787	binding-protein-dependent transport system innermembrane protein	DOWN	DOWN	-	-	-	-
YPK_3307	glutamine amidotransferase	DOWN	-	-	-	-	-
YPK_2010	rnfD electron transport complex protein RnfD	DOWN	-	-	-	-	-
YPK_2750	transport system permease	DOWN	-	-	-	-	DOWN
YPK_3006	PhoH family protein	DOWN	-	UP	-	-	-
YPK_0351	hemE uroporphyrinogen decarboxylase	DOWN	-	-	-	-	-
YPK_2168	ABC transporter-like protein	DOWN	-	-	-	-	-
YPK_1357	hypothetical protein	DOWN	-	-	-	-	-
YPK_3180	glycosyl transferase family protein	DOWN	-	UP	-	-	-
YPK_3687	binding-protein-dependent transport system innermembrane protein	DOWN	-	-	-	-	-
YPK_1533	hypothetical protein	DOWN	-	-	-	UP	UP
YPK_3636	rsmC 16S ribosomal RNA m2G1207 methyltransferase	DOWN	-	UP	-	-	-
YPK_0824	hypothetical protein	DOWN	-	-	-	-	-
YPK_0231	tryptophanyl-tRNA synthetase	DOWN	-	UP	-	-	-
YPK_3352	outer membrane protein assembly complex subunitYfiO	DOWN	-	UP	-	-	-
YPK_4080	glutaredoxin family protein	DOWN	-	-	-	-	-
YPK_0672	tonB-system energizer ExbB	DOWN	-	-	-	-	-
YPK_0326	murB UDP-N-acetylenolpyruvoylglucosamine reductase	DOWN	-	UP	-	-	-
YPK_2656	condesin subunit E	DOWN	-	UP	-	UP	-
YPK_0535	mtgA monofunctional biosynthetic peptidoglycantransglycosylase	DOWN	-	-	-	-	-
YPK_1068	pyrH uridylate kinase	DOWN	-	UP	-	-	-
YPK_2788	binding-protein-dependent transport system innermembrane protein	DOWN	DOWN	-	-	-	-
YPK_2377	D-cysteine desulfhydrase	DOWN	-	UP	-	-	-
YPK_4235	transcriptional regulator PhoU	DOWN	-	-	-	-	-
YPK_2078	response regulator of RpoS	DOWN	-	-	-	-	UP
YPK_1614	LacI family transcriptional regulator	DOWN	-	-	-	-	-
YPK_2066	oligopeptide/dipeptide ABC transporter ATPase	DOWN	-	-	-	-	-
YPK_0568	von Willebrand factor type A	DOWN	-	-	-	-	-
YPK_3995	lysophospholipase L2	DOWN	-	-	-	-	-
YPK_0431	carbohydrate ABC transporter periplasmic-bindingprotein	DOWN	-	-	-	-	DOWN
YPK_1134	ureE urease accessory protein UreE	DOWN	-	UP	-	UP	UP
YPK_2782	cobalamin synthesis protein P47K	DOWN	-	-	-	-	-
YPK_3351	rluD 23S rRNA pseudouridine synthase D	DOWN	-	-	-	-	-
YPK_3158	putative ABC transporter ATP-binding proteinYbbA	DOWN	-	-	-	UP	-
YPK_1984	DNA replication terminus site-binding protein	DOWN	-	-	-	-	-
YPK_3417	DeoR family transcriptional regulator	DOWN	-	UP	-	-	-
YPK_4129	putative transposase YhgA family protein	DOWN	-	-	-	-	-
YPK_1940	hypothetical protein	DOWN	-	UP	-	-	-
YPK_2259	LysR family transcriptional regulator	DOWN	-	-	-	-	-
YPK_0083	regulatory protein UhpC	DOWN	-	-	-	UP	-
YPK_4179	tRNA guanosine-2'-O-methyltransferase	DOWN	-	-	-	-	-
YPK_1167	allophanate hydrolase subunit 1	DOWN	-	UP	-	-	DOWN
YPK_0923	prfB peptide chain release factor 2	DOWN	-	UP	-	-	-
YPK_0355	hypothetical protein	DOWN	-	UP	-	UP	-
YPK_4184	thioesterase domain-containing protein	DOWN	-	-	-	-	-
YPK_2069	oppB oligopeptide transporter permease	DOWN	-	-	-	UP	-
YPK_2093	nicotinamidase/pyrazinamidase	DOWN	-	-	-	-	-
YPK_3737	rrmJ 23S rRNA methyltransferase J	DOWN	-	UP	-	-	-

YPK_2536	UTP-glucose-1-phosphate uridylyltransferase	DOWN	-	-	-	UP	UP
YPK_2858	hypothetical protein	DOWN	UP	-	-	-	-
YPK_4034	rho transcription termination factor Rho	DOWN	DOWN	UP	-	-	-
YPK_0035	superoxide dismutase	DOWN	-	UP	DOWN	DOWN	DOWN
YPK_0931	putative bacteriophage protein	DOWN	-	-	-	-	-
YPK_2970	sdhA succinate dehydrogenase flavoprotein subunit	DOWN	-	UP	-	UP	-
YPK_3555	hypothetical protein	DOWN	-	-	UP	UP	UP
YPK_1366	hypothetical protein	DOWN	-	-	-	-	-
YPK_3161	thioredoxin domain-containing protein	DOWN	-	UP	-	UP	-
pYV0074	yscU, type III secretion protein	DOWN	DOWN	-	-	DOWN	-
YPK_2292	transposase mutator type	DOWN	-	-	-	UP	-
YPK_3392	DMSO reductase anchor subunit DmsC	DOWN	-	UP	-	-	-
pYV0037		DOWN	DOWN	-	-	-	-
YPK_2036	putative periplasmic protease	DOWN	-	-	-	-	-
YPK_1390	NUDIX hydrolase	DOWN	-	-	-	-	-
YPK_0661	NAD(P)H dehydrogenase (quinone)	DOWN	-	-	-	DOWN	DOWN
YPK_1695	shikimate 5-dehydrogenase	DOWN	-	UP	-	-	-
YPK_3196	recR recombination protein RecR	DOWN	-	UP	-	-	-
pYV0076	lcrF, virF, thermoregulatory protein	DOWN	DOWN	-	-	DOWN	-
YPK_2163	hypothetical protein	DOWN	-	-	-	-	-
YPK_1275	DNA-binding transcriptional regulator IscR	DOWN	DOWN	DOWN	-	-	-
YPK_3366	cytochrome c assembly protein	DOWN	-	-	DOWN	DOWN	-
YPK_0991	PTS sorbose-specific transporter subunit IIC	DOWN	-	UP	-	-	-
YPK_3406	methionine aminopeptidase	DOWN	-	DOWN	-	-	-
YPK_4150	glycosyl transferase family protein	DOWN	UP	-	-	-	-
YPK_0489	hypothetical protein	DOWN	-	UP	-	-	-
YPK_1349	N-acetyltransferase GCN5	DOWN	DOWN	UP	-	DOWN	DOWN
YPK_2055	transporter	DOWN	DOWN	-	-	DOWN	DOWN
YPK_2037	short chain dehydrogenase	DOWN	-	UP	-	-	-
YPK_2293	hypothetical protein	DOWN	-	-	-	-	-
YPK_0557	hypothetical protein	DOWN	-	-	-	-	-
YPK_3753	hypothetical protein	DOWN	-	UP	-	DOWN	-
YPK_2847	3-demethylubiquinone-9 3-methyltransferase	DOWN	-	-	-	-	-
YPK_4121	hypothetical protein	DOWN	-	-	-	-	-
YPK_2245	redoxin domain-containing protein	DOWN	-	-	-	-	-
YPK_2793	16S rRNA pseudouridylate synthase A	DOWN	UP	-	-	-	-
YPK_3725	lipoprotein NlpI	DOWN	UP	-	-	-	-
YPK_0177	HAD family hydrolase	DOWN	-	-	-	-	-
YPK_1189	rnc ribonuclease III	DOWN	-	UP	-	-	-
YPK_1511	phosphohistidine phosphatase	DOWN	-	-	-	-	-
YPK_0147	glycogen branching protein	DOWN	-	-	-	UP	-
YPK_3020	rRNA large subunit methyltransferase	DOWN	-	UP	-	-	-
YPK_1405	hypothetical protein	DOWN	-	-	-	DOWN	DOWN
YPK_0459	acetyl-CoA carboxylase biotin carboxyl carrierprotein subunit	DOWN	DOWN	UP	-	-	-
YPK_3223	AsnC family transcriptional regulator	DOWN	-	-	-	-	-
YPK_1685	malonyl CoA-acyl carrier protein transacylase	DOWN	-	UP	-	-	-
YPK_3890	hemin-degrading family protein	DOWN	-	UP	UP	UP	-
YPK_0856	hypothetical protein	DOWN	-	UP	-	UP	UP
YPK_4159	pyrE orotate phosphoribosyltransferase	DOWN	-	UP	-	DOWN	DOWN
YPK_2739	serine transporter	DOWN	-	UP	-	-	DOWN
YPK_1694	PTS system glucose-specific transporter subunitIIBC	DOWN	-	UP	-	DOWN	DOWN
YPK_4076	DNA-binding transcriptional repressor FabR	DOWN	-	-	-	-	-
YPK_0602	YheO domain-containing protein	DOWN	-	-	-	DOWN	DOWN
YPK_1384	two component LuxR family transcriptionalregulator	DOWN	-	-	-	-	-
YPK_1313		DOWN	-	-	-	-	-
YPK_4140	rhodanese domain-containing protein	DOWN	-	UP	-	DOWN	DOWN
YPK_0730	MotA/TolQ/ExbB proton channel	DOWN	-	-	-	-	-
YPK_3877	stress protein	DOWN	-	UP	-	UP	-
pYV0025	outer membrane virulence protein	DOWN	-	UP	-	DOWN	-
YPK_0658	esterase YqiA	DOWN	-	UP	-	-	-
YPK_0242	peptidyl-prolyl cis-trans isomerase A	DOWN	-	UP	-	-	DOWN
YPK_2121	minC septum formation inhibitor	DOWN	-	UP	-	UP	-
pYV0073	yscT, type III secretion protein	DOWN	DOWN	-	-	DOWN	-
YPK_2643	hypothetical protein	DOWN	UP	DOWN	DOWN	-	-
YPK_4220	F0F1 ATP synthase subunit A	DOWN	-	UP	-	-	DOWN
YPK_0507	lipopolysaccharide transport periplasmic proteinLptA	DOWN	-	UP	-	UP	-
YPK_3319	acireductone dioxygenase ARD	DOWN	-	UP	-	-	-
YPK_2094	hypothetical protein	DOWN	-	DOWN	-	-	-
YPK_1921	hypothetical protein	DOWN	-	-	-	-	UP
YPK_2251	hypothetical protein	DOWN	-	-	-	DOWN	-
YPK_1976	3-hydroxy acid dehydrogenase	DOWN	-	UP	-	-	-
pYV0062	yscX, type III secretion protein	DOWN	-	UP	-	-	UP
YPK_1945	methylated-DNA--protein-cysteinemethyltransferase	DOWN	-	-	-	-	-
YPK_0771	hypothetical protein	DOWN	-	-	-	-	-
YPK_2198	hypothetical protein	DOWN	DOWN	-	-	UP	UP
YPK_3247	putative nucleotide-binding protein	DOWN	-	UP	-	-	DOWN
YPK_2291	transposase	DOWN	DOWN	-	-	UP	-
YPK_0031	hypothetical protein	DOWN	-	-	-	-	UP
YPK_2759	pH 6 antigen	DOWN	-	DOWN	-	UP	-
YPK_4138	preprotein translocase subunit SecB	DOWN	-	UP	-	-	-
YPK_2023	translation initiation factor Sui1	DOWN	-	-	-	-	-
YPK_4215	DNA-binding transcriptional regulator AsnC	DOWN	UP	-	-	-	-
YPK_3209	transposase IS200-family protein	DOWN	-	-	-	-	-
YPK_4194	engB ribosome biogenesis GTP-binding protein YsxC	DOWN	-	-	-	-	-
YPK_2164	cytoplasmic chaperone TorD family protein	DOWN	-	-	-	-	-

YPK_2027	hypothetical protein	DOWN	-	-	-	-	-
YPK_3390	cytochrome b561	DOWN	DOWN	-	-	DOWN	-
YPK_3463	mrcB penicillin-binding protein 1b	DOWN	-	-	-	UP	-
YPK_3120	putative bacteriophage protein	DOWN	-	-	-	-	-
pYV0094	yopH, protein-tyrosine phosphatase Yop effector	DOWN	DOWN	DOWN	-	-	UP
pYV0091	transposase	DOWN	DOWN	-	-	-	-
YPK_0274	sulfur transfer complex subunit TusB	DOWN	-	UP	-	-	-
YPK_0647	hypothetical protein	DOWN	-	-	-	DOWN	-
YPK_4177	rpoZ DNA-directed RNA polymerase subunit omega	DOWN	UP	UP	-	-	-
YPK_0525	rpsI 30S ribosomal protein S9	DOWN	-	UP	-	DOWN	DOWN
YPK_0501	phosphohistidinoprotein-hexosephosphotransferase component of N·	DOWN	-	UP	-	-	-
pYV0090	transposase	DOWN	-	DOWN	-	-	-
YPK_0909	hypothetical protein	DOWN	-	-	-	-	-
YPK_3929	hemG protoporphyrinogen oxidase	DOWN	-	-	-	-	-
YPK_1847	sufA iron-sulfur cluster assembly scaffold protein	DOWN	DOWN	-	-	-	-
YPK_2295	hypothetical protein	DOWN	-	-	-	-	-
YPK_3486	hypothetical protein	DOWN	-	-	-	-	UP
YPK_2879	hypothetical protein	DOWN	UP	DOWN	-	UP	UP
YPK_2971	sdhD succinate dehydrogenase cytochrome b556 smallmembrane s	DOWN	-	-	-	UP	DOWN
YPK_3108	DinI family protein	DOWN	-	-	-	-	-
pYV0079	yscC, type III secretion protein	DOWN	DOWN	-	-	-	-
YPK_3153	peptidyl-prolyl cis-trans isomerase B	DOWN	DOWN	UP	DOWN	DOWN	DOWN
YPK_3457	iron-sulfur cluster insertion protein ErpA	DOWN	-	-	-	-	-
pYV0082	yscF, type III secretion protein	DOWN	DOWN	UP	-	-	-
YPK_3722	XRE family transcriptional regulator	DOWN	-	-	-	-	-
pYV0049	hypothetical protein	DOWN	-	-	-	-	-
pYV0069	yscP, type III secretion protein	DOWN	DOWN	UP	-	-	-
pYV0017	resolvase	DOWN	-	-	-	-	-
YPK_3655	autoinducer-2 (AI-2) modifying protein LsrG	DOWN	-	DOWN	-	-	-
YPK_0284	rplD 50S ribosomal protein L4	DOWN	-	UP	UP	UP	-
YPK_1765	hypothetical protein	DOWN	-	DOWN	-	-	UP
YPK_0280	bacterioferritin-associated ferredoxin	DOWN	DOWN	DOWN	-	DOWN	DOWN
YPK_1807	transposase	DOWN	-	-	-	UP	-
YPK_0494	trehalose-6-phosphate hydrolase	DOWN	-	-	-	-	UP
YPK_3236	hypothetical protein	DOWN	-	UP	-	-	DOWN
pYV0039	transposase	DOWN	DOWN	-	-	DOWN	DOWN
YPK_0301	rpmD 50S ribosomal protein L30	DOWN	-	UP	UP	UP	UP
YPK_2088	DNA topoisomerase III	DOWN	-	-	-	-	-
YPK_3757	rplU 50S ribosomal protein L21	DOWN	-	UP	-	DOWN	DOWN
YPK_0299	rplR 50S ribosomal protein L18	DOWN	-	UP	UP	UP	UP
pYV0060	lcrD, yscV, membrane-bound Yop protein	DOWN	DOWN	UP	-	-	-
YPK_3885	coproporphyrinogen III oxidase	DOWN	DOWN	-	-	-	-
YPK_4154	rpmB 50S ribosomal protein L28	DOWN	-	UP	DOWN	DOWN	DOWN
YPK_2651	hypothetical protein	DOWN	-	-	-	-	-
pYV0007	replication protein	DOWN	UP	UP	-	DOWN	DOWN
pYV0054	yopD, Yop negative regulation/targetingcomponent	DOWN	DOWN	UP	-	-	-
YPK_0554	glucuronate isomerase	DOWN	-	-	-	-	-
YPK_4078	soluble pyridine nucleotide transhydrogenase	DOWN	-	-	-	-	-
YPK_1810	hypothetical protein	DOWN	-	-	-	-	-
YPK_1525	hypothetical protein	DOWN	-	-	-	-	-
YPK_1194	iron-sulfur cluster-binding protein	DOWN	-	UP	DOWN	DOWN	DOWN
YPK_3445	superoxide dismutase	DOWN	-	-	-	UP	-
YPK_3889	TonB-dependent heme/hemoglobin receptor familyprotein	DOWN	-	-	-	-	-
YPK_2252	iron permease FTR1	DOWN	DOWN	DOWN	-	DOWN	-
YPK_2789	ABC transporter-like protein	DOWN	-	UP	-	UP	-
pYV0001	ypkA, targeted effector protein kinase	DOWN	-	UP	-	-	-
YPK_0269	hypothetical protein	DOWN	-	-	-	-	-
YPK_0520	protease Do	DOWN	-	-	-	UP	-
pYV0002	hypothetical protein	DOWN	DOWN	UP	-	-	-
YPK_1858	cyclopropane-fatty-acyl-phospholipid synthase	DOWN	DOWN	DOWN	-	-	UP
YPK_2831	outer membrane protease	DOWN	DOWN	-	-	-	-
YPK_1688	3-oxoacyl-(acyl carrier protein) synthase II	DOWN	-	UP	-	-	-
YPK_1031	tas putative aldo-keto reductase	DOWN	-	UP	-	UP	UP
YPK_3795	FtsH protease regulator HflC	DOWN	-	UP	-	-	-
pYV0080	yscD, type III secretion protein	DOWN	DOWN	-	-	-	-
YPK_3338	efflux pump membrane protein	DOWN	-	-	-	UP	-
YPK_2565	galactose/methyl galaxtoside transporterATP-binding protein	DOWN	-	-	-	-	DOWN
YPK_3388	catalase/peroxidase HPI	DOWN	-	DOWN	-	DOWN	UP
YPK_3949	protein tyrosine/serine phosphatase	DOWN	-	-	-	-	-
YPK_1177	ankyrin	DOWN	-	-	-	-	UP
YPK_3452	serine endoprotease	DOWN	-	-	UP	UP	UP
YPK_1375	extracellular solute-binding protein	DOWN	-	DOWN	UP	UP	-
YPK_1364	phosphoribosylaminoimidazole-succinocarboxamidesynthase	DOWN	-	UP	-	DOWN	-
YPK_0320	putative ribosome maturation factor	DOWN	UP	-	-	-	-
pYV0083	yscG, type III secretion protein	DOWN	DOWN	UP	-	-	-
pYV0087	yscK, type III secretion protein	DOWN	DOWN	UP	-	DOWN	-
YPK_3194	adk adenylate kinase	DOWN	-	UP	-	-	DOWN
YPK_2634	3-hydroxydecanoyl-ACP dehydratase	DOWN	-	UP	-	-	-
pYV0057	lcrV, V antigen, antihost protein/regulator	DOWN	DOWN	UP	-	DOWN	-
pYV0070	type III secretion system protein	DOWN	DOWN	UP	-	-	-
YPK_2197	hypothetical protein	DOWN	DOWN	-	-	UP	UP
YPK_3713	N-acetyltransferase GCN5	DOWN	-	-	-	-	-
YPK_2199	hypothetical protein	DOWN	DOWN	-	-	UP	UP
pYV0055	yopB, Yop targeting protein	DOWN	DOWN	UP	UP	-	UP
YPK_2974	heat shock protein GrpE	DOWN	-	UP	-	-	DOWN

pYV0092	transposase	DOWN	DOWN	-	-	-	-
YPK_0272	sulfur transfer complex subunit TusD	DOWN	UP	-	-	-	-
pYV0047	yopM, targeted effector protein	DOWN	DOWN	-	-	-	-
pYV0098	yopP, yopJ, targeted effector protein	DOWN	DOWN	-	-	-	-
pYV0071	type III secretion system protein	DOWN	DOWN	-	-	-	-
YPK_2795	50S ribosomal protein L25	DOWN	-	UP	-	-	DOWN
pYV0020	sycH, yopH targeting protein	DOWN	-	-	-	DOWN	-
YPK_0521	cytochrome d ubiquinol oxidase subunit III	DOWN	UP	UP	DOWN	DOWN	DOWN
YPK_1569	NADH dehydrogenase subunit K	DOWN	-	UP	-	-	-
YPK_4139	glutaredoxin 3	DOWN	-	UP	-	-	-
YPK_2200	hypothetical protein	DOWN	DOWN	DOWN	DOWN	UP	UP
pYV0088	type III secretion system protein	DOWN	DOWN	UP	-	-	-
YPK_0636	rpsU 30S ribosomal protein S21	DOWN	UP	UP	DOWN	DOWN	DOWN
pYV0040	yop targeting protein yopK, yopQ	DOWN	DOWN	-	DOWN	DOWN	DOWN
pYV0086	yscJ, ylpB, type III secretion lipoprotein	DOWN	DOWN	UP	-	-	-
pYV0063	sycN, type III secretion protein	DOWN	-	UP	-	UP	UP
pYV0015		DOWN	DOWN	-	-	-	-
pYV0068	yscO, type III secretion protein	DOWN	DOWN	UP	-	-	-
pYV0078	hypothetical protein	DOWN	DOWN	-	-	DOWN	-
pYV0089	yscM, lcrQ, type III secretion regulatory	DOWN	DOWN	-	DOWN	DOWN	-
YPK_3389	cytochrome b562	DOWN	DOWN	DOWN	-	DOWN	-
pYV0084	yscH, yopR, lcrP, type III secretion protein	DOWN	DOWN	UP	-	-	-
pYV0085	yscI, lcrO, type III secretion protein	DOWN	DOWN	UP	-	-	-
pYV0058	lcrG, Yop regulator	DOWN	DOWN	UP	-	DOWN	-
pYV0053	hypothetical protein	DOWN	DOWN	-	-	-	-
pYV0064	tyeA, Yop secretion and targeting protein	DOWN	DOWN	-	-	UP	UP
pYV0081	yscE, type III secretion protein	DOWN	DOWN	UP	-	-	-
pYV0056	lcrH, sycD, low calcium response protein H	DOWN	DOWN	UP	-	-	-
