# Supplementary material for: Reprogramming of Yersinia from Virulent to Persistent Mode Revealed by Complex In Vivo RNA-seq Analysis
Source: PLoS Pathog. 2015 Jan 15;11(1):e1004600. doi: 10.1371/journal.ppat.1004600 (PMC4295882; doi:10.1371/journal.ppat.1004600)
Supplement: S6 Table — Differences (p-values; Fisher’s Exact Test) in clearance of infection between wt and indicated mutants at different time points (1–42 dpi) of infection (DOCX) [file ppat.1004600.s013.docx]

**Table S6.** The difference between wt and indicated mutants in clearance according to *p*-values

| **dpi** | ***p-*value** | | | | |
| --- | --- | --- | --- | --- | --- |
|  | ***arcA*** | ***fnr*** | ***frdA*** | ***wrbA*** | ***rovA*** |
| 1 dpi | 1 | 1 | 1 | 1 | 0.0001 |
| 3 dpi | 1 | 1 | 1 | 1 | 0.0001 |
| 7 dpi | 0,4063 | 1 | 1 | 1 | 0.0001 |
| 10 dpi | 0,4063 | 1 | 0,4063 | 1 | 0.0001 |
| 15 dpi | 0,3565 | 1 | 0,3565 | 1 | 0.0001 |
| 22 dpi | 0,0114 | 0,5419 | 0,1638 | 0,3508 | 0.0001 |
| 29 dpi | 0,0519 | 0,2169 | 0,1138 | 0,2661 | 0.0001 |
| 36 dpi | 0,0145 | 0,0407 | 0,1859 | 0,065 | 0.0001 |
| 42 dpi | 0,0091 | 0,0744 | 0,2776 | 0,0202 | 0.0001 |
