# Supplementary material for: Reprogramming of Yersinia from Virulent to Persistent Mode Revealed by Complex In Vivo RNA-seq Analysis
Source: PLoS Pathog. 2015 Jan 15;11(1):e1004600. doi: 10.1371/journal.ppat.1004600 (PMC4295882; doi:10.1371/journal.ppat.1004600)
Supplement: S7 Table — All the strains used in this study indicated with genotypes and references. (DOCX) [file ppat.1004600.s014.docx]

**Table S7.** Strains used in this study

| **Strain** | **Genotype** | **Reference** |
| --- | --- | --- |
| *E. coli* | | |
| DH5αλpir | F^−^φ80 ΔlacZ ΔM15 endA1 recA1 hsdR17(r_K_ ^−^ m_K_ ^+^)supE44 thi-1 λ^−^ gyrA96 relA1Δ |  |
| S17-1λpir | *RP4-2(Km::Tn7,Tc::Mu-1)*, *pro-82*, *LAMpir*, *recA1*, *endA1*, *thiE1*, *hsdR17*, *creC510* | Milton et al., 1992 |
| *Y. pseudotuberculosis* YPIII | | |
| YPIII/pIBX (originally called Xen4) | *putative transposase*(*pYV0017)*::Tn5*﷽﷽﷽﷽﷽﷽﷽﷽     luxCDAB,* Km^R^ | Caliper Life Sciences, Inc. |
| YPIII,∆*hdeB/*pIBX | *hdeB (YPK_1140)*_,_ Km^R^ | This study |
| YPIII,∆*fnr/*pIBX | *fnr* *(YPK_1944),* Km^R^ | This study |
| YPIII,∆*frdA/*pIBX | *frdA*_,_ *(YPK_3813),* Km^R^ | This study |
| YPIII,∆*arcA/*pIBX | *arcA*_,_ *(YPK_3606),* Km^R^ | This study |
| YPIII,∆*uspA/*pIBX | *uspA (YPK_0120)*_,_ Km^R^ | This study |
| YPIII,∆*napA/*pIBX | *napA (YPK_1387)*_,_ Km^R^ | This study |
| YPIII,∆*wrbA/*pIBX | *wrbA (YPK_2363)*_,_ Km^R^ | Wang et al., 2011 |
| YPIII,∆*motB/*pIBX | *motB (YPK_0802)*_,_ Km^R^ | This study |
| YPIII,∆*cheW/*pIBX | *cheW (YPK_1750)*_,_ Km^R^ | This study |
| YPIII,∆*rovA/*pIBX | *rovA (YPK_1876)*_,_ Km^R^ | This study |
| YPIII,∆*fliC/*pIBX | *fliC (YPK_2381)*_,_ Km^R^ | This study |
